# Sesquiterpene Lactones and Flavonoid from the Leaves of Basin Big Sagebrush (*Artemisia tridentata* subsp. *tridentata*): Isolation, Characterization and Biological Activities

**DOI:** 10.3390/molecules29040802

**Published:** 2024-02-09

**Authors:** Rosemary Anibogwu, Karl De Jesus, Samjhana Pradhan, Shanae Van Leuven, Kavita Sharma

**Affiliations:** 1 Department of Chemistry, Idaho State University, Pocatello, ID 83209, USA; anibrose@isu.edu (R.A.); dejekarl@isu.edu (K.D.J.); samjhanapradhan@isu.edu (S.P.); shanaevanleuven@isu.edu (S.V.L.); 2 Biomedical and Pharmaceutical Sciences, Kasiska Division of Health Sciences, College of Pharmacy, Pocatello, ID 83209, USA

**Keywords:** 60 cell line study, antioxidant activity, bioactive components, desacetylmatricarin, leucodin, matricarin, melanoma, quercetagetin 3,6,4′-trimethyl ether

## Abstract

This research is an exploratory study on the sesquiterpenes and flavonoid present in the leaves of *Artemisia tridentata* subsp. *tridentata*. The leaf foliage was extracted with 100% chloroform. Thin-layer chromatography (TLC) analysis of the crude extract showed four bands. Each band was purified by column chromatography followed by recrystallization. Three sesquiterpene lactones (SLs) were isolated—leucodin, matricarin and desacetylmatricarin. Of these, desacetylmatricarin was the major component. In addition, a highly bio-active flavonoid, quercetagetin 3,6,4′-trimethyl ether (QTE), was also isolated. This is the first report on the isolation of this component from the leaves of *Artemisia tridentata* subsp. *tridentata*. All the components were identified and isolated by TLC, high-performance liquid chromatography (HPLC) and mass spectrometry (MS) techniques. Likewise, the structure and stereochemistry of the purified components were characterized by extensive spectroscopic analysis, including 1D and 2D nuclear magnetic resonance (NMR) and Fourier transform infrared spectroscopy (FTIR) studies. The antioxidant activities of crude extract were analyzed, and their radical-scavenging ability was determined by Ferric reducing antioxidant power (FRAP) and 2,2-diphenyl-1-picrylhydrazyl (DPPH) assay. The crude extract showed antioxidant activity of 18.99 ± 0.51 and 11.59 ± 0.38 µmol TEg^−1^ FW for FRAP and DPPH assay, respectively, whereas the activities of matricarin, leucodin, desacetylmatricarin and QTE were 13.22, 13.03, 14.90 and 15.02 µmol TEg^−1^ FW, respectively, for the FRAP assay. The antitumor properties were probed by submitting the four isolated compounds to the National Cancer Institute (NCI) for NCI-60 cancer cell line screening. Overall, the results of the one-dose assay for each SL were unremarkable. However, the flavonoid’s one-dose mean graph demonstrated significant growth inhibition and lethality, which prompted an evaluation of this compound against the 60-cell panel at a five-dose assay. Tests from two separate dates indicate a lethality of approximately 75% and 98% at the log^−4^ concentration when tested against the melanoma cancer line SK-Mel 5. This warrants further testing and derivatization of the bioactive components from sagebrush as a potential source for anticancer properties.

## 1. Introduction

*Artemisia tridentata* subsp. *Tridentata,* commonly known as the Basin big sagebrush, is the tallest shrub in the *Artemisia* genus [1]. These plant species dominate a large portion of the western U.S., mainly in the regions that are too dry to support forest or grassland [2]. *Artemisia* is widely distributed and considered the largest member of the Asteraceae (Sunflower) family [3]. As they are rich in bioactive components, such as terpenes, sesquiterpene lactones (SLs), phenolic acids, flavonoids, sterols, fatty acids, lignans and acetylenes, among others [4], these plant species have garnered huge attention in pharmaceutical [5] and food industries [6]. *Artemisia* is an aromatic shrub native to a variety of habitats and climates which range from cold desert conditions to the intermountain states of the United States. It encompasses 43 million ha of semi-arid lands in the western United States [7]. The use of *Artemisia* species in traditional medicine is well known and demonstrates the great ethno-pharmacological value of this genus [8]. It has the capacity to alleviate human afflictions such as internal bleeding, headaches, external infections and respiratory malfunctions. It also has antitumor properties [9]. These compounds have been shown to possess important medicinal properties with uses as an antiseptic, digestive, disinfectant, ophthalmic and sedative [10]. Besides their application in traditional medicine, they also have high food value where most of the species have been used either as food, spices, condiments or beverages [11]. Leaves and seeds from Basin big sagebrush are also reported to be consumed. Leaves can be cooked as a condiment and also infused in water to make tea with a sage-like flavor [12], whereas seeds can be roasted, ground into powder and consumed with water [13,14,15]. Because sagebrush has high glyceride fat content, it is an excellent source of feed for animals [12]. 

The easy availability of sagebrush has a wide scope in the field of pharmaceuticals. In particular, plant extracts that are rich in SLs and flavonoids have gained considerable attention for treating human diseases. SLs are highly prevalent in the Asteraceae family and are probably the largest class of secondary metabolites in plants, with over 5000 structures reported to date [16]. They constitute a diverse set of biologically active compounds and have been reported to show a wide range of biological activities, including cytotoxic, anti-inflammatory, antifungal, antibacterial, antimalarial and phytotoxic properties [17]. SLs in cancer clinical trials have properties that enable them to target tumor and cancer stem cells while sparing normal ones [18]. The structural diversity in SLs is attributed to the presence of a γ-lactone ring with an α-methylene group (αMγL) and an α, β-unsaturated cyclopentenone ring. Additionally, the αMγL group acts as a Michael acceptor and is responsible for the underlying mechanism of the action of SLs. αMγL reacts with nucleophiles in proteins, enzymes and transcription factors to generate an alkylating product. Due to the steric effect, these alkylated products disrupt the biological functions of macromolecules and exert their cytotoxic activity [17]. 

**Figure 1:**
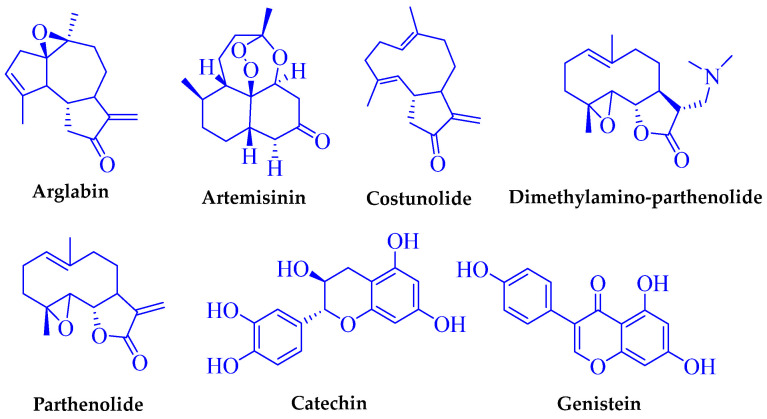
Typical sesquiterpene lactone and flavonoid-based drugs.

Similarly, flavonoids also possess a broad range of biological activities such as antioxidant, anticancer and anti-inflammatory properties, among others [19]. Due to these beneficial properties, they have a crucial role in maintaining our health. The biological properties of any plant species are also dependent on the type of secondary metabolites, the mode of action and their bioavailability [20]. In this regard, a wide variety of flavonoids from the *Artemisia* species have been reported on mediating antioxidant and anticancer activities [21,22,23]. Examples of SLs and flavonoid drugs and their derivatives in clinical trials are presented in Figure 1.

From time immemorial, plant-derived extracts, natural products and their structural analogues have been used for treating ailments. They have earned a central role in drug discovery which is mainly attributed to their complex and diverse structural arrangements. Naturally, these scaffolds are structurally optimized to perform specific biological functions and have occupied a special place in the fields of nutrition, pharmacology and agronomy, among others [24]. Despite their unique features and functionalities, their applications are limited by the complex process of isolation, characterization and structural elucidation. In this regard, it is highly crucial to conduct systematic research that focuses on the extraction, isolation, purification and characterization of bioactive compounds from various natural resources. Due to the diverse structure of bioactive components, the *Artemisia* (sagebrush) species have gained special attention in food and medicinal applications. Although the essential oil composition of *Artemisia* aerial parts has been investigated [25], full extensive characterizations of the components isolated from the plant extracts are scarce. Previous reports of sesquiterpene lactones [26,27] and polyphenolic compounds [28] have been reported but they lack an in-depth study on the structural characterization, stereochemistry assignments and biological studies. One of the crucial steps in natural product drug discovery is structural elucidation, which not only provides elaborate information on the chemical composition of the molecules but also on their characteristics, novelty and derivatization potential. Thus, with this objective, in the present study, we aim to justify the structure and stereochemistry of the sesquiterpene lactones and flavonoids, isolated from the foliage of Basin big sagebrush (*Artemisia tridentata* subsp. *tridentata*), through extensive 1D and 2D NMR techniques. Antioxidant and anticancer studies have also been conducted to evaluate the biological properties of the purified components. To the best of our knowledge, this is the first report on the identification of quercetagetin 3,6,4′-trimethyl ether (QTE) and the first detailed explanation of the structure and stereochemistry of the components isolated from the foliage of Basin big sagebrush along with the evaluation of their biological activities.

## 2. Results

### 2.1. Thin-Layer Chromatography (TLC) Analysis

TLC analysis of the chloroform extract was carried out as a preliminary step for screening the secondary metabolites present in the crude extract. Using 30% ethyl acetate in hexane, the crude extract showed a purple band at retention factor (Rf) 0.25, 0.5 and 0.64, and a grey band at Rf 0.74, which corresponded to the bands of Leucodin (**1**), Matricarin (**2**), QTE (**3**) and Desacetylmatricarin (**4**), respectively, at 254 nm (Figure S1, Supplementary Data S1).

### 2.2. Reversed-Phase High-Performance Liquid Chromatography (RP-HPLC) Analysis

HPLC analysis further confirmed the presence of the above components in the crude extract. The results garnered from the HPLC chromatogram, as shown in Figure S2 (Supplementary Data S1), demonstrate the presence of five distinct components, one of which has not been identified (15.789 min). These results are consistent with the fundamental principles of separation based on polarity. Desacetylmatricarin was the most polar compound and was eluted from the column after 8.457 min. QTE was eluted after 13.166 min. Following QTE, matricarin was obtained at 14.548 min. And finally, leucodin, the least polar compound, was eluted from the column at about 17.093 min.

### 2.3. Liquid Chromatography Mass Spectrometry (LC-MS) Analysis

LC-MS study was conducted for the mass analysis of the components present in the crude extract. And accordingly, the components were identified on the basis of their molecular weight and fragmentation pattern. Four isolated compounds were identified in the crude extract as matricarin at retention time (RT) 0.34 min with (*m*/*z*) 305.13524 g/mol and a calculated mass of 304.13090; Leucodin at RT 3.91 min with (*m*/*z*) 247.13293 g/mol and a calculated mass of 246.12546 g/mol; Desacetylmatricarin at RT 1.40 min with (*m*/*z*) 263.13348 g/mol and a calculated mass of 262.12036 g/mol; quercetagetin trimethyl ether at RT 6.26 with *m*/*z* 361.09176 g/mol and a calculated mass of 362.00 g/mol. Total ion chromatogram (TIC) and extracted ion chromatogram (EIC) are provided in the Supplementary Materials (Supplementary Data S2-LCMS).

### 2.4. Nuclear Magnetic Resonance (NMR) and Fourier Transform Infrared Spectroscopy (FTIR) Studies

**Figure 2:**
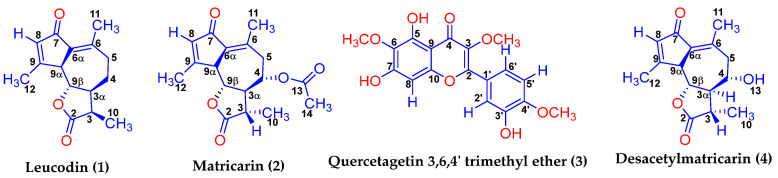
Structure of the isolated compounds with labeled protons, carbons and specified stereochemistry.

In order to confirm the structure of the pure isolated components (Figure 2), extensive FTIR and NMR (1D and 2D) studies were conducted. FTIR helped to determine the fingerprints of specific functional groups, whereas 1D and 2D NMR studies helped to elucidate and confirm the detailed structure and stereochemistry of the pure components isolated from the leaves of Basin big sagebrush.

#### 2.4.1. Matricarin (**2**)

The IR analysis of matricarin (Figure S3, Supplementary Data S1) revealed five bands of interest to the goal of structural elucidation. The ester in the five-membered ring was identified to be the sharp peak at 1782 cm^−1^. The abnormally high frequency of this cyclic ester can be attributed to the ring strain, thus more energy would be required to stretch it. The acyclic ester of C13 falls within the normal range of an ester with a sharp peak at 1739 cm^−1^. Because conjugation lowers the frequency of absorption and thereby makes bonds easier to stretch, the carbonyl on C7 was associated with the signal at 1681 cm^−1^. Two alkene groups were also distinguished in the IR. The alkene between C8 and C9 was assigned to the narrow peak at 1618 cm^−1^ while the alkene between C6 and C6α was correlated to 1637 cm^−1^. 

^1^H NMR was a great asset as it enabled the attainment of valuable pieces of information like chemical shift, integration, multiplicity and coupling constants. Through the coordination of these data, the stereochemistry of matricarin was determined. Table 1 and Table 2 represent all proton and carbon correlations for matricarin, respectively. For our ease of understanding in the structural assignment, Compound **2**, subsequently characterized as matricarin, was used for the initial stereochemical assignment. This compound not only has the advantage of a simpler ^1^H NMR spectrum, lacking Compound 1’s second proton at C4, but also a greater spread of signals that allows for first-order analysis of protons at C4 and C9β.

After assignment of all the protons and carbons using the HETCOR, COSY and HMBC data shown below (Table 3, Table 4 and Table 5, respectively), the stereochemical analysis was performed, starting with H9α. With a chemical shift of 3.416 ppm, H9α was found as a broad, not a sharp doublet. The source of its breadth was revealed by the COSY spectra (Table 4) which indicated it coupled long range with H8, H11 and H12, with a coupling constant that was unresolved by the instrument. The 10.0 Hz coupling constant shared with H9β indicated a dihedral angle approaching 180°, according to the Karplus curve, and was consistent with an anti or trans-stereochemical relationship between H9α and H9β. H9β, for its part, was a doublet of doublets with 10.3 and 10.0 Hz coupling constants resulting from coupling to H3α and H9β, respectively. The magnitude of the coupling constant with H3α also indicated an anti or trans-stereochemical relationship between the two protons. 

The signals for H3α were not first order; some of the peaks were concealed by the methyl singlet at 2.349 ppm and encroached upon by H5α at 2.375 ppm. Therefore, its stereochemical assignment was made by using the corresponding coupling constants to adjacent protons, H3 and H4. To complete the stereochemistry of the azulene ring, H4 at 4.830 ppm was analyzed next. This was found to be a triplet of doublets with coupling constants of 10.7 and 2.1 Hz, respectively. The triplet with large (10.7 Hz) coupling constants is consistent with anti or trans-stereochemistry with one of the protons in C5, H5α and H3α. The smaller (2.1 Hz) coupling constant is consistent with syn or cis stereochemistry between H5β and H4. 

To verify this, the coupling constants of H5α and H5β were considered. H5α appeared as a doublet of doublets at 2.726 ppm with coupling constants of 13.4 and 11.0 Hz. The former is an outsized coupling constant corresponding to the coupling between H5α and H5β, which are diastereotopic. The latter is due to the coupling between H5α and H4. The coupling constant of 11.0 Hz is slightly larger than the 10.7 Hz indicated by the triplet coupling constant for H4. Perhaps this is the result of a lack of resolution for the instrument on the triplet. Nevertheless, its magnitude verifies there is anti or trans-stereochemistry between the two protons. The fact that the 13.4 Hz coupling constant is due to coupling between diastereotopic protons is demonstrated in the signals for H5β. These appeared at 2.375 ppm as a doublet of doublets with coupling constants of 13.4 and 2.1 Hz. The small (2.1 Hz) coupling constant indicates syn or cis stereochemistry between H5β and H4. As a result, the stereochemistry for protons H9α, H9β, H3α and H4 is trans throughout the ring. 

The only stereochemistry remaining to be assigned is that of H3. This proton appeared at 2.458 ppm as a doublet of quartets with coupling constants of 12.0 and 6.9 Hz, respectively. The large (12.0 Hz) coupling constant indicates anti or trans-stereochemistry between H3 and H3α, while the 6.9 Hz coupling constant is in the normal range for freely rotating methyl groups. Had the H3 proton been syn or cis to H3α, this would have corresponded to the achillin skeleton, but the trans relationship between the two protons establishes Compound **2** as matricarin. As further proof, a change in NMR solvent to acetone-d6 did spread out the H3, H3α, H5α, H5β, H11 and H12 signals (Table S2, Supplementary Data S1). Near first-order signals were seen for H3α at 2.568 ppm as a doublet of triplets with coupling constants of 12.0 and 10.0 Hz, respectively. Thus, establishing the all-trans stereochemistry of H3α with protons H9β, H4 and H3.

#### 2.4.2. Desacetylmatricarin (**4**)

The IR analysis of desacetylmatricarin (Figure S4, Supplementary Data S1) demonstrates close similarity to matricarin, differing primarily due to the absence of the acyclic ester at Position 13 (Figure 2). The hydroxy group at Position 13 is denoted by a broad peak observed at 3423 cm^−1^. Additionally, the ester in the five-membered ring is distinctly characterized by a sharp peak at 1764 cm^−1^. The carbonyl peak at C7 appears at 1675 cm^−1^. Two alkene functional groups were identified within the IR spectrum. The alkene between C8 and C9 is represented by a narrow peak at 1612 cm^−1^, while the alkene between positions C6 and C6α is correlated to a peak at 1634 cm^−1^. After the assignment of all the protons and carbons using the ^1^H NMR, ^13^C NMR, HETCOR, COSY and HMBC data shown below from Tables S1–S5 (Supplementary Data S1), respectively, the stereochemical analysis was performed as above starting with H9α. As was the case for matricarin, Compound **2**, H9α was found as a broad, not a sharp, doublet, this time at 3.554 ppm. Its broad nature implied there was long-range coupling, which was verified by COSY NMR. Curiously, the COSY spectrum indicated, in addition to the expected long-range coupling with H8, coupling with H5α and H5β. The doublet’s 10.0 Hz coupling with H9β indicated an anti or trans-stereochemical relationship.

For 4, H9β appeared as a triplet at 3.702 with a 10.0 Hz coupling constant. This resulted from identical coupling constants for its coupling with H3α and H9β. The magnitude of the coupling constant once again indicated an anti or trans-stereochemical relationship between the two protons. Unfortunately, in this case, the signals for H3α were buried within those of neighboring protons, preventing any analysis of the multiplet at 2.219 ppm. There was no improvement, as was seen for matricarin above when the NMR solvent was changed. Therefore, its stereochemical assignment was performed after analysis of H4 and H3. H4 appeared as a triplet of doublets at 3.730 ppm. The triplet with a coupling constant of 10.3 Hz resulted from the equivalent coupling of H4 to H5α and H3α, indicating an anti or trans-stereochemistry between them. The instrument’s resolution was not able to differentiate between the 2.0 Hz coupling constant between H5β and H4 and the reciprocal 1.7 Hz coupling constant between H4 and H5β. Regardless, both are small coupling constants, indicating a syn or cis stereochemistry between the two protons. One can conclude, therefore, that the azulene ring has an all-trans stereochemistry between H9α, H9β, H3α and H4. 

The only remaining question is the stereochemical relationship between H3α and H3. A trans relationship would be characteristic of a matricarin skeleton, while a cis relationship would yield an achillin derivative. The doublet of quartets found at 2.615 ppm had coupling constants of 11.8 Hz between H3 and H3α and 6.9 Hz between H3 and the H10 methyl protons. The large (11.8 Hz) coupling constant established the stereochemical relationship between H3 and H3α as trans, thereby identifying Compound **4** as desacetylmatricarin.

#### 2.4.3. Leucodin (**1**)

The IR analysis of leucodin (Figure S5, Supplementary Data S1) highlights several key functional groups within the compound. The presence of the 5-membered lactone ring is evidenced by a distinctive peak observed at 1764 cm^−1^. Additionally, a carbonyl peak is registered at 1682 cm^−1^, indicative of a specific functional group in the compound. The IR spectrum also reveals the presence of two alkene functional groups. A narrow peak at 1612 cm^−1^ is attributed to the alkene between C8 and C9. Furthermore, another alkene group, possibly located between C6 and C6α, is represented by a peak at 1637 cm^−1^.

Having established Compounds **2** and **4** as matricarin and desacetylmatricarin, it would be logical to expect that these resulted from a common biosynthetic pathway that would also yield Compound 1. As such, the expectations were that Compound **1** shared the same stereochemical features and was leucodin, also known as desacetoxymatricarin (Figure 2). Assignment of ^1^H NMR, comparative ^1^H NMR and ^13^C NMR, HETCOR, COSY and HMBC data are presented in Tables S6–S11 (Supplementary Data S1), respectively. The compound’s nature hinged on the stereochemistry between H3 and H3α. A trans-stereochemistry would establish it as leucodin, and a cis stereochemistry as achillin. Unfortunately, the H3α proton signals were masked by the surrounding methyl signals. Nevertheless, all but one peak of the dq for H3 could be distinguished at 2.246 ppm. The H3 coupled to the methyl group with a normal 6.8 Hz coupling constant, but to H3α with a 13.2 Hz coupling constant, consistent with trans-stereochemistry. Curiously, the proton chemical shift was consistently 0.029 ppm lower than those reported by Martinez et al., 1988 [29] (Table S8, Supplementary Data S1). Nevertheless, they were substantially different from those of Achillin in CDCl_3_ at H3, H3α and H4α (Table S7, Supplementary Data S1) to definitively assign 1 as leucodin. This was further verified by comparison of the ^13^C NMR spectra. As shown in Table S8 (Supplementary Data S1), ^13^C data for Compound **1** were entirely consistent with Martinez et al.’s 1988 [29] ^13^C data for Leucodin, whereas there were significant differences with Achillin at C3α (56.41 vs. 51.9 d), C4 (26.04 vs. 23.6 d) and C10 (12.37 vs. 9.9 d).

#### 2.4.4. Quercetagetin 3,6,4′-Trimethyl Ether (**3**)

The IR for quercetagetin trimethyl ether (Figures S6–S8, Supplementary Data S1) was of limited utility because its high degree of conjugation made IR analysis somewhat perplexing. For instance, the band at 1655 cm^−1^ in the IR would typically indicate the presence of a regular alkene in a compound as alkenes tend to fall in the 1650 cm^−1^ region. However, the 1655 cm^−1^ peak actually specifies the presence of a ketone group at C4. Ketones are usually seen at around 1725 cm^−1^. This result of the conjugation present on both sides of the ketone serves to lower the frequency of absorption by about 30–40 cm^−1^ for each conjugation. Hence, the ketone on C4 (Figure 2) appears at 1655 cm^−1^. Additionally, this compound’s highly substituted aromatic rings significantly complicated the IR in the 700–900 cm^−1^ region, a region that would have been useful in determining the substitution pattern of the aromatic ring (ortho, meta or para-substitution). Finally, the broad peak seen at 3219 cm^−1^ corresponds to non-hydrogen bonding alcohols. Turning to NMR analysis, more concrete relationships were more discernable between the atoms of quercetagetin trimethyl ether.

Quercetagetin is also known as 6-hydroxy quercetin. The hydroxy/methoxy positions on the flavone backbone for Compound **3** were determined from the ^1^H NMR data summarized in Table S12, and ^13^C NMR and HMBC NMR summarized in Tables S13 and S14 (Supplementary Data S1), respectively. For assigning the aromatic protons, the lone singlet at 6.58 ppm was assigned as H8. The doublet at 7.10 ppm, J = 8.3 Hz, was assigned as H5′. This is consistent with the slight up-field shift resulting from the electron-donating methoxy group at adjacent C4′, a normal coupling constant to adjacent H6′, and a very small long-range coupling constant to H2′ at the para position—so small as to be rendered unresolved by the instrument. Although H6′ does experience a slight electronic shielding effect from the para hydroxy group at C3, the effect is not as great as for H5′ resulting in a more downfield chemical shift of 7.65. Zhanzhaxina et al. [30] and Cuong et al. [31] saw the same effects in their NMR analysis of quercetagetin 3,7,3′-trimethyl ether and quercetin, respectively. Moreover, its long-range coupling constant with the meta H2′ proton is large enough, 1.0 Hz, to yield a doublet of doublets. This is reflected in the doublet at 7.63 ppm for H2′. COSY NMR data (Supplementary Data S4-NMR) were not very useful as they were only able to distinguish a correlation between H5′ and H6′.

A NOESY experiment (Supplementary Data S4-NMR) was performed to ascertain the position of the methoxy groups. The only correlation of note besides that of the H5′ and H6′ protons was to the 3.93 ppm methyl group and H5′. This implied a methoxy substituent at C4′. The absence of a methoxy group correlation to H2′ and H8 meant no methoxy groups were present at C3′ or C7. Indeed, as indicated by Table S14 (Supplementary Data S1), HMBC NMR indicated methoxy methyl group correlations to ^13^C NMR signals at 132.0 ppm, 139.3 ppm and 150.0 ppm corresponding to C6, C3 and C4′, respectively. Key to the assignment of carbon signals were HMBC correlations to H8 at 6.60 ppm. H8 had correlations to 13C signals at 157.7, 153, 132.0 and 106.5 ppm. Comparison with 13C data for quercetagetin 3,7,3′-trimethyl ether and quercetin allowed these to be assigned as C7, C9, C6 and C10, respectively. The 13C signals for the aromatic ring substituent to C2 of the chromone core were similar to those found in quercetin and the quercetagetin derivative. In these, C3′ was always more upfield than C4′ with methoxy group substitution providing a downfield shift vs. hydroxy substitution. As a result, the 150.0 ppm signal was assigned to C4′ and the 147.4 ppm signal to C3′. Compound 4, therefore, proved to be quercetagetin 3,6,4′-trimethyl ether.

### 2.5. Antioxidant Assay

Antioxidant activities of the compounds isolated from *Artemisia tridentata* and the crude extract were investigated in terms of their radical-scavenging and reducing capacities. The scavenging capacities of crude and isolated compounds were evaluated using DPPH and FRAP assays. The scavenging activities of crude extracts were significant at 18.99 ± 0.51 µmol TEg^−1^ FW for FRAP and 11.59 ± 0.38 µmol TEg^−1^ FW for DPPH (Figures S9 and S10, Supplementary Data S1). The activities of matricarin, leucodin, desacetylmatricarin and quercetagetin 3,6,4′-trimethyl ether were 13.22, 13.03, 14.90 and 15.02 µmol TEg^−1^ FW, respectively, for the FRAP assay. However, there was less to report on DPPH assay results. Structural analysis of the active isolates confirmed our suspicion that the phenoxy groups on the aromatic ring are responsible for the radical-scavenging activity by donating hydrogen atoms to radicals as the hydroquinone rings are oxidized to quinones. Not surprisingly, given its quinoid structure, quercetagetin trimethyl ether, 3, showed the strongest activity.

### 2.6. NCI—60 Cell Lines Evaluation

**Figure 3:**
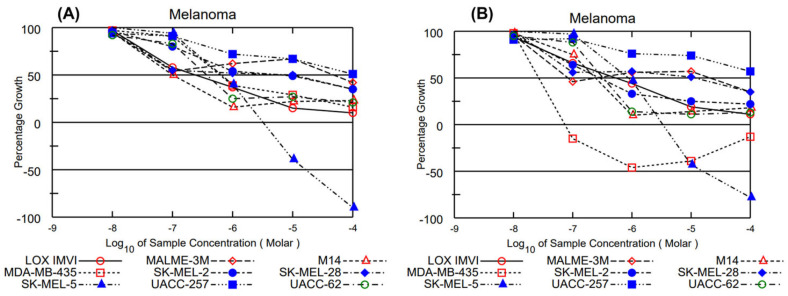
Five-dose assay of Quercetagetin trimethyl ether against melanoma at two separate dates: (**A**) 28 September 2020 and (**B**) 16 November 2020.

The results of the one-dose assay for each compound were reported as a mean graph of the percent growth inhibition, and the lethality of treated cells is provided in the supplementary materials (Supplementary Data S3-NCI). The reported number for each cell line in the one-dose assay represents growth relative to the no-drug control and to the time-zero number of cells. In this manner, the detection of growth inhibition (values between 0 and 100) and lethality (values less than 0) can be relayed. The graph for matricarin displayed a maximum growth inhibition of 89% in one of the ovarian cancer cell lines (OVCAR-3) and 28% cell lethality in one of the renal cancer cell lines (A498), but the results for this assay were overall unremarkable. Leucodin demonstrated maximum growth inhibition of 88% in one of the colon cancer cell lines (HT29) and 40% cell lethality in one of the renal cancer cell lines (A498), but the results for this assay were overall unremarkable. Desacetylmatricarin displayed growth inhibition of 83% in one of the breast cancer cell lines (BT-549) and 21% cell lethality in one of the CNS cancer cell lines (SNB-75), but the results for this assay were overall unremarkable. QTE’s one-dose mean graph demonstrated significant growth inhibition and lethality, which prompted an evaluation of this compound against the 60-cell panel at 5 concentration levels. The results of the five-dose assay for QTE were reported similarly to the one-dose assay. A series of 10-fold or ½ log serial dilutions of the compound being tested are made to provide a total of 5 drug concentrations plus control. Tests from 2 separate dates indicate lethality of approximately 75% (16 November 2020) and 98% (28 September 2020) at the log^−4^ concentration when tested against the melanoma cancer line SK-Mel 5 (Figure 3), which may require further testing with derivatization. The remainder of the results from the five-dose assay were unremarkable.

## 3. Discussion

Sesquiterpene lactones and the flavonoid, obtained from the leaves of *Artemisia tridentata*, have been identified, isolated and purified through chromatographic and mass spectroscopic techniques. The structure and stereochemistry of the purified components, viz, leucodin, matricarin, desacetylmatricarin and quercetagetin 3,6,4′-trimethyl ether, were assigned after IR, and extensive 1D and 2D NMR studies. This is the first report on the detailed structural elucidation with stereochemistry, and an attempt to evaluate the biological activities of SLs and the flavonoid from this plant species. Proper assignment of structure and stereochemistry is highly crucial as it provides knowledge on their potential biological activities, chemical composition, novelty and derivatization potential. Nonetheless, previous accounts are lacking a detailed and elaborative study to justify the structure, stereochemistry and biological properties of the isolated components from these plant species. In this regard, we have also identified a new flavonoid, quercetagetin 3,6,4′-trimethyl ether, for the first time from the leaves of Basin big sagebrush. Previous reports have accounted for polyphenolic compounds [28] with four previously unreported compounds (chlorogenic acid, axillarin, methyl axillarin and casticin) from this plant species. 

Flavonoids belong to a unique class of naturally occurring compounds that have proven to be an important source of chemically diverse novel metabolites [32]. They have the properties to scavenge reactive oxygen species and have also been reported to have cytotoxic effects on human cancer cells. A large number of studies have identified flavonoids, including gallic acid, catechine, rutin, cinnamic acid, quercetin and kaempferol, from several *Artemisia* species [21,22,33] and have found marked cytotoxic and antioxidant activities. Likewise, cytotoxic sesquiterpene lactones 8α-acetoxy-1,10α-epoxy-2-oxo-guaia-3,11(13)-dien-12,6α-olide and 13-acetoxy-1-oxo-4α-hydroxy-eudesman-2(11)-dien-12,6α-olide have been reported by Li et al. [34]. Monoterpenes 2,2-Dimethyl-6-isopropenyl-2*H*-pyran, 2,3-dimethyl-6-isopropyl-4*H*-pyran and 2-isopropenyl-5-methylhexa-trans-3,5-diene-1-ol were isolated from both *A. tridentata* ssp. *vaseyana* and *A*. *cana* ssp. *viscidula* and characterized by Gunawardena et al. [35]. Arglabin, another class of sesquiterpenes denominated as guaianolides, possesses promising antitumor activity against different tumor cell lines [36]. The molecule bears a 5,7,5-tricyclic ring system having five contiguous stereocenters in which the two five-membered rings are trans-annulated. According to a recent comprehensive review of sesquiterpenes [37], their biological activity has been ascribed to the α-methyl-γ-lactone group, or the unsaturated carbonyl moiety within the compound, both present in our compounds. Numerous studies on chemical modifications and total synthesis of natural sesquiterpenes have been carried out with the goal of producing biological active molecules for breast, kidney and prostate cancer treatments [38]. 

Antioxidant activity of *Artemisia* species has been confirmed in many studies and the majority of the identified compounds in this study were found to be powerful antioxidants that help neutralize harmful free radicals and prevent oxidative stress which damage cells and DNA [39]. Such activity was also strongly exhibited by leucodin, matricarin, desacetylmatricarin and QTE. Considering the high occurrence of sesquiterpenes found in our extract and the wide range of biological activities of these compounds in humans, the isolated compounds might contribute some anticancer, anti-inflammatory as well as anti-diabetic properties. As a result, the isolated compounds were analyzed for their antitumor activity by submitting the compounds to the National Cancer Institute’s 60 cancer cell line screen. It was found that quercetagetin trimethylether has the highest activity to all cell lines as compared to other isolated sesquiterpenes. The present study confirms that some of the flavonoids comprising a quinoid structure have much higher bioactivities, especially their anticancer and antioxidant properties, as compared to lactones containing sesquiterpenes.

## 4. Materials and Methods

### 4.1. Plant Collection

Aerial parts of Sagebrush were collected from Red Hill, Idaho State University (ISU), Pocatello, Idaho, in the month of May 2019. The collected plant materials were cleaned and shade-dried before initiating any extraction process. The plant species was identified by the Idaho Museum of Natural History, ISU.

### 4.2. Materials

All chemicals and solvents were commercially available and used as received without further purification. Analytical-grade reagents, such as hexane, chloroform, ethyl acetate and methanol, required for extraction and isolation of the components, were purchased from Fisher Scientific (Sumner, WA, USA). Standard chemicals, for instance, gallic acid, 6-hydroxy-2,5,7,8-tetramethylchroman-2-carboxylic acid (Trolox), 2,4,6-Tris (1-pyridyl)-5-triazine (TPTZ), ferric chloride and 2,2-diphenyl-1-picrylhydrazyl (DPPH), were also purchased from Fisher Scientific (Chelmsford, MA, USA). LC-MS-grade water and acetonitrile were purchased from Honeywell (Muskegon, MI, USA). 

### 4.3. Methods

#### 4.3.1. Extraction of Plant Material

Plant materials were shade-dried for two weeks before extraction. Drying was mainly performed to remove any excess water content from plants so that these could be stored for future experiments. Plant material was frozen using liquid nitrogen and subsequently crushed using a mortar and pestle. Twenty grams of the powdered sample was used for this experiment. The Soxhlet extraction technique was used to extract the phytochemicals from the powdered plant material with the use of 100% chloroform as the extracting solvent. Residues that were present in the extract were filtered, and the filtrate was concentrated using a high vacuum rotary evaporator. Thus, the obtained crude chloroform extract was further used for fractionation and isolation of the components. The average extraction yield of the crude extract was around 40%. 

#### 4.3.2. TLC Profile

The composition profile for each crude chloroform sample was determined using TLC. TLC was performed with different percentage concentrations of ethyl acetate and hexane to confirm the movement of compounds present in the crude chloroform extract. Silica gel 60 F254 and Silica gel 60 RP-18 F254S pre-coated plates (Merck, Darmstadt, Germany) were used for TLC. The spots were detected under a UV lamp (254 nm).

#### 4.3.3. Fractionation and Isolation of Crude Chloroform Extract by Column Chromatography

Column chromatography was carried out using silica gel (230–400 mesh) and RP-18 (230–400 mesh) obtained from Merck, Darmstadt, Germany. Based on TLC results, gravity silica gel column chromatography was performed to yield relatively pure compounds upon fraction collection. Approximately 8 gm of chloroform residue was mixed with silica, the mixture evaporated and then poured into a column filled with silica. This was then eluted using a hexane:ethyl acetate (7:3) solvent system to yield four fractions: viz, leucodin, matricarin, QTE and desacetylmatricarin, respectively. 

#### 4.3.4. Recrystallization of Isolated Components

The pure components obtained from column chromatography were recrystallized. Leucodin, matricarin and QTE were recrystallized using toluene while desacetylmatricarin was recrystallized in benzene.

#### 4.3.5. Total Antioxidant Assay

The total antioxidant activity of crude sagebrush extracts and purified components was estimated by using two standard assays: the Ferric reducing antioxidant power (FRAP) assay [40] and the DPPH assay [41]. Trolox was used as a standard for both experiments. Results are expressed in terms of µmol Trolox equivalent per gram fresh weight (µmol TEg^−1^ FW).

##### FRAP Assay

FRAP assay was performed in accordance with Benzie and Strain’s method with slight modification. The FRAP working reagent was prepared freshly by mixing 300 mM acetate buffer (pH 3.6), 10 mM TPTZ in 40 mM HCl and 20 mM FeCl_3_·H_2_O in a 10:1:1 ratio. The experiment was conducted at 37 °C under low pH (3.6) with a blank sample in parallel. For each assay, 2.90 mL of FRAP reagent and 100 µL of chloroform extract were mixed. The absorbance of the reaction (incubated at 37 °C) was measured at 593 nm after 30 min with a Shimadzu UV-1700 spectrophotometer. Similarly, for preparing the calibration curve, 100µL of different concentrations of Trolox (0.1 mg/mL, 0.05 mg/mL, 0.025 mg/mL, 0.125 mg/mL and 0.00625 mg/mL) was added to 2.9 mL of FRAP reagent.

##### DPPH Assay

The DPPH assay was carried out according to the method of Brand–Williams with some modifications. The working solution was prepared by diluting the DPPH solution with methanol to obtain an absorbance in the range of 1.1 ± 0.02 units at 515 nm. The diluted DPPH solution (950 µL) was mixed with a 50 µL blank, standards or sample extract; diluted with methanol (1:1, *v*/*v*) and incubated for 20 min. The absorbance was measured at 515 nm against the blank methanol by using a Shimadzu UV-1700 spectrophotometer. All results were expressed in terms of µmol TEg^−1^ FW. 

#### 4.3.6. National Cancer Institute (NCI)—60 Cell Line Evaluation

All four compounds isolated from *Artemisia tridentata* were submitted to the NCI to evaluate their antitumor properties. The compounds were solubilized in DMSO at 40 mg/mL for the 1-dose assay at a single high dose of 10^−5^ M in the full NCI 60-cell panel [42]. The compounds that met an established threshold inhibition criterion in a minimum number of cell lines were advanced to the full 5-dose assay in which they were evaluated against the 60-cell panel at 5 concentration levels. Of the four compounds isolated, only quercetagetin 3,6,4′-trimethyl ether was taken for the five-dose assay. For more details on the methodology used, please visit https://dtp.cancer.gov/discovery_development/nci-60/methodology.htm (accessed on 17 December 2020).

#### 4.3.7. General Instrumental Analysis Procedures

##### Reversed-Phase High-Performance Liquid Chromatography (RP-HPLC)

The crude chloroform extract was filtered using a 0.45 µm nylon membrane filter (Biotech, Rheinbrohl, Germany) prior to HPLC analysis. HPLC analysis of all extracts was carried out using an Agilent 1100 chromatograph (Agilent, Palo Alto, CA, USA) equipped with a solvent delivery system, an auto-sampler, a DAD detector set at 360 nm, and a ChemStation data acquisition system. Flavonoids were separated on a Zorbax Eclipse XDB C-18 column (250 mm × 4.6 mm) with a particle size of 5 µm (Agilent, Santa Clara CA, USA) protected with a Phenomenex (Torrance, CA, USA) C18-type guard column. The column was maintained at 25 °C. The mobile phase consisted of 0.1% TFA in water (solvent A) and methanol (solvent B). A gradient elution program was set as follows: 0–10 min, 20% B; 10–15 min, 20–80% B; 15–22 min, 80–20% B. The flow rate was 0.8 mL min^−1^, and the injected volume was 10 µL. The HPLC chromatogram was generated from the Waters Alliance 2695 HPLC System with Empower software, version 3.0. The identification of purified fractions was compared with the retention time and UV–visible spectra of the peaks present in the crude extract. The TLC analysis performed prior to the HPLC analysis indicated that leucodin, QTE and desacetylmatricarin could be easily resolved while matricarin may pose a separation challenge. Nonetheless, HPLC’s strong separation capability enabled the high resolution of all four compounds of interest.

##### LC-MS

Agilent 6545 LC-MS quadrupole time-of-flight (QTOF) was used to obtain mass spectrometry with processing performed using Mass Hunter software, version 10.1. The purified extracts from *Artemisia tridentata* were analyzed using an Agilent 1290 series LC with 6545 Q-TOF with a Jet Stream dual ESI interface (Agilent Technologies, Palo Alto, CA, USA). The LC was equipped with a vacuum degasser, a binary pump, a thermostat autosampler and a column compartment. The crude extracted was separated using a Kinetex Omega C-18 (2.1 × 100 mm, 3 μm; Phenomenex, Torrance, CA, USA, Agilent Technologies, Palo Alto, CA, USA). The mobile phase used was 0.1% formic acid in water and acetonitrile with the gradient method. The MS operating conditions were as follows: dry nitrogen temperature at 325 °C with a flow of 10 L/min; nebulizer pressure, 18 psi; sheath gas temperature at 400 °C with a flow of 12 L/min and capillary, nozzle, fragmentor, skimmer and octupole radiofrequency voltages of 4500, 500, 180, 45 and 750 V, respectively. LC-MS analysis of chloroform extracts of 1ug/mL concentration was performed in positive mode with a range of 100–1700 *m*/*z*. The data were analyzed with qualitative analysis navigator B.08.00 mass hunter software. 

##### NMR and FTIR

^1^H NMR, ^13^C NMR, heteronuclear multiple bond correlation (HMBC), heteronuclear multiple quantum correlation (HMQC) and H-H contraction of correlation spectroscopy (COSY) spectra were recorded on a JEOL ECX-300 NMR (JEOL, Akishima, Japan) and Bruker AscendTM 400 Spectrometer (Bruker, Billerica, MA, USA) using tetramethylsilane as an internal standard. To determine the structure of the four isolated compounds, a ^1^H NMR experiment was performed to acquire the characteristic chemical shift, integrations, multiplicities and coupling constants of protons. Subsequently, a ^13^C NMR was performed to determine the chemical shift of the carbons present. The distortionless enhancement by polarization transfer (DEPT) experiment was subsequentially carried out to determine the substitution pattern of carbons. Using heteronuclear correlation spectroscopy (HETCOR) provided insights into which proton and carbon groups are bonded to each other. Using DEPT in combination with HETCOR enabled the determination of multiplicity for the carbon atoms. The use of (COSY) and nuclear overhauser effect spectroscopy (NOESY) spectra to correlate protons to other protons verified the chemical environment. IR spectra were obtained on a Bruker TENSOR 37 FTIR spectrometer (Bruker, Billerica, MA, USA).

## 5. Conclusions

Sesquiterpene lactones and flavonoids have diverse structures and exhibit a wide range of biological activities, including antioxidant and anticancer properties. Despite their burgeoning application in food and medicine, their use has been limited by the complex process of isolation, purification and identification from plant species. In this regard, we have used both chromatographic and extensive spectroscopic techniques for the isolation, identification and structural elucidation of the SLs, viz leucodin, matricarin and desacetylmatricarin, and flavonoid quercetagetin 3,6,4′-trimethyl ether from the foliage of Basin big sagebrush (*Artemisia tridentata* ssp. *tridentata*). This is the first report on the expansive study of the justification of the structure and stereochemistry of the isolated components. Given the importance of their diverse structure, the purified components were subjected to antioxidant and anti-cancer studies. To assess the antioxidant properties of the bioactive compounds, DPPH and FRAP assays were performed. These assays confirmed that the isolated compounds contained antioxidant activity. In addition, the four compounds were submitted to the NCI to be tested against 60 human cell lines. The results from NCI showcased both growth inhibitory and lethality capabilities against cancer cells. In particular, QTE was potent against melanoma cancer line SK-Mel 5. Considering the significance of antioxidant and anti-tumor activity, QTE along with other extracted compounds can be chemically modified as their derivatives may hold potential features for medical applications. The derivatization of these compounds can be carried out to understand the structure–activity relationship (SAR) of the secondary metabolites isolated from the plant species.

## Figures and Tables

**Table 1:** Correlation of ^1^H NMR data of Compound **2**, Matricarin.

Protons	Chemical Shift (ppm)	Integration	Multiplicity	Coupling Constants (Hz)
H3	2.458	1H	dq	*J*3,3α =11.2 Hz; *J*3,10 =6.9 Hz
H3α	2.347	1H	m	
H4	4.830	1H	td	*J*4,3α/*J*4,5α = 10.7 Hz; *J*4,5β = 2.1 Hz
H5α	2.726	1H	dd	*J*5α,5β = 13.4 Hz; *J*5α,4 = 11.0 Hz
H5β	2.375	1H	dd	*J*5α,5β = 13.4 Hz; *J*5β,4 = 2.1 Hz
H8	6.179	1H	m	*J*6,12 = 1.8 Hz
H9α	3.416	1H	d	*J*9α,9β = 10.0 Hz
H9β	3.728	1H	dd	*J*9β,3α = 10.3 Hz; *J*9β,9α = 10.0 Hz
H10	1.340	3H	d	*J*10,3 = 6.9 Hz
H11	2.413	3H	bs	
H12	2.349	3H	bs	
H14	2.117	3H	s	

**Table 2:** ^13^C NMR and comparative BioRad data for Compound **2**, Matricarin.

^13^C NMR: CDCl3	Chemical Shift (ppm)	BioRad Spectra Base Comparison
C2	176.85	176.6
C3	40.73	40.7
C3α	59.10	59.1
C4	70.38	70.4
C5	44.75	44.7
C6	145.15	145.0
C6α	133.28	133.3
C7	195.27	195.1
C8	135.95	135.8
C9	169.73	169.6
C9α	51.59	51.6
C9β	81.12	81.1
C10	15.06	15.0
C11	21.44	21.1
C12	20.01	19.0
C13	169.82	169.7
C14	21.23	21.3

**Table 3:** HETCOR correlations for Compound **2**, Matricarin.

HETCOR (CDCl_3_)	Chemical Shift (ppm)	^13^C
H3	2.458	40.73
H3α	2.347	59.10
H4	4.830	70.38
H5α	2.726	44.75
H5β	2.375	44.75
H8	6.179	135.95
H9α	3.416	51.59
H9b	3.728	81.12
H10	1.340	15.06
H11	2.413	21.44
H12	2.349	20.01
H14	2.117	21.23

**Table 4:** COSY correlations for Compound **2**, Matricarin.

COSY (CDCl_3_)	Chemical Shift (ppm)	Correlations			
H3	2.458	2.347	1.340		
H3α	2.347	4.830	3.728	2.458	
H4	4.830	2.726	2.375	2.347	
H5α	2.726	4.830	2.726		
H5β	2.375	4.830	2.375		
H8	6.179	2.349	3.416 (wk)	2.413 (wk)	
H9α	3.416	3.728	6.179 (wk)	2.413 (wk)	2.347 (wk)
H9β	3.728	3.416	2.347		
H10	1.340	2.458			
H11	2.413	6.179	3.416		
H12	2.349	6.179	3.416		
H14	2.117				

wk = weak.

**Table 5:** HMBC correlations for Compound **2,** Matricarin.

HMBC (CDCl_3_)	Chemical Shift (ppm)	Correlations						
H3	2.458	176.85	59.10	15.06	70.38	20.01		
H3α	2.347	81.12	70.38	40.73	195.27	51.59	44.75	15.06
H4	4.830	169.82						
H5α	2.375	145.15	70.38	133.28	59.10	21.44		
H5β	2.726	145.15	70.38	133.28	59.10	21.44		
H8	6.179	195.27	169.73	133.28	51.59	20.01		
H9α	3.416	169.73	133.28	81.12	145.15 (wk)	135.95	59.10	
H9β	3.728	133.28	70.38					
H10	1.340	40.73	176.85	59.10				
H11	2.413	145.15	133.28	44.75				
H12	2.349	169.73		51.59				
H14	2.117	169.82	20.01					

wk = weak.

## Data Availability

Data are contained within the article and Supplementary Materials.

## Supplementary Materials

The following supporting information can be downloaded at https://www.mdpi.com/article/10.3390/molecules29040802/s1, Supplementary Data S1-Figures S1–S10 and Tables S1–S14. Supplementary Data S2-LCMS. Supplementary Data S3-NCI. Supplementary Data S4a-NMR-Scanned Files. Supplementary Data S4b-NMR-Raw Data.

## References

[1] Boyle S.A., Reeder D.R. (2005). Colorado Sagebrush: A Conservation Assessment and Strategy. https://cpw.state.co.us/Documents/WildlifeSpecies/Sagebrush/CHAPTER2overviewsagebrushecosystems.pdf.

[2] Briggs G.M. (2021). Inanimate Life. https://milnepublishing.geneseo.edu/botany/chapter/sagebrush/.

[3] Anibogwu R., Jesus K.D., Pradhan S., Pashikanti S., Mateen S., Sharma K. (2021). Extraction, isolation and characterization of bioactive compounds from *Artemisia* and their biological significance: A review. Molecules.

[4] Bora K.S., Sharma A. (2011). The genus *Artemisia*: A comprehensive review. Pharm. Biol..

[5] Bisht D., Kumar D., Kumar D., Dua K., Chellappan D.K. (2021). Phytochemistry and pharmacological activity of the genus *Artemisia*. Arch. Pharm. Res..

[6] Cui Z., Li S., Chang J., Zang E., Liu Q., Zhou B., Li C., Li M., Huang X., Zhang Z. (2022). The pharmacophylogenetic relationships of two edible medicinal plants in the genus *Artemisia*. Front. Plant Sci..

[7] West N.E., West N.E. (1983). Western Intermountain sagebrush steppe. Temperate Deserts and Semi-Deserts.

[8] Hussain M., Thakur R.K., Khazir J., Ahmed S., Khan M.I., Rahi P., Peer L.A., Pragadheesh V., Kaur S., Raina S.N. (2023). Traditional uses, phytochemistry, pharmacology, and toxicology of the genus *Artemisia*, L. (Asteraceae): A high-value medicinal plant. Curr. Top. Med. Chem..

[9] Erdogrul O.T. (2002). Antibacterial activities of some plant extracts used in folk medicine. Pharm. Biol..

[10] Jelodar N.B., Bhatt A., Mohamed K., Keng C.L. (2014). New cultivation approaches of *Artemisia* annua L. for a sustainable production of the antimalarial drug artemisinin. J. Med. Plant Resour..

[11] Trendafilova A., Moujir L., Sousa P.M.C., Seca A.M.L. (2020). Research advances on health effects of edible *Artemisia* species and some sesquiterpene lactones constituents. Foods.

[12] Kinney C.R., Sugihara J. (1943). Constituents of *Artemisia* tridentate (American Sage brush) II. J. Org. Chem..

[13] Allen G. (2010). The Herbalist in the Kitchen.

[14] Yanovsky E. (1936). Food Plants of the North American Indians.

[15] Moerman D.E. (2010). Native American Food Plants: An Ethnobotanical Dictionary.

[16] Amorim M.H.R., Gilda Costa R.M., Lopes C., Bastos M.M.S.M. (2013). Sesquiterpene lactones: Adverse health effects and toxicity mechanisms. Crit. Rev. Toxicol..

[17] Ivanescu B., Miron A., Corciova A. (2015). Sesquiterpene lactones from *Artemisia* Genus: Biological activities and methods of analysis. J. Anal. Methods Chem..

[18] Kawasaki B.T., Kalathur M., Ana M. (2009). Effects of the sesquiterpene lactone parthenolide on prostate tumor-initiating cells: An integrated molecular profiling approach. Prostate.

[19] Ullah A., Munir S., Badshah S.L., Khan N., Ghani L., Jaremko M., Emwas A. (2020). Important flavonoids and their role as a therapeutic agent. Molecules.

[20] Lh Y., Ym J., Shi J., Fa T., Datta N., Singanusong R., Ss C. (2004). Flavonoids in food and their health benefits. Plant Foods Hum. Nutr..

[21] Khan A.N., Dilshad E. (2023). Enhanced Antioxidant and Anticancer Potential of *Artemisia carvifolia* Buch Transformed with rol A Gene. Metabolites.

[22] Ferreira J., Luthria D.L., Sasaki T., Heyerick A. (2010). Flavonoids from *Artemisia annua* L. as antioxidants and their potential synergism with Artemisinin against malaria and cancer. Molecules.

[23] Matvieieva N., Drobot K., Duplij V., Ratushniak Y., Shakhovsky A.M., Kyrpa-Nesmiian T., Mickevičius S., Brindza J. (2019). Flavonoid content and antioxidant activity of *Artemisia vulgaris* L. “hairy” roots. Prep. Biochem. Biotechnol..

[24] Atanasov A.G., Zotchev S.B., Dirsch V.M., Supuran C.T. (2021). Natural products in drug discovery: Advances and opportunities. Nat. Rev. Drug Discov..

[25] Swor K., Satyal P., Timsina S., Setzer W.N. (2022). Chemical Composition and Terpenoid Enantiomeric Distribution of the Essential oil of *Artemisia tridentata* Subsp. *tridentata* From Southwestern Idaho. Nat. Prod. Commun..

[26] Shafizadeh F., Bhadane N., Morris M.S., Kelsey R.G., Khanna S.N. (1971). Sesquiterpene lactones of big sagebrush. Phytochemistry.

[27] Geisssman T.A., Stewart T., Irwin M.A. (1967). Sesquiterpene lactones of *Artemisia* species-II. Phytochemistry.

[28] Dadabay C.Y., Spaulding P.B., Valenzuela E., Turner M., Eckert K.E., Julkunen-Tiitto R., Noblit N., Mansfield D.H. (2019). Polyphenols from the sagebrush *Artemisia tridentata* ssp. *tridentata* affect the redox state of cultured hepatocytes by direct and indirect mechanisms. Curr. Top. Phytochem..

[29] Martinez M.V., Munnoz-Zamora A., Joseph-Nathan P. (1988). Conformational analysis of achillin and leukodin. J. Nat. Prod..

[30] Zhanzhaxina A., Seiilgazy M., Jalmakhanbetova R.I., Ishmuratova M., Seilkhanov T.M., Oyama M., Sarmurzina Z., Tekebayeva Z.B., Suleimen E.M. (2020). flavonoids from Pulicaria vulgaris and their Antimicrobial Activity. Chem. Nat. Compd..

[31] Cuong D.T.D., Dat H.T., Duan N.T., Thuong P.D., Phat N.T., Tri M.D., Dang V., Hoa N.T., Tuyen P.N.K., Phụng N.K.P. (2019). Isolation and characterization of six flavonoids from the leaves of Sterculia foetida Linn. Vietnam. J. Chem..

[32] Batra P., Sharma A.K. (2013). Anti-cancer potential of flavonoids: Recent trends and future perspectives. Biotech.

[33] Yuan H., Lu X., Ma Q., Li D., Xu G., Piao G. (2016). Flavonoids from *Artemisia* sacrorum Ledeb. and their cytotoxic activities against human cancer cell lines. Exp. Ther. Med..

[34] Li L., Liu H., Tang C., Yao S., Ke C., Xu C., Ye Y. (2017). Cytotoxic sesquiterpene lactones from *Artemisia* anomala. Phytochem. Lett..

[35] Gunawardena K., Rivera S.B., Epstein W.W. (2002). The monoterpenes of *Artemisia tridentata* ssp. vaseyana, *Artemisia cana* ssp. viscidula and *Artemisia tridentata* ssp. spiciformis. Phytochemistry.

[36] Lone S.H., Bhat K.A., Khuroo M.A. (2015). Arglabin: From isolation to antitumor evaluation. Chem.-Biol. Interact..

[37] Chadwick M., Trewin H., Gawthrop F., Wagstaff C. (2013). Sesquiterpenoids lactones: Benefits to plants and people. Int. J. Mol. Sci..

[38] Kalidindi S., Jeong W.B., Schall A., Bandichhor R., Nosse B., Reiser O. (2007). Enantioselective synthesis of arglabin. Angew. Chem. Int. Ed..

[39] Burits M., Asres K., Bucar F. (2001). The antioxidant activity of the essential oils of *Artemisia* afra, *Artemisia* abyssinica and Juniperus procera. Phytother. Res..

[40] Benzie I.F., Strain S. (1996). The ferric reducing ability of plasma (FRAP) as a measure of “Antioxidant power”: The FRAP assay. Anal. Biochem..

[41] Brand-Williams W., Cuvelier M., Berset C. (1995). Use of a free radical method to evaluate antioxidant activity. LWT Food Sci. Technol..

[42] NCI-60 Screening Methodology|NCI-60 Human Tumor Cell Lines Screen|Discovery & Development Services|Developmental Therapeutics Program (DTP). https://dtp.cancer.gov/discovery_development/nci-60/methodology.htm.

