# Supplementary material for: Sesquiterpene Lactones and Flavonoid from the Leaves of Basin Big Sagebrush (Artemisia tridentata subsp. tridentata): Isolation, Characterization and Biological Activities
Source: Molecules. 2024 Feb 9;29(4):802. doi: 10.3390/molecules29040802 (PMC10892904; doi:10.3390/molecules29040802)
Supplement: Supplementary file 1 [file molecules-29-00802-s001.zip › molecules-2800749-supplementary/Supplementary Data/molecules-2800749-Supplementary Data S1-Figures S1-S10 and Tables S1-S14.pdf]

Sesquiterpene Lactones and Flavonoid from the Leaves of Basin Big Sagebrush (*Artemisia tridentata* subsp. *tridentata*): Isolation, Characterization and Biological Activities

Rosemary Anibogwu[1], Karl De Jesus[1], Samjhana Pradhan[1], Shanae Van Leuven[1], and Kavita Sharma[1,2*]

[1]Department of Chemistry, Idaho State University, Pocatello, ID 83209, USA; anibrose@isu.edu (R.A.); dejekarl@isu.edu (K.D.J.); samjhanapradhan@isu.edu (S.P.); shanaevanleuven@isu.edu (S.V.L.)

[2]Biomedical and Pharmaceutical Sciences, Kasiska Division of Health Sciences, College of Pharmacy, Pocatello, ID 83209, USA

*Corresponding author: kavitasharma@isu.edu

TLC Analysis

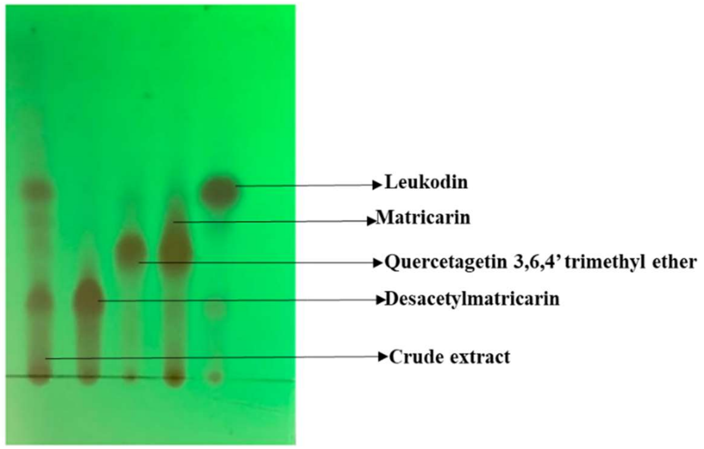

Figure S1. TLC of crude extract compared to pure Leucodin, Matricarin, Desacetylmatricarin and Quercetagetin 3,6,4′-trimethyl ether.

HPLC Analysis

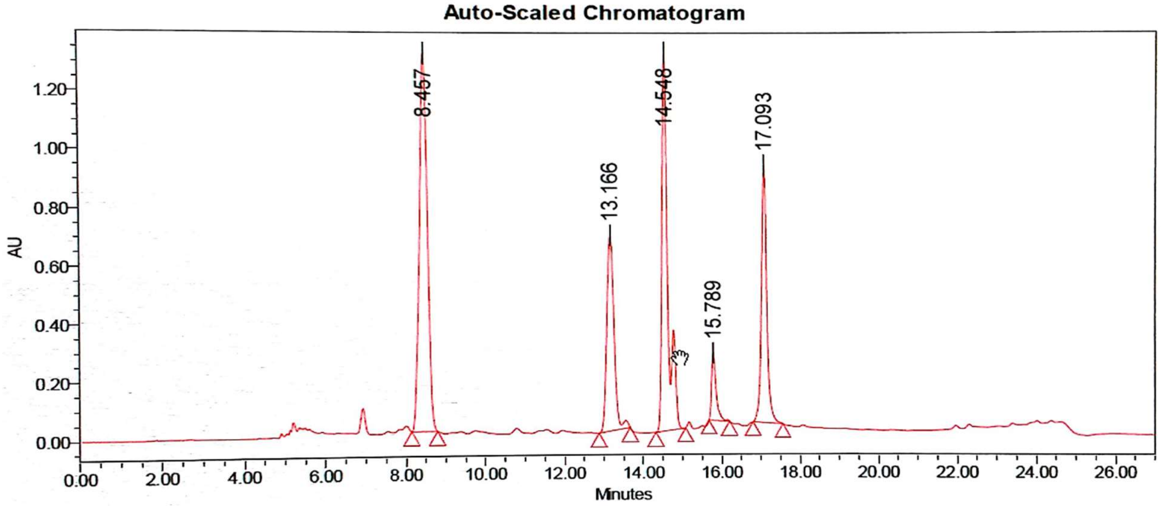

Figure S2. HPLC chromatogram of the bioactive compounds from *Artemisia tridentata* ssp. *tridentata*.

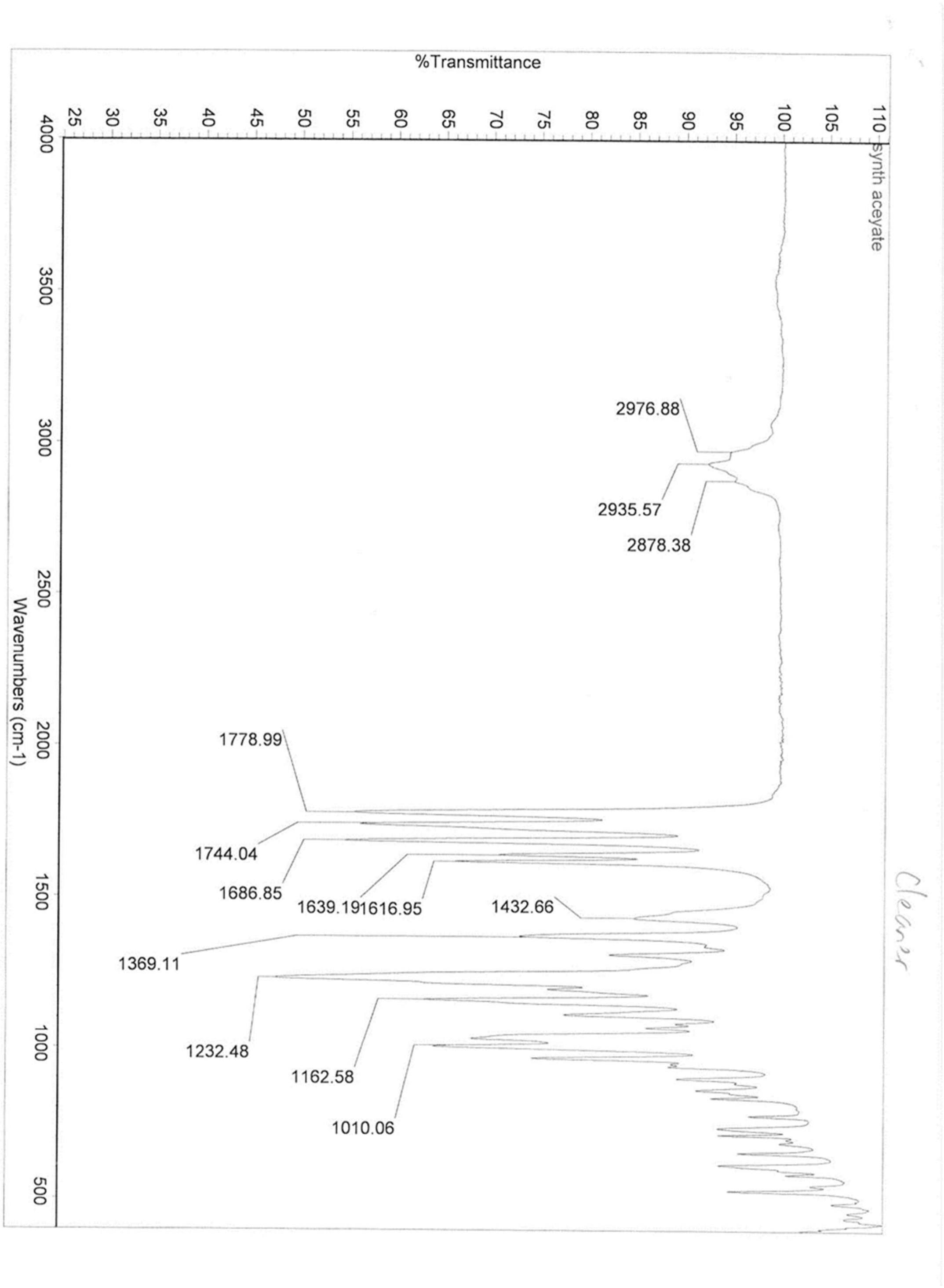

Figure S3. IR spectra of compound 2, matricarin.

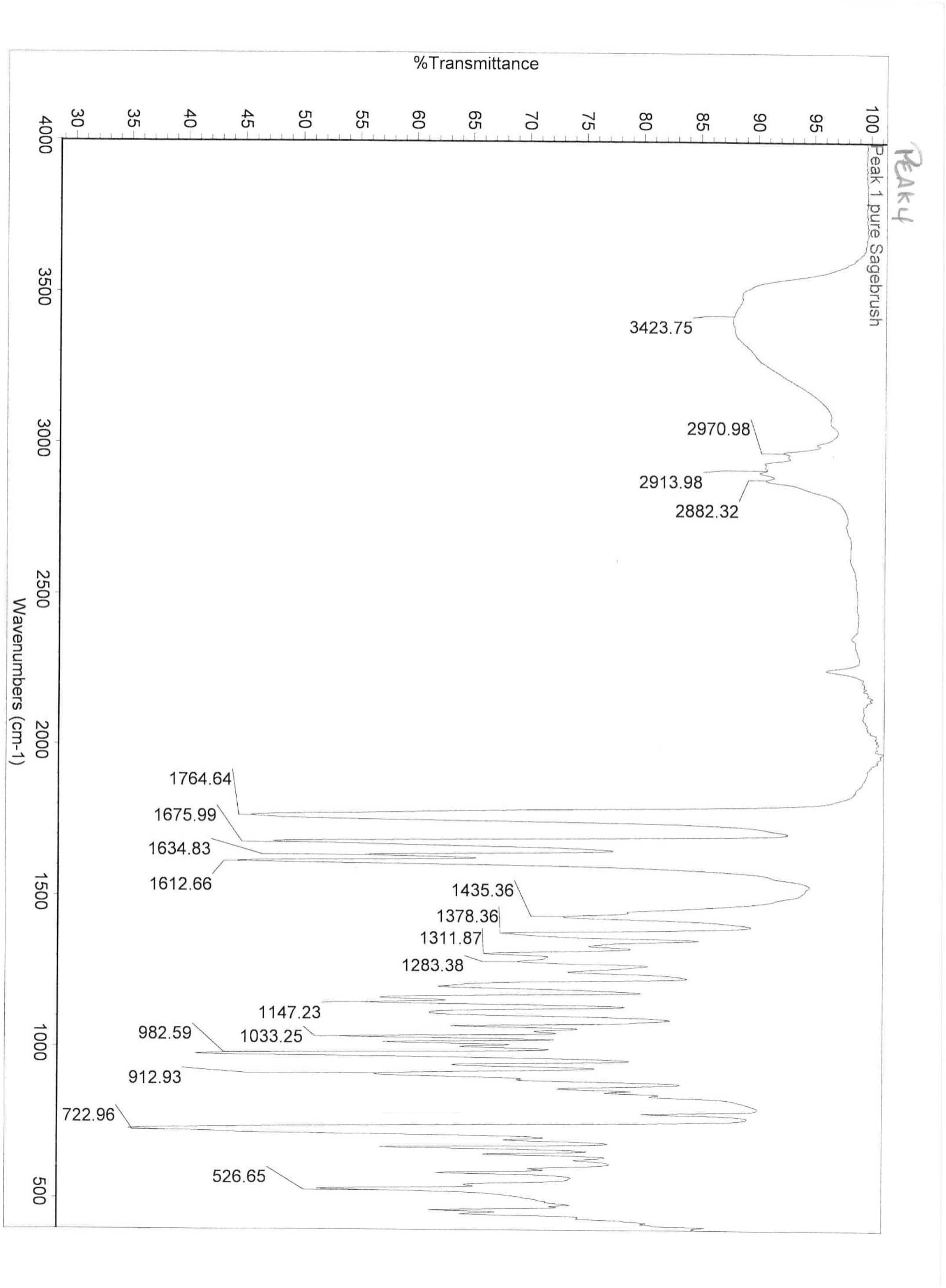

Figure S4. IR spectra of compound 4, desacetylmatricarin.

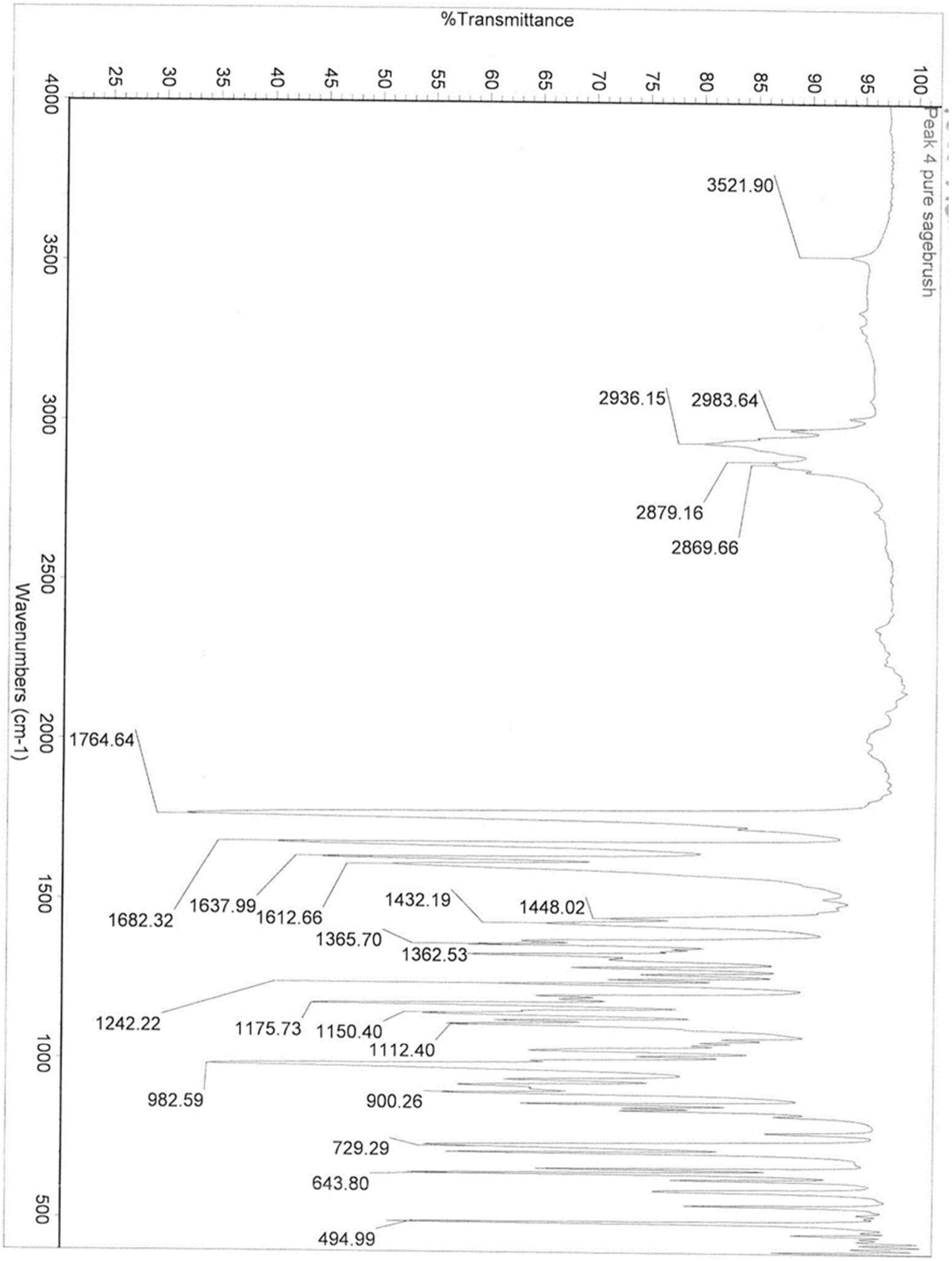

Figure S5. IR spectra of compound 1, leucodin.

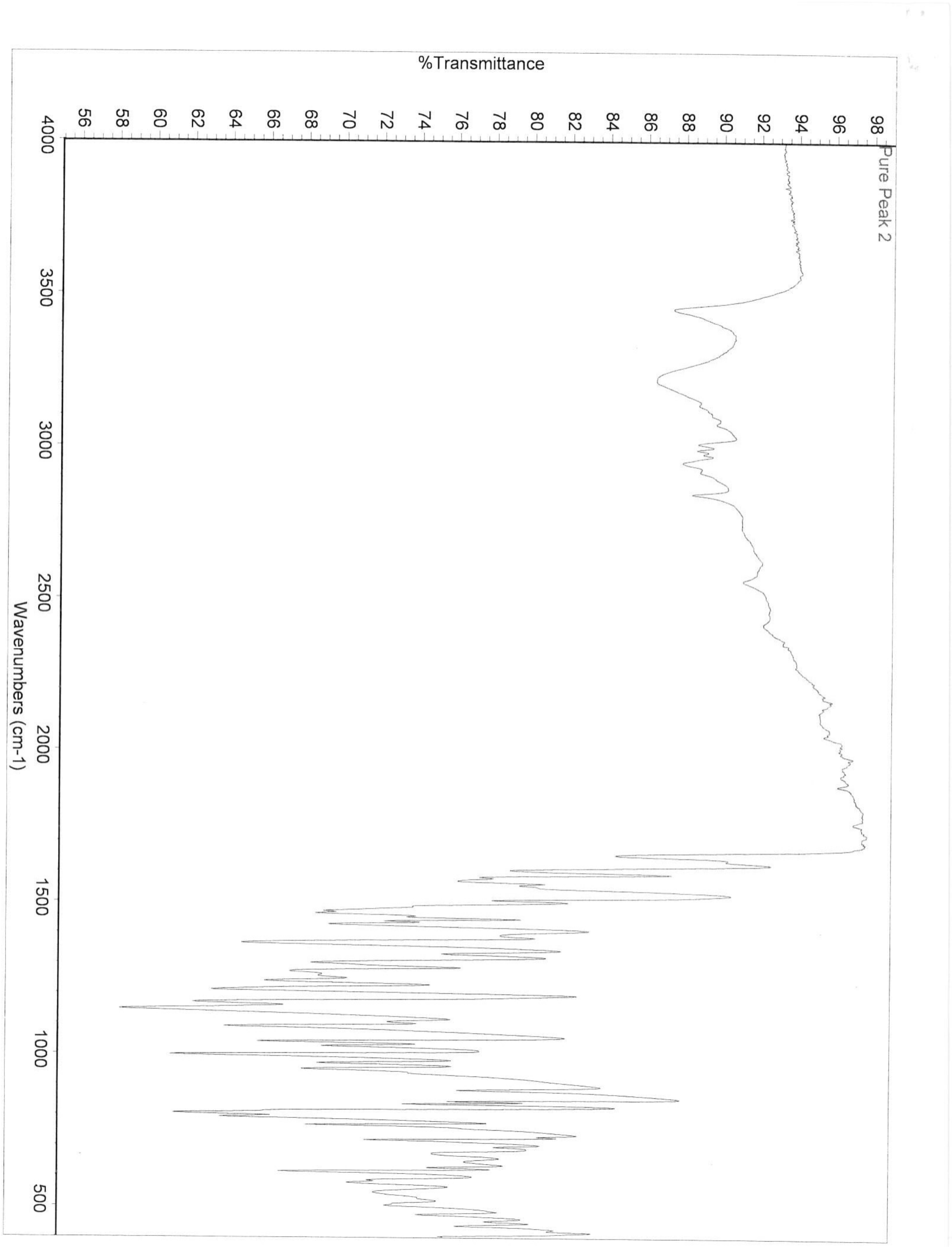

Figure S6. IR spectra (full scan range) of compound 3, Quercetagetin 3,6,4′-trimethyl ether.

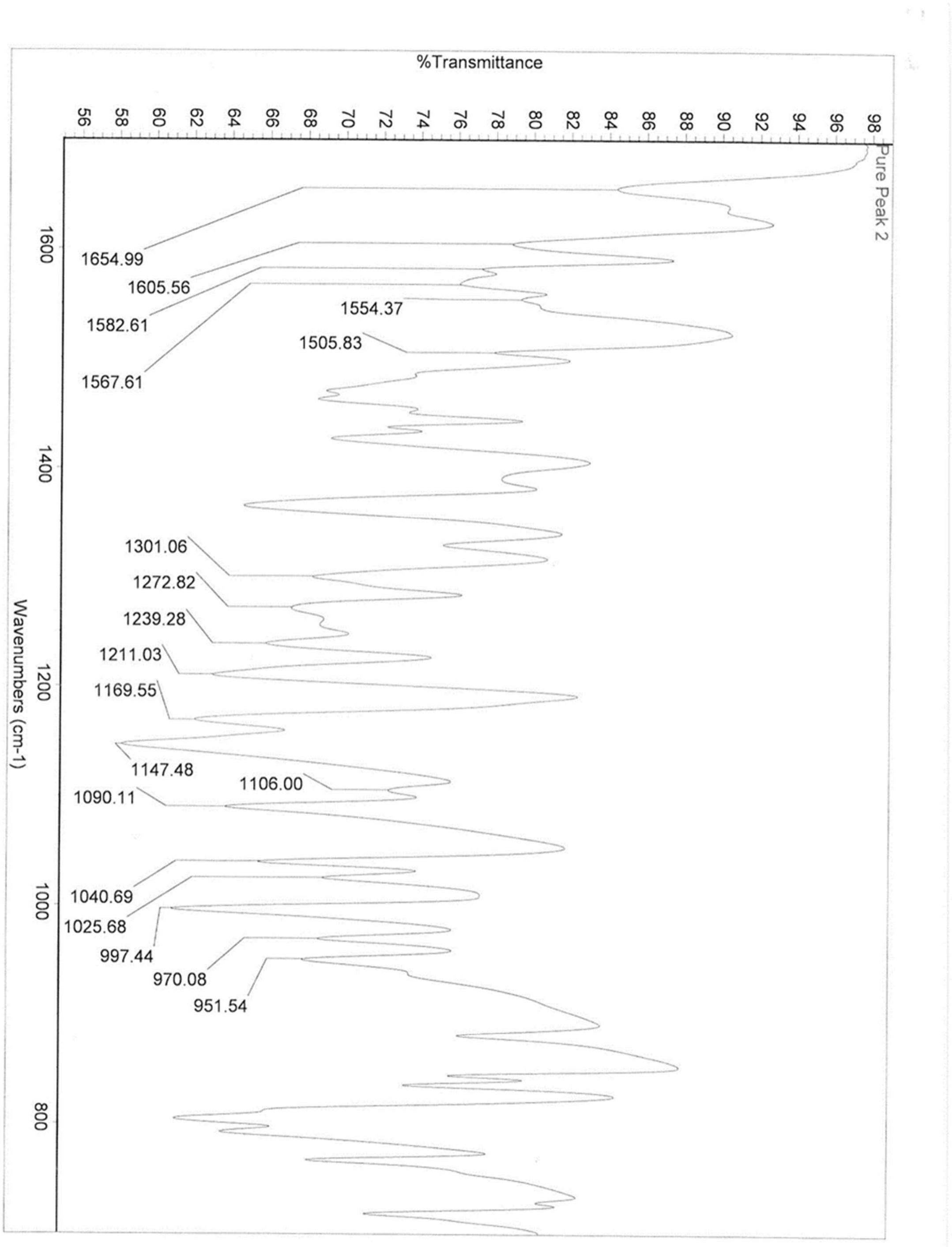

Figure S7. IR spectra (magnified section from wavenumber 800 to 1600 cm-1) of compound 3, Quercetagetin 3,6,4′-trimethyl ether.

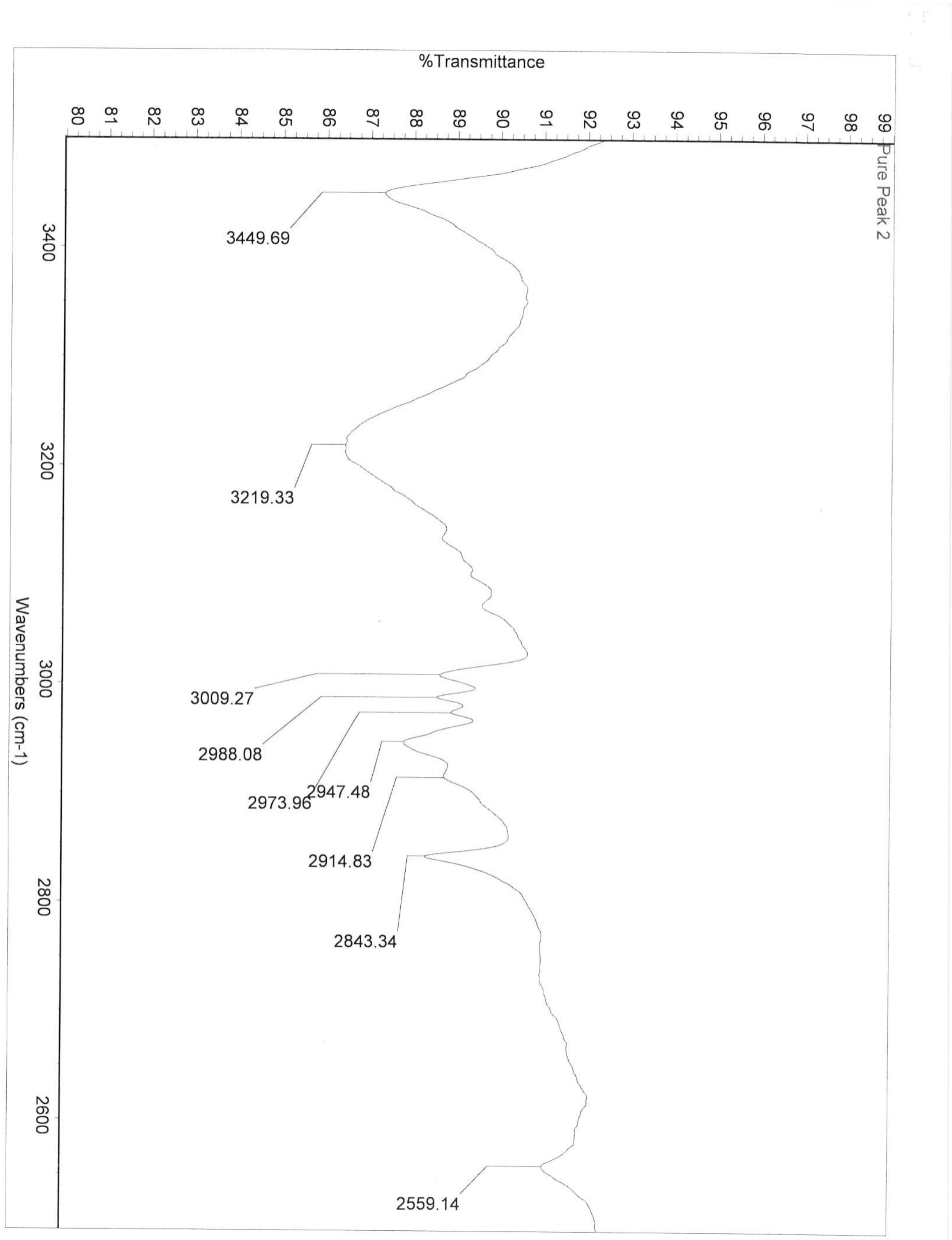

Figure S8. IR spectra (magnified section from wavenumber 2600 to 3400 cm^{-1}) of compound 3, Quercetagetin 3,6,4′-trimethyl ether.

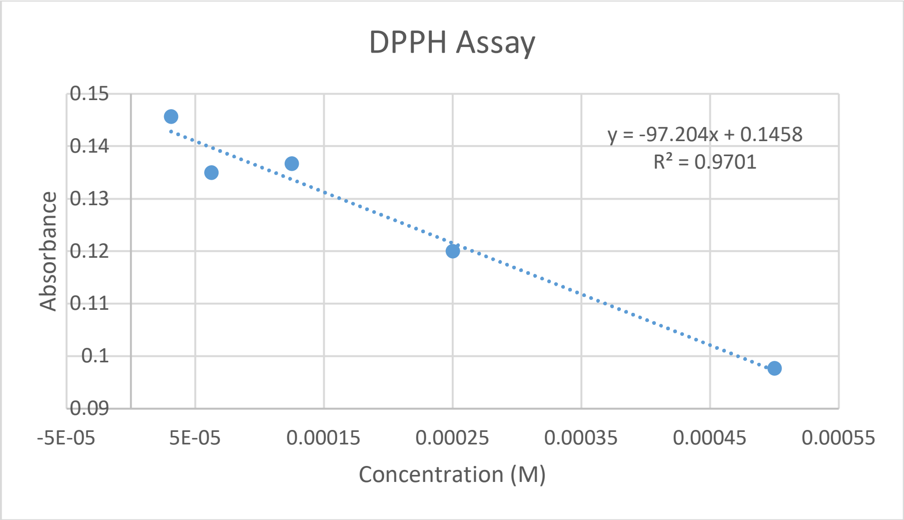

Figure S9. Reduction of free radicals using Trolox standards of increasing concentration and DPPH reagent.

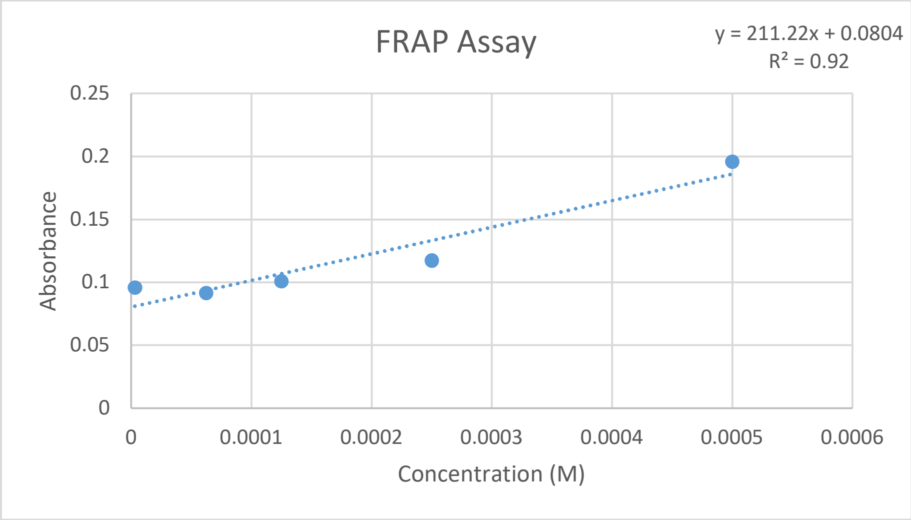

Figure S10. Reduction of free radicals using Trolox standards of increasing concentration and FRAP working reagent.

Table S1. Correlation of ^{1}H NMR data of compound 4, Desacetylmatricarin.

Protons	Chemical shift (ppm)	Integration	Multiplicity	Coupling constants (Hz)
H3	2.615	1H	dq	J3,3α = 11.8 Hz; J3,10 =6.9 Hz
H3α	2.219	1H	m	
H4	3.730	1H	td	J4,5α,3α = 10.3 Hz; J4,5β = 1.7 Hz
H5α	2.794	1H	dd	J5α,5β = 13.7 Hz; J5α,4 = 11.0 Hz
H5β	2.346	1H	dd	J5α,5β = 13.7 Hz; J5β,4 = 2.0 Hz
H8	6.085	1H	m	J8,12 = 1.4 Hz, J8, 11 = 1.0 Hz
H9α	3.554	1H	d	J9α,9β = 10.0 Hz
H9β	3.702	1H	t	J9β,9α/3α = 10.0 Hz
H10	1.335	3H	d	J10,3 = 6.9 Hz
H11	2.338	3H	bs	
H12	2.230	3H	bs	
H13	4.391	1H	bd	J13,4 = 4.8 Hz

Table S2. ^{13}C NMR and comparative BioRad data for compound **4**, Desacetylmatricarin.

^{13}C NMR	Chemical shift ppm ($CDCl_3$)	Chemical shift ppm (Acetone d6)	BioRad spectra base comparison ($CDCl_3$)
C2	177.38	178.48	177.5
C3	41.43	41.98	41.4
C3α	61.56	62.42	61.6
C4	69.60	70.14	69.6
C5	49.13	49.70	41.4
C6	145.84	146.94	145.3
C6α	131.05	134.18	133.1
C7	194.92	196.02	195
C8	135.71	136.13	135.7
C9	170.31	171.41	170
C9α	51.73	52.36	51.7
C9β	81.20	82.43	81.1
C10	15.61	16.25	15.5
C11	21.82	21.63	21.6
C12	20.04	20.16	19.8

Table S3. HETCOR correlations for compound **4**, Desacetylmatricarin.

HETCOR: $CDCl_3$	^{1}H	^{13}C
H3	2.615	41.98
H3α	2.219	62.42
H4	3.730	70.14
H5α	2.794	49.70
H5β	2.346	49.70

H8	6.085	136.13
H9α	3.554	52.36
H9β	3.702	82.43
H10	1.335	16.25
H11	2.338	21.63
H12	2.230	20.16
H13	4.391	

Table S4. COSY correlations for compound **4**, Desacetylmatricarin.

**COSY ($CDCl_3$)**	**Chemical shift (ppm)**	**Correlations**			
H3	2.615	2.219	1.335		
H3α	2.219	3.730	3.702		
H4	3.730	4.391	2.794	2.346	2.219
H5α	2.794	3.730	2.346		
H5β	2.346	4.391	3.730	2.794	
H8	6.085	3.554	2.338 (wk)	2.230	
H9α	3.554	6.085 (wk)	3.702	2.338	2.230
H9β	3.702	3.554	2.219		
H10	1.335	2.615	2.219 (wk)		
H11	2.338	6.085 (wk)	4.391		
H12	2.230	6.085			
H13	4.391	3.730	2.346		

wk = weak

Table S5. HMBC correlations for compound **4**, Desacetylmatricarin.

**HMBC ($CDCl_3$)**	**Chemical shift (ppm)**	**Correlations**					
H3	2.615	178.48	70.14	62.42	16.25		
H3α	2.219	82.43	70.14	52.36	49.70	41.98	16.25
H4	3.730	82.43	others missing				
H5α	2.794	146.94	134.18	70.14	62.42	21.63	
H5β	2.346	146.94	134.18	70.14	62.42	21.63	
H8	6.085	196.02	171.41	134.18	52.36	20.16	
H9α	3.554	171.41	134.18	82.43	62.42		
H9β	3.702	52.36	others missing				
H10	1.335	178.48	62.42	41.98			
H11	2.338	146.94	134.18	49.13			
H12	2.230	171.41	136.13	51.73			
H13	4.391	70.14	70.14				

wk = weak

Table S6. Correlation of ^{1}H NMR data of compound **1**, Leucodin.

Protons	Chemical shift (ppm)	Integration	Multiplicity	Coupling constants (Hz)
H3	2.246	1H	dq	J3,3α = 13.2 Hz; J3,10 =6.8 Hz
H3a	1.94	1H	m	
H4α	2.00	1H	m	
H4β	1.354	1H	m	
H5α	2.413	1H	m	
H5β	2.344	1H	m	
H8	6.164	1H	bs	
H9α	3.407	1H	bd	J9α,9β = 10.0 Hz
H9β	3.620	1H	t	J9β,9α/3α = 10.0 Hz
H10	1.266	3H	d	J10,3 = 6.8 Hz
H11	2.435	3H	bs	
H12	2.294	3H	bs	

Table S7. Comparison of ^{1}H NMR data of compound **1**, Leucodin with Achillin, literature values [32].

^{1}H NMR ($CDCl_3$)	Chemical shift (ppm)	Leucodin data	Achillin data
H3	2.246	2.275	2.702
H3α	1.94	1.964	2.491
H4α	2.00	2.040	1.861
H4β	1.354	1.380	1.44
H5α	2.413	2.445	2.382
H5β	2.344	2.370	2.337
H8	6.164	6.187	6.167
H9α	3.407	3.433	3.417
H9β	3.620	3.645	3.810
H10	1.266	1.296	1.136
H11	2.435	2.456	2.428
H12	2.294	2.318	2.278

Table S8. Comparison of ^{13}C NMR data of compound **1**, Leucodin with Achillin, literature values, [32].

^{13}C NMR ($CDCl_3$)	Chemical shift (ppm)	DEPT	Leucodin data	Achillin data
C2	177.74		177.5	178.3
C3	41.21	CH	41.1	39.3
C3α	56.41	CH	56.4	51.9
C4	26.04	CH_2	26.0	23.6
C5	37.64	CH_2	37.6	37.6
C6	152.37		152.1	152.1
C6α	131.91		131.9	131.8

C7	196.12		195.8	195.7
C8	135.60	CH	135.5	135.4
C9	170.18		169.9	170.1
C9α	52.62	CH	52.6	52.9
C9β	84.28	CH	84.2	83.5
C10	12.37	CH_3	12.3	9.9
C11	21.73	CH_3	21.6	21.5
C12	19.94	CH_3	19.8	19.7

Table S9. HETCOR correlations for compound **1**, Leucodin.

HETCOR ($CDCl_3$)	Chemical shift (ppm)	^{13}C
H3	2.246	41.21
H3α	1.94	56.41
H4α	2.00	26.04
H4β	1.354	26.04
H5α	2.413	37.64
H5β	2.344	37.64
H8	6.164	135.60
H9α	3.407	52.62
H9β	3.620	84.28
H10	1.266	12.37
H11	2.435	21.73
H12	2.294	19.94

Table S10. COSY correlations for compound **1**, Leucodin.

COSY ($CDCl_3$)	Chemical shift (ppm)	Correlations			
H3	2.246	1.94	1.266		
H3α	1.94	3.620	2.246	2.00	1.354
H4α	2.00	2.413	2.344	1.94	1.354
H4β	1.354	2.413	2.344	2.00	1.94
H5α	2.413	2.344	2.00	1.354	
H5β	2.344	2.413	2.00	1.354	
H8	6.164	3.407	2.294		
H9α	3.407	6.164 (wk)	3.620	2.435	2.294
H9β	3.620	3.407	1.94		
H10	1.266	2.246			
H11	2.435	4.391	3.407		
H12	2.294	6.164	3.407		

Table S11. HMBC correlations for compound **1**, Leucodin.

HMBC ($CDCl_3$)	Chemical shift (ppm)	Correlations				
H3	2.246	177.74	56.41	26.04	12.37	
H3α	1.94	84.28	52.62			12.37
H4α	2.00	152.37	84.28	56.41	41.21	37.64
H4β	1.354	152.37	84.28	56.41	41.21	37.64
H5α	2.413	152.37	131.91	37.64	26.04	
H5β	2.344	152.37	131.91	37.64	26.04	
H8	6.164	196.12	170.18	131.91	52.62	19.94
H9α	3.407	170.18	135.60	131.91	84.28	56.41
H9β	3.620	131.91	52.62	41.21(wk)	26.04	
H10	1.266	177.74	56.41	41.21		
H11	2.435	152.37	131.91	37.64		
H12	2.294	196.12	170.18	135.60	52.62	

Table S12. Correlation of ^{1}H NMR data for compound **3**, quercetagetin trimethyl ether.

^{1}H NMR (Acetone d6)	Chemical shift (ppm)	Integration	Multiplicity	Coupling constants
H8	6.60	1H	s	
H11, H12	3.88	3H	s	
H13	3.95	3H	s	
H2'	7.65	1H	d	J2',6' = 1.0 Hz
H5'	7.12	1H	d	J5',6' = 8.3 Hz
H6'	7.67	1H	dd	J6',5' = 8.3 Hz; J6',2' = 1.0 Hz

Table S13. ^{13}C NMR data for compound **3**, Quercetagetin trimethyl ether.

^{13}C NMR (Acetone d6)	Chemical shift (ppm)
C2	156.8
C3	139.3
C4	180.0
C5	153*
C6	132.0
C7	157.9
C8	94.6
C9	106.5
C10	153*
C11	60**
C12	60**
C13	56.4

C1'	124.3
C2'	112.2
C3'	147.4
C4'	151.0
C5'	115.9
C6'	122.0

Table S14. HMBC correlations for compound **3**, Quercetagetin trimethyl ether.

^{1}H NMR (Acetone d6)	Chemical shift (ppm)	Correlations				
H8	6.6	180.0 (wk)	157.7	153	132.0	106.5
H11	3.88	139.3				
H12	3.88	132.0				
H13	3.95	151.0				
H2'	7.65	156.8	151.0	147.4	124.3	122.0
H5'	7.12	151.0	147.4	122		
H6'	7.67	156.8	151.0	115.9	112 (wk)	

wk = weak
