# Supplementary material for: Sesquiterpene Lactones and Flavonoid from the Leaves of Basin Big Sagebrush (Artemisia tridentata subsp. tridentata): Isolation, Characterization and Biological Activities
Source: Molecules. 2024 Feb 9;29(4):802. doi: 10.3390/molecules29040802 (PMC10892904; doi:10.3390/molecules29040802)

Developmental Therapeutics Program
One Dose Mean Graph

NSC: D-824349 / 1	Conc: 1.00E-5 Molar	Test Date: Aug 17, 2020
Experiment ID: 2008OS33		Report Date: Nov 21, 2021

Panel/Cell Line	Growth Percent
**Leukemia**	
CCRF-CEM	91.62
HL-60(TB)	94.44
K-562	97.47
MOLT-4	95.07
RPMI-8226	103.27
SR	89.04
**Non-Small Cell Lung Cancer**	
A549/ATCC	96.16
EKVX	89.06
HOP-62	108.14
NCI-H226	96.78
NCI-H23	91.22
NCI-H322M	96.85
NCI-H460	101.77
NCI-H522	89.33
**Colon Cancer**	
COLO 205	110.81
HCC-2998	108.14
HCT-116	97.00
HCT-15	101.36
HT29	109.71
KM12	99.88
SW-620	105.67
**CNS Cancer**	
SF-268	98.83
SF-295	103.01
SF-539	98.82
SNB-19	95.29
SNB-75	83.55
U251	101.66
**Melanoma**	
LOX IMVI	89.20
MALME-3M	102.61
M14	92.52
MDA-MB-435	98.81
SK-MEL-2	103.63
SK-MEL-28	100.66
SK-MEL-5	97.78
UACC-257	93.02
UACC-62	89.15
**Ovarian Cancer**	
IGROV1	100.67
OVCAR-3	111.32
OVCAR-4	106.24
OVCAR-5	96.34
OVCAR-8	99.84
NCI/ADR-RES	93.33
SK-OV-3	112.09
**Renal Cancer**	
786-0	109.58
A498	72.19
ACHN	100.51
CAKI-1	105.32
RXF 393	102.38
SN12C	100.68
TK-10	100.75
UO-31	79.44
**Prostate Cancer**	
PC-3	98.42
DU-145	107.82
**Breast Cancer**	
MCF7	93.10
MDA-MB-231/ATCC	102.09
HS 578T	103.84
BT-549	99.49
T-47D	100.87
MDA-MB-468	79.83
Mean	98.26
Delta	26.07
Range	39.90

Mean Growth Percent - Growth Percent

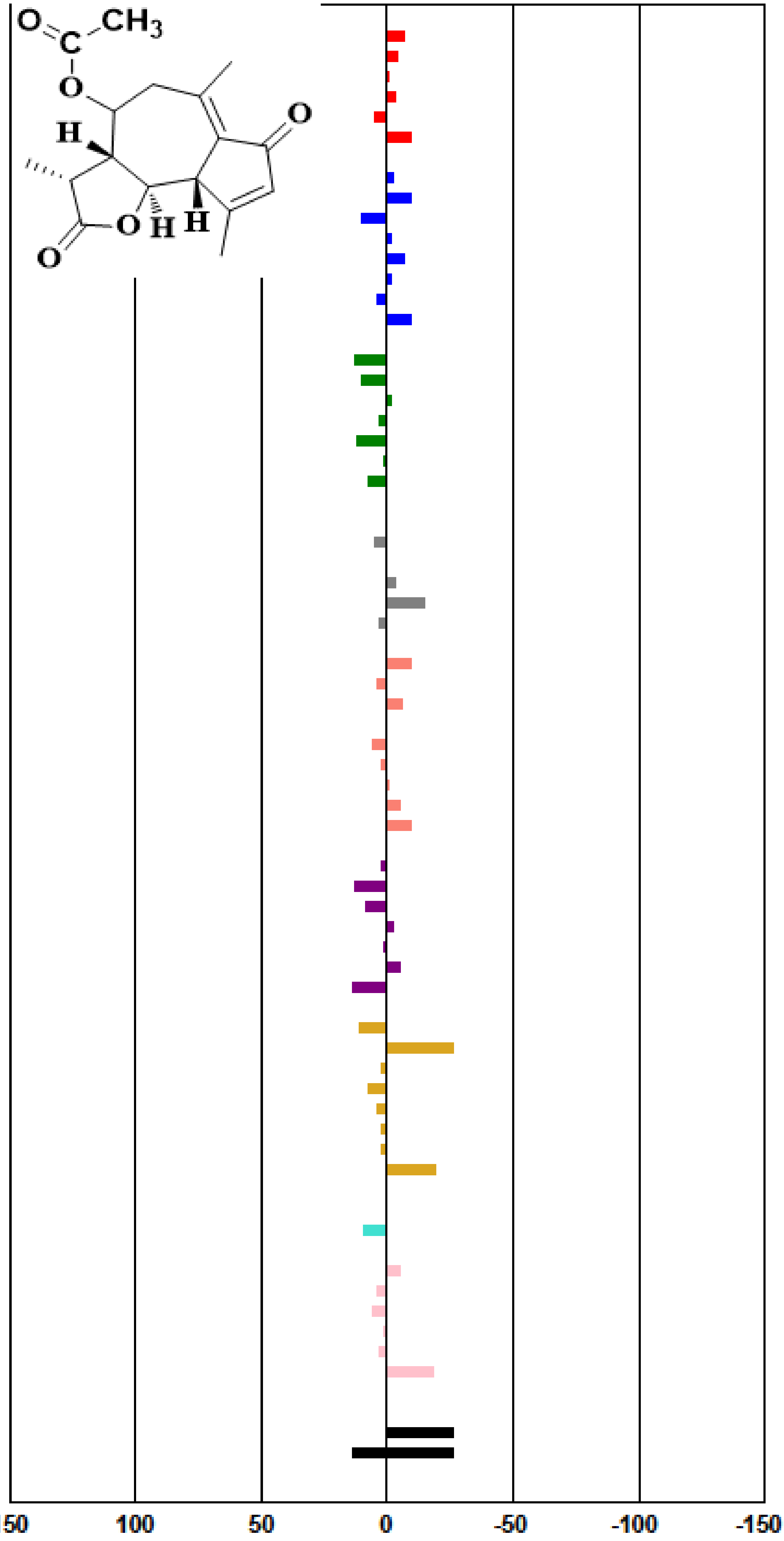

Developmental Therapeutics Program	NSC: D-824350 / 1	Conc: 1.00E-5 Molar	Test Date: Aug 17, 2020
One Dose Mean Graph	Experiment ID: 2008OS33		Report Date: Nov 21, 2021

Panel/Cell Line	Growth Percent
**Leukemia**	
CCRF-CEM	94.70
HL-60(TB)	95.08
K-562	97.23
MOLT-4	93.44
RPMI-8226	107.20
SR	88.15
**Non-Small Cell Lung Cancer**	
A549/ATCC	96.79
EKVX	96.80
HOP-62	102.36
NCI-H226	97.29
NCI-H23	97.24
NCI-H322M	96.27
NCI-H460	102.79
NCI-H522	90.21
**Colon Cancer**	
COLO 205	105.15
HCC-2998	98.67
HCT-116	98.73
HCT-15	100.59
HT29	112.78
KM12	99.00
SW-620	98.07
**CNS Cancer**	
SF-268	100.50
SF-295	102.25
SF-539	103.64
SNB-19	95.58
SNB-75	83.64
U251	99.26
**Melanoma**	
LOX IMVI	93.57
MALME-3M	100.29
M14	96.07
MDA-MB-435	97.66
SK-MEL-2	103.86
SK-MEL-28	99.15
SK-MEL-5	97.93
UACC-257	95.59
UACC-62	93.92
**Ovarian Cancer**	
IGROV1	98.29
OVCAR-3	107.75
OVCAR-4	100.32
OVCAR-5	101.43
OVCAR-8	96.57
NCI/ADR-RES	101.59
SK-OV-3	108.42
**Renal Cancer**	
786-0	99.00
A498	60.02
ACHN	103.79
CAKI-1	85.34
RXF 393	100.65
SN12C	95.48
TK-10	96.13
UO-31	84.96
**Prostate Cancer**	
PC-3	110.27
DU-145	108.43
**Breast Cancer**	
MCF7	98.06
MDA-MB-231/ATCC	99.65
HS 578T	93.34
BT-549	95.62
T-47D	100.16
MDA-MB-468	85.35
Mean	97.66
Delta	37.64
Range	52.76

Mean Growth Percent - Growth Percent

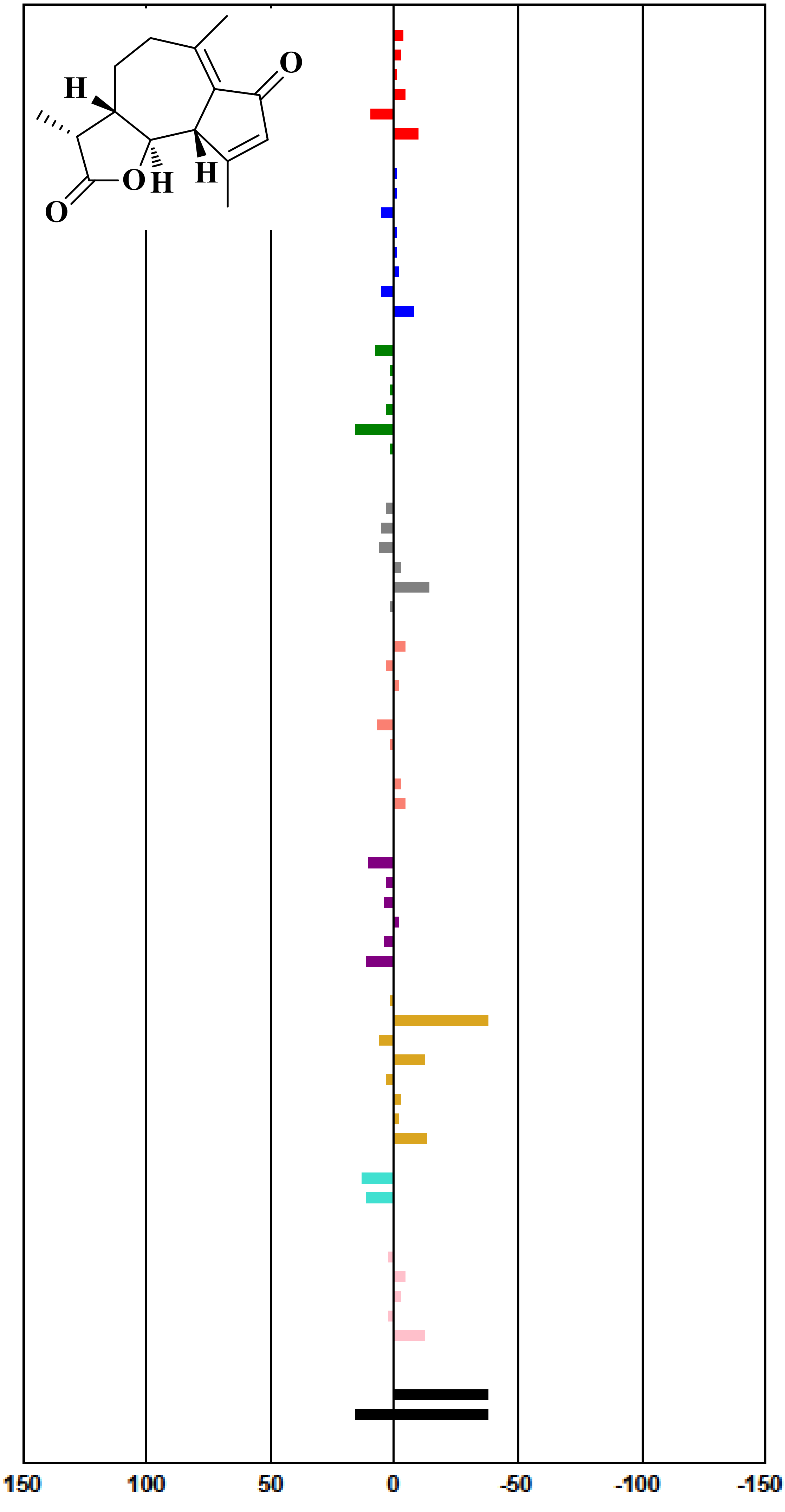

Developmental Therapeutics Program One Dose Mean Graph	NSC: D-824352 / 1	Conc: 1.00E-5 Molar	Test Date: Aug 17, 2020
	Experiment ID: 2008OS33		Report Date: Nov 21, 2021

Panel/Cell Line	Growth Percent
**Leukemia**	
CCRF-CEM	104.57
HL-60(TB)	98.53
K-562	95.32
MOLT-4	106.72
RPMI-8226	111.96
SR	89.38
**Non-Small Cell Lung Cancer**	
A549/ATCC	97.48
EKVX	98.98
HOP-62	102.42
HOP-92	116.19
NCI-H226	97.84
NCI-H23	97.38
NCI-H322M	103.93
NCI-H460	104.16
NCI-H522	85.54
**Colon Cancer**	
COLO 205	107.05
HCC-2998	107.46
HCT-116	101.50
HCT-15	100.13
HT29	102.86
KM12	103.32
SW-620	101.87
**CNS Cancer**	
SF-268	100.42
SF-295	103.79
SF-539	100.55
SNB-19	96.78
SNB-75	79.52
U251	95.36
**Melanoma**	
LOX IMVI	93.23
MALME-3M	94.24
M14	96.17
MDA-MB-435	99.06
SK-MEL-2	102.09
SK-MEL-28	110.07
SK-MEL-5	100.79
UACC-257	98.14
UACC-62	93.82
**Ovarian Cancer**	
IGROV1	97.06
OVCAR-3	111.43
OVCAR-4	94.05
OVCAR-5	105.39
OVCAR-8	98.59
NCI/ADR-RES	99.96
SK-OV-3	106.23
**Renal Cancer**	
786-0	105.00
A498	87.61
ACHN	102.39
CAKI-1	90.24
RXF 393	109.61
SN12C	92.07
TK-10	99.89
UO-31	80.94
**Prostate Cancer**	
PC-3	99.65
DU-145	107.36
**Breast Cancer**	
MCF7	95.92
MDA-MB-231/ATCC	87.91
HS 578T	105.18
BT-549	117.98
T-47D	95.77
MDA-MB-468	95.18
Mean	99.73
Delta	20.21
Range	38.46

Mean Growth Percent - Growth Percent

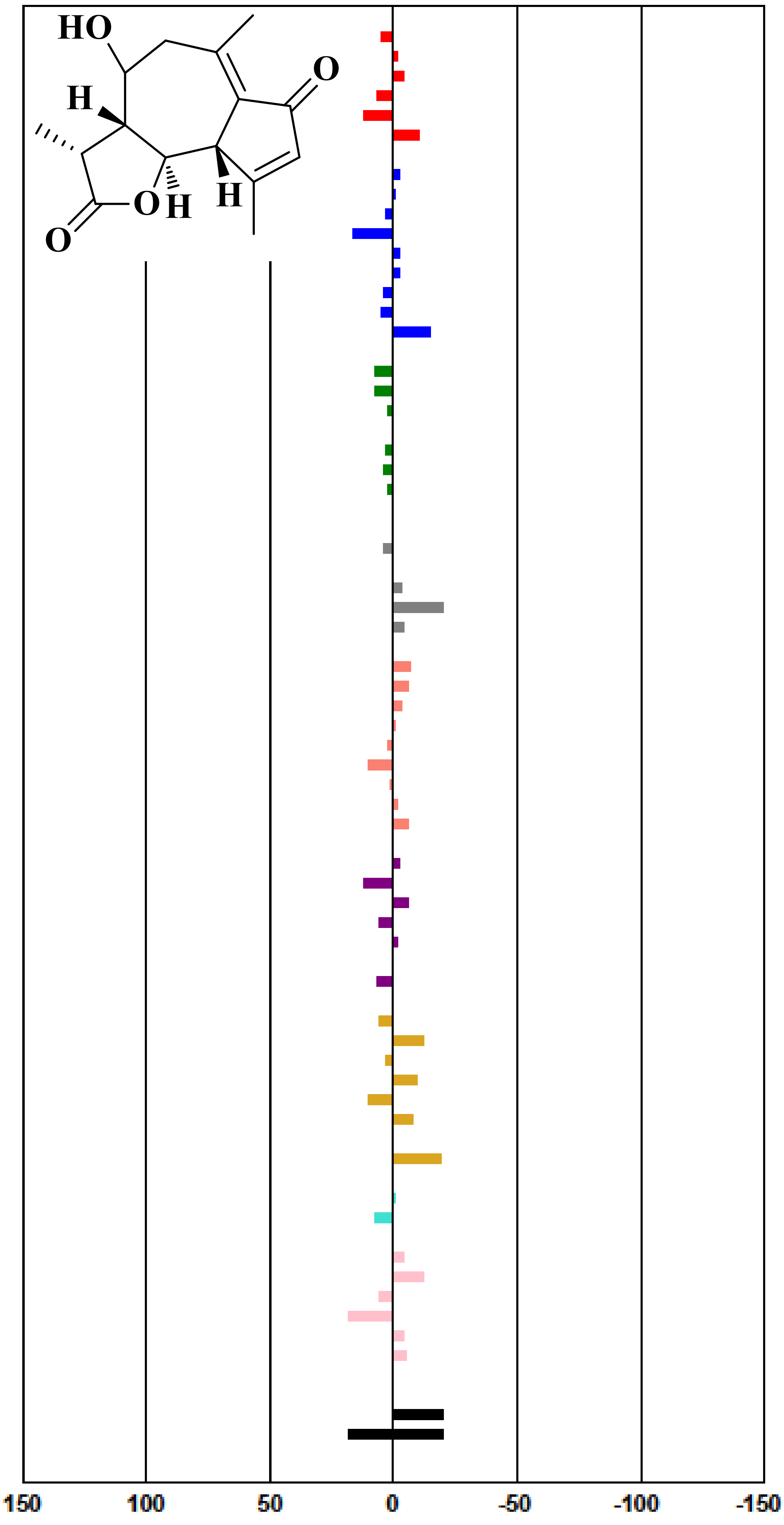

Developmental Therapeutics Program
One Dose Mean Graph

NSC: D-824351 / 1	Conc: 1.00E-5 Molar	Test Date: Aug 17, 2020
Experiment ID: 2008OS33		Report Date: Nov 21, 2021

Panel/Cell Line	Growth Percent
**Leukemia**	
CCRF-CEM	-5.15
HL-60(TB)	-28.59
K-562	6.80
MOLT-4	9.18
RPMI-8226	-19.29
SR	10.79
**Non-Small Cell Lung Cancer**	
A549/ATCC	17.68
EKVX	46.77
HOP-62	42.30
NCI-H226	16.78
NCI-H23	24.69
NCI-H322M	36.96
NCI-H460	4.55
NCI-H522	-11.89
**Colon Cancer**	
COLO 205	87.95
HCC-2998	20.11
HCT-116	3.39
HCT-15	3.60
HT29	46.78
KM12	8.81
SW-620	22.56
**CNS Cancer**	
SF-268	24.62
SF-295	-6.27
SF-539	-22.97
SNB-19	20.99
SNB-75	-8.14
U251	15.41
**Melanoma**	
LOX IMVI	12.87
MALME-3M	52.25
M14	-3.36
MDA-MB-435	-23.78
SK-MEL-2	46.88
SK-MEL-28	56.18
SK-MEL-5	-23.59
UACC-257	69.08
UACC-62	29.34
**Ovarian Cancer**	
IGROV1	14.09
OVCAR-3	-24.02
OVCAR-4	37.83
OVCAR-5	42.51
OVCAR-8	17.11
NCI/ADR-RES	16.63
SK-OV-3	46.48
**Renal Cancer**	
786-0	16.98
A498	-18.27
ACHN	21.61
CAKI-1	28.43
RXF 393	17.10
SN12C	26.61
TK-10	66.09
UO-31	44.83
**Prostate Cancer**	
PC-3	-22.25
DU-145	5.45
**Breast Cancer**	
MCF7	10.96
MDA-MB-231/ATCC	-8.18
HS 578T	24.65
BT-549	-14.63
T-47D	50.28
MDA-MB-468	7.99
Mean	16.82
Delta	45.41
Range	116.54

Mean Growth Percent - Growth Percent

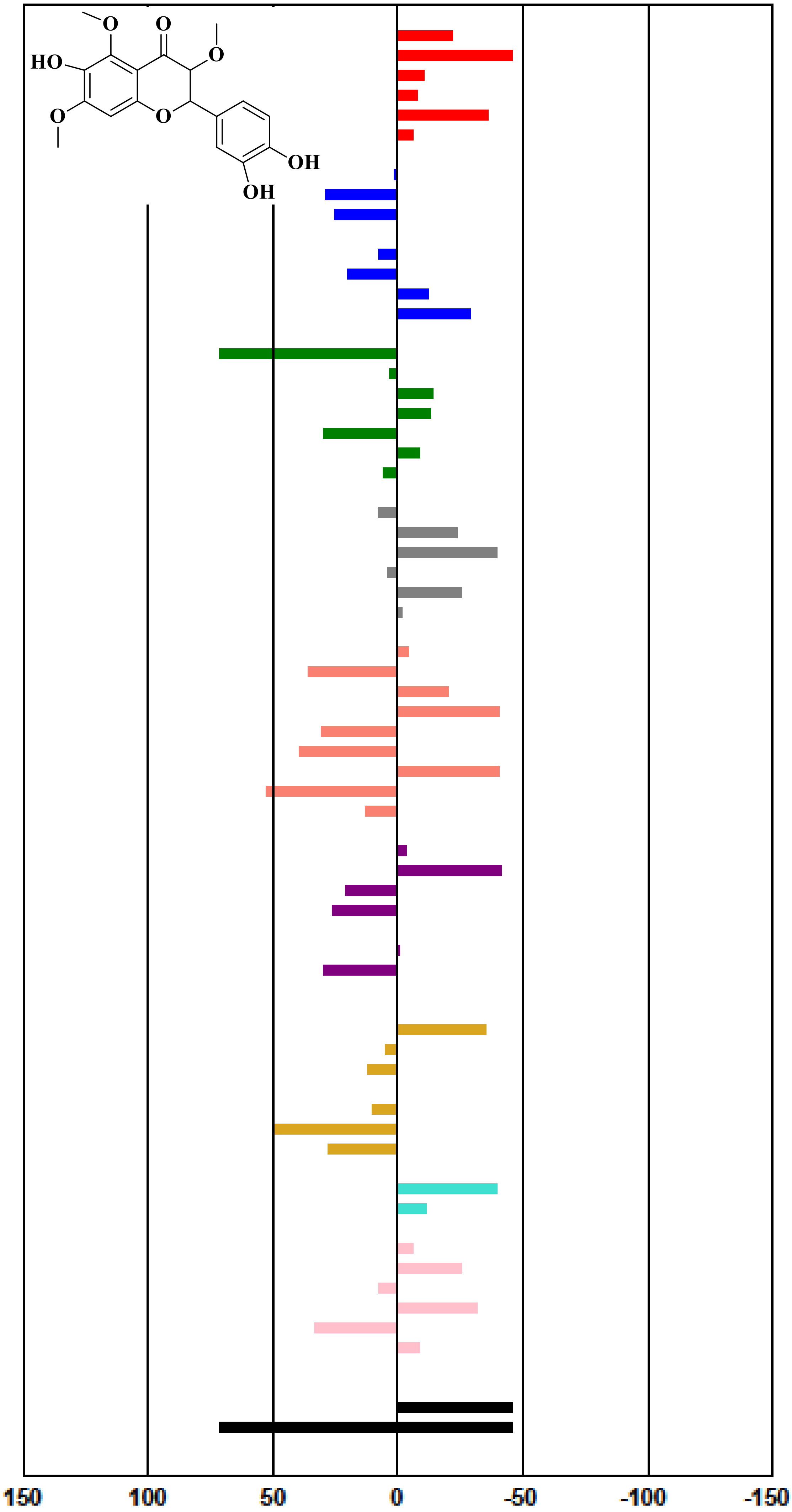

National Cancer Institute Developmental Therapeutics Program Dose Response Curves	NSC: D - 824351 / 1	SSPL: 1BZB	EXP. ID: 2011RS62
	Report Date: November 21, 2021	Test Date: November 16, 2020	

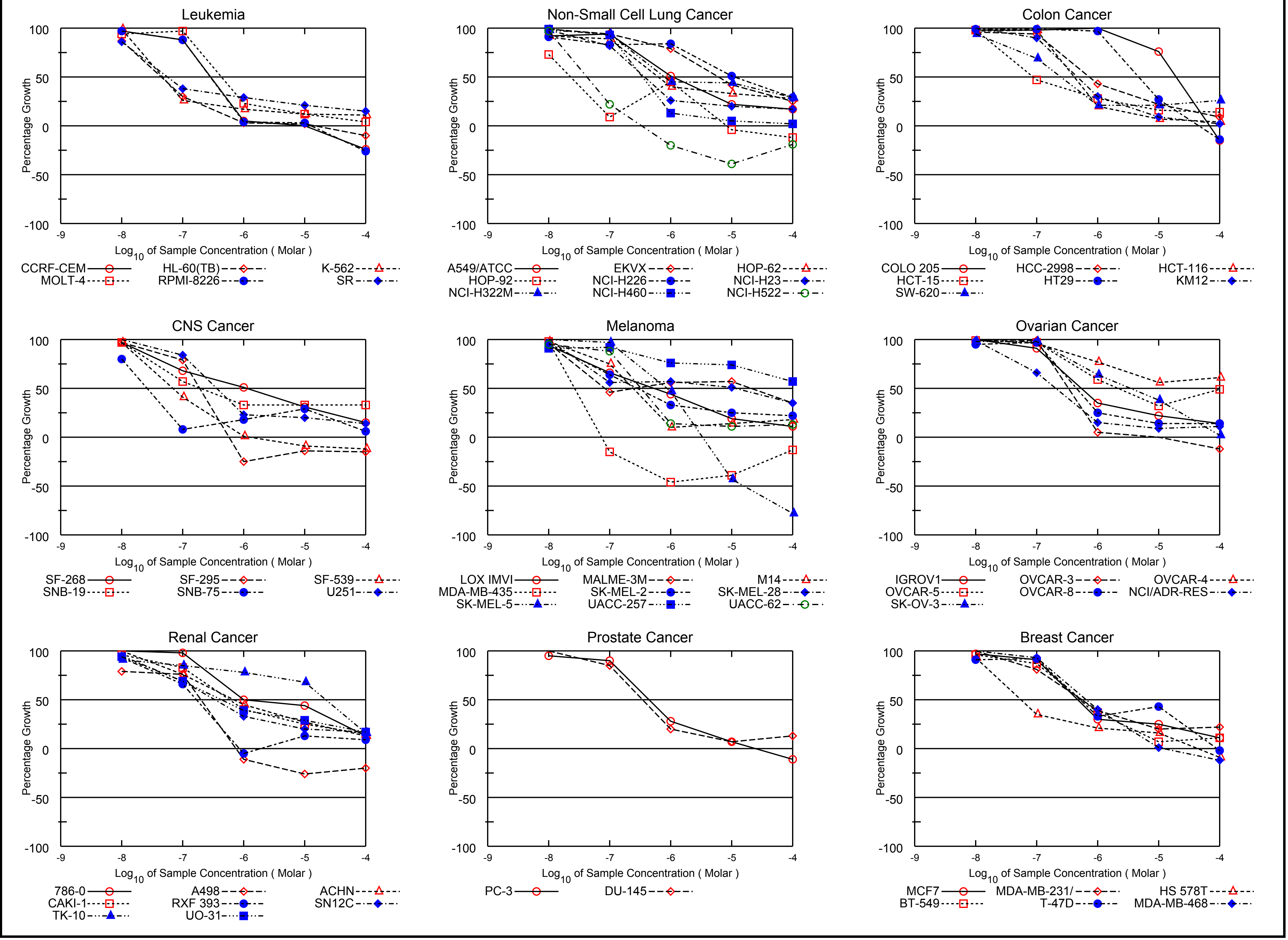

National Cancer Institute Developmental Therapeutics Program
In-Vitro Testing Results

NSC : D - 824351 / 1	Experiment ID : 2011RS62	Test Type : 08	Units : Molar
Report Date : November 21, 2021	Test Date : November 16, 2020	QNS :	MC :
COMI : 3	Stain Reagent : SRB Dual-Pass Related	SSPL : 1BZB	

			Log10 Concentration												
	Time		Mean Optical Densities					Percent Growth							
Panel/Cell Line	Zero	Ctrl	-8.0	-7.0	-6.0	-5.0	-4.0	-8.0	-7.0	-6.0	-5.0	-4.0	GI50	TGI	LC50
**Leukemia**															
CCRF-CEM	0.584	2.345	2.291	2.137	0.668	0.584	0.445	97	88	5	.	-24	2.87E-7	9.60E-6	> 1.00E-4
HL-60(TB)	0.530	2.748	2.465	1.195	0.603	0.584	0.475	87	30	3	2	-10	4.47E-8	1.54E-5	> 1.00E-4
K-562	0.174	1.923	1.900	0.628	0.467	0.382	0.363	99	26	17	12	11	4.67E-8	> 1.00E-4	> 1.00E-4
MOLT-4	0.484	2.661	2.532	2.606	0.981	0.755	0.568	94	97	23	12	4	4.32E-7	> 1.00E-4	> 1.00E-4
RPMI-8226	0.781	2.835	2.770	2.595	0.867	0.846	0.580	97	88	4	3	-26	2.85E-7	1.29E-5	> 1.00E-4
SR	0.192	0.715	0.644	0.393	0.343	0.300	0.270	86	38	29	21	15	5.72E-8	> 1.00E-4	> 1.00E-4
**Non-Small Cell Lung Cancer**															
A549/ATCC	0.589	2.721	2.559	2.584	1.683	1.059	0.954	92	94	51	22	17	1.11E-6	> 1.00E-4	> 1.00E-4
EKVX	0.858	2.474	2.463	2.374	2.137	1.530	1.267	99	94	79	42	25	5.97E-6	> 1.00E-4	> 1.00E-4
HOP-62	1.364	2.752	2.644	2.596	1.914	1.828	1.759	92	89	40	33	28	6.14E-7	> 1.00E-4	> 1.00E-4
HOP-92	1.325	1.612	1.534	1.350	1.453	1.275	1.164	73	9	45	-4	-12	2.27E-8	8.35E-6	> 1.00E-4
NCI-H226	1.196	2.563	2.444	2.335	2.341	1.895	1.592	91	83	84	51	29	1.12E-5	> 1.00E-4	> 1.00E-4
NCI-H23	0.554	2.141	2.115	1.857	0.962	0.871	0.817	98	82	26	20	17	3.71E-7	> 1.00E-4	> 1.00E-4
NCI-H322M	0.664	2.163	2.126	2.069	1.345	1.328	1.103	98	94	45	44	29	8.03E-7	> 1.00E-4	> 1.00E-4
NCI-H460	0.331	2.854	2.817	2.688	0.655	0.455	0.393	99	93	13	5	2	3.46E-7	> 1.00E-4	> 1.00E-4
NCI-H522	1.542	3.206	3.164	1.915	1.239	0.939	1.247	97	22	-20	-39	-19	4.29E-8	3.41E-7	> 1.00E-4
**Colon Cancer**															
COLO 205	0.571	2.165	2.132	2.126	2.188	1.777	0.486	98	98	101	76	-15	1.92E-5	6.85E-5	> 1.00E-4
HCC-2998	0.964	3.052	2.977	2.933	1.853	1.417	1.148	96	94	43	22	9	7.18E-7	> 1.00E-4	> 1.00E-4
HCT-116	0.252	2.561	2.485	2.520	0.711	0.417	0.349	97	98	20	7	4	4.13E-7	> 1.00E-4	> 1.00E-4
HCT-15	0.464	2.911	2.872	1.610	1.161	0.845	0.812	98	47	28	16	14	8.67E-8	> 1.00E-4	> 1.00E-4
HT29	0.361	1.894	1.882	1.872	1.848	0.770	0.312	99	99	97	27	-14	4.66E-6	4.58E-5	> 1.00E-4
KM12	0.510	2.711	2.783	2.500	1.168	0.709	0.563	103	90	30	9	2	4.65E-7	> 1.00E-4	> 1.00E-4
SW-620	0.339	2.449	2.320	1.797	0.778	0.793	0.891	94	69	21	21	26	2.48E-7	> 1.00E-4	> 1.00E-4
**CNS Cancer**															
SF-268	0.816	2.532	2.481	1.976	1.699	1.340	1.067	97	68	51	31	15	1.17E-6	> 1.00E-4	> 1.00E-4
SF-295	0.394	2.151	2.101	1.774	0.295	0.338	0.333	97	79	-25	-14	-15	1.88E-7	5.71E-7	> 1.00E-4
SF-539	0.788	2.476	2.432	1.473	0.812	0.716	0.693	97	41	1	-9	-12	6.82E-8	1.35E-6	> 1.00E-4
SNB-19	0.575	2.069	2.021	1.423	1.074	1.066	1.062	97	57	33	33	33	1.95E-7	> 1.00E-4	> 1.00E-4
SNB-75	1.328	2.160	1.991	1.394	1.479	1.570	1.376	80	8	18	29	6	2.59E-8	> 1.00E-4	> 1.00E-4
U251	0.452	2.165	2.161	1.898	0.839	0.788	0.687	100	84	23	20	14	3.60E-7	> 1.00E-4	> 1.00E-4
**Melanoma**															
LOX IMVI	0.550	2.944	2.795	2.121	1.602	0.998	0.809	94	66	44	19	11	5.26E-7	> 1.00E-4	> 1.00E-4
MALME-3M	0.651	1.746	1.708	1.150	1.260	1.272	1.036	96	46	56	57	35	.	> 1.00E-4	> 1.00E-4
M14	0.544	2.580	2.547	2.069	0.757	0.825	0.906	98	75	10	14	18	2.43E-7	> 1.00E-4	> 1.00E-4
MDA-MB-435	0.900	3.202	3.147	0.761	0.482	0.550	0.780	98	-15	-46	-39	-13	2.64E-8	7.30E-8	> 1.00E-4
SK-MEL-2	1.353	3.007	2.941	2.410	1.898	1.762	1.722	96	64	33	25	22	2.81E-7	> 1.00E-4	> 1.00E-4
SK-MEL-28	0.666	2.212	2.225	1.537	1.541	1.454	1.213	101	56	57	51	35	1.15E-5	> 1.00E-4	> 1.00E-4
SK-MEL-5	1.427	3.385	3.384	3.336	2.357	0.817	0.314	100	97	47	-43	-78	8.90E-7	3.36E-6	1.60E-5
UACC-257	1.619	3.039	2.918	2.919	2.701	2.666	2.425	91	92	76	74	57	> 1.00E-4	> 1.00E-4	> 1.00E-4
UACC-62	0.902	3.018	2.907	2.762	1.197	1.135	1.181	95	88	14	11	13	3.25E-7	> 1.00E-4	> 1.00E-4
**Ovarian Cancer**															
IGROV1	0.362	1.757	1.763	1.627	0.848	0.664	0.556	100	91	35	22	14	5.35E-7	> 1.00E-4	> 1.00E-4
OVCAR-3	0.570	1.904	1.993	1.884	0.639	0.575	0.500	107	99	5	.	-12	3.31E-7	1.07E-5	> 1.00E-4
OVCAR-4	1.066	1.999	2.033	1.960	1.788	1.584	1.632	104	96	77	56	61	> 1.00E-4	> 1.00E-4	> 1.00E-4
OVCAR-5	0.513	1.576	1.562	1.540	1.144	0.855	1.032	99	97	59	32	49	2.21E-6	> 1.00E-4	> 1.00E-4
OVCAR-8	0.513	2.353	2.261	2.296	0.978	0.775	0.765	95	97	25	14	14	4.51E-7	> 1.00E-4	> 1.00E-4
NCI/ADR-RES	0.825	2.756	2.734	2.091	1.118	0.999	1.045	99	66	15	9	11	2.03E-7	> 1.00E-4	> 1.00E-4
SK-OV-3	1.195	2.227	2.205	2.204	1.856	1.589	1.217	98	98	64	38	2	3.47E-6	> 1.00E-4	> 1.00E-4
**Renal Cancer**															
786-0	0.614	2.326	2.320	2.293	1.467	1.372	0.831	100	98	50	44	13	9.92E-7	> 1.00E-4	> 1.00E-4
A498	1.885	2.646	2.486	2.467	1.669	1.389	1.507	79	76	-11	-26	-20	2.00E-7	7.41E-7	> 1.00E-4
ACHN	0.271	1.384	1.383	1.113	0.768	0.572	0.413	100	76	45	27	13	6.70E-7	> 1.00E-4	> 1.00E-4
CAKI-1	0.638	2.303	2.240	2.015	1.296	1.054	0.894	96	83	40	25	15	5.72E-7	> 1.00E-4	> 1.00E-4
RXF 393	1.079	1.983	1.924	1.673	1.021	1.198	1.161	93	66	-5	13	9	1.66E-7	.	> 1.00E-4
SN12C	0.526	1.928	1.857	1.492	0.993	0.801	0.766	95	69	33	20	17	3.39E-7	> 1.00E-4	> 1.00E-4
TK-10	1.310	2.101	2.026	1.985	1.930	1.848	1.434	91	85	78	68	16	2.20E-5	> 1.00E-4	> 1.00E-4
UO-31	0.478	2.055	1.966	1.561	1.099	0.938	0.749	94	69	39	29	17	4.34E-7	> 1.00E-4	> 1.00E-4
**Prostate Cancer**															
PC-3	0.572	1.900	1.838	1.772	0.940	0.669	0.512	95	90	28	7	-11	4.41E-7	2.56E-5	> 1.00E-4
DU-145	0.480	2.143	2.159	1.893	0.807	0.593	0.703	101	85	20	7	13	3.43E-7	> 1.00E-4	> 1.00E-4
**Breast Cancer**															
MCF7	0.917	3.047	2.973	2.860	1.558	1.456	1.159	97	91	30	25	11	4.72E-7	> 1.00E-4	> 1.00E-4
MDA-MB-231/ATCC	0.589	1.477	1.502	1.312	0.940	0.763	0.784	103	81	39	20	22	5.61E-7	> 1.00E-4	> 1.00E-4
HS 578T	1.473	2.288	2.235	1.761	1.646	1.604	1.344	93	35	21	16	-9	5.59E-8	4.43E-5	> 1.00E-4
BT-549	1.041	2.337	2.285	2.165	1.523	1.131	1.182	96	87	37	7	11	5.52E-7	> 1.00E-4	> 1.00E-4
T-47D	0.850	1.856	1.764	1.778	1.180	1.282	0.837	91	92	33	43	-2	5.13E-7	9.24E-5	> 1.00E-4
MDA-MB-468	1.631	2.919	2.930	2.834	2.145	1.650	1.431	101	93	40	1	-12	6.47E-7	1.28E-5	> 1.00E-4

National Cancer Institute Developmental Therapeutics Program<br>Mean Graphs	NSC : D - 824351/1	Units :Molar	SSPL :1BZB	EXP. ID :2011RS62
	Report Date :November 21, 2021		Test Date :November 16, 2020	

Panel/Cell Line	Log_{10}GI50	Log_{10}TGI	Log_{10}LC50
**Leukemia**			
CCRF-CEM	-6.54	-5.02	> -4.00
HL-60(TB)	-7.35	-4.81	> -4.00
K-562	-7.33	> -4.00	> -4.00
MOLT-4	-6.36	> -4.00	> -4.00
RPMI-8226	-6.54	-4.89	> -4.00
SR	-7.24	> -4.00	> -4.00
**Non-Small Cell Lung Cancer**			
A549/ATCC	-5.96	> -4.00	> -4.00
EKVX	-5.22	> -4.00	> -4.00
HOP-62	-6.21	> -4.00	> -4.00
HOP-92	-7.64	-5.08	> -4.00
NCI-H226	-4.95	> -4.00	> -4.00
NCI-H23	-6.43	> -4.00	> -4.00
NCI-H322M	-6.10	> -4.00	> -4.00
NCI-H460	-6.46	> -4.00	> -4.00
NCI-H522	-7.37	-6.47	> -4.00
**Colon Cancer**			
COLO 205	-4.72	-4.16	> -4.00
HCC-2998	-6.14	> -4.00	> -4.00
HCT-116	-6.38	> -4.00	> -4.00
HCT-15	-7.06	> -4.00	> -4.00
HT29	-5.33	-4.34	> -4.00
KM12	-6.33	> -4.00	> -4.00
SW-620	-6.61	> -4.00	> -4.00
**CNS Cancer**			
SF-268	-5.93	> -4.00	> -4.00
SF-295	-6.73	-6.24	> -4.00
SF-539	-7.17	-5.87	> -4.00
SNB-19	-6.71	> -4.00	> -4.00
SNB-75	-7.59	> -4.00	> -4.00
U251	-6.44	> -4.00	> -4.00
**Melanoma**			
LOX IMVI	-6.28	> -4.00	> -4.00
MALME-3M		> -4.00	> -4.00
M14	-6.61	> -4.00	> -4.00
MDA-MB-435	-7.58	-7.14	> -4.00
SK-MEL-2	-6.55	> -4.00	> -4.00
SK-MEL-28	-4.94	> -4.00	> -4.00
SK-MEL-5	-6.05	-5.47	-4.80
UACC-257	> -4.00	> -4.00	> -4.00
UACC-62	-6.49	> -4.00	> -4.00
**Ovarian Cancer**			
IGROV1	-6.27	> -4.00	> -4.00
OVCAR-3	-6.48	-4.97	> -4.00
OVCAR-4	> -4.00	> -4.00	> -4.00
OVCAR-5	-5.66	> -4.00	> -4.00
OVCAR-8	-6.35	> -4.00	> -4.00
NCI/ADR-RES	-6.69	> -4.00	> -4.00
SK-OV-3	-5.46	> -4.00	> -4.00
**Renal Cancer**			
786-0	-6.00	> -4.00	> -4.00
A498	-6.70	-6.13	> -4.00
ACHN	-6.17	> -4.00	> -4.00
CAKI-1	-6.24	> -4.00	> -4.00
RXF 393	-6.78		> -4.00
SN12C	-6.47	> -4.00	> -4.00
TK-10	-4.66	> -4.00	> -4.00
UO-31	-6.36	> -4.00	> -4.00
**Prostate Cancer**			
PC-3	-6.36	-4.59	> -4.00
DU-145	-6.46	> -4.00	> -4.00
**Breast Cancer**			
MCF7	-6.33	> -4.00	> -4.00
MDA-MB-231/ATCC	-6.25	> -4.00	> -4.00
HS 578T	-7.25	-4.35	> -4.00
BT-549	-6.26	> -4.00	> -4.00
T-47D	-6.29	-4.03	> -4.00
MDA-MB-468	-6.19	-4.89	> -4.00
_MID	-6.29	-4.35	-4.01
Delta	1.35	2.79	0.79
Range	3.64	3.14	0.8

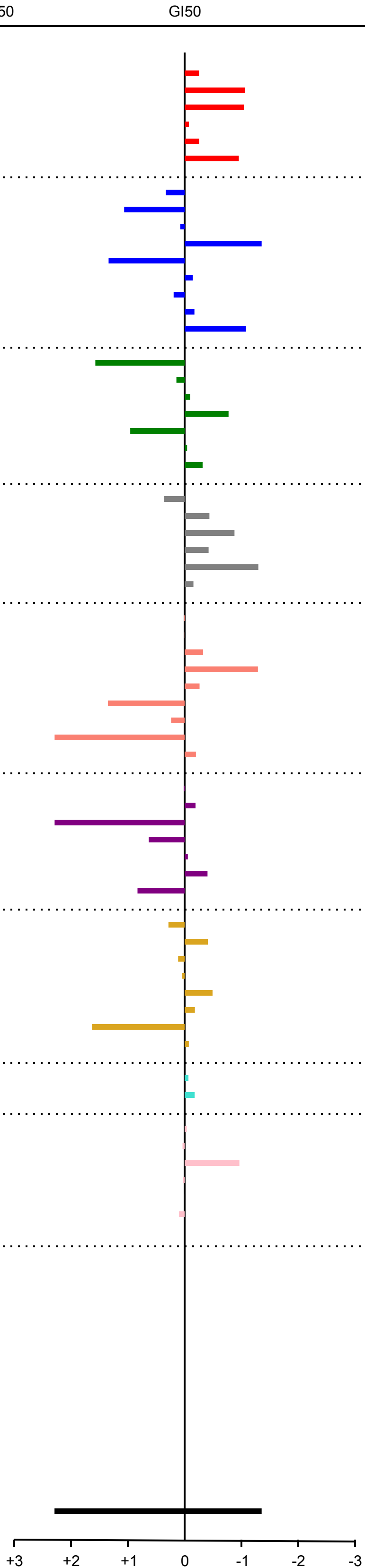

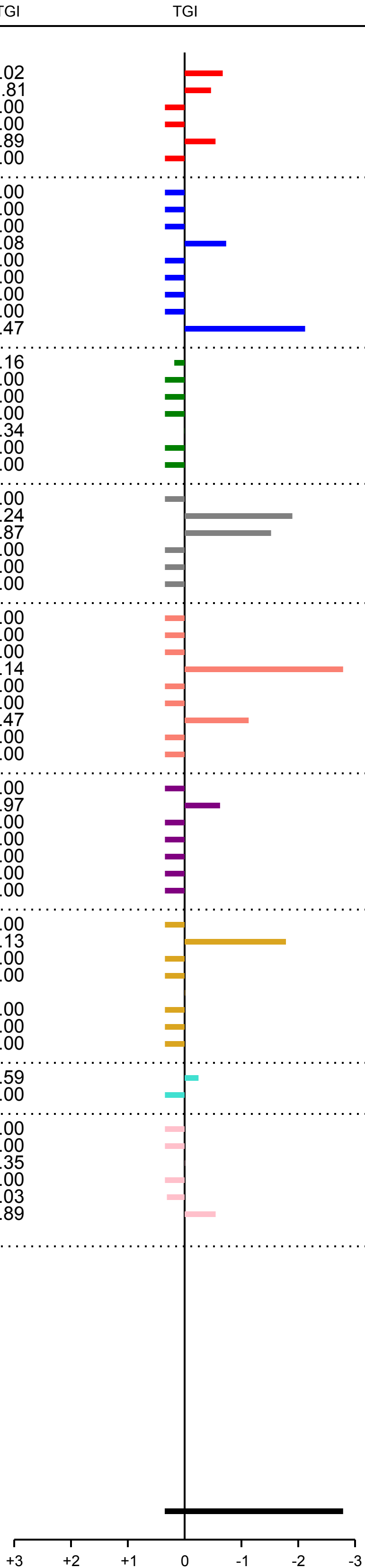

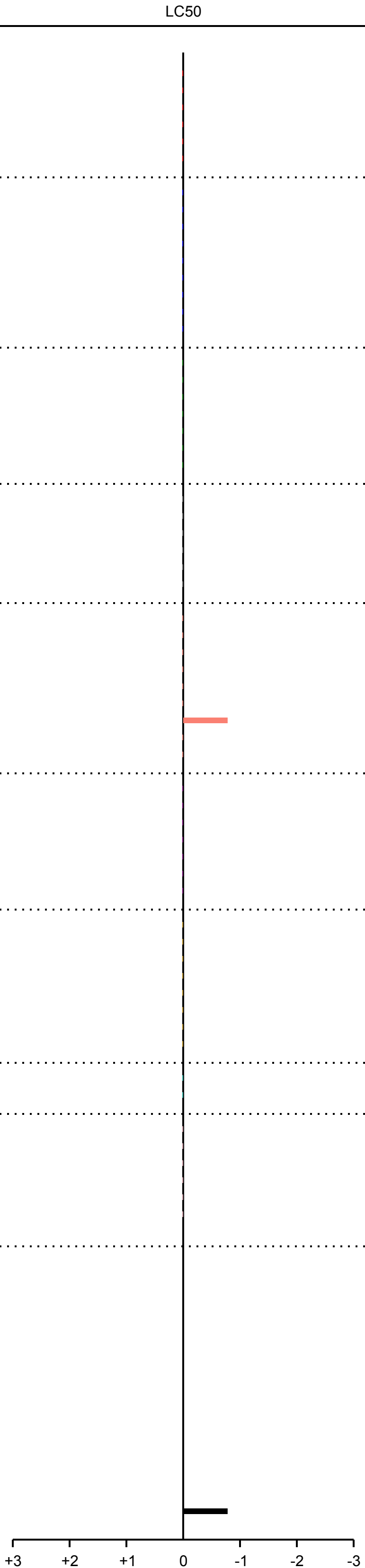

National Cancer Institute Developmental Therapeutics Program<br>Dose Response Curves	NSC : D - 824351/1	SSPL:1BZB	EXP. ID:2011RS62
	Report Date:November 21, 2021	Test Date:November 16, 2020	

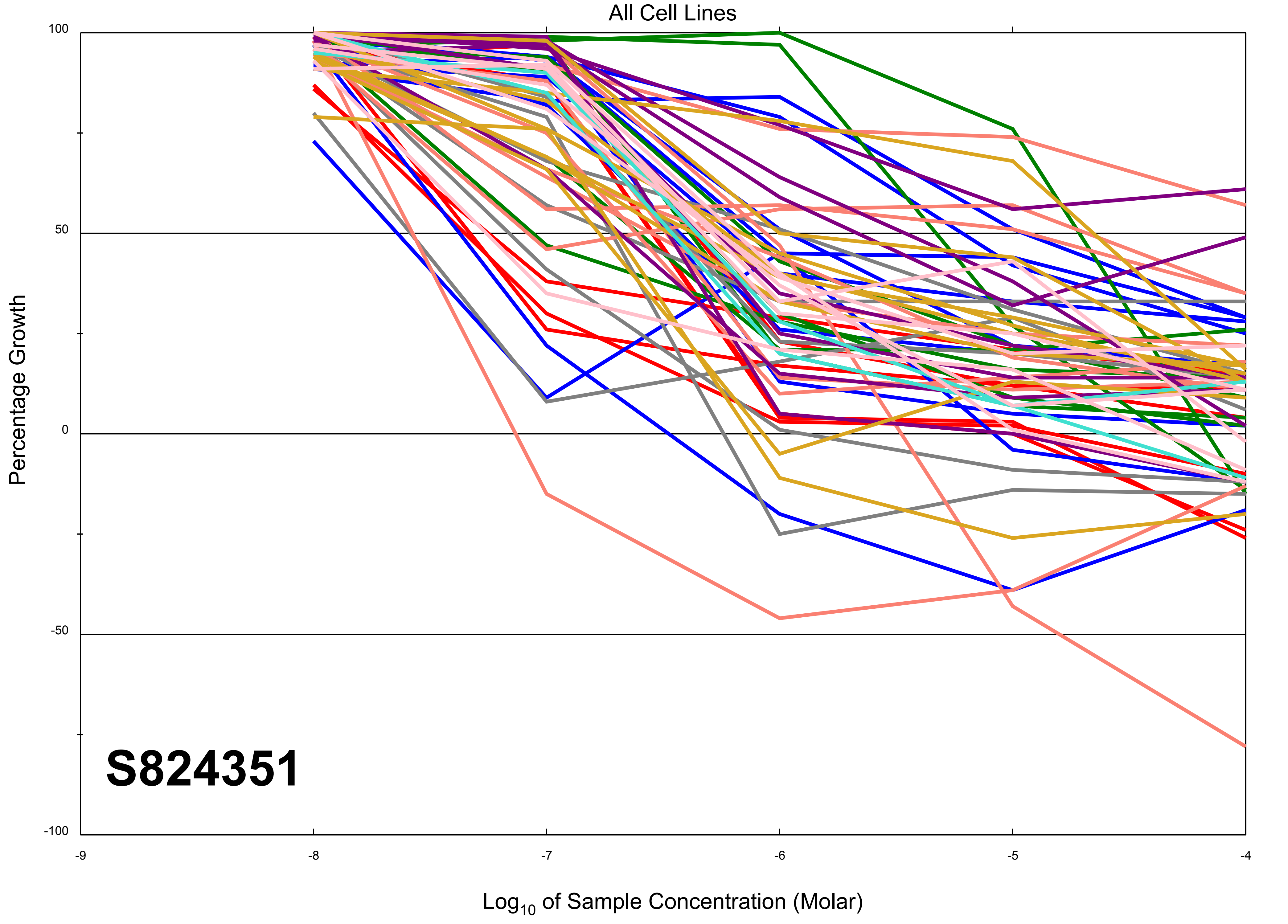

Supplement: Supplementary file 1 [file molecules-29-00802-s001.zip › molecules-2800749-supplementary/Supplementary Data/Supplementary Data-3-NCI data.pdf]
