# Supplementary material for: Sesquiterpene Lactones and Flavonoid from the Leaves of Basin Big Sagebrush (Artemisia tridentata subsp. tridentata): Isolation, Characterization and Biological Activities
Source: Molecules. 2024 Feb 9;29(4):802. doi: 10.3390/molecules29040802 (PMC10892904; doi:10.3390/molecules29040802)
Supplement: Supplementary file 1 [file molecules-29-00802-s001.zip › molecules-2800749-supplementary/Supplementary Data/Supplementary Data-4a-NMR-Scanned Files/Desacetylmatricarin NMR.pdf]

6.4 Hydroxyachillin NMR Spectra

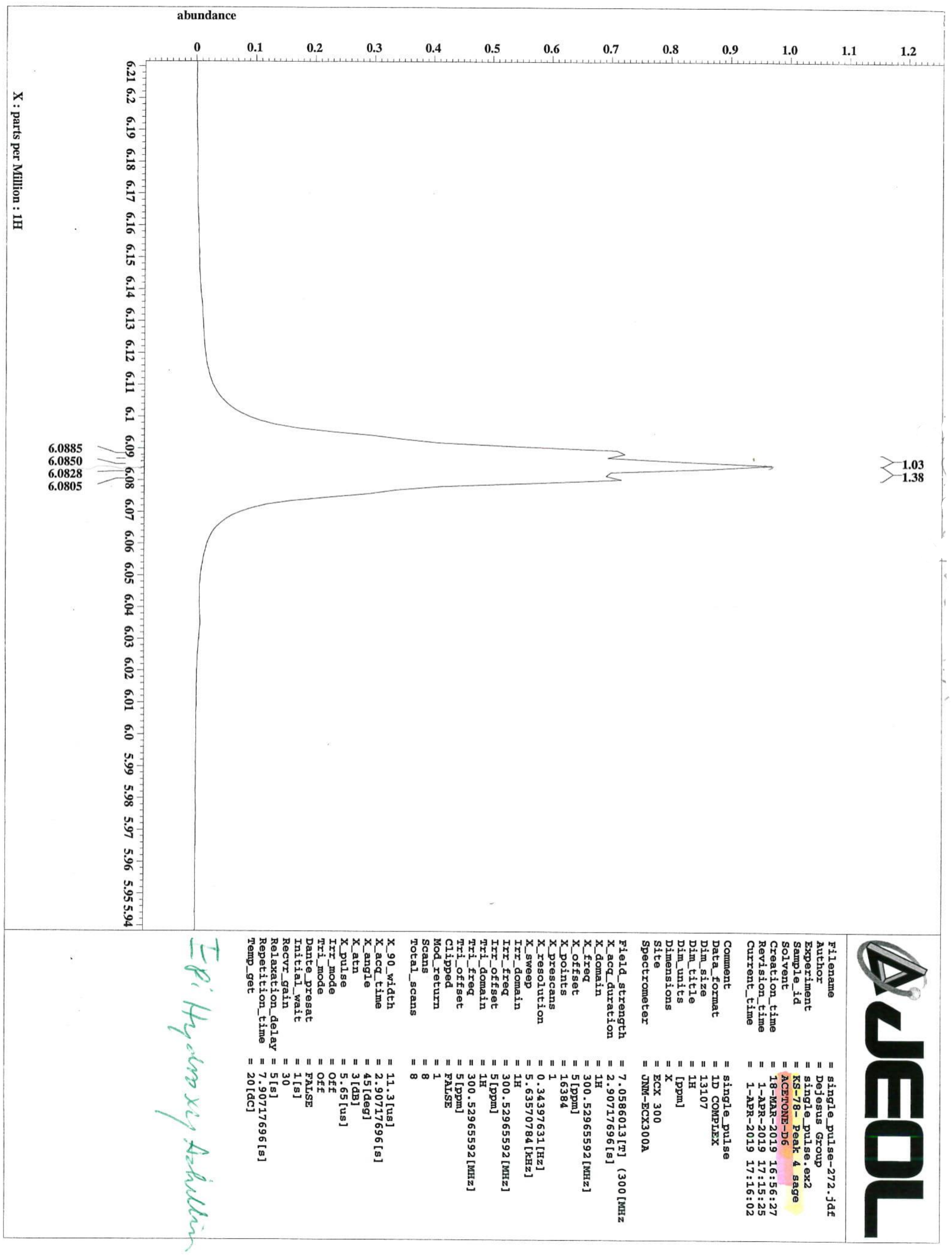

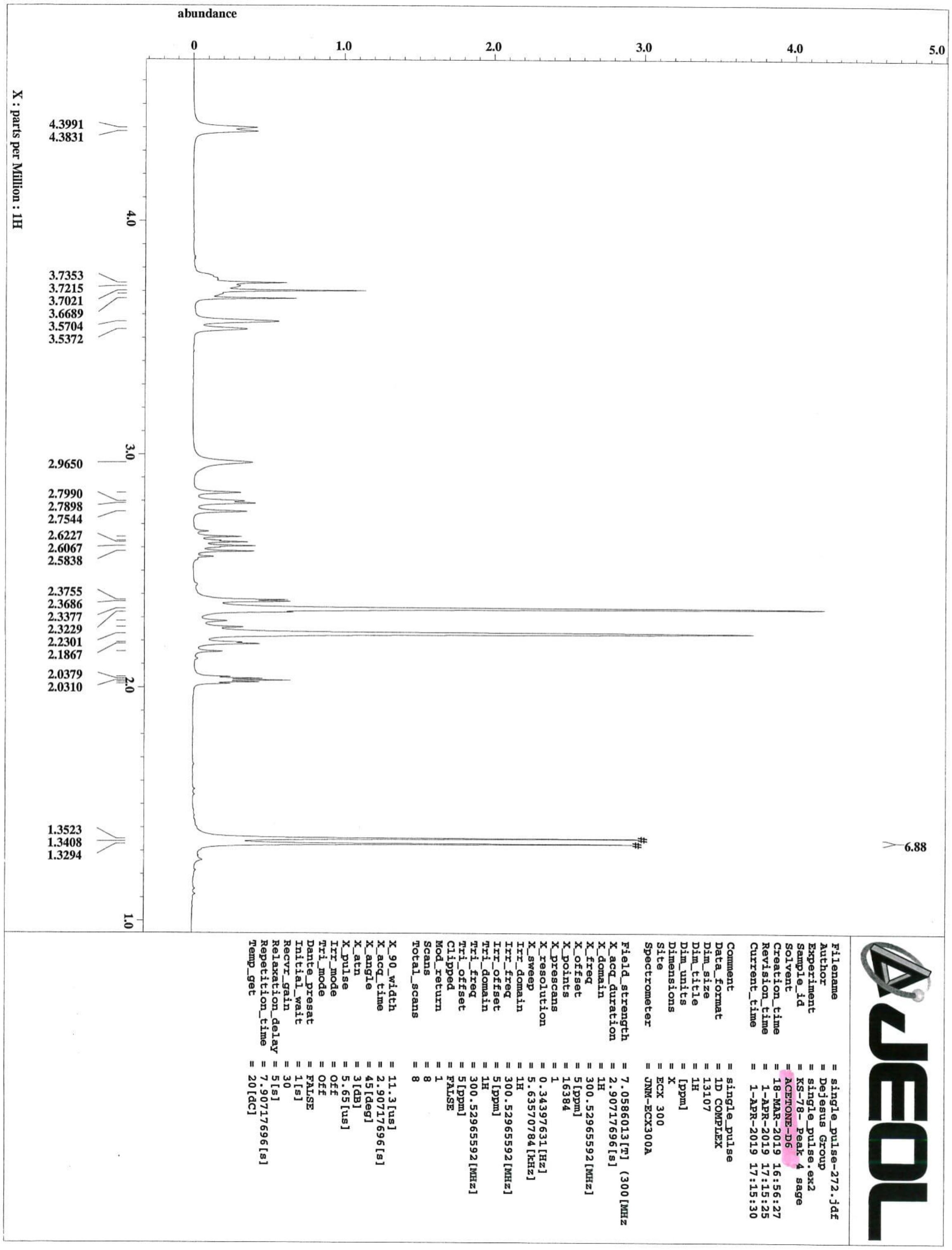
abundance
0
1.0
2.0
3.0
4.0
5.0
X : parts per Million : 1H
4.3991
4.3831
4.0
3.7353
3.7215
3.7021
3.6689
3.5704
3.5372
3.0
2.9650
2.7990
2.7898
2.7544
2.6227
2.6067
2.5838
2.3755
2.3686
2.3377
2.3229
2.2301
2.1867
2.0379
2.0310
2.0
1.3523
1.3408
1.3294
6.88
1.0
JEOL
Filename = single_pulse-272.jdf
Author = Dejesus Group
Experiment = single_pulse.ex2
Sample_id = KS-78- Peak 4 sage
Solvent = ACETONE-D6
Creation_time = 18-MAR-2019 16:56:27
Revision_time = 1-APR-2019 17:15:25
Current_time = 1-APR-2019 17:15:30
Comment = single_pulse
Data_format = 1D COMPLEX
Dim_size = 13107
Dim_title = 1H
Dim_units = [ppm]
Dimensions = X
Site = ECX 300
Spectrometer = JNM-ECX300A
Field_strength = 7.0586013[T] (300[MHz
X_acq_duration = 2.90717696[s]
X_domain = 1H
X_freq = 300.52965592[MHz]
X_offset = 5[ppm]
X_points = 16384
X_prescans = 1
X_resolution = 0.34397631[Hz]
X_sweep = 5.63570784[kHz]
Irr_domain = 1H
Irr_freq = 300.52965592[MHz]
Irr_offset = 5[ppm]
Tri_domain = 1H
Tri_freq = 300.52965592[MHz]
Tri_offset = 5[ppm]
Clipped = FALSE
Mod_return = 1
Scans = 8
Total_scans = 8
X_90_width = 11.3[us]
X_acq_time = 2.90717696[s]
X_angle = 45[deg]
X_atn = 3[dB]
X_pulse = 5.65[us]
Irr_mode = Off
Tri_mode = Off
Dante_presat = FALSE
Initial_wait = 1[s]
Recvr_gain = 30
Relaxation_delay = 5[s]
Repetition_time = 7.90717696[s]
Temp_get = 20[dC]

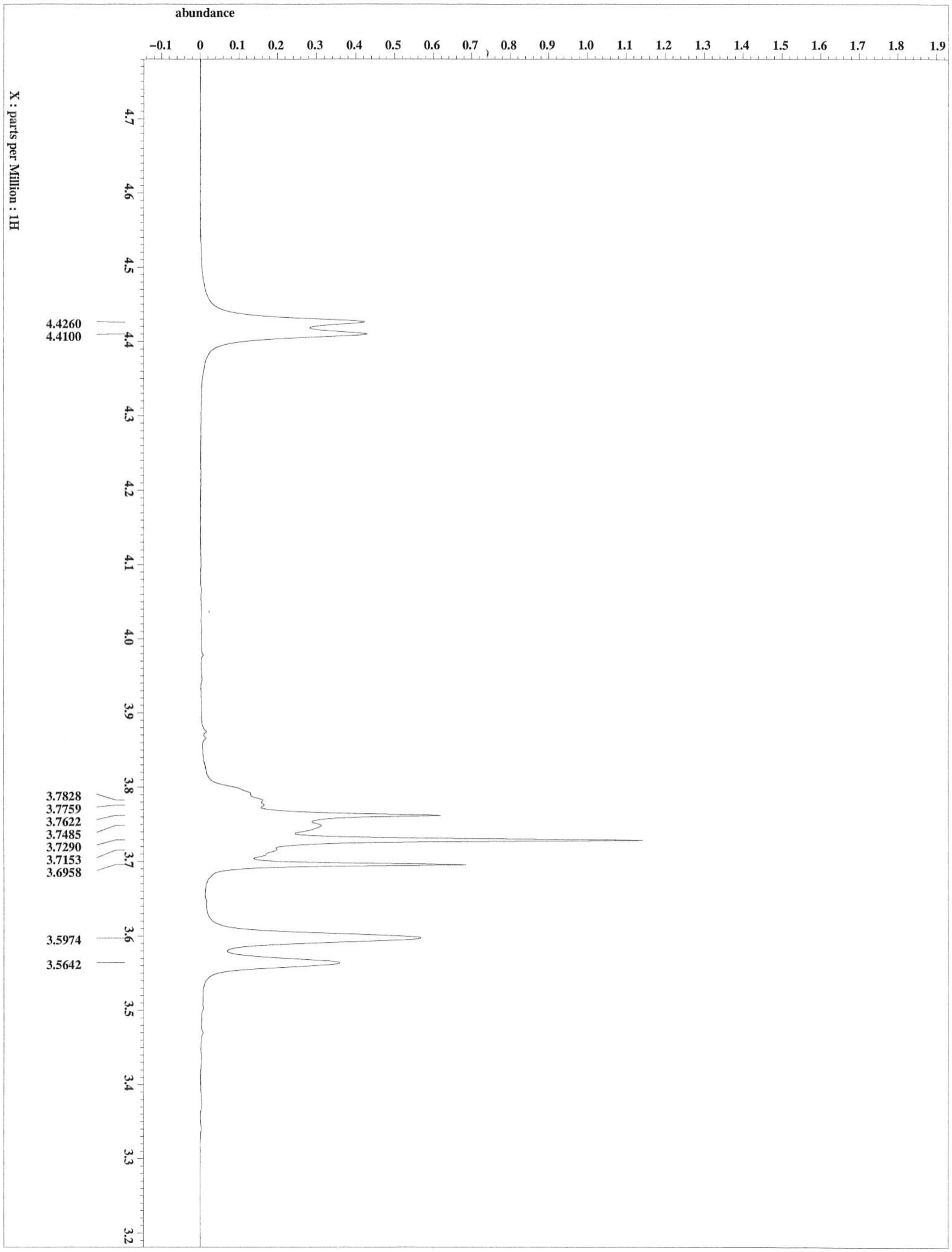
abundance
X : parts per Million : 1H
4.4260
4.4100
3.7828
3.7759
3.7622
3.7485
3.7290
3.7153
3.6958
3.5974
3.5642

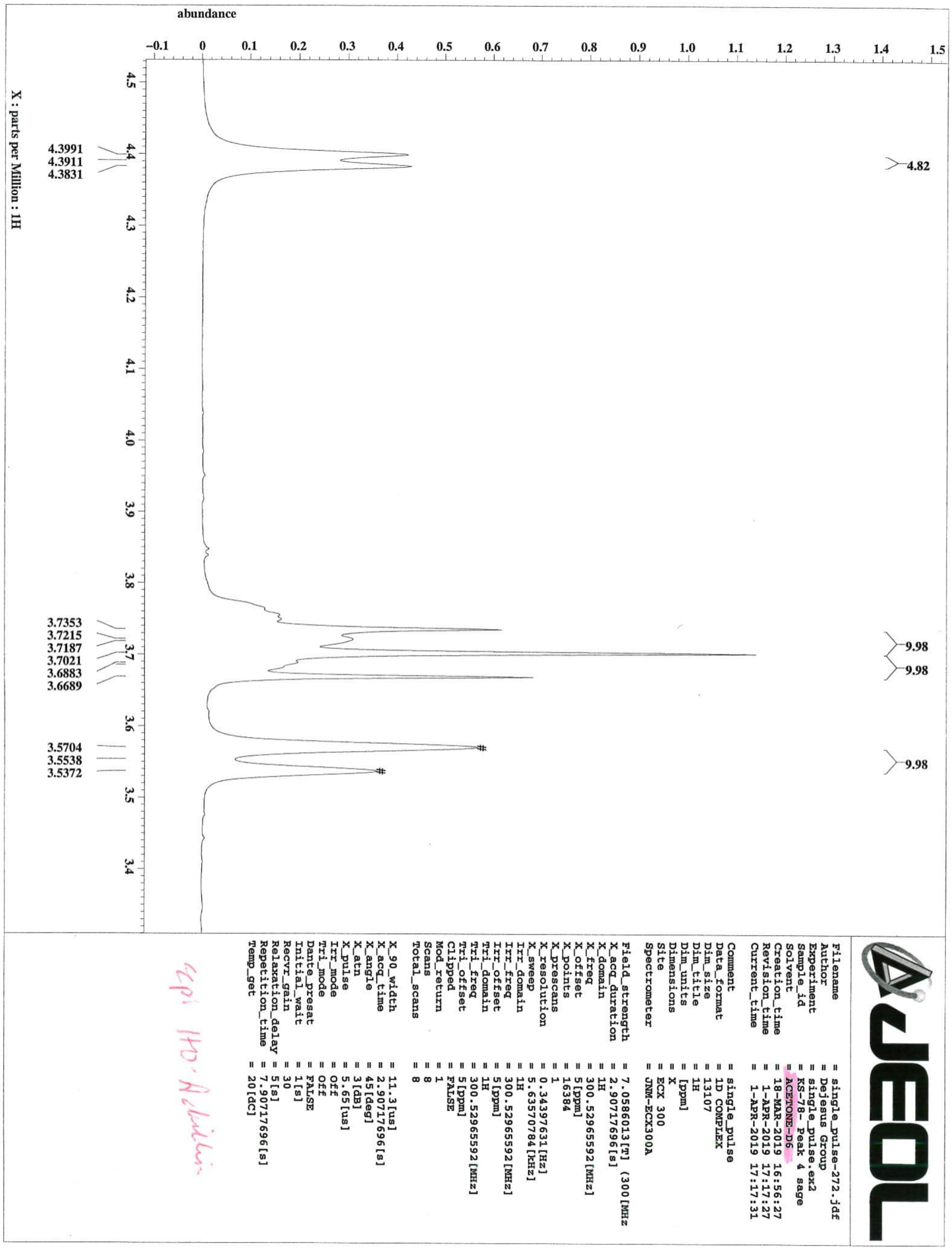

abundance
X : parts per Million : 1H
4.3991
4.3911
4.3831
3.7353
3.7215
3.7187
3.7021
3.6883
3.6689
3.5704
3.5538
3.5372
4.82
9.98
9.98
9.98
JEOL
Filename = single_pulse-272.jdf
Author = Dejesus Group
Experiment = single_pulse.ex2
Sample_id = KS-78- Peak 4 sage
Solvent = ACETONE-D6
Creation_time = 18-MAR-2019 16:56:27
Revision_time = 1-APR-2019 17:17:27
Current_time = 1-APR-2019 17:17:31
Comment = single_pulse
Data_format = 1D COMPLEX
Dim_size = 13107
Dim_title = 1H
Dim_units = [ppm]
Dimensions = X
Site = ECX 300
Spectrometer = JNM-ECX300A
Field_strength = 7.0586013[T] (300[MHz
X_acq_duration = 2.9071696[s]
X_domain = 1H
X_freq = 300.52965592[MHz]
X_offset = 5[ppm]
X_points = 16384
X_prescans = 1
X_resolution = 0.34397631[Hz]
X_sweep = 5.63570784[kHz]
Irr_domain = 1H
Irr_freq = 300.52965592[MHz]
Irr_offset = 5[ppm]
Tri_domain = 1H
Tri_freq = 300.52965592[MHz]
Tri_offset = 5[ppm]
Clipped = FALSE
Mod_return = 1
Scans = 8
Total_scans = 8
X_90_width = 11.3[us]
X_acq_time = 2.9071696[s]
X_angle = 45[deg]
X_atn = 3[dB]
X_pulse = 5.65[us]
Irr_mode = Off
Tri_mode = Off
Dante_presat = FALSE
Initial_wait = 1[s]
Recvr_gain = 30
Relaxation_delay = 5[s]
Repetition_time = 7.9071696[s]
Temp_get = 20[dC]

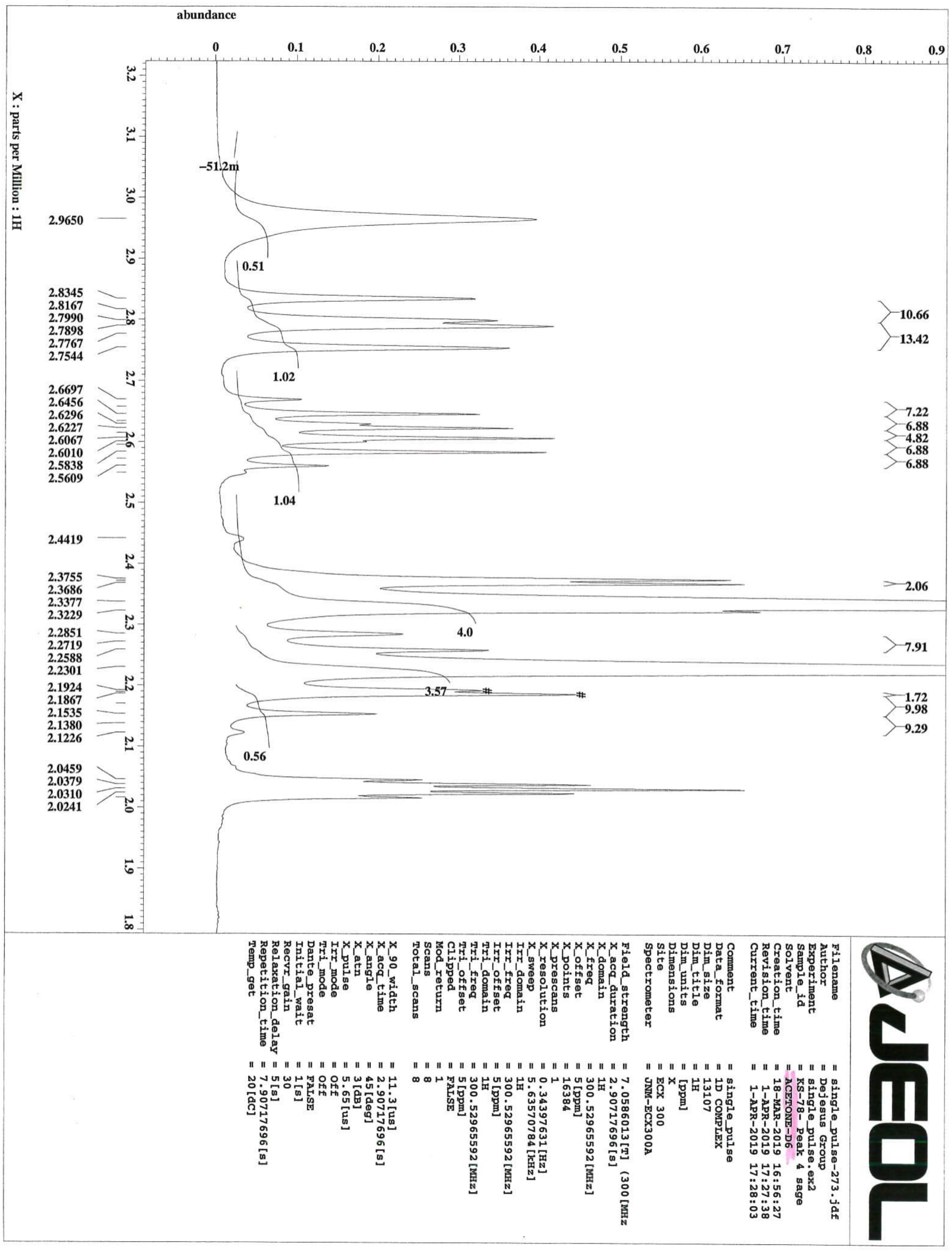
abundance
X : parts per Million : 1H
Filename = single_pulse-273.jdf
Author = Dejesus Group
Experiment = single_pulse.ex2
Sample_id = KS-78- Peak 4 sage
Solvent = ACETONE-D6
Creation_time = 18-MAR-2019 16:56:27
Revision_time = 1-APR-2019 17:27:38
Current_time = 1-APR-2019 17:28:03
Comment = single_pulse
Data_format = 1D COMPLEX
Dim_size = 13107
Dim_title = 1H
Dim_units = [ppm]
Dimensions = X
Site = ECX 300
Spectrometer = JNM-ECX300A
Field_strength = 7.0586013[T] (300[MHz
X_acq_duration = 2.9071769 6[s]
X_domain = 1H
X_freq = 300.52965592[MHz]
X_offset = 5[ppm]
X_points = 16384
X_prescans = 1
X_resolution = 0.34397631[Hz]
X_sweep = 5.63570784[kHz]
Irr_domain = 1H
Irr_freq = 300.52965592[MHz]
Irr_offset = 5[ppm]
Tri_domain = 1H
Tri_freq = 300.52965592[MHz]
Tri_offset = 5[ppm]
Clipped = FALSE
Mod_return = 1
Scans = 8
Total_scans = 8
X_90_width = 11.3[us]
X_acq_time = 2.9071769 6[s]
X_angle = 45[deg]
X_atn = 3[dB]
X_pulse = 5.65[us]
Irr_mode = Off
Tri_mode = Off
Dante_presat = FALSE
Initial_wait = 1[s]
Recvr_gain = 30
Relaxation_delay = 5[s]
Repetition_time = 7.9071769 6[s]
Temp_get = 20[dC]

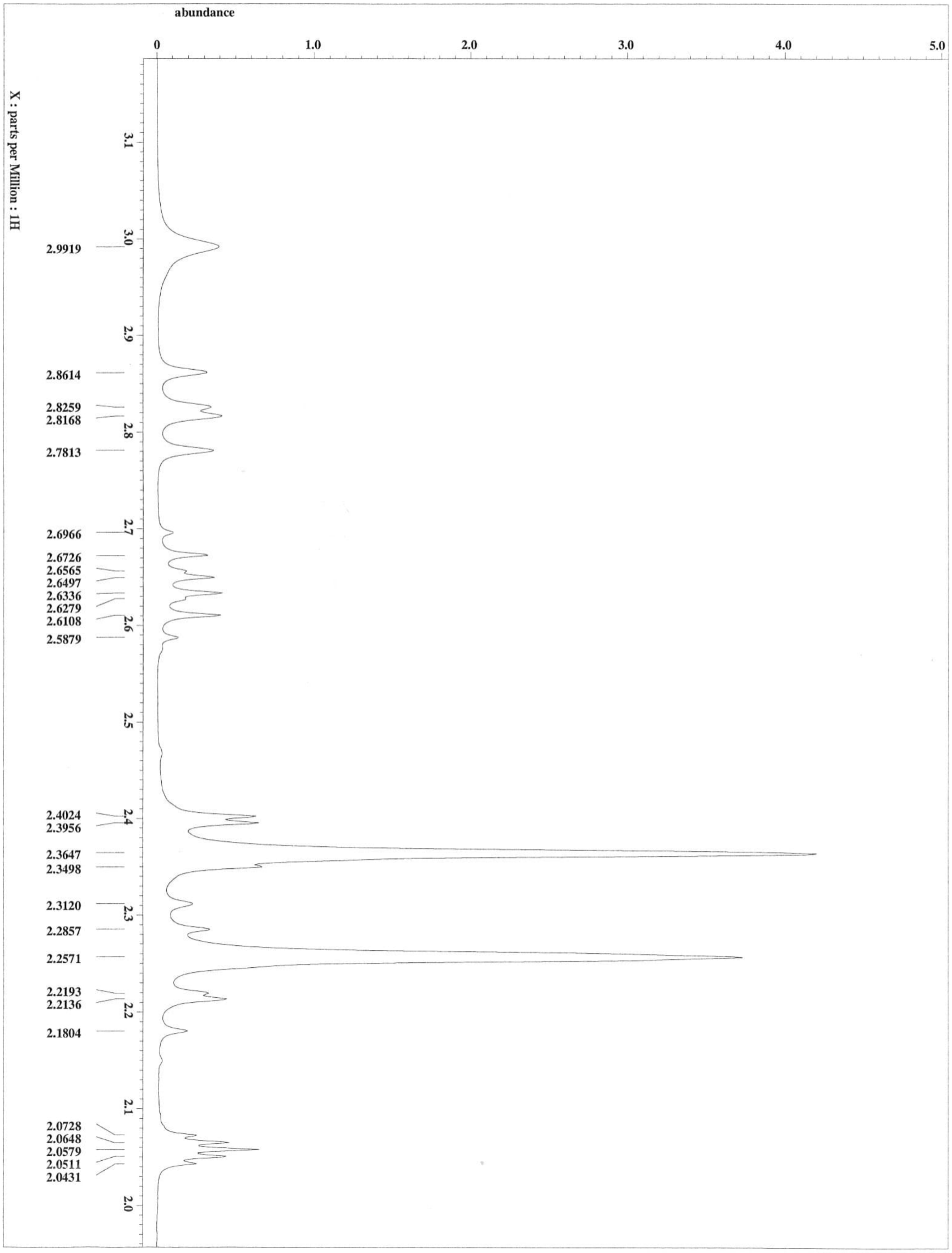
abundance
0
1.0
2.0
3.0
4.0
5.0
X : parts per Million : 1H
3.1
3.0
2.9
2.8
2.7
2.6
2.5
2.4
2.3
2.2
2.1
2.0
2.9919
2.8614
2.8259
2.8168
2.7813
2.6966
2.6726
2.6565
2.6497
2.6336
2.6279
2.6108
2.5879
2.4024
2.3956
2.3647
2.3498
2.3120
2.2857
2.2571
2.2193
2.2136
2.1804
2.0728
2.0648
2.0579
2.0511
2.0431

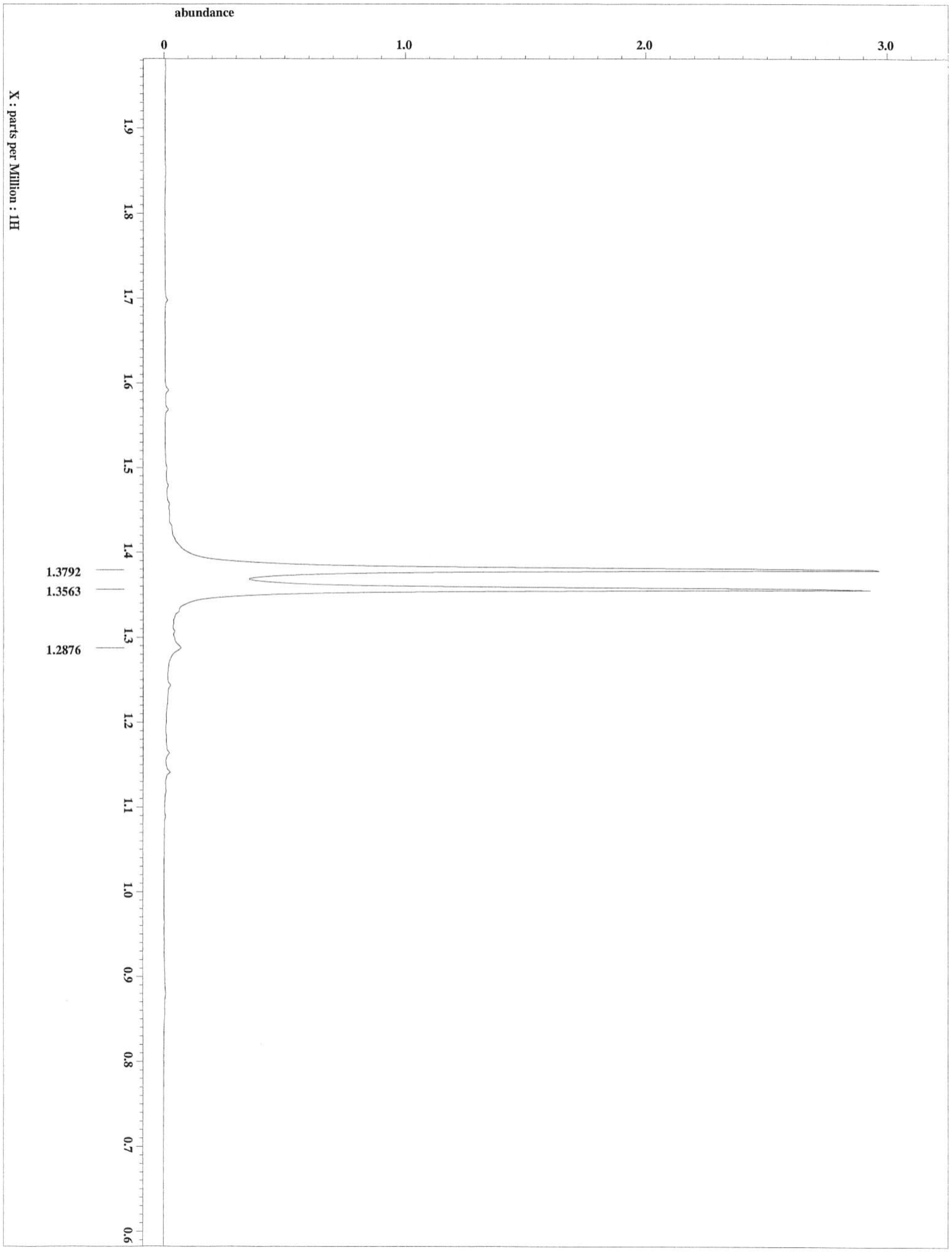
abundance
0
1.0
2.0
3.0
X : parts per Million : 1H
1.9
1.8
1.7
1.6
1.5
1.4
1.3792
1.3563
1.3
1.2876
1.2
1.1
1.0
0.9
0.8
0.7
0.6

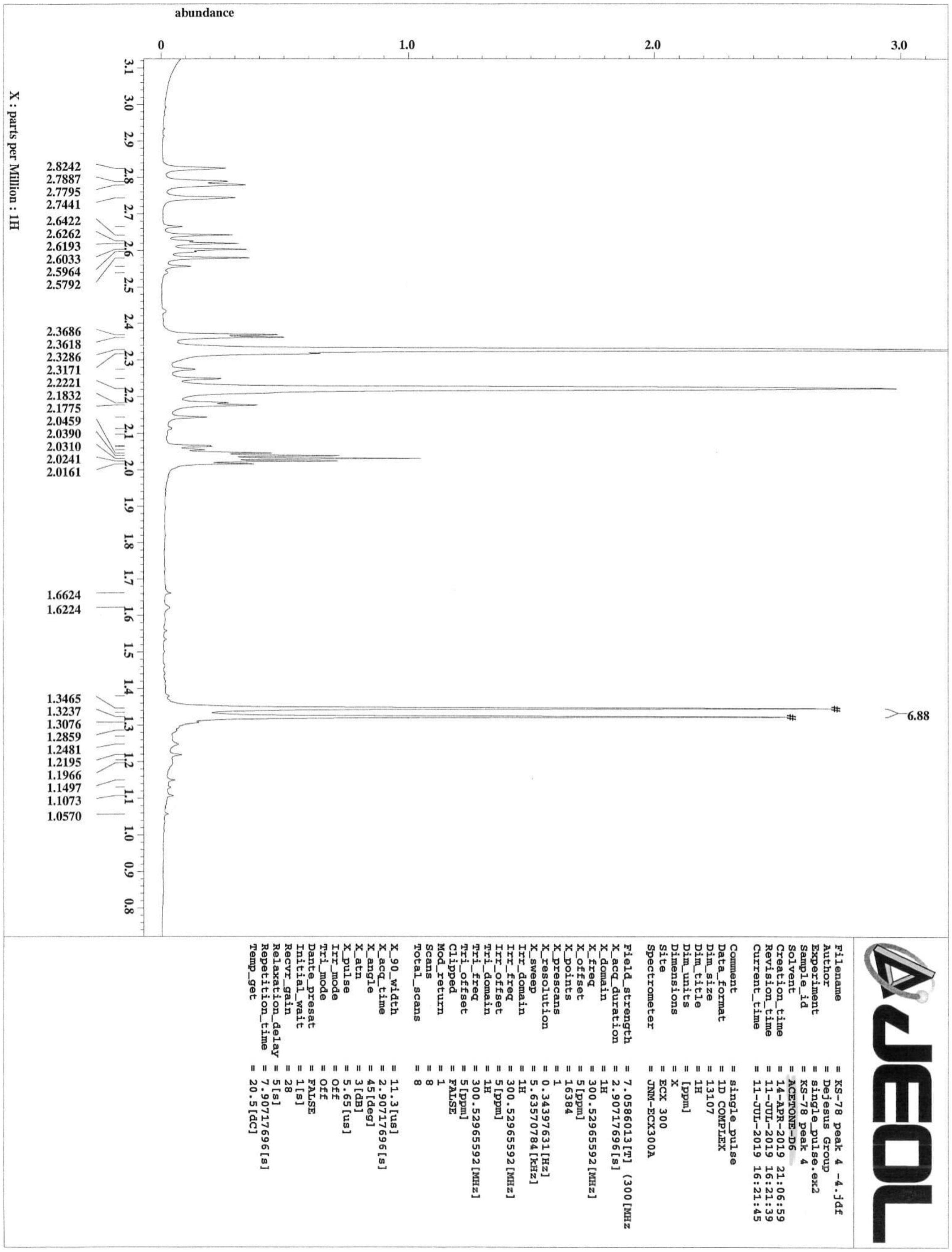
abundance
0
1.0
2.0
3.0
X : parts per Million : 1H
2.8242
2.7887
2.7795
2.7441
2.6422
2.6262
2.6193
2.6033
2.5964
2.5792
2.3686
2.3618
2.3286
2.3171
2.2221
2.1832
2.1775
2.0459
2.0390
2.0310
2.0241
2.0161
1.6624
1.6224
1.3465
1.3237
1.3076
1.2859
1.2481
1.2195
1.1966
1.1497
1.1073
1.0570
6.88
Filename = KS-78 peak 4 -4.jdf
Author = Dejesus Group
Experiment = single_pulse.ex2
Sample_id = KS-78 peak 4
Solvent = ACETONE-D6
Creation_time = 14-APR-2019 21:06:59
Revision_time = 11-JUL-2019 16:21:39
Current_time = 11-JUL-2019 16:21:45
Comment = single_pulse
Data_format = 1D COMPLEX
Dim_size = 13107
Dim_title = 1H
Dim_units = [ppm]
Dimensions = X
Site = ECX 300
Spectrometer = JNM-ECX300A
Field_strength = 7.0586013[T] (300[MHz
X_acq_duration = 2.90717696[s]
X_domain = 1H
X_freq = 300.52965592[MHz]
X_offset = 5[ppm]
X_points = 16384
X_prescans = 1
X_resolution = 0.34397631[Hz]
X_sweep = 5.63570784[kHz]
Irr_domain = 1H
Irr_freq = 300.52965592[MHz]
Irr_offset = 5[ppm]
Tri_domain = 1H
Tri_freq = 300.52965592[MHz]
Tri_offset = 5[ppm]
Clipped = FALSE
Mod_return = 1
Scans = 8
Total_scans = 8
X_90_width = 11.3[us]
X_acq_time = 2.90717696[s]
X_angle = 45[deg]
X_atn = 3[dB]
X_pulse = 5.65[us]
Irr_mode = Off
Tri_mode = Off
Dante_presat = FALSE
Initial_wait = 1[s]
Recvr_gain = 28
Relaxation_delay = 5[s]
Repetition_time = 7.90717696[s]
Temp_get = 20.5[dC]
JEOL

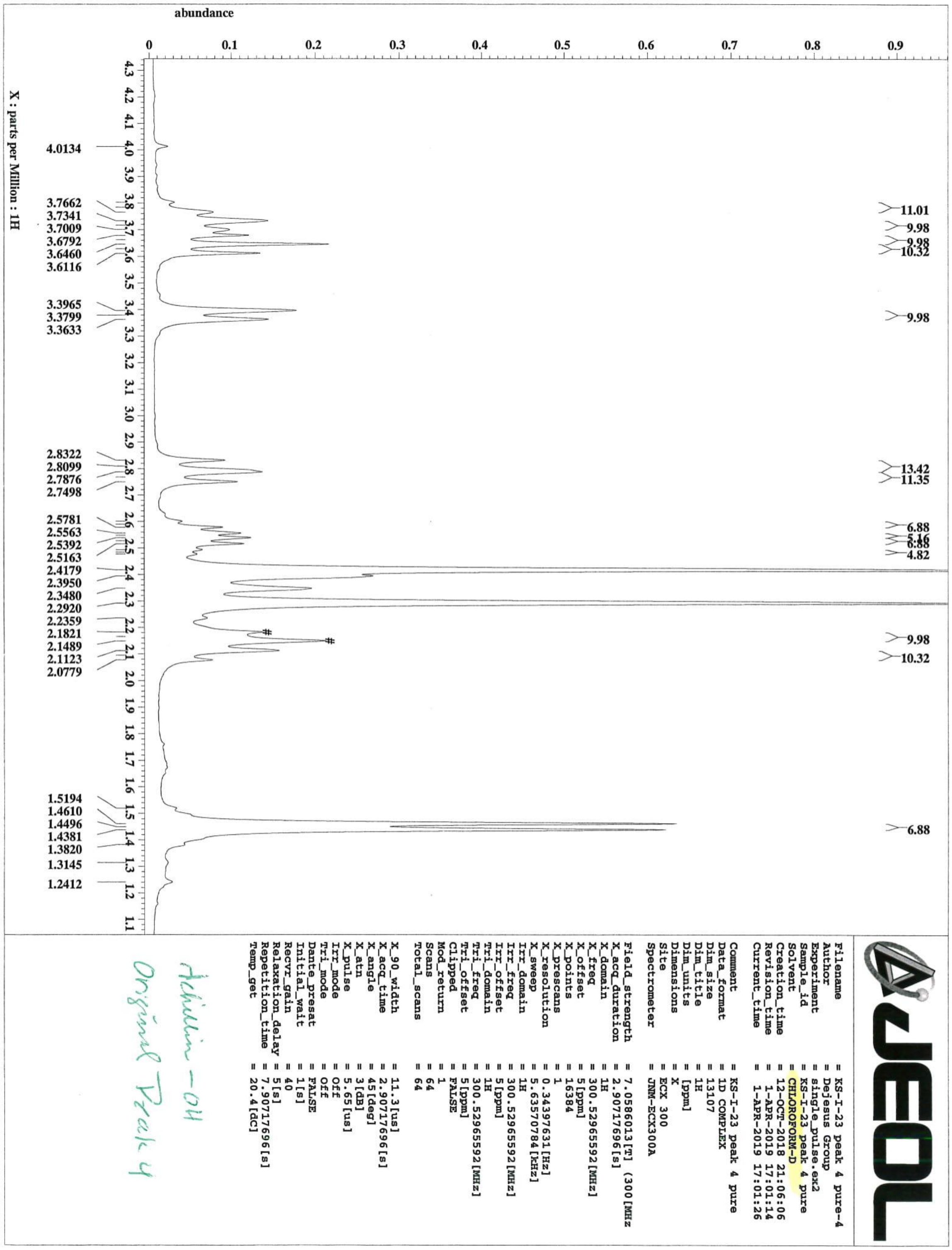
abundance
X : parts per Million : 1H
JEOL
Filename = KS-I-23 peak 4 pure-4
Author = Dejesus Group
Experiment = single_pulse.ex2
Sample_id = KS-I-23 peak 4 pure
Solvent = CHLOROFORM-D
Creation_time = 12-OCT-2018 21:06:06
Revision_time = 1-APR-2019 17:01:14
Current_time = 1-APR-2019 17:01:26
Comment = KS-I-23 peak 4 pure
Data_format = 1D COMPLEX
Dim_size = 13107
Dim_title = 1H
Dim_units = [ppm]
Dimensions = X
Site = ECX 300
Spectrometer = JNM-ECX300A
Field_strength = 7.0586013[T] (300[MHz
X_acq_duration = 2.90717696[s]
X_domain = 1H
X_freq = 300.52965592[MHz]
X_offset = 5[ppm]
X_points = 16384
X_prescans = 1
X_resolution = 0.34397631[Hz]
X_sweep = 5.63570784[kHz]
Irr_domain = 1H
Irr_freq = 300.52965592[MHz]
Irr_offset = 5[ppm]
Tri_domain = 1H
Tri_freq = 300.52965592[MHz]
Tri_offset = 5[ppm]
Clipped = FALSE
Mod_return = 1
Scans = 64
Total_scans = 64
X_90_width = 11.3[us]
X_acq_time = 2.90717696[s]
X_angle = 45[deg]
X_atn = 3[dB]
X_pulse = 5.65[us]
Irr_mode = Off
Tri_mode = Off
Dante_presat = FALSE
Initial_wait = 1[s]
Recvr_gain = 40
Relaxation_delay = 5[s]
Repetition_time = 7.90717696[s]
Temp_get = 20.4[dC]
Achillin -OH
Original Peak 4

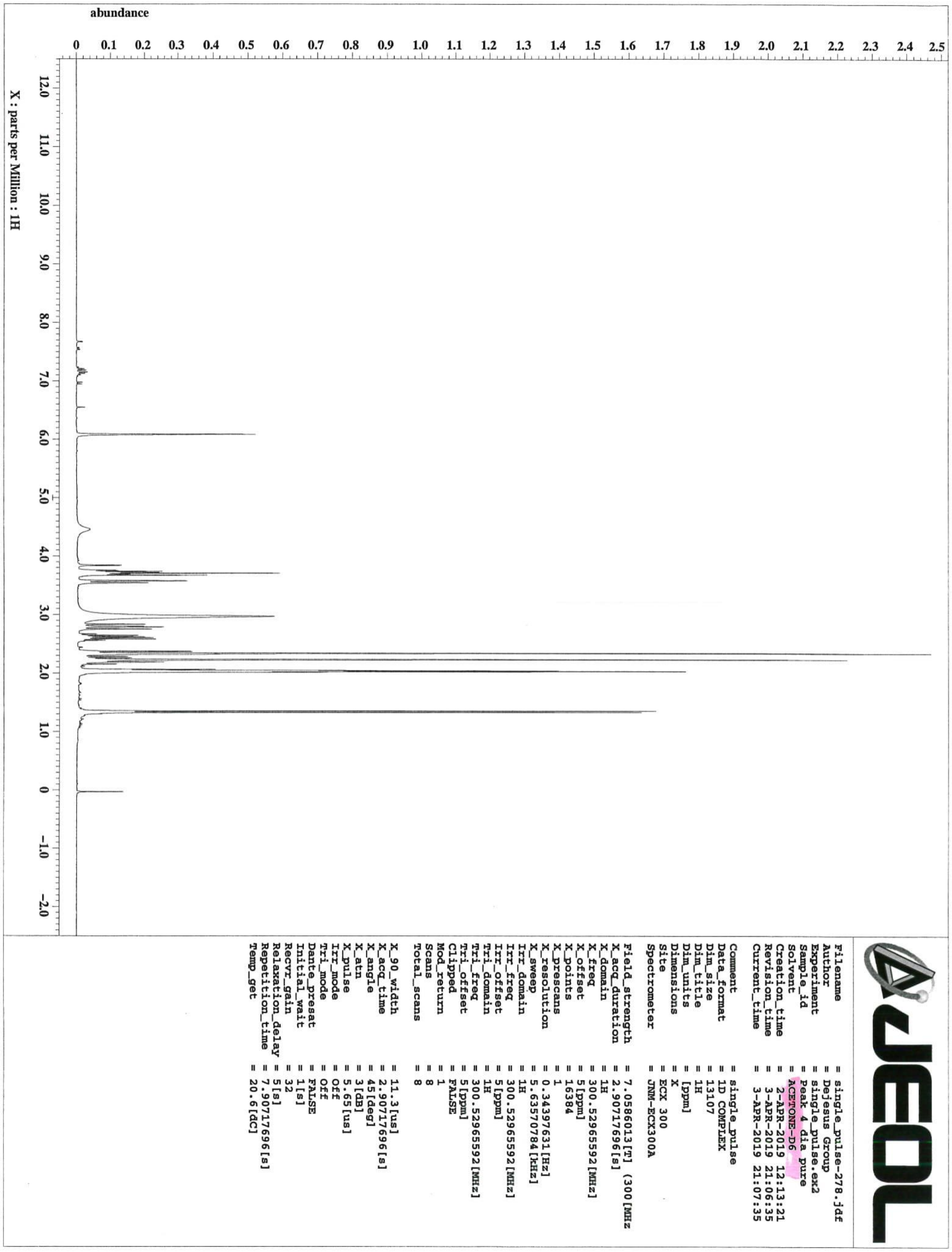
abundance
0 0.1 0.2 0.3 0.4 0.5 0.6 0.7 0.8 0.9 1.0 1.1 1.2 1.3 1.4 1.5 1.6 1.7 1.8 1.9 2.0 2.1 2.2 2.3 2.4 2.5
12.0 11.0 10.0 9.0 8.0 7.0 6.0 5.0 4.0 3.0 2.0 1.0 0 -1.0 -2.0
X : parts per Million : 1H
JEOL
Filename = single_pulse-278.jdf
Author = Dejesus Group
Experiment = single_pulse.ex2
Sample_id = Peak 4 dia pure
Solvent = ACETONE-D6
Creation_time = 2-APR-2019 12:13:21
Revision_time = 3-APR-2019 21:06:35
Current_time = 3-APR-2019 21:07:35
Comment = single_pulse
Data_format = 1D COMPLEX
Dim_size = 13107
Dim_title = 1H
Dim_units = [ppm]
Dimensions = X
Site = ECX 300
Spectrometer = JNM-ECX300A
Field_strength = 7.0586013[T] (300[MHz
X_acq_duration = 2.90717696[s]
X_domain = 1H
X_freq = 300.52965592[MHz]
X_offset = 5[ppm]
X_points = 16384
X_prescans = 1
X_resolution = 0.34397631[Hz]
X_sweep = 5.63570784[kHz]
Irr_domain = 1H
Irr_freq = 300.52965592[MHz]
Irr_offset = 5[ppm]
Tri_domain = 1H
Tri_freq = 300.52965592[MHz]
Tri_offset = 5[ppm]
Clipped = FALSE
Mod_return = 1
Scans = 8
Total_scans = 8
X_90_width = 11.3[us]
X_acq_time = 2.90717696[s]
X_angle = 45[deg]
X_atn = 3[dB]
X_pulse = 5.65[us]
Irr_mode = Off
Tri_mode = Off
Dante_presat = FALSE
Initial_wait = 1[s]
Recvr_gain = 32
Relaxation_delay = 5[s]
Repetition_time = 7.90717696[s]
Temp_get = 20.6[dC]

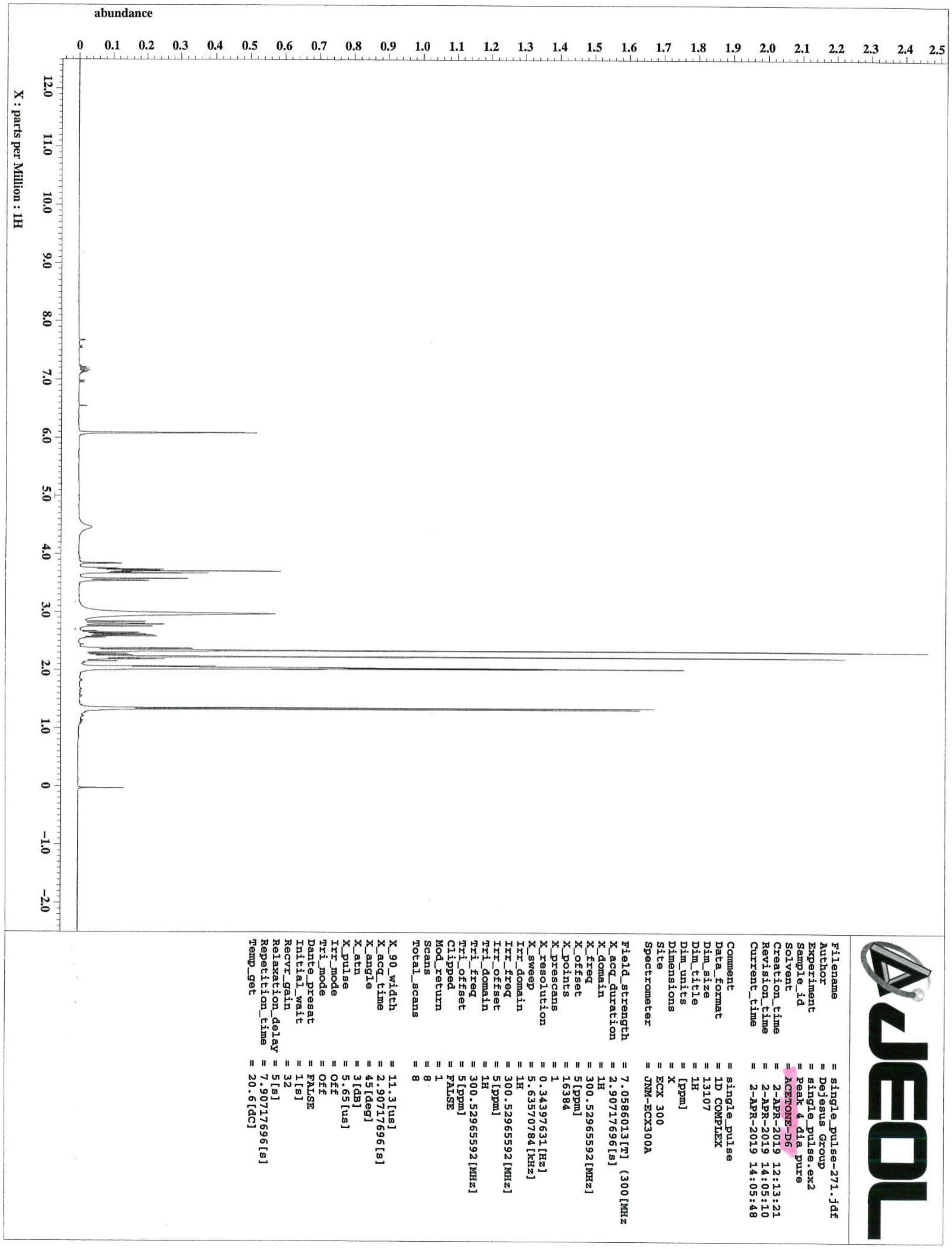
abundance
X : parts per Million : 1H
JEOL
Filename = single_pulse-271.jdf
Author = Dejesus Group
Experiment = single_pulse.ex2
Sample_id = Peak 4 dia pure
Solvent = ACETONE-D6
Creation_time = 2-APR-2019 12:13:21
Revision_time = 2-APR-2019 14:05:10
Current_time = 2-APR-2019 14:05:48
Comment = single_pulse
Data_format = 1D COMPLEX
Dim_size = 13107
Dim_title = 1H
Dim_units = [ppm]
Dimensions = X
Site = ECX 300
Spectrometer = JNM-ECX300A
Field_strength = 7.0586013[T] (300[MHz
X_acq_duration = 2.90717696[s]
X_domain = 1H
X_freq = 300.52965592[MHz]
X_offset = 5[ppm]
X_points = 16384
X_prescans = 1
X_resolution = 0.34397631[Hz]
X_sweep = 5.63570784[kHz]
Irr_domain = 1H
Irr_freq = 300.52965592[MHz]
Irr_offset = 5[ppm]
Tri_domain = 1H
Tri_freq = 300.52965592[MHz]
Tri_offset = 5[ppm]
Clipped = FALSE
Mod_return = 1
Scans = 8
Total_scans = 8
X_90_width = 11.3[us]
X_acq_time = 2.90717696[s]
X_angle = 45[deg]
X_atn = 3[dB]
X_pulse = 5.65[us]
Irr_mode = Off
Tri_mode = Off
Dante_presat = FALSE
Initial_wait = 1[s]
Recvr_gain = 32
Relaxation_delay = 5[s]
Repetition_time = 7.90717696[s]
Temp_get = 20.6[dC]

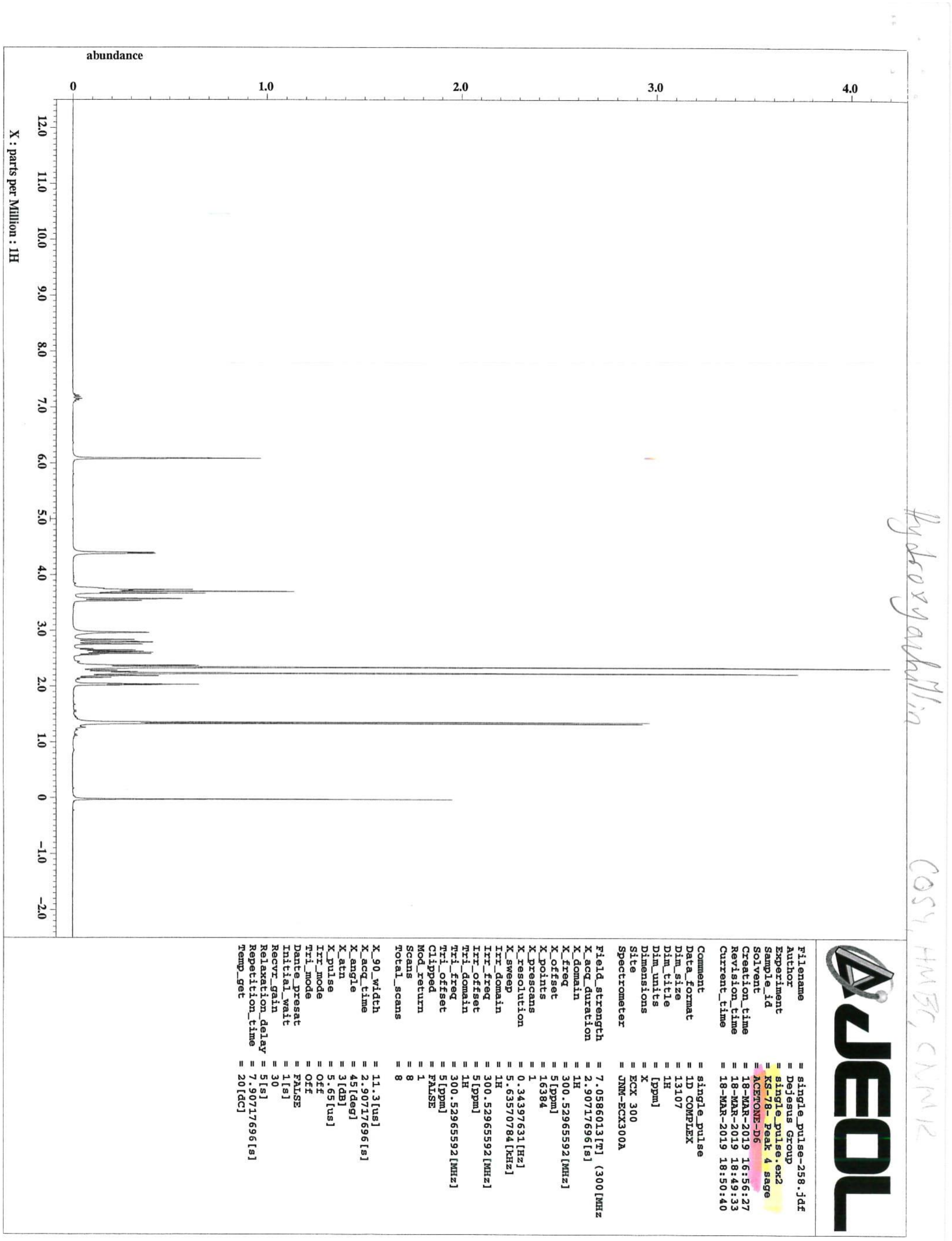
abundance
0
1.0
2.0
3.0
4.0
12.0
11.0
10.0
9.0
8.0
7.0
6.0
5.0
4.0
3.0
2.0
1.0
0
-1.0
-2.0
X : parts per Million : 1H
JEOL
Filename = single_pulse-258.jdf
Author = DeJesus Group
Experiment = single_pulse.ex2
Sample_id = KS-78- Peak 4 sage
Solvent = ACETONE-D6
Creation_time = 18-MAR-2019 16:56:27
Revision_time = 18-MAR-2019 18:49:33
Current_time = 18-MAR-2019 18:50:40
Comment = single_pulse
Data_format = 1D COMPLEX
Dim_size = 13107
Dim_title = 1H
Dim_units = [ppm]
Dimensions = X
Site = ECX 300
Spectrometer = JNM-ECX300A
Field_strength = 7.0586013[T] (300[MHz
X_acq_duration = 2.90717696[s]
X_domain = 1H
X_freq = 300.52965592[MHz]
X_offset = 5[ppm]
X_points = 16384
X_prescans = 1
X_resolution = 0.34397631[Hz]
X_sweep = 5.63570784[kHz]
Irr_domain = 1H
Irr_freq = 300.52965592[MHz]
Irr_offset = 5[ppm]
Tri_domain = 1H
Tri_freq = 300.52965592[MHz]
Tri_offset = 5[ppm]
Clipped = FALSE
Mod_return = 1
Scans = 8
Total_scans = 8
X_90_width = 11.3[us]
X_acq_time = 2.90717696[s]
X_angle = 45[deg]
X_atn = 3[dB]
X_pulse = 5.65[us]
Irr_mode = Off
Tri_mode = Off
Dante_presat = FALSE
Initial_wait = 1[s]
Recvr_gain = 30
Relaxation_delay = 5[s]
Repetition_time = 7.90717696[s]
Temp_get = 20[dC]
COSY, HMBC, CNMR

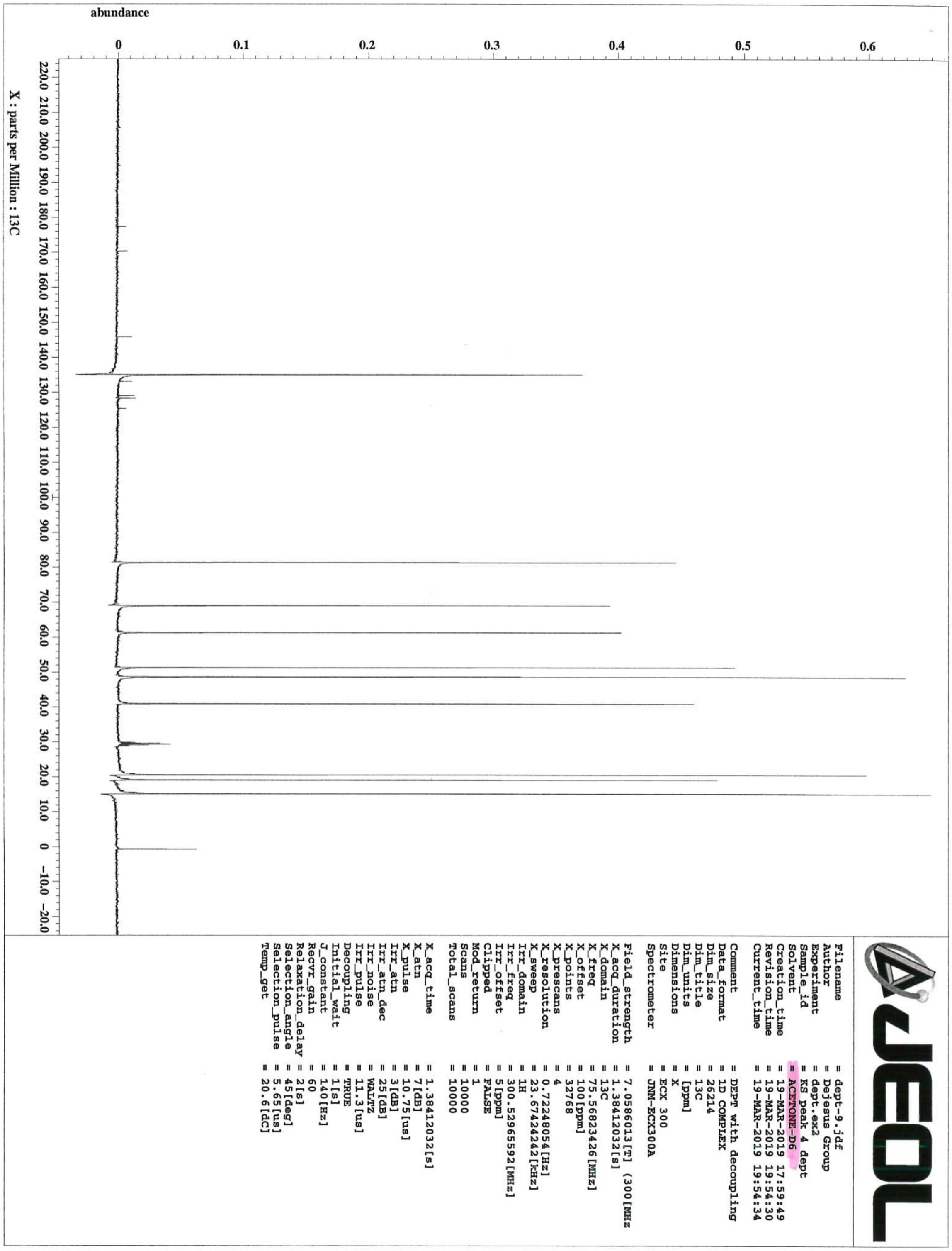
abundance
0
0.1
0.2
0.3
0.4
0.5
0.6
220.0 210.0 200.0 190.0 180.0 170.0 160.0 150.0 140.0 130.0 120.0 110.0 100.0 90.0 80.0 70.0 60.0 50.0 40.0 30.0 20.0 10.0 0 −10.0 −20.0
X : parts per Million : 13C
JEOL
Filename = dept-9.jdf
Author = Dejesus Group
Experiment = dept.ex2
Sample_id = KS peak 4 dept
Solvent = ACETONE-D6
Creation_time = 19-MAR-2019 17:59:49
Revision_time = 19-MAR-2019 19:54:30
Current_time = 19-MAR-2019 19:54:34
Comment = DEPT with decoupling
Data_format = 1D COMPLEX
Dim_size = 26214
Dim_title = 13C
Dim_units = [ppm]
Dimensions = X
Site = ECX 300
Spectrometer = JNM-ECX300A
Field_strength = 7.0586013[T] (300[MHz
X_acq_duration = 1.38412032[s]
X_domain = 13C
X_freq = 75.56823426[MHz]
X_offset = 100[ppm]
X_points = 32768
X_prescans = 4
X_resolution = 0.72248054[Hz]
X_sweep = 23.67424242[kHz]
Irr_domain = 1H
Irr_freq = 300.52965592[MHz]
Irr_offset = 5[ppm]
Clipped = FALSE
Mod_return = 1
Scans = 10000
Total_scans = 10000
X_acq_time = 1.38412032[s]
X_atn = 7[dB]
X_pulse = 10.75[us]
Irr_atn = 3[dB]
Irr_atn_dec = 25[dB]
Irr_noise = WALTZ
Irr_pulse = 11.3[us]
Decoupling = TRUE
Initial_wait = 1[s]
J_constant = 140[Hz]
Recvr_gain = 60
Relaxation_delay = 2[s]
Selection_angle = 45[deg]
Selection_pulse = 5.65[us]
Temp_get = 20.6[dC]

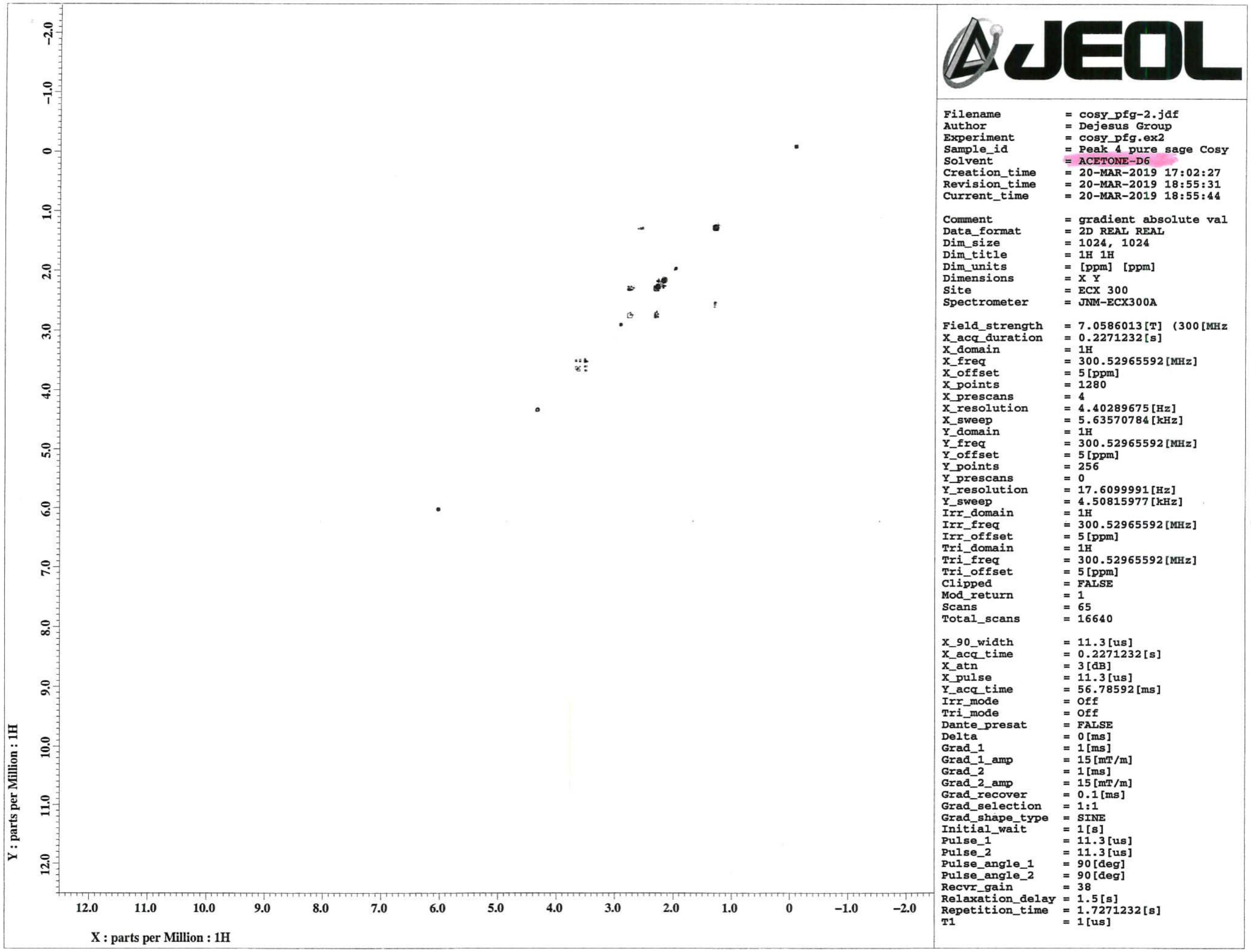
JEOL
Y : parts per Million : 1H
X : parts per Million : 1H
12.0 11.0 10.0 9.0 8.0 7.0 6.0 5.0 4.0 3.0 2.0 1.0 0 −1.0 −2.0
Filename = cosy_pfg-2.jdf
Author = Dejesus Group
Experiment = cosy_pfg.ex2
Sample_id = Peak 4 pure sage Cosy
Solvent = ACETONE-D6
Creation_time = 20-MAR-2019 17:02:27
Revision_time = 20-MAR-2019 18:55:31
Current_time = 20-MAR-2019 18:55:44
Comment = gradient absolute val
Data_format = 2D REAL REAL
Dim_size = 1024, 1024
Dim_title = 1H 1H
Dim_units = [ppm] [ppm]
Dimensions = X Y
Site = ECX 300
Spectrometer = JNM-ECX300A
Field_strength = 7.0586013[T] (300[MHz
X_acq_duration = 0.2271232[s]
X_domain = 1H
X_freq = 300.52965592[MHz]
X_offset = 5[ppm]
X_points = 1280
X_prescans = 4
X_resolution = 4.40289675[Hz]
X_sweep = 5.63570784[kHz]
Y_domain = 1H
Y_freq = 300.52965592[MHz]
Y_offset = 5[ppm]
Y_points = 256
Y_prescans = 0
Y_resolution = 17.6099991[Hz]
Y_sweep = 4.50815977[kHz]
Irr_domain = 1H
Irr_freq = 300.52965592[MHz]
Irr_offset = 5[ppm]
Tri_domain = 1H
Tri_freq = 300.52965592[MHz]
Tri_offset = 5[ppm]
Clipped = FALSE
Mod_return = 1
Scans = 65
Total_scans = 16640
X_90_width = 11.3[us]
X_acq_time = 0.2271232[s]
X_atn = 3[dB]
X_pulse = 11.3[us]
Y_acq_time = 56.78592[ms]
Irr_mode = Off
Tri_mode = Off
Dante_presat = FALSE
Delta = 0[ms]
Grad_1 = 1[ms]
Grad_1_amp = 15[mT/m]
Grad_2 = 1[ms]
Grad_2_amp = 15[mT/m]
Grad_recover = 0.1[ms]
Grad_selection = 1:1
Grad_shape_type = SINE
Initial_wait = 1[s]
Pulse_1 = 11.3[us]
Pulse_2 = 11.3[us]
Pulse_angle_1 = 90[deg]
Pulse_angle_2 = 90[deg]
Recvr_gain = 38
Relaxation_delay = 1.5[s]
Repetition_time = 1.7271232[s]
T1 = 1[us]

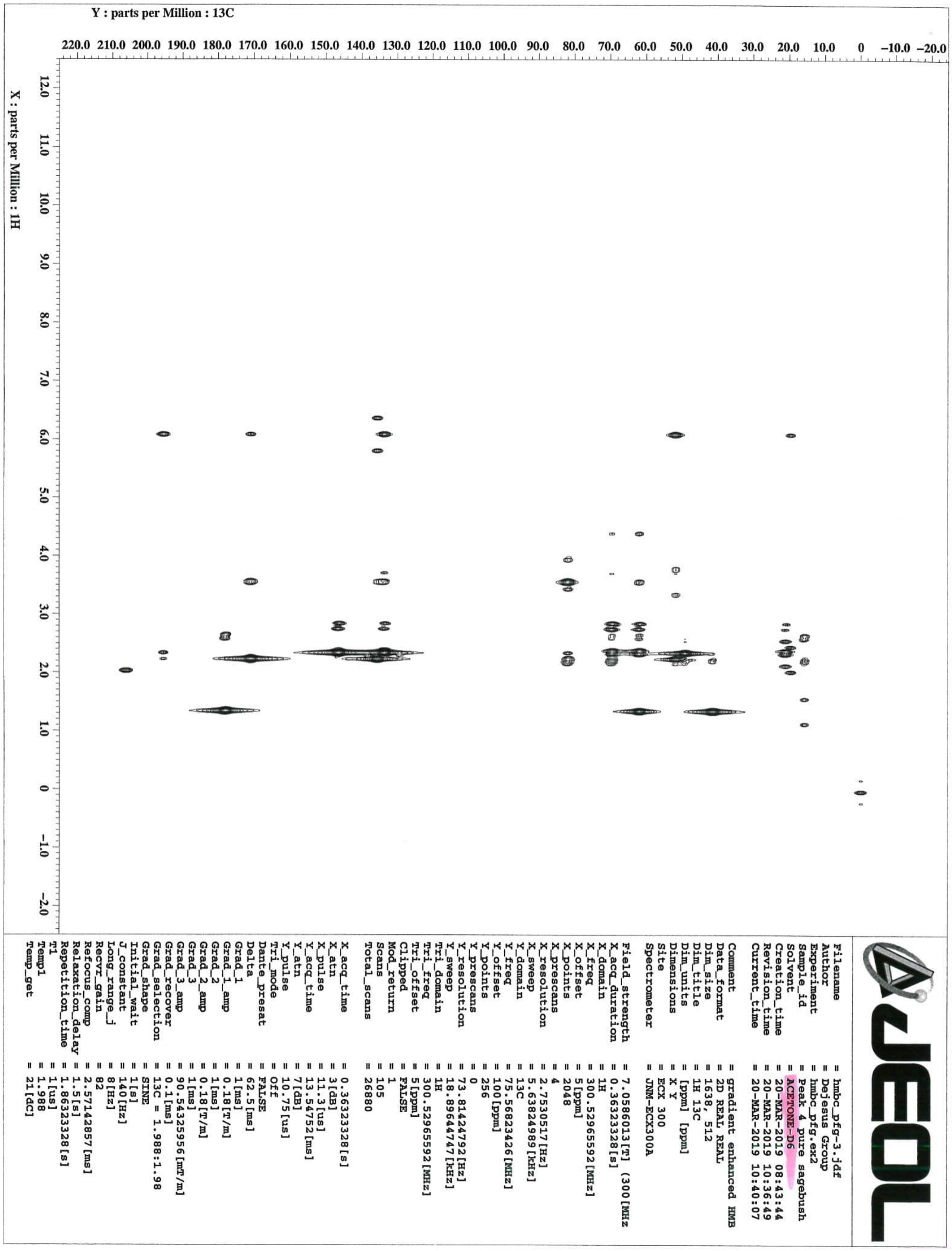
Y : parts per Million : 13C
X : parts per Million : 1H
JEOL
Filename = hmbc_pfg-3.jdf
Author = Dejesus Group
Experiment = hmbc_pfg.ex2
Sample_id = Peak 4 pure sagebush
Solvent = ACETONE-D6
Creation_time = 20-MAR-2019 08:43:44
Revision_time = 20-MAR-2019 10:36:49
Current_time = 20-MAR-2019 10:40:07
Comment = gradient enhanced HMB
Data_format = 2D REAL REAL
Dim_size = 1638, 512
Dim_title = 1H 13C
Dim_units = [ppm] [ppm]
Dimensions = X Y
Site = ECX 300
Spectrometer = JNM-ECX300A
Field_strength = 7.0586013[T] (300[MHz
X_acq_duration = 0.36323328[s]
X_domain = 1H
X_freq = 300.52965592[MHz]
X_offset = 5[ppm]
X_points = 2048
X_prescans = 4
X_resolution = 2.7530517[Hz]
X_sweep = 5.63824989[kHz]
Y_domain = 13C
Y_freq = 75.56823426[MHz]
Y_offset = 100[ppm]
Y_points = 256
Y_prescans = 0
Y_resolution = 73.81424792[Hz]
Y_sweep = 18.89644747[kHz]
Tri_domain = 1H
Tri_freq = 300.52965592[MHz]
Tri_offset = 5[ppm]
Clipped = FALSE
Mod_return = 1
Scans = 105
Total_scans = 26880
X_acq_time = 0.36323328[s]
X_atn = 3[dB]
X_pulse = 11.3[us]
Y_acq_time = 13.54752[ms]
Y_atn = 7[dB]
Y_pulse = 10.75[us]
Tri_mode = Off
Dante_presat = FALSE
Delta = 62.5[ms]
Grad_1 = 1[ms]
Grad_1_amp = 0.18[T/m]
Grad_2 = 1[ms]
Grad_2_amp = 0.18[T/m]
Grad_3 = 1[ms]
Grad_3_amp = 90.54325956[mT/m]
Grad_recover = 0.1[ms]
Grad_selection = 13C = 1.988:1.98
Grad_shape = SINE
Initial_wait = 1[s]
J_constant = 140[Hz]
Long_range_j = 8[Hz]
Recvr_gain = 82
Refocus_comp = 2.57142857[ms]
Relaxation_delay = 1.5[s]
Repetition_time = 1.86323328[s]
T1 = 1[us]
Temp1 = 1.988
Temp_get = 21[dC]

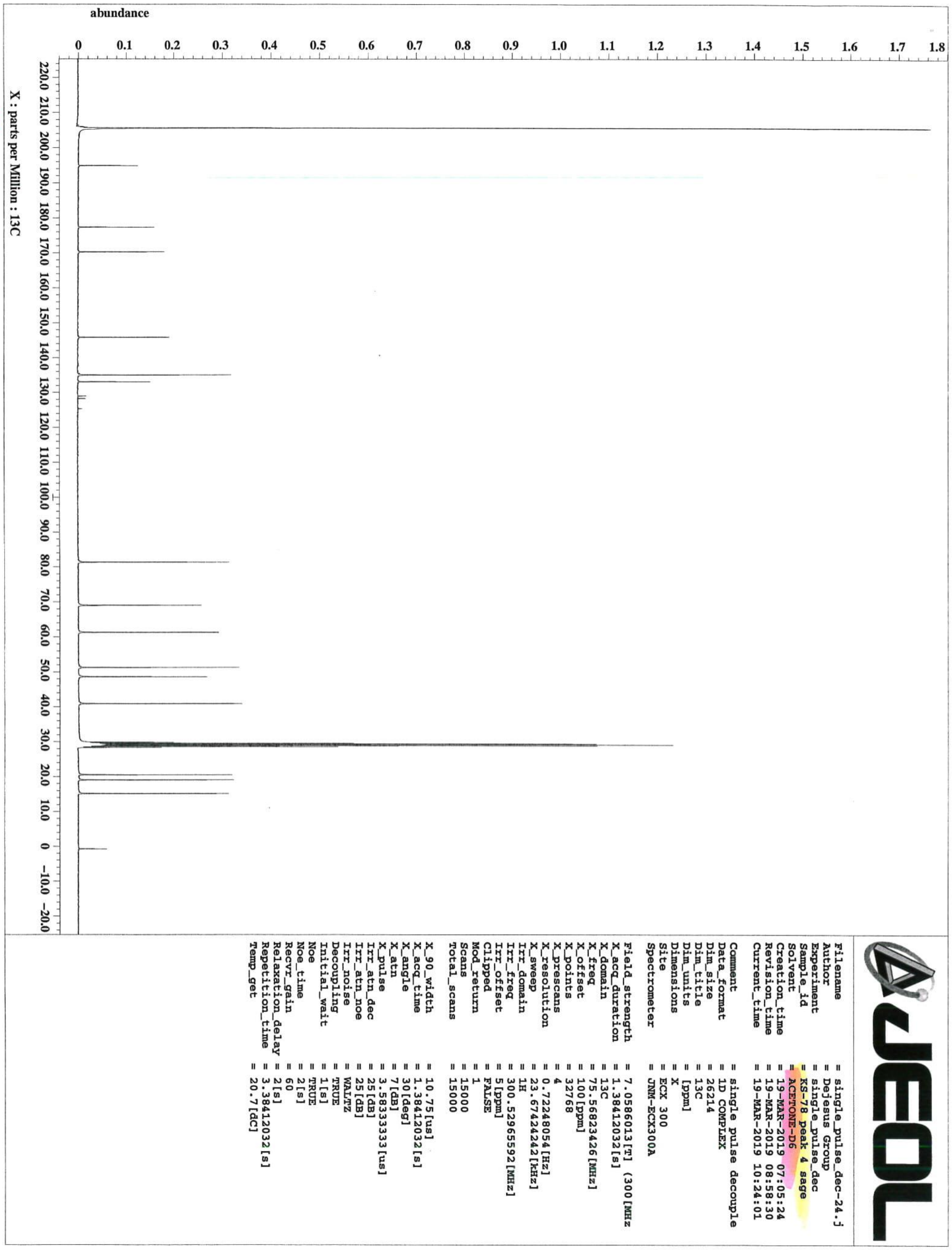
abundance
0 0.1 0.2 0.3 0.4 0.5 0.6 0.7 0.8 0.9 1.0 1.1 1.2 1.3 1.4 1.5 1.6 1.7 1.8
220.0 210.0 200.0 190.0 180.0 170.0 160.0 150.0 140.0 130.0 120.0 110.0 100.0 90.0 80.0 70.0 60.0 50.0 40.0 30.0 20.0 10.0 0 −10.0 −20.0
X : parts per Million : 13C
JEOL
Filename = single_pulse_dec-24.j
Author = Dejesus Group
Experiment = single_pulse_dec
Sample_id = KS-78 peak 4 sage
Solvent = ACETONE-D6
Creation_time = 19-MAR-2019 07:05:24
Revision_time = 19-MAR-2019 08:58:30
Current_time = 19-MAR-2019 10:24:01
Comment = single pulse decouple
Data_format = 1D COMPLEX
Dim_size = 26214
Dim_title = 13C
Dim_units = [ppm]
Dimensions = X
Site = ECX 300
Spectrometer = JNM-ECX300A
Field_strength = 7.0586013[T] (300[MHz
X_acq_duration = 1.38412032[s]
X_domain = 13C
X_freq = 75.56823426[MHz]
X_offset = 100[ppm]
X_points = 32768
X_prescans = 4
X_resolution = 0.72248054[Hz]
X_sweep = 23.67424242[kHz]
Irr_domain = 1H
Irr_freq = 300.52965592[MHz]
Irr_offset = 5[ppm]
Clipped = FALSE
Mod_return = 1
Scans = 15000
Total_scans = 15000
X_90_width = 10.75[us]
X_acq_time = 1.38412032[s]
X_angle = 30[deg]
X_atn = 7[dB]
X_pulse = 3.58333333[us]
Irr_atn_dec = 25[dB]
Irr_atn_noe = 25[dB]
Irr_noise = WALTZ
Decoupling = TRUE
Initial_wait = 1[s]
Noe = TRUE
Noe_time = 2[s]
Recvr_gain = 60
Relaxation_delay = 2[s]
Repetition_time = 3.38412032[s]
Temp_get = 20.7[dC]

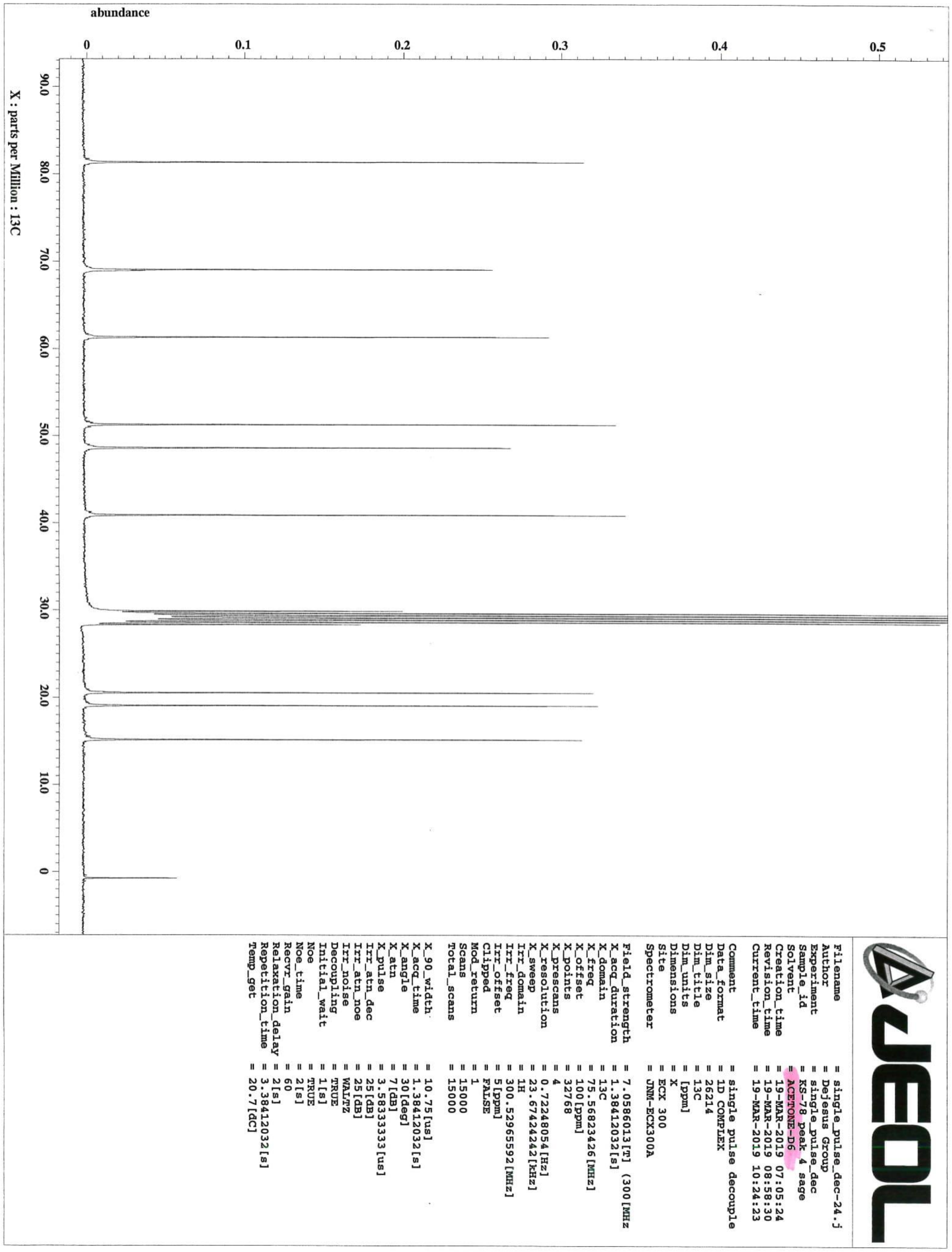
abundance
X : parts per Million : 13C
JEOL
Filename = single_pulse_dec-24.j
Author = Dejesus Group
Experiment = single_pulse_dec
Sample_id = KS-78 peak 4 sage
Solvent = ACETONE-D6
Creation_time = 19-MAR-2019 07:05:24
Revision_time = 19-MAR-2019 08:58:30
Current_time = 19-MAR-2019 10:24:23
Comment = single pulse decouple
Data_format = 1D COMPLEX
Dim_size = 26214
Dim_title = 13C
Dim_units = [ppm]
Dimensions = X
Site = ECX 300
Spectrometer = JNM-ECX300A
Field_strength = 7.0586013[T] (300[MHz
X_acq_duration = 1.38412032[s]
X_domain = 13C
X_freq = 75.56823426[MHz]
X_offset = 100[ppm]
X_points = 32768
X_prescans = 4
X_resolution = 0.72248054[Hz]
X_sweep = 23.67424242[kHz]
Irr_domain = 1H
Irr_freq = 300.52965592[MHz]
Irr_offset = 5[ppm]
Clipped = FALSE
Mod_return = 1
Scans = 15000
Total_scans = 15000
X_90_width = 10.75[us]
X_acq_time = 1.38412032[s]
X_angle = 30[deg]
X_atn = 7[dB]
X_pulse = 3.58333333[us]
Irr_atn_dec = 25[dB]
Irr_atn_noe = 25[dB]
Irr_noise = WALTZ
Decoupling = TRUE
Initial_wait = 1[s]
Noe = TRUE
Noe_time = 2[s]
Recvr_gain = 60
Relaxation_delay = 2[s]
Repetition_time = 3.38412032[s]
Temp_get = 20.7[dC]

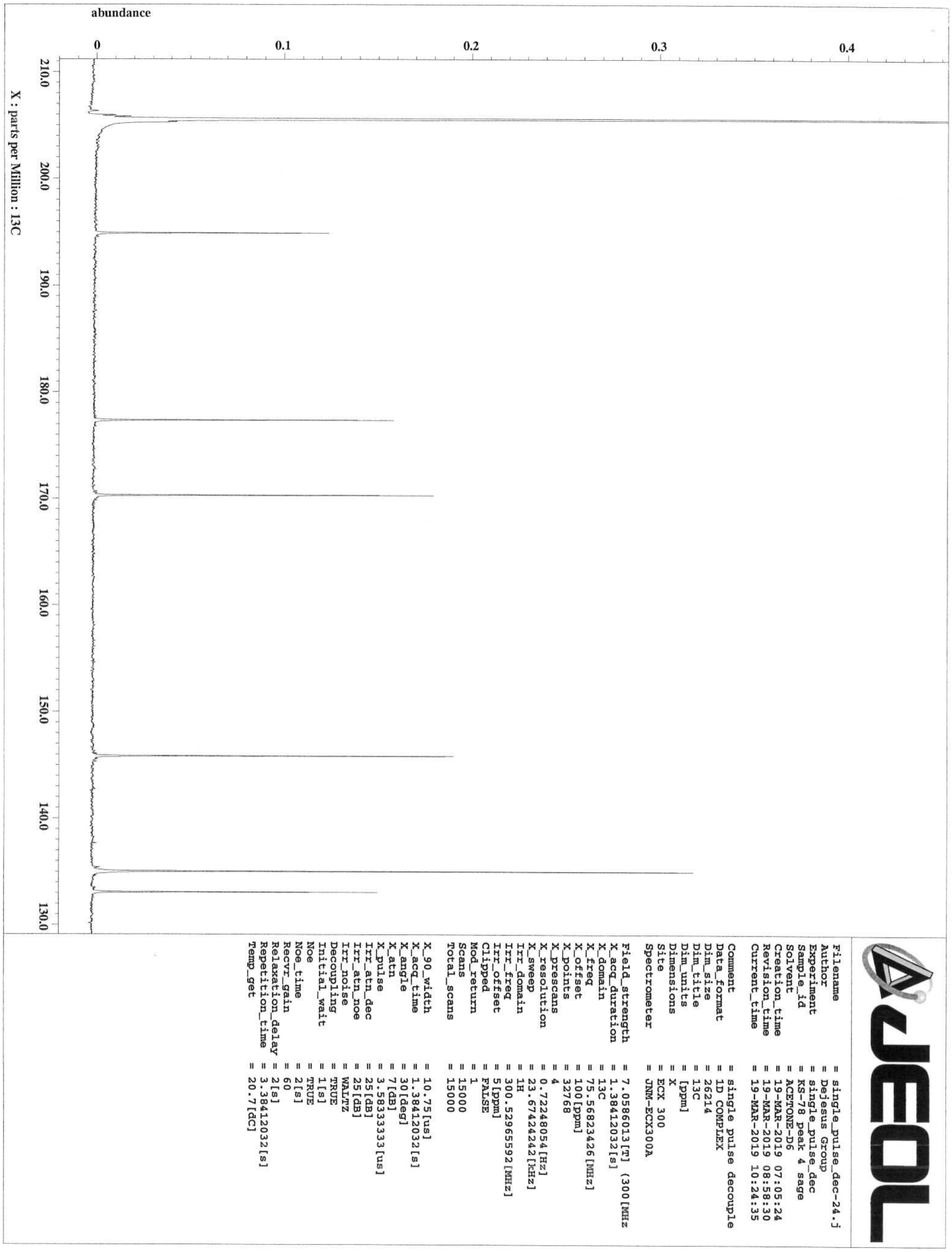
abundance
0
0.1
0.2
0.3
0.4
210.0
200.0
190.0
180.0
170.0
160.0
150.0
140.0
130.0
X : parts per Million : 13C
Filename = single_pulse_dec-24.j
Author = Dejesus Group
Experiment = single_pulse_dec
Sample_id = KS-78 peak 4 sage
Solvent = ACETONE-D6
Creation_time = 19-MAR-2019 07:05:24
Revision_time = 19-MAR-2019 08:58:30
Current_time = 19-MAR-2019 10:24:35
Comment = single pulse decouple
Data_format = 1D COMPLEX
Dim_size = 26214
Dim_title = 13C
Dim_units = [ppm]
Dimensions = X
Site = ECX 300
Spectrometer = JNM-ECX300A
Field_strength = 7.0586013[T] (300[MHz
X_acq_duration = 1.38412032[s]
X_domain = 13C
X_freq = 75.56823426[MHz]
X_offset = 100[ppm]
X_points = 32768
X_prescans = 4
X_resolution = 0.72248054[Hz]
X_sweep = 23.67424242[kHz]
Irr_domain = 1H
Irr_freq = 300.52965592[MHz]
Irr_offset = 5[ppm]
Clipped = FALSE
Mod_return = 1
Scans = 15000
Total_scans = 15000
X_90_width = 10.75[us]
X_acq_time = 1.38412032[s]
X_angle = 30[deg]
X_atn = 7[dB]
X_pulse = 3.58333333[us]
Irr_atn_dec = 25[dB]
Irr_atn_noe = 25[dB]
Irr_noise = WALTZ
Decoupling = TRUE
Initial_wait = 1[s]
Noe = TRUE
Noe_time = 2[s]
Recvr_gain = 60
Relaxation_delay = 2[s]
Repetition_time = 3.38412032[s]
Temp_get = 20.7[dC]

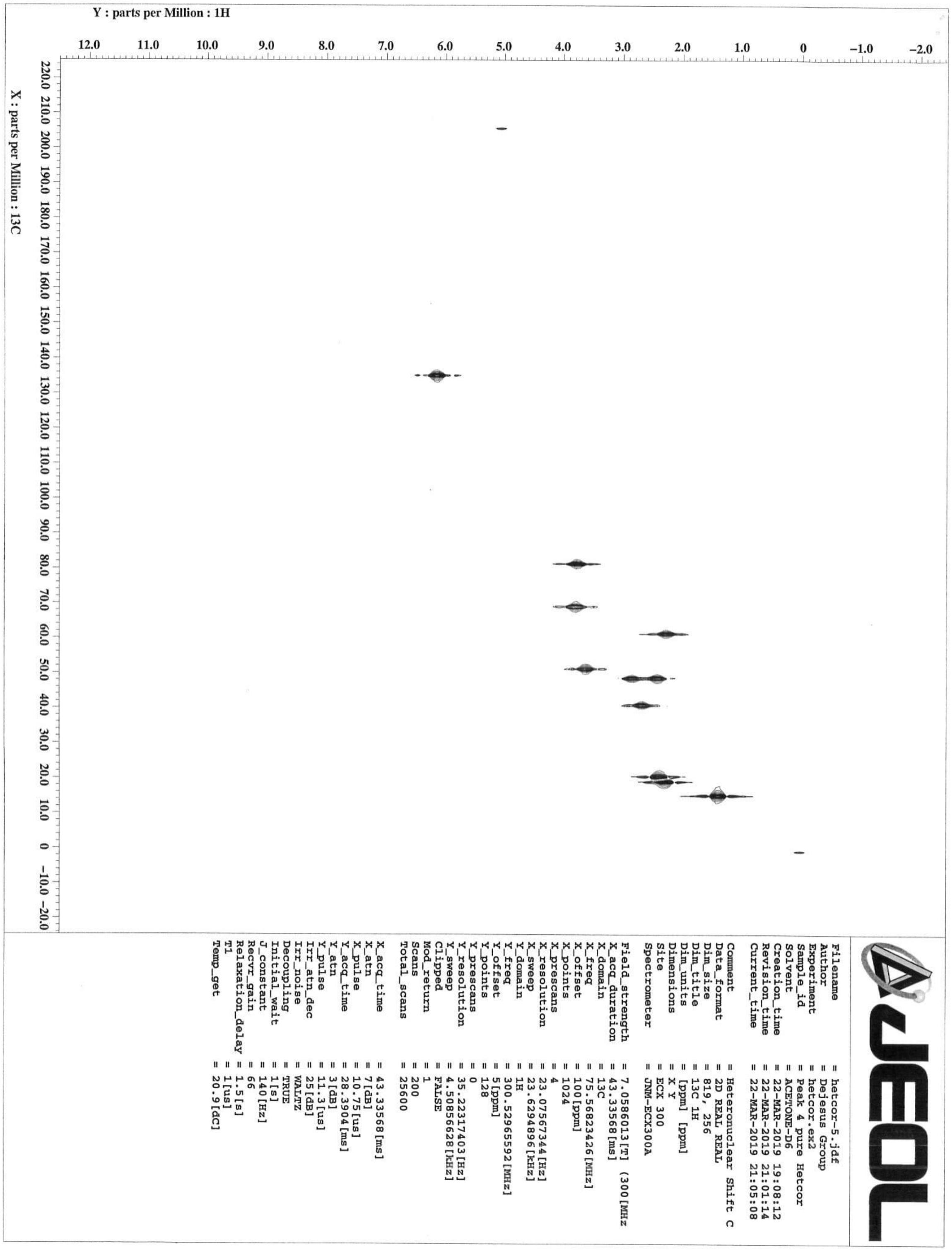
Y : parts per Million : 1H
X : parts per Million : 13C
JEOL
Filename = hetcor-5.jdf
Author = Dejesus Group
Experiment = hetcor.ex2
Sample_id = Peak 4 pure Hetcor
Solvent = ACETONE-D6
Creation_time = 22-MAR-2019 19:08:12
Revision_time = 22-MAR-2019 21:01:14
Current_time = 22-MAR-2019 21:05:08
Comment = Heteronuclear Shift C
Data_format = 2D REAL REAL
Dim_size = 819, 256
Dim_title = 13C 1H
Dim_units = [ppm] [ppm]
Dimensions = X Y
Site = ECX 300
Spectrometer = JNM-ECX300A
Field_strength = 7.0586013[T] (300[MHz
X_acq_duration = 43.33568[ms]
X_domain = 13C
X_freq = 75.56823426[MHz]
X_offset = 100[ppm]
X_points = 1024
X_prescans = 4
X_resolution = 23.07567344[Hz]
X_sweep = 23.6294896[kHz]
Y_domain = 1H
Y_freq = 300.52965592[MHz]
Y_offset = 5[ppm]
Y_points = 128
Y_prescans = 0
Y_resolution = 35.22317403[Hz]
Y_sweep = 4.50856628[kHz]
Clipped = FALSE
Mod_return = 1
Scans = 200
Total_scans = 25600
X_acq_time = 43.33568[ms]
X_atn = 7[dB]
X_pulse = 10.75[us]
Y_acq_time = 28.3904[ms]
Y_atn = 3[dB]
Y_pulse = 11.3[us]
Irr_atn_dec = 25[dB]
Irr_noise = WALTZ
Decoupling = TRUE
Initial_wait = 1[s]
J_constant = 140[Hz]
Recvr_gain = 66
Relaxation_delay = 1.5[s]
T1 = 1[us]
Temp_get = 20.9[dC]

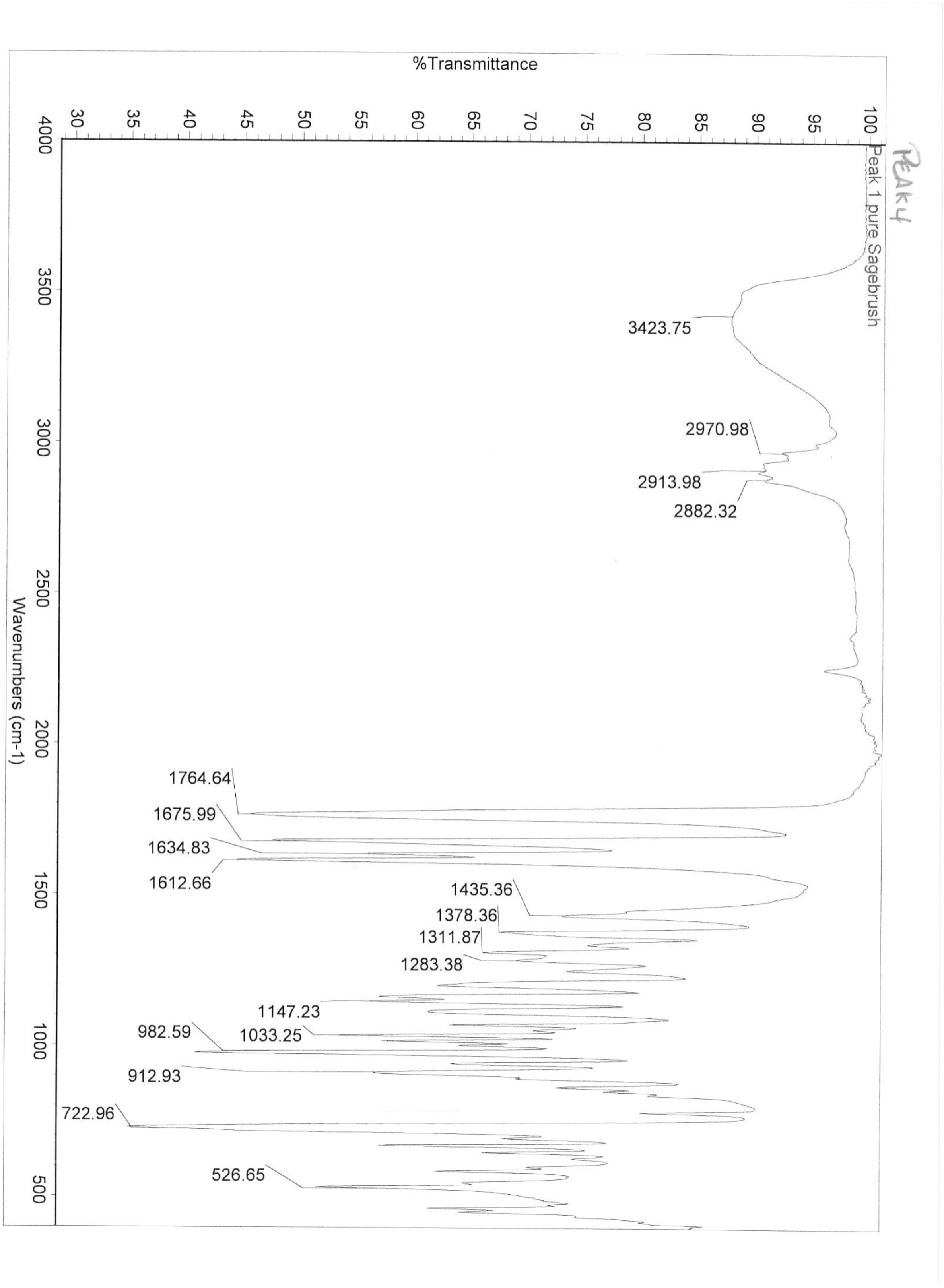

%Transmittance
Wavenumbers (cm-1)
Peak 1 pure Sagebrush
PEAK4
3423.75
2970.98
2913.98
2882.32
1764.64
1675.99
1634.83
1612.66
1435.36
1378.36
1311.87
1283.38
1147.23
982.59
1033.25
912.93
722.96
526.65

Qualitative Analysis Report

Fragmentor Voltage 180 **Collision Energy** 0 **Ionization Mode** ESI

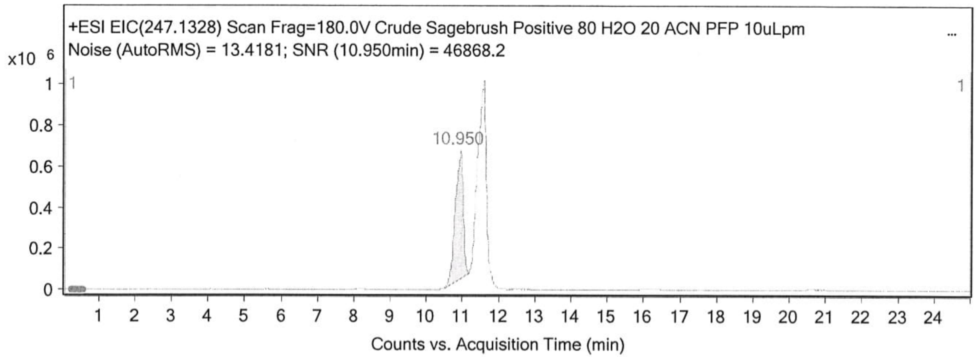

Integration Peak List

Peak	Start	RT	End	Height	Area	Area %	Signal to Noise
1	10.486	10.95	11.181	628882	9481329	100	46868.2

Noise Measurements

Noise Type	Signal Definition	Noise Multiplier	Noise Value
Auto-RMS	Height	1	13.4180994033813

Noise Regions

Start	End
.232366667	.582366667

Fragmentor Voltage 180 **Collision Energy** 0 **Ionization Mode** ESI

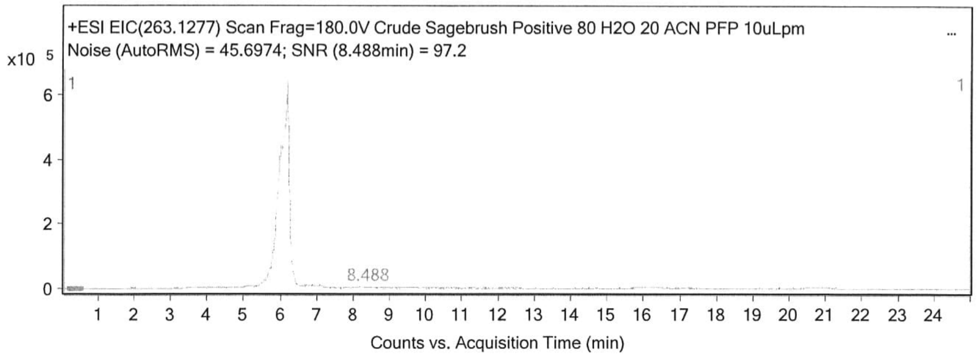

Integration Peak List

Peak	Start	RT	End	Height	Area	Area %	Signal to Noise
1	8.466	8.488	8.51	4443	3909	100	97.2

Agilent Technologies

Calculated 247.13347

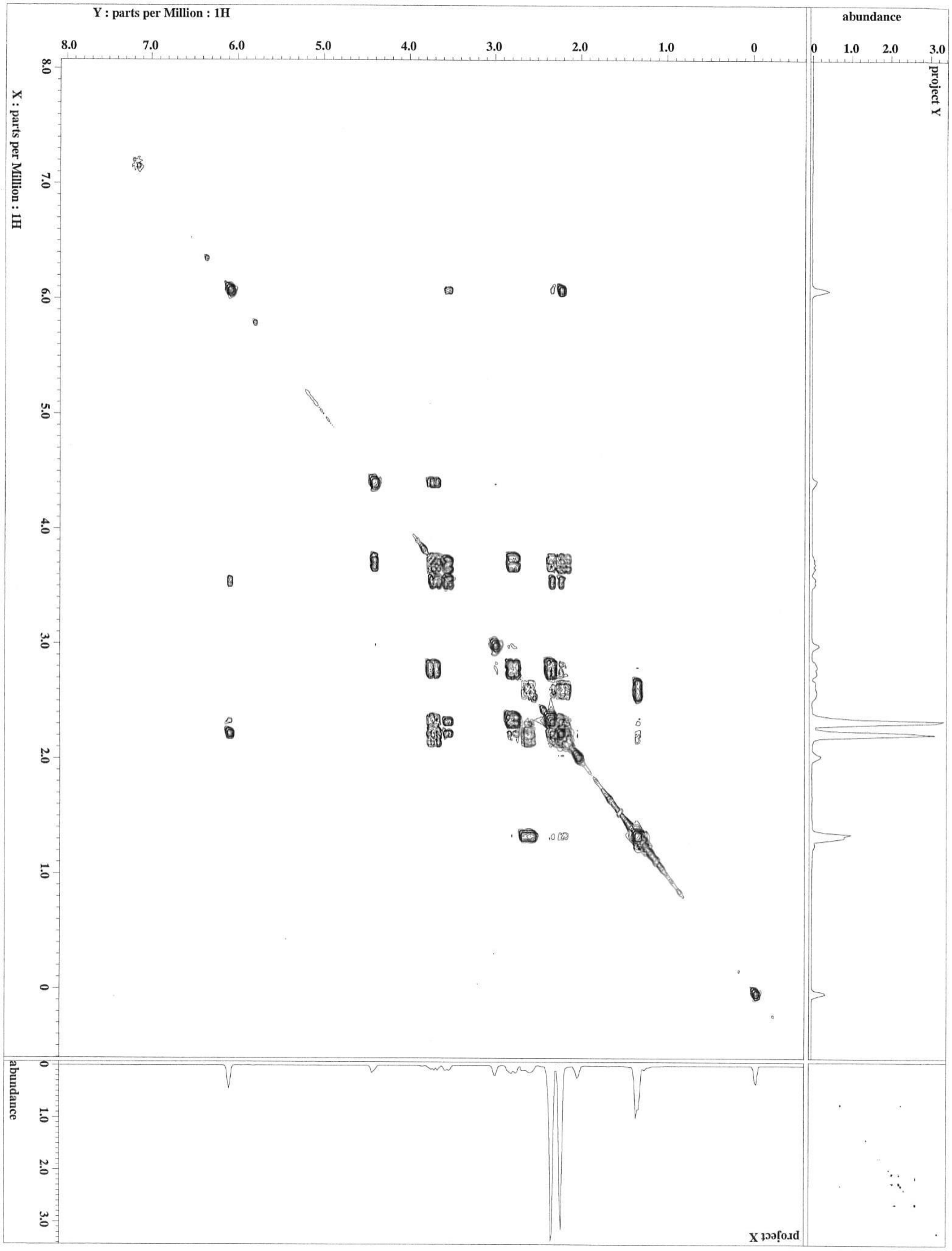
Y : parts per Million : 1H
abundance
X : parts per Million : 1H
project Y
abundance
project X

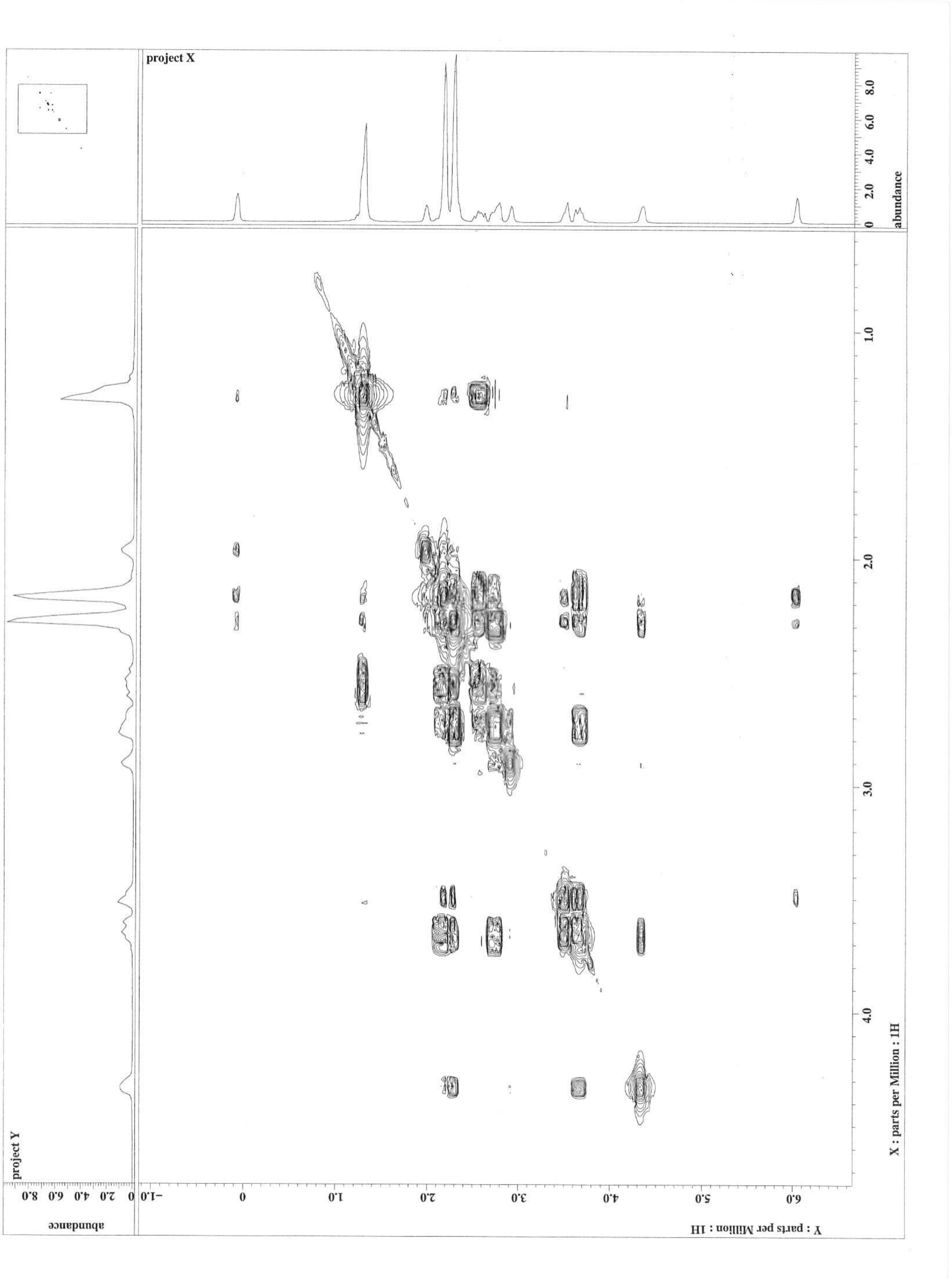
project X
abundance
project Y
abundance
X : parts per Million : 1H
Y : parts per Million : 1H

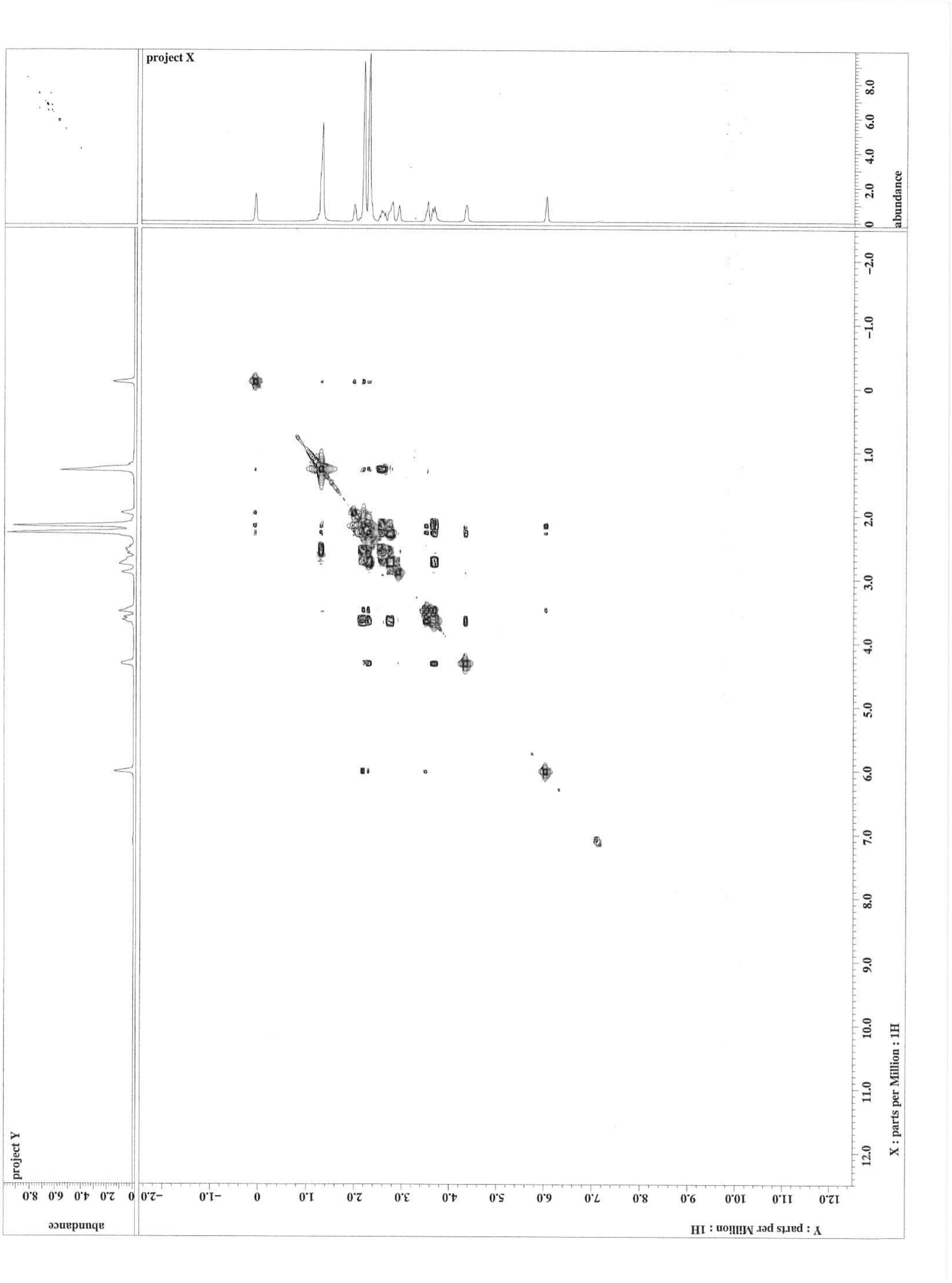
project X
abundance
X : parts per Million : 1H
project Y
abundance
Y : parts per Million : 1H
