# Supplementary material for: Sesquiterpene Lactones and Flavonoid from the Leaves of Basin Big Sagebrush (Artemisia tridentata subsp. tridentata): Isolation, Characterization and Biological Activities
Source: Molecules. 2024 Feb 9;29(4):802. doi: 10.3390/molecules29040802 (PMC10892904; doi:10.3390/molecules29040802)
Supplement: Supplementary file 1 [file molecules-29-00802-s001.zip › molecules-2800749-supplementary/Supplementary Data/Supplementary Data-4a-NMR-Scanned Files/Quercetagetin trimethyl ether NMR.pdf]

6.5 Quercetagetin Trimethyl Ether NMR Spectra

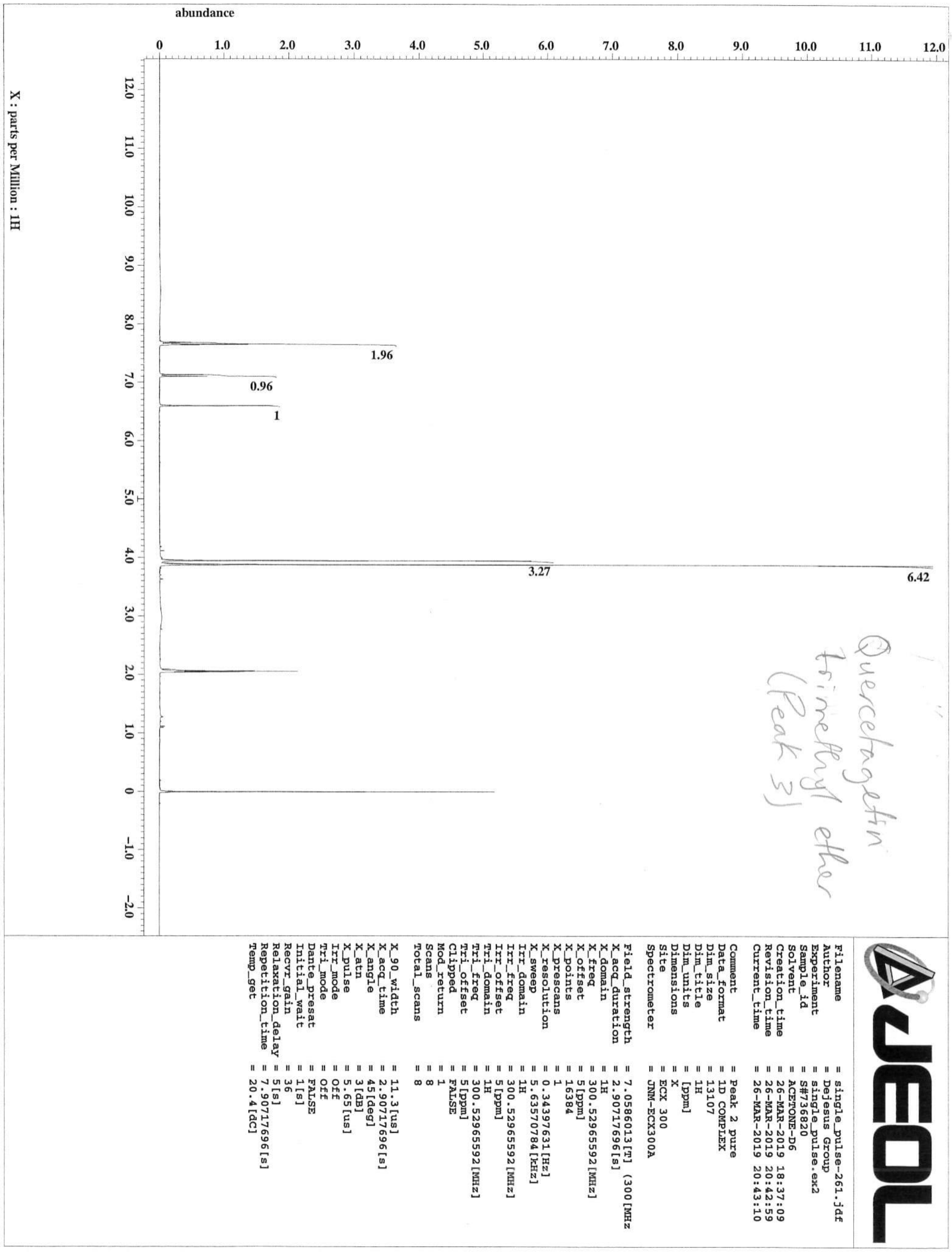

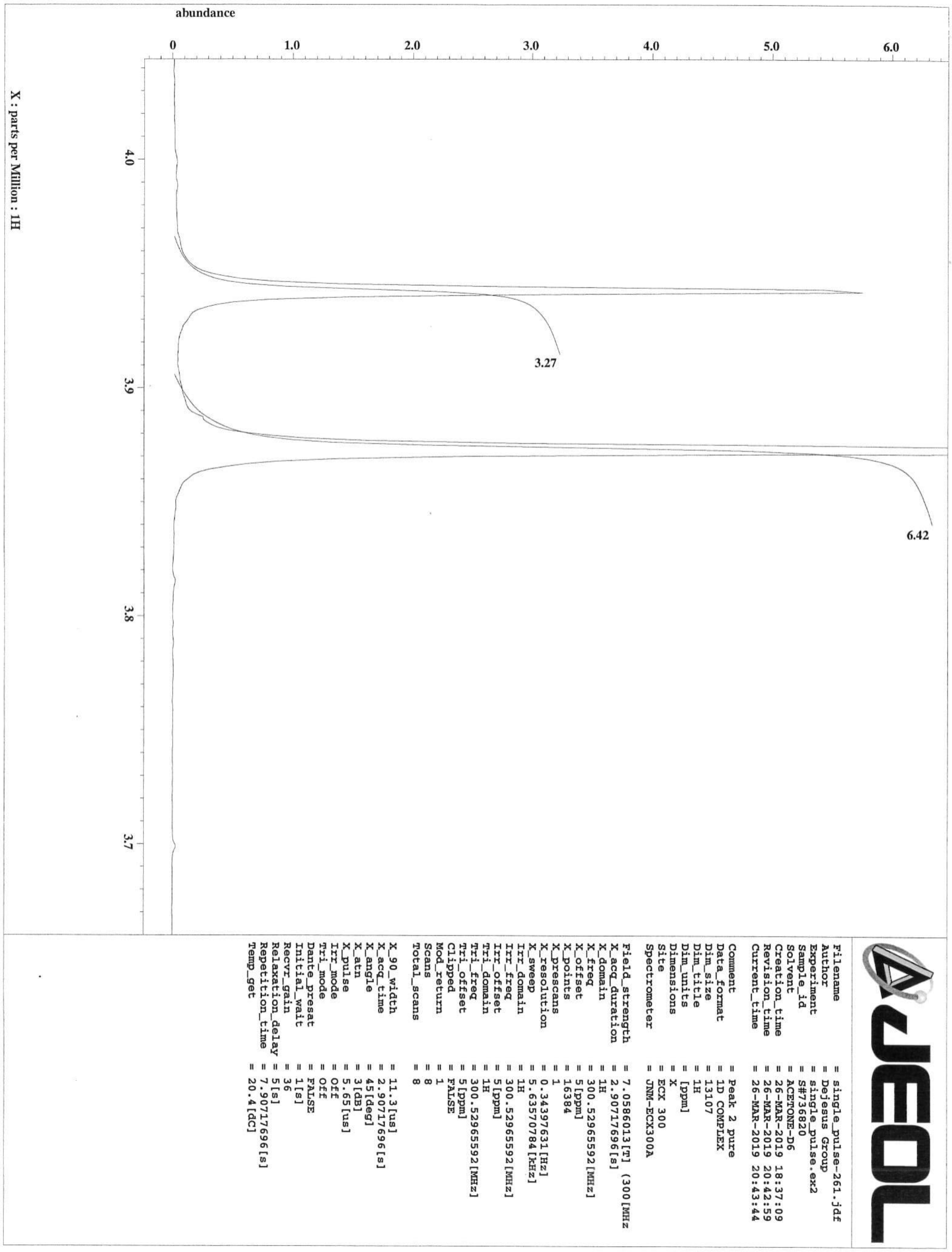
abundance
0
1.0
2.0
3.0
4.0
5.0
6.0
X : parts per Million : 1H
4.0
3.9
3.8
3.7
3.27
6.42
JEOL
Filename = single_pulse-261.jdf
Author = Dejesus Group
Experiment = single_pulse.ex2
Sample_id = s#736820
Solvent = ACETONE-D6
Creation_time = 26-MAR-2019 18:37:09
Revision_time = 26-MAR-2019 20:42:59
Current_time = 26-MAR-2019 20:43:44
Comment = Peak 2 pure
Data_format = 1D COMPLEX
Dim_size = 13107
Dim_title = 1H
Dim_units = [ppm]
Dimensions = X
Site = ECX 300
Spectrometer = JNM-ECX300A
Field_strength = 7.0586013[T] (300[MHz
X_acq_duration = 2.90717696[s]
X_domain = 1H
X_freq = 300.52965592[MHz]
X_offset = 5[ppm]
X_points = 16384
X_prescans = 1
X_resolution = 0.34397631[Hz]
X_sweep = 5.63570784[kHz]
Irr_domain = 1H
Irr_freq = 300.52965592[MHz]
Irr_offset = 5[ppm]
Tri_domain = 1H
Tri_freq = 300.52965592[MHz]
Tri_offset = 5[ppm]
Clipped = FALSE
Mod_return = 1
Scans = 8
Total_scans = 8
X_90_width = 11.3[us]
X_acq_time = 2.90717696[s]
X_angle = 45[deg]
X_atn = 3[dB]
X_pulse = 5.65[us]
Irr_mode = Off
Tri_mode = Off
Dante_presat = FALSE
Initial_wait = 1[s]
Recvr_gain = 36
Relaxation_delay = 5[s]
Repetition_time = 7.90717696[s]
Temp_get = 20.4[dC]

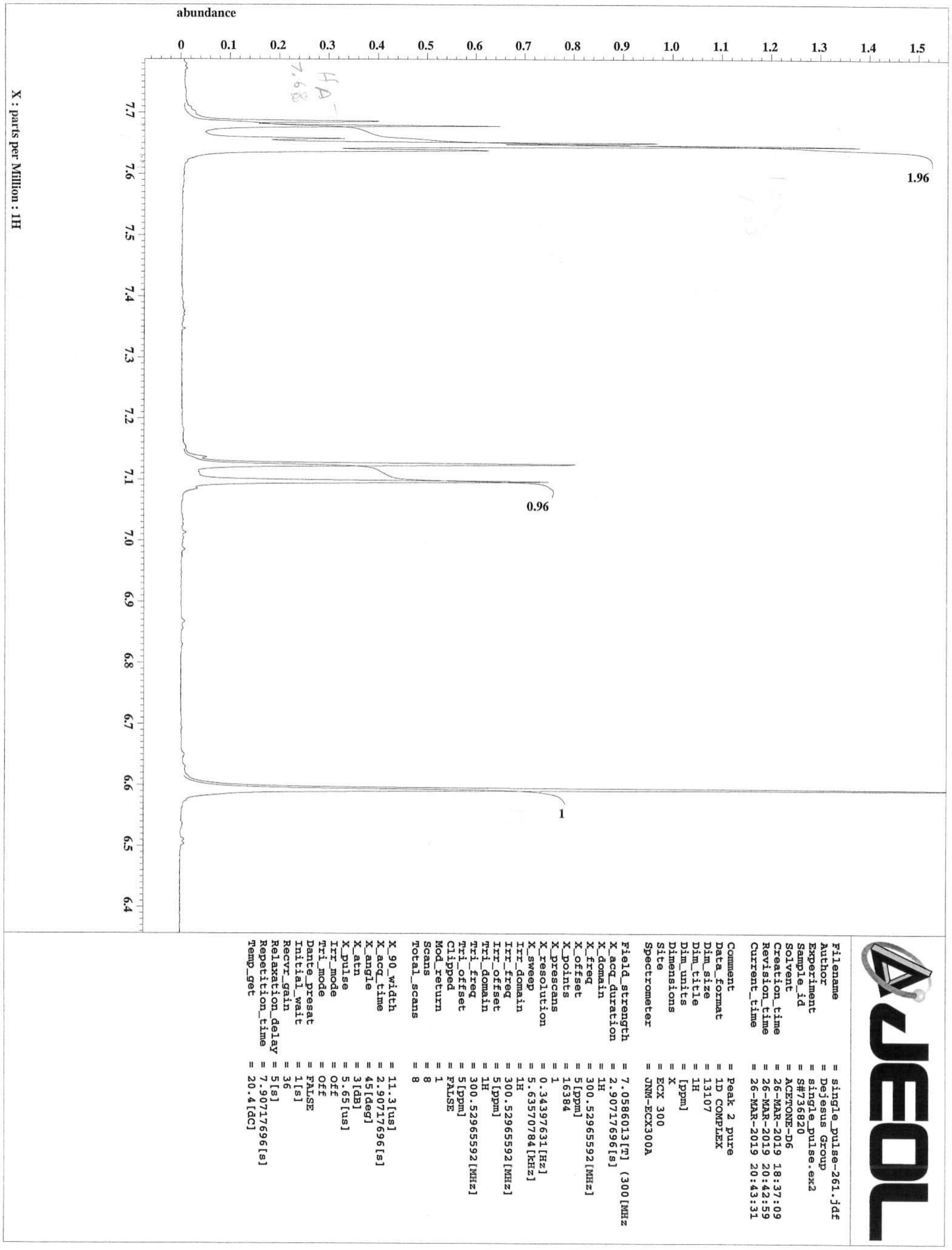
abundance
0 0.1 0.2 0.3 0.4 0.5 0.6 0.7 0.8 0.9 1.0 1.1 1.2 1.3 1.4 1.5
HA⁻
7.68
1.96
0.96
1
7.7 7.6 7.5 7.4 7.3 7.2 7.1 7.0 6.9 6.8 6.7 6.6 6.5 6.4
X : parts per Million : 1H
JEOL
Filename = single_pulse-261.jdf
Author = Dejesus Group
Experiment = single_pulse.ex2
Sample_id = S#736820
Solvent = ACETONE-D6
Creation_time = 26-MAR-2019 18:37:09
Revision_time = 26-MAR-2019 20:42:59
Current_time = 26-MAR-2019 20:43:31
Comment = Peak 2 pure
Data_format = 1D COMPLEX
Dim_size = 13107
Dim_title = 1H
Dim_units = [ppm]
Dimensions = X
Site = ECX 300
Spectrometer = JNM-ECX300A
Field_strength = 7.0586013[T] (300[MHz
X_acq_duration = 2.90717696[s]
X_domain = 1H
X_freq = 300.52965592[MHz]
X_offset = 5[ppm]
X_points = 16384
X_prescans = 1
X_resolution = 0.34397631[Hz]
X_sweep = 5.63570784[kHz]
Irr_domain = 1H
Irr_freq = 300.52965592[MHz]
Irr_offset = 5[ppm]
Tri_domain = 1H
Tri_freq = 300.52965592[MHz]
Tri_offset = 5[ppm]
Clipped = FALSE
Mod_return = 1
Scans = 8
Total_scans = 8
X_90_width = 11.3[us]
X_acq_time = 2.90717696[s]
X_angle = 45[deg]
X_atn = 3[dB]
X_pulse = 5.65[us]
Irr_mode = Off
Tri_mode = Off
Dante_presat = FALSE
Initial_wait = 1[s]
Recvr_gain = 36
Relaxation_delay = 5[s]
Repetition_time = 7.90717696[s]
Temp_get = 20.4[dC]

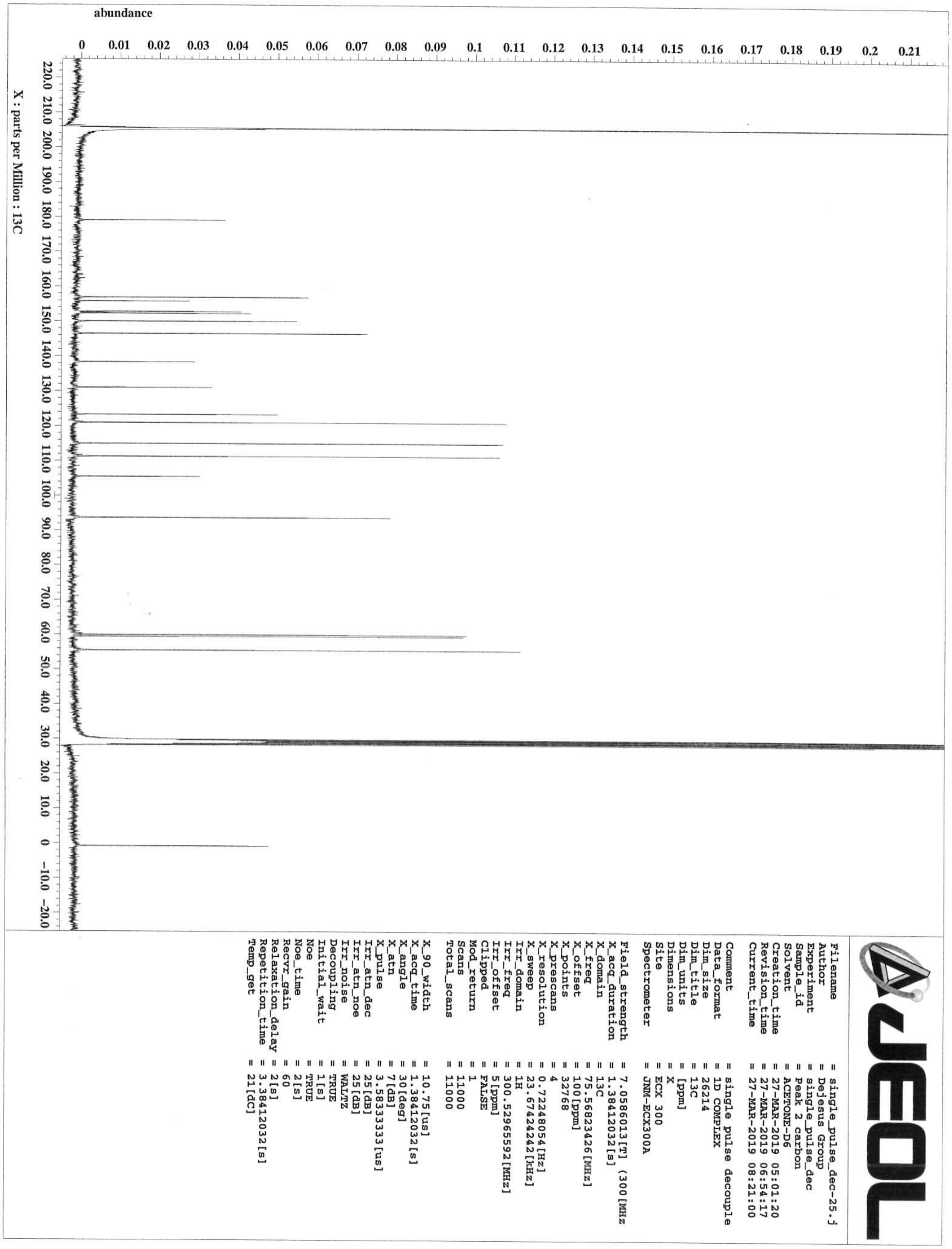
abundance
0 0.01 0.02 0.03 0.04 0.05 0.06 0.07 0.08 0.09 0.1 0.11 0.12 0.13 0.14 0.15 0.16 0.17 0.18 0.19 0.2 0.21
220.0 210.0 200.0 190.0 180.0 170.0 160.0 150.0 140.0 130.0 120.0 110.0 100.0 90.0 80.0 70.0 60.0 50.0 40.0 30.0 20.0 10.0 0 -10.0 -20.0
X : parts per Million : 13C
JEOL
Filename = single_pulse_dec-25.j
Author = Dejesus Group
Experiment = single_pulse_dec
Sample_id = Peak 2 carbon
Solvent = ACETONE-D6
Creation_time = 27-MAR-2019 05:01:20
Revision_time = 27-MAR-2019 06:54:17
Current_time = 27-MAR-2019 08:21:00
Comment = single pulse decouple
Data_format = 1D COMPLEX
Dim_size = 26214
Dim_title = 13C
Dim_units = [ppm]
Dimensions = X
Site = ECX 300
Spectrometer = JNM-ECX300A
Field_strength = 7.0586013[T] (300[MHz
X_acq_duration = 1.38412032[s]
X_domain = 13C
X_freq = 75.56823426[MHz]
X_offset = 100[ppm]
X_points = 32768
X_prescans = 4
X_resolution = 0.72248054[Hz]
X_sweep = 23.67424242[kHz]
Irr_domain = 1H
Irr_freq = 300.52965592[MHz]
Irr_offset = 5[ppm]
Clipped = FALSE
Mod_return = 1
Scans = 11000
Total_scans = 11000
X_90_width = 10.75[us]
X_acq_time = 1.38412032[s]
X_angle = 30[deg]
X_atn = 7[dB]
X_pulse = 3.58333333[us]
Irr_atn_dec = 25[dB]
Irr_atn_noe = 25[dB]
Irr_noise = WALTZ
Decoupling = TRUE
Initial_wait = 1[s]
Noe = TRUE
Noe_time = 2[s]
Recvr_gain = 60
Relaxation_delay = 2[s]
Repetition_time = 3.38412032[s]
Temp_get = 21[dC]

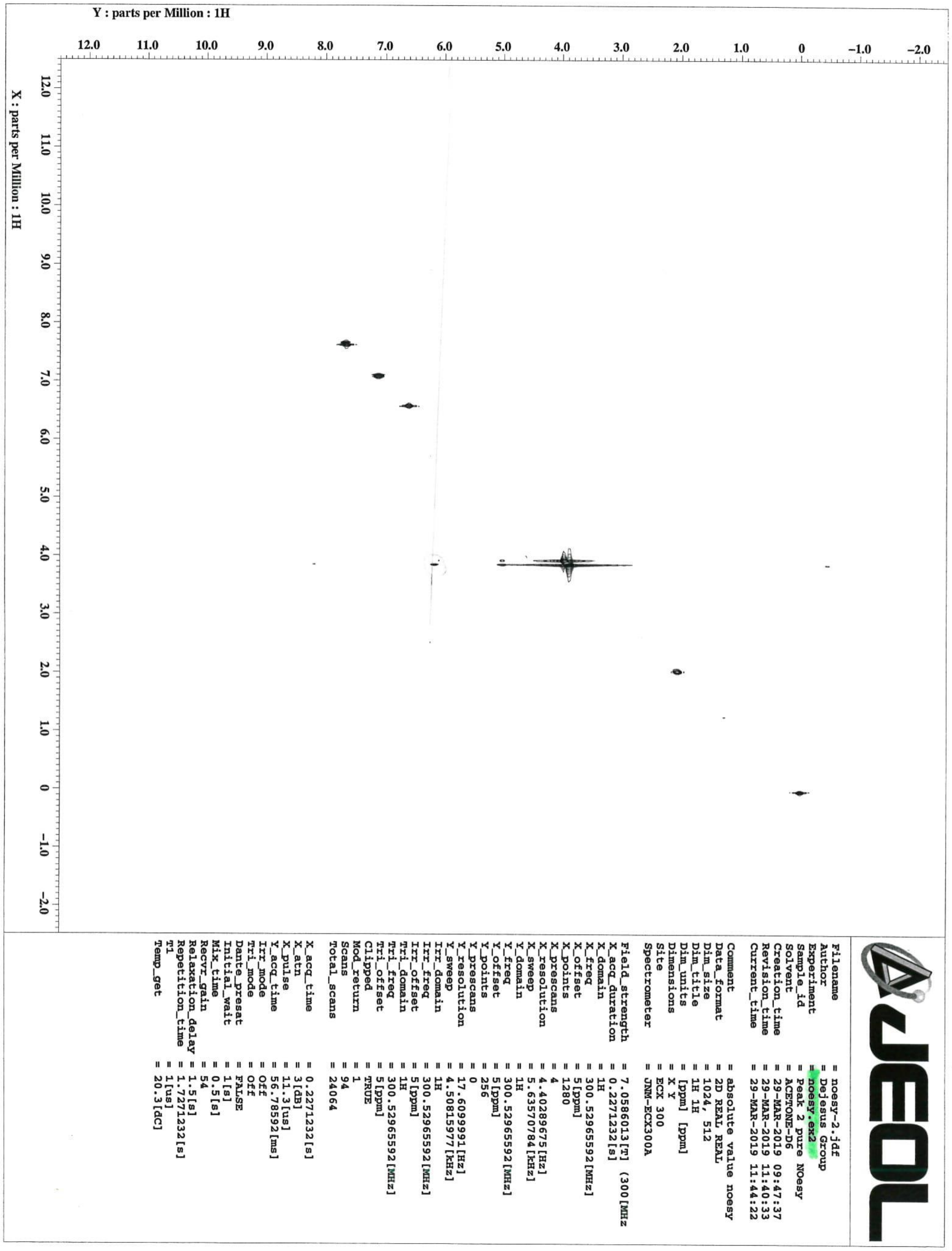

Y : parts per Million : 1H
X : parts per Million : 1H
12.0
11.0
10.0
9.0
8.0
7.0
6.0
5.0
4.0
3.0
2.0
1.0
0
−1.0
−2.0
Filename = noesy-2.jdf
Author = Dejesus Group
Experiment = noesy.ex2
Sample_id = Peak 2 pure NOesy
Solvent = ACETONE-D6
Creation_time = 29-MAR-2019 09:47:37
Revision_time = 29-MAR-2019 11:40:33
Current_time = 29-MAR-2019 11:44:22
Comment = absolute value noesy
Data_format = 2D REAL REAL
Dim_size = 1024, 512
Dim_title = 1H 1H
Dim_units = [ppm] [ppm]
Dimensions = X Y
Site = ECX 300
Spectrometer = JNM-ECX300A
Field_strength = 7.0586013[T] (300[MHz
X_acq_duration = 0.2271232[s]
X_domain = 1H
X_freq = 300.52965592[MHz]
X_offset = 5[ppm]
X_points = 1280
X_prescans = 4
X_resolution = 4.40289675[Hz]
X_sweep = 5.63570784[kHz]
Y_domain = 1H
Y_freq = 300.52965592[MHz]
Y_offset = 5[ppm]
Y_points = 256
Y_prescans = 0
Y_resolution = 17.6099991[Hz]
Y_sweep = 4.50815977[kHz]
Irr_domain = 1H
Irr_freq = 300.52965592[MHz]
Irr_offset = 5[ppm]
Tri_domain = 1H
Tri_freq = 300.52965592[MHz]
Tri_offset = 5[ppm]
Clipped = TRUE
Mod_return = 1
Scans = 94
Total_scans = 24064
X_acq_time = 0.2271232[s]
X_atn = 3[dB]
X_pulse = 11.3[us]
Y_acq_time = 56.78592[ms]
Irr_mode = Off
Tri_mode = Off
Dante_presat = FALSE
Initial_wait = 1[s]
Mix_time = 0.5[s]
Recvr_gain = 54
Relaxation_delay = 1.5[s]
Repetition_time = 1.7271232[s]
T1 = 1[us]
Temp_get = 20.3[dC]

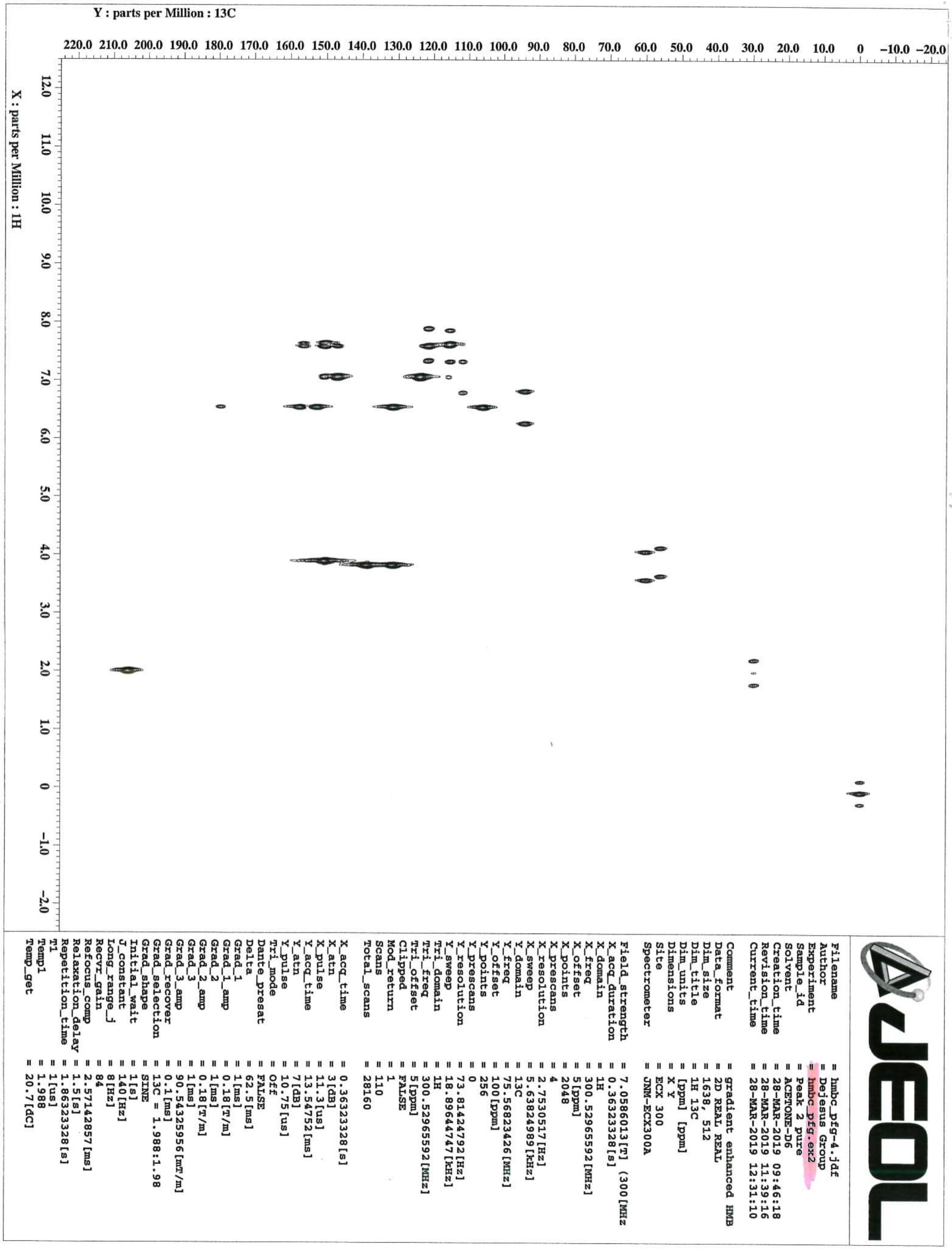
Y : parts per Million : 13C
220.0 210.0 200.0 190.0 180.0 170.0 160.0 150.0 140.0 130.0 120.0 110.0 100.0 90.0 80.0 70.0 60.0 50.0 40.0 30.0 20.0 10.0 0 −10.0 −20.0
X : parts per Million : 1H
12.0 11.0 10.0 9.0 8.0 7.0 6.0 5.0 4.0 3.0 2.0 1.0 0 −1.0 −2.0
JEOL
Filename = hmbc_pfg-4.jdf
Author = Dejesus Group
Experiment = hmbc_pfg.ex2
Sample_id = Peak 2 pure
Solvent = ACETONE-D6
Creation_time = 28-MAR-2019 09:46:18
Revision_time = 28-MAR-2019 11:39:16
Current_time = 28-MAR-2019 12:31:10
Comment = gradient enhanced HMB
Data_format = 2D REAL REAL
Dim_size = 1638, 512
Dim_title = 1H 13C
Dim_units = [ppm] [ppm]
Dimensions = X Y
Site = ECX 300
Spectrometer = JNM-ECX300A
Field_strength = 7.0586013[T] (300[MHz
X_acq_duration = 0.36323328[s]
X_domain = 1H
X_freq = 300.52965592[MHz]
X_offset = 5[ppm]
X_points = 2048
X_prescans = 4
X_resolution = 2.7530517[Hz]
X_sweep = 5.63824989[kHz]
Y_domain = 13C
Y_freq = 75.56823426[MHz]
Y_offset = 100[ppm]
Y_points = 256
Y_prescans = 0
Y_resolution = 73.81424792[Hz]
Y_sweep = 18.89644747[kHz]
Tri_domain = 1H
Tri_freq = 300.52965592[MHz]
Tri_offset = 5[ppm]
Clipped = FALSE
Mod_return = 1
Scans = 110
Total_scans = 28160
X_acq_time = 0.36323328[s]
X_atn = 3[dB]
X_pulse = 11.3[us]
Y_acq_time = 13.54752[ms]
Y_atn = 7[dB]
Y_pulse = 10.75[us]
Tri_mode = Off
Dante_presat = FALSE
Delta = 62.5[ms]
Grad_1 = 1[ms]
Grad_1_amp = 0.18[T/m]
Grad_2 = 1[ms]
Grad_2_amp = 0.18[T/m]
Grad_3 = 1[ms]
Grad_3_amp = 90.54325956[mT/m]
Grad_recover = 0.1[ms]
Grad_selection = 13C = 1.988:1.98
Grad_shape = SINE
Initial_wait = 1[s]
J_constant = 140[Hz]
Long_range_j = 8[Hz]
Recvr_gain = 84
Refocus_comp = 2.57142857[ms]
Relaxation_delay = 1.5[s]
Repetition_time = 1.86323328[s]
T1 = 1[us]
Temp1 = 1.988
Temp_get = 20.7[dC]

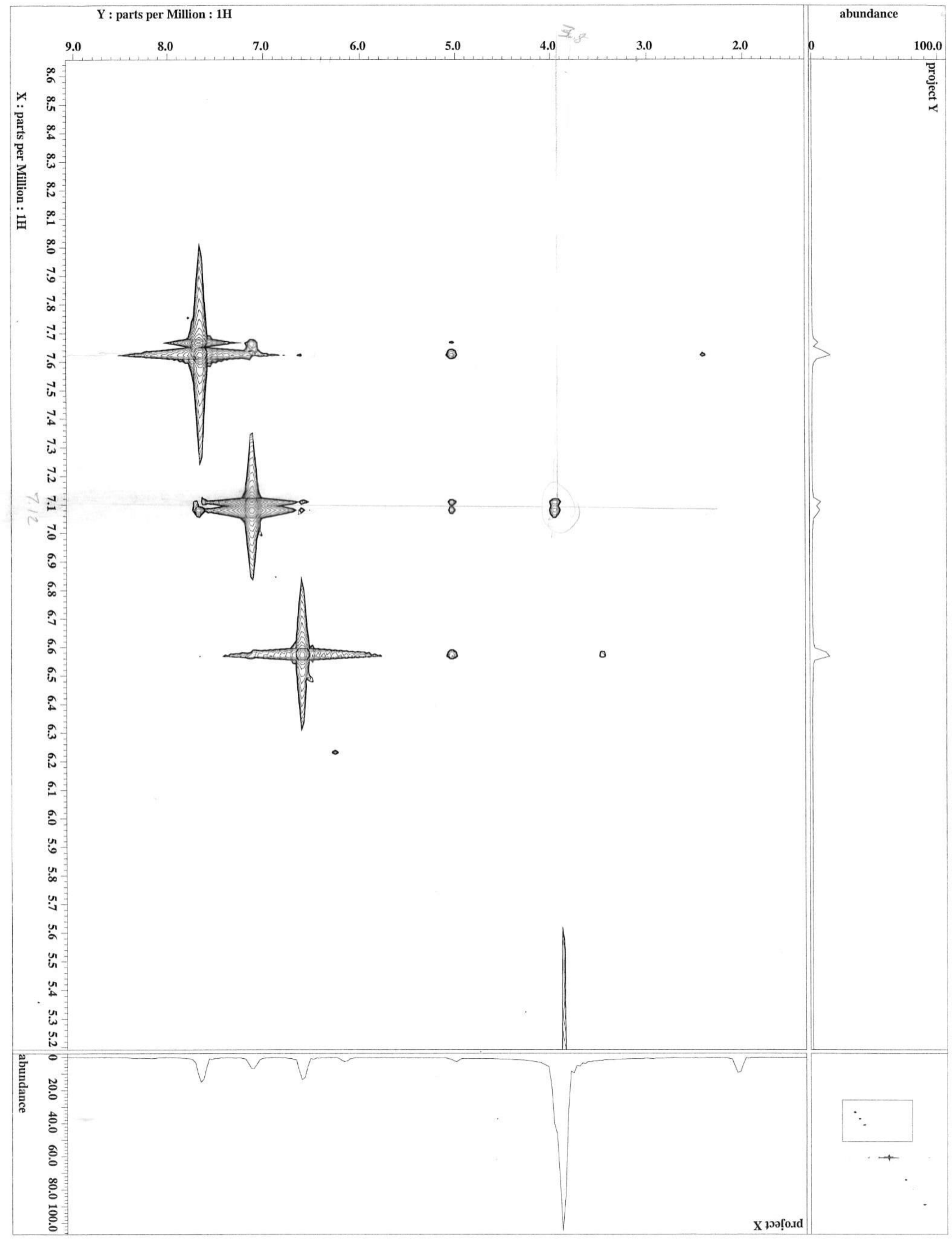
Y : parts per Million : 1H
abundance
X : parts per Million : 1H
project Y
project X

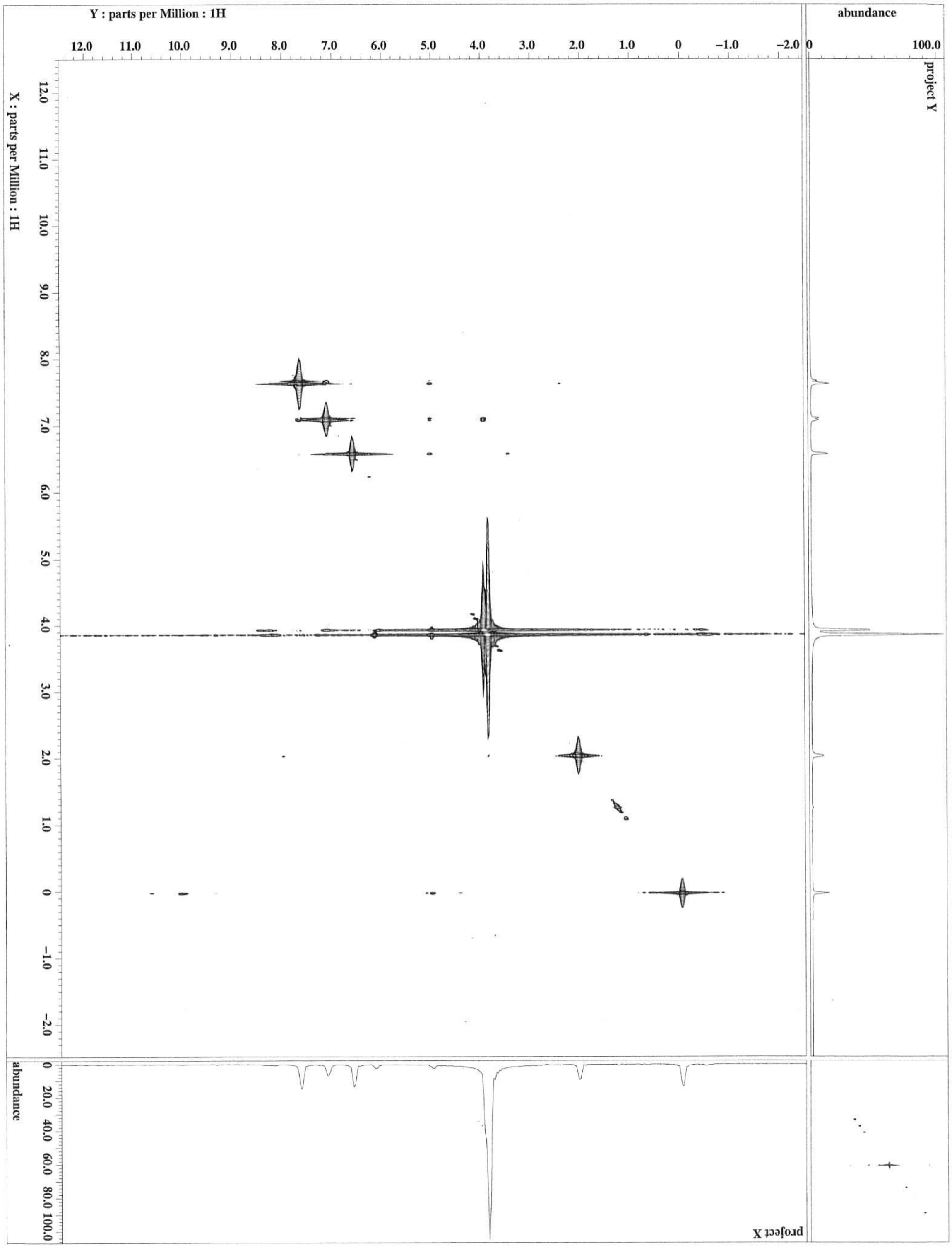
Y : parts per Million : 1H
abundance
12.0 11.0 10.0 9.0 8.0 7.0 6.0 5.0 4.0 3.0 2.0 1.0 0 −1.0 −2.0
0 100.0
project Y
X : parts per Million : 1H
abundance
0 20.0 40.0 60.0 80.0 100.0
project X

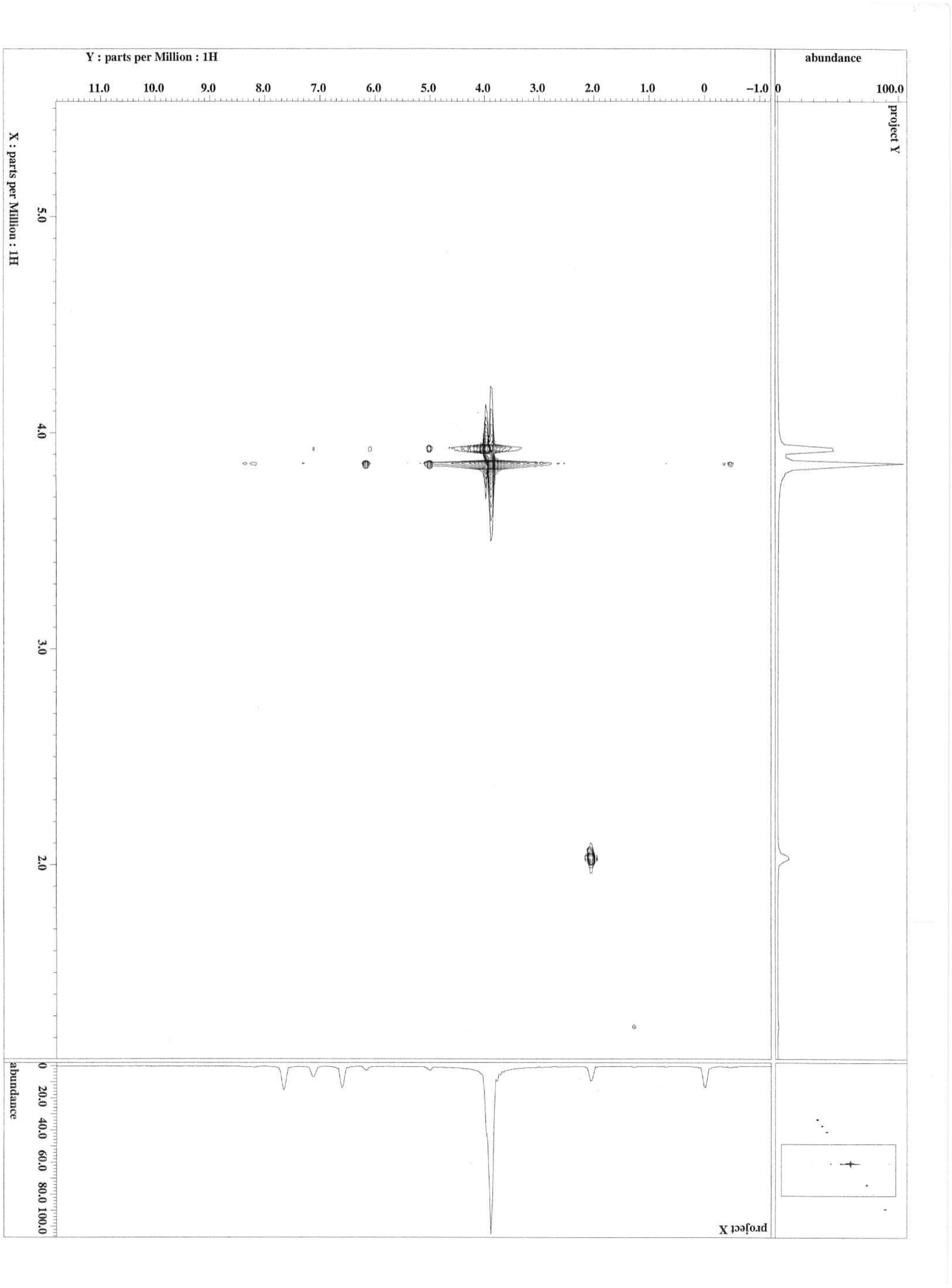
Y : parts per Million : 1H
11.0 10.0 9.0 8.0 7.0 6.0 5.0 4.0 3.0 2.0 1.0 0 −1.0
abundance
0 100.0
project Y
X : parts per Million : 1H
5.0
4.0
3.0
2.0
abundance
0 20.0 40.0 60.0 80.0 100.0
project X

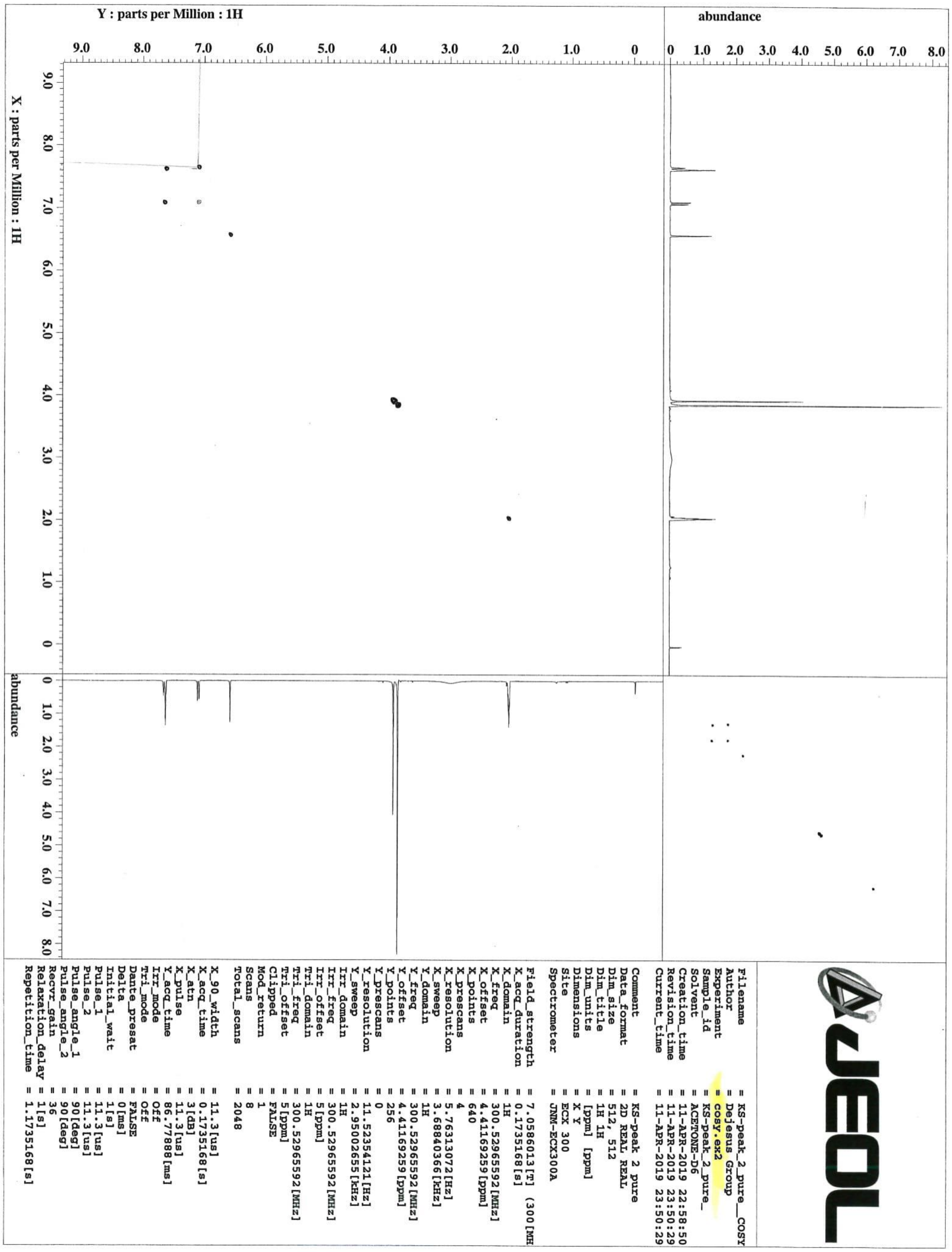
Y : parts per Million : 1H
X : parts per Million : 1H
abundance
abundance
JEOL
Filename = KS-peak_2_pure__COSY
Author = Dejesus Group
Experiment = cosy.ex2
Sample_id = KS-peak_2_pure_
Solvent = ACETONE-D6
Creation_time = 11-APR-2019 22:58:50
Revision_time = 11-APR-2019 23:50:29
Current_time = 11-APR-2019 23:50:29
Comment = KS-peak 2 pure
Data_format = 2D REAL REAL
Dim_size = 512, 512
Dim_title = 1H 1H
Dim_units = [ppm] [ppm]
Dimensions = X Y
Site = ECX 300
Spectrometer = JNM-ECX300A
Field_strength = 7.0586013[T] (300[MH
X_acq_duration = 0.17351168[s]
X_domain = 1H
X_freq = 300.52965592[MHz]
X_offset = 4.41169259[ppm]
X_points = 640
X_prescans = 4
X_resolution = 5.76313072[Hz]
X_sweep = 3.68840366[kHz]
Y_domain = 1H
Y_freq = 300.52965592[MHz]
Y_offset = 4.41169259[ppm]
Y_points = 256
Y_prescans = 0
Y_resolution = 11.52354121[Hz]
Y_sweep = 2.95002655[kHz]
Irr_domain = 1H
Irr_freq = 300.52965592[MHz]
Irr_offset = 5[ppm]
Tri_domain = 1H
Tri_freq = 300.52965592[MHz]
Tri_offset = 5[ppm]
Clipped = FALSE
Mod_return = 1
Scans = 8
Total_scans = 2048
X_90_width = 11.3[us]
X_acq_time = 0.17351168[s]
X_atn = 3[dB]
X_pulse = 11.3[us]
Y_acq_time = 86.77888[ms]
Irr_mode = Off
Tri_mode = Off
Dante_presat = FALSE
Delta = 0[ms]
Initial_wait = 1[s]
Pulse_1 = 11.3[us]
Pulse_2 = 11.3[us]
Pulse_angle_1 = 90[deg]
Pulse_angle_2 = 90[deg]
Recvr_gain = 36
Relaxation_delay = 1[s]
Repetition_time = 1.17351168[s]

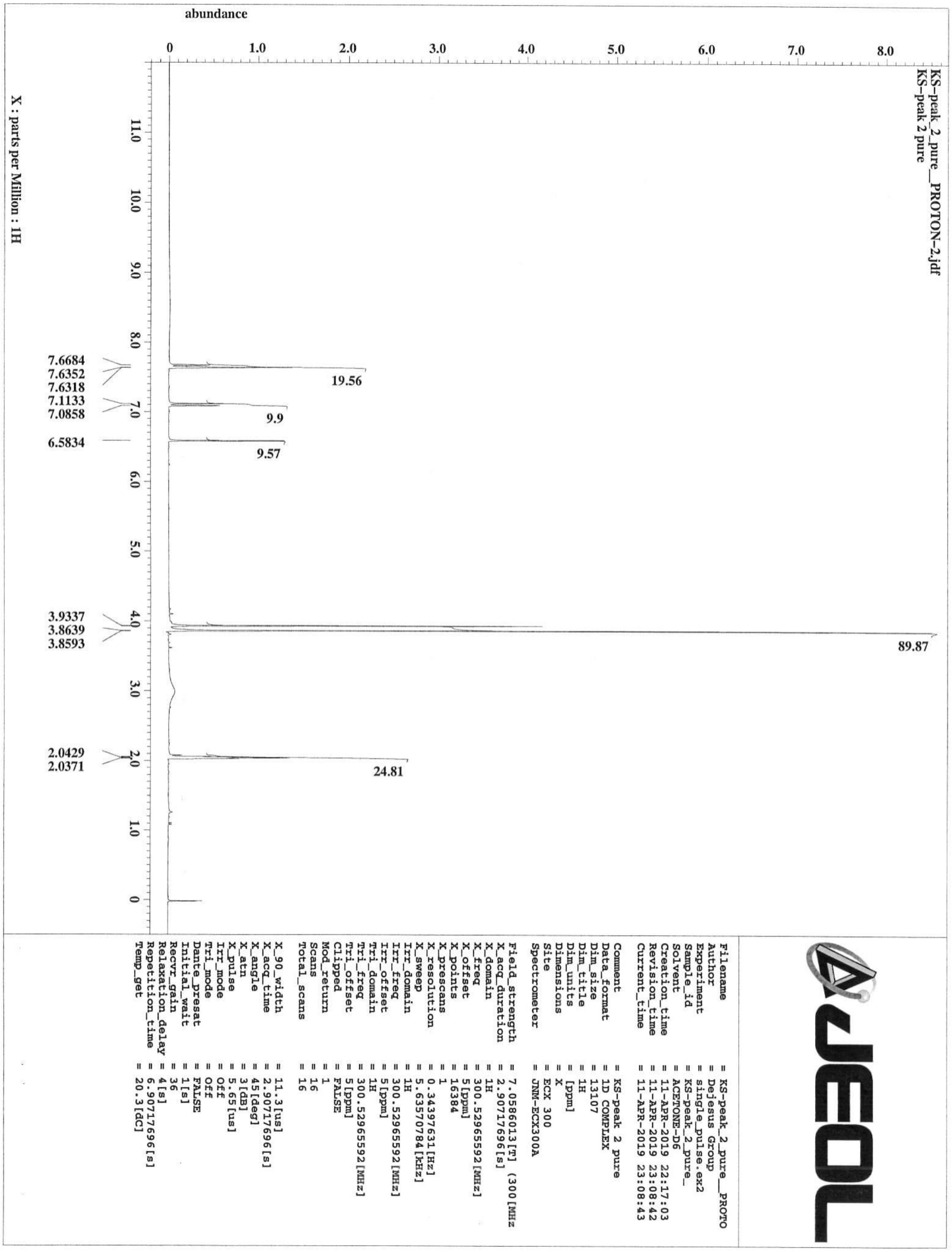
abundance
0
1.0
2.0
3.0
4.0
5.0
6.0
7.0
8.0
KS−peak_2_pure__PROTON−2.jdf
KS−peak 2 pure
X : parts per Million : 1H
11.0
10.0
9.0
8.0
7.0
6.0
5.0
4.0
3.0
2.0
1.0
0
7.6684
7.6352
7.6318
7.1133
7.0858
6.5834
3.9337
3.8639
3.8593
2.0429
2.0371
19.56
9.9
9.57
89.87
24.81
Filename = KS-peak_2_pure__PROTO
Author = Dejesus Group
Experiment = single_pulse.ex2
Sample_id = KS-peak_2_pure_
Solvent = ACETONE-D6
Creation_time = 11-APR-2019 22:17:03
Revision_time = 11-APR-2019 23:08:42
Current_time = 11-APR-2019 23:08:43
Comment = KS-peak 2 pure
Data_format = 1D COMPLEX
Dim_size = 13107
Dim_title = 1H
Dim_units = [ppm]
Dimensions = X
Site = ECX 300
Spectrometer = JNM-ECX300A
Field_strength = 7.0586013[T] (300[MHz
X_acq_duration = 2.90717696[s]
X_domain = 1H
X_freq = 300.52965592[MHz]
X_offset = 5[ppm]
X_points = 16384
X_prescans = 1
X_resolution = 0.34397631[Hz]
X_sweep = 5.63570784[kHz]
Irr_domain = 1H
Irr_freq = 300.52965592[MHz]
Irr_offset = 5[ppm]
Tri_domain = 1H
Tri_freq = 300.52965592[MHz]
Tri_offset = 5[ppm]
Clipped = FALSE
Mod_return = 1
Scans = 16
Total_scans = 16
X_90_width = 11.3[us]
X_acq_time = 2.90717696[s]
X_angle = 45[deg]
X_atn = 3[dB]
X_pulse = 5.65[us]
Irr_mode = Off
Tri_mode = Off
Dante_presat = FALSE
Initial_wait = 1[s]
Recvr_gain = 36
Relaxation_delay = 4[s]
Repetition_time = 6.90717696[s]
Temp_get = 20.3[dC]
JEOL

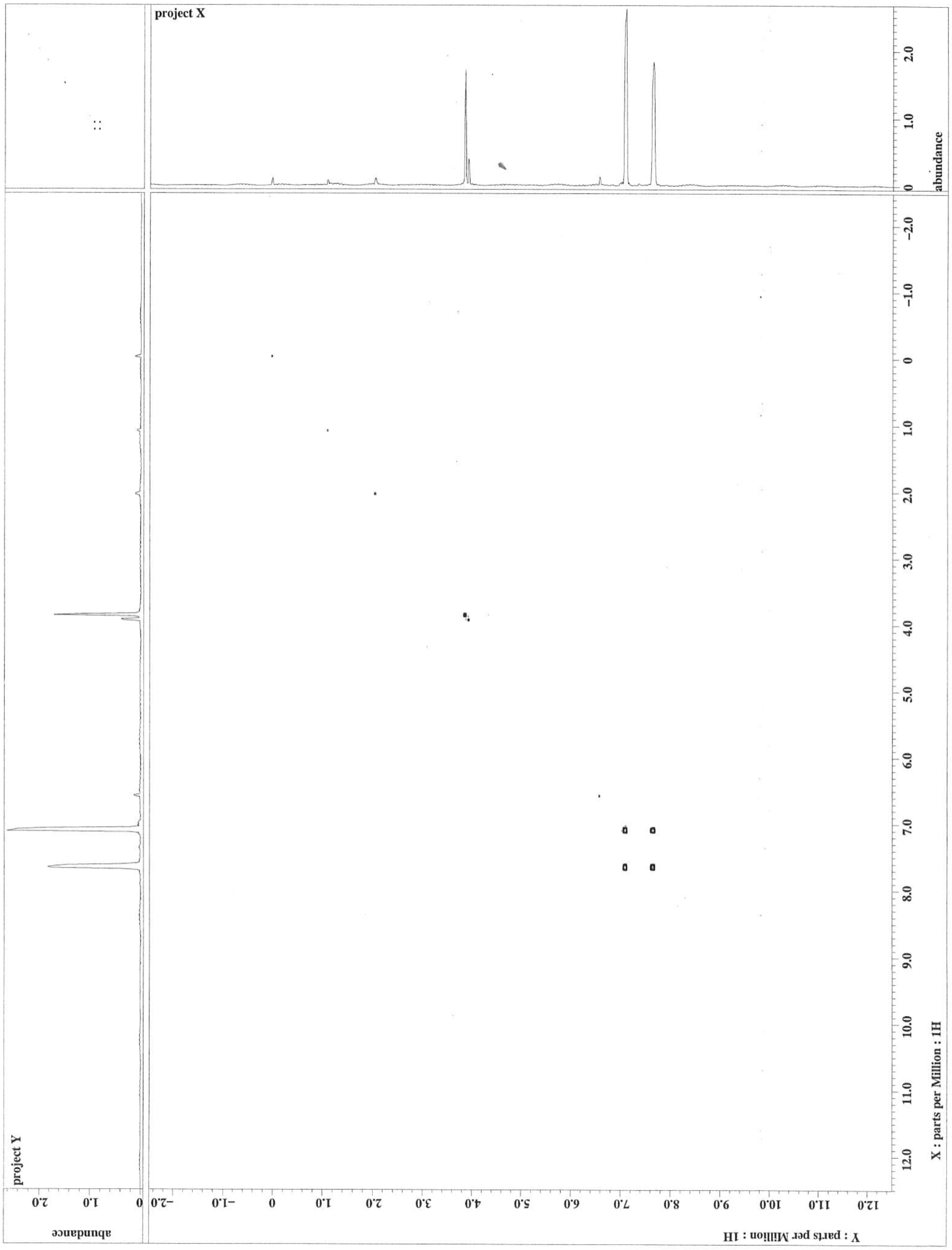
project X
abundance
project Y
abundance
X : parts per Million : 1H
Y : parts per Million : 1H

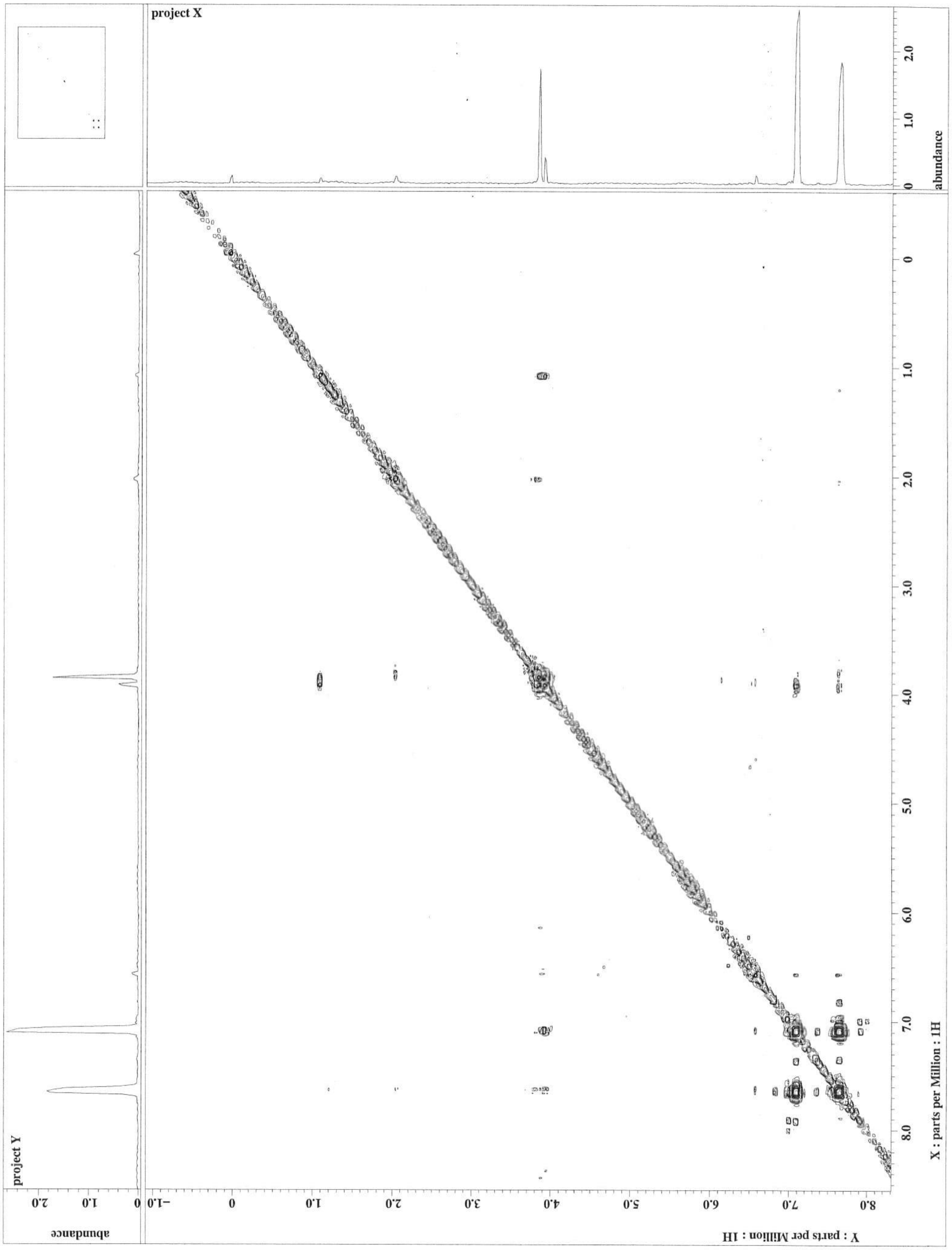
project X
abundance
project Y
abundance
X : parts per Million : 1H
Y : parts per Million : 1H

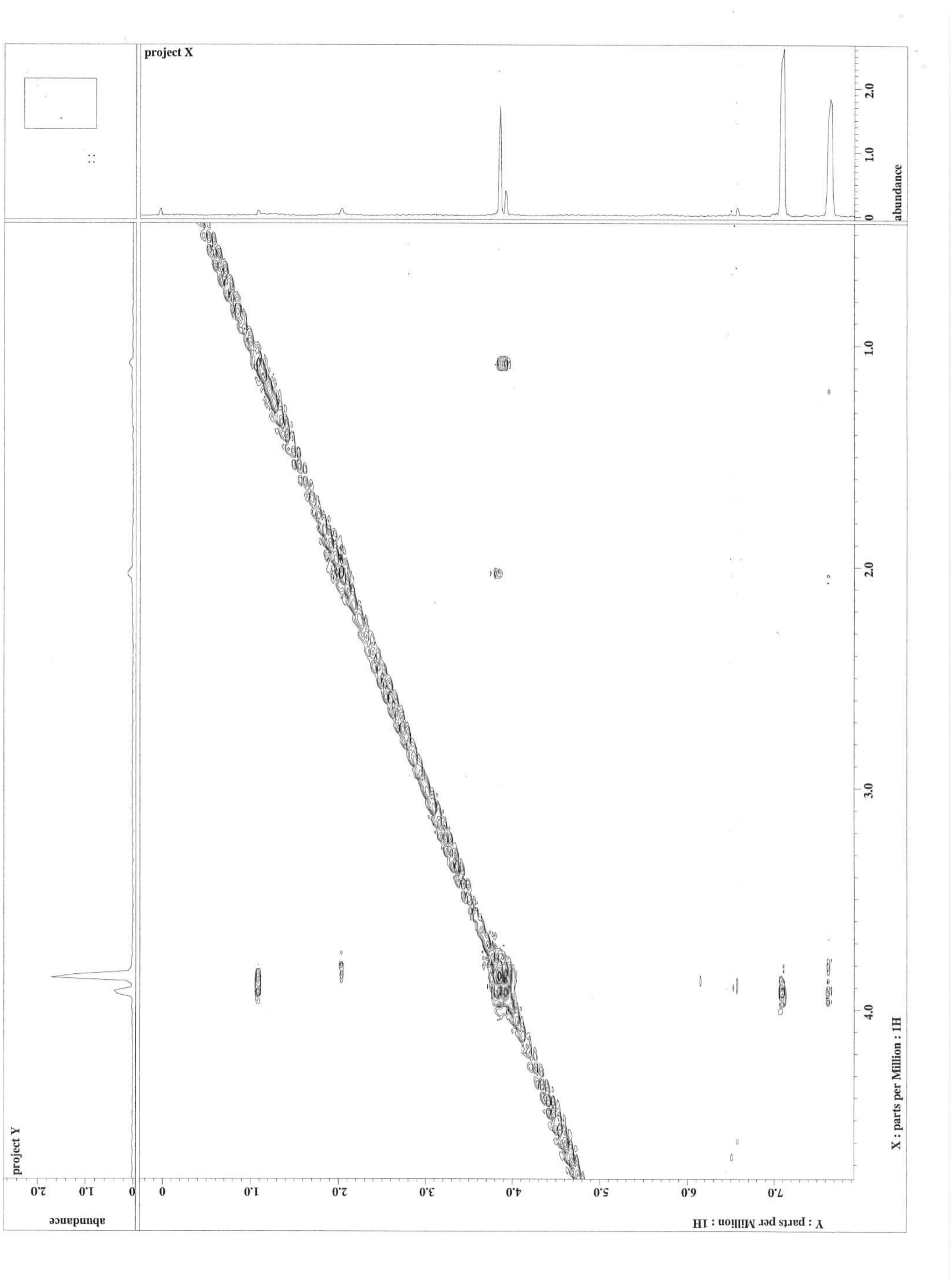
project X
abundance
X : parts per Million : 1H
project Y
abundance
Y : parts per Million : 1H

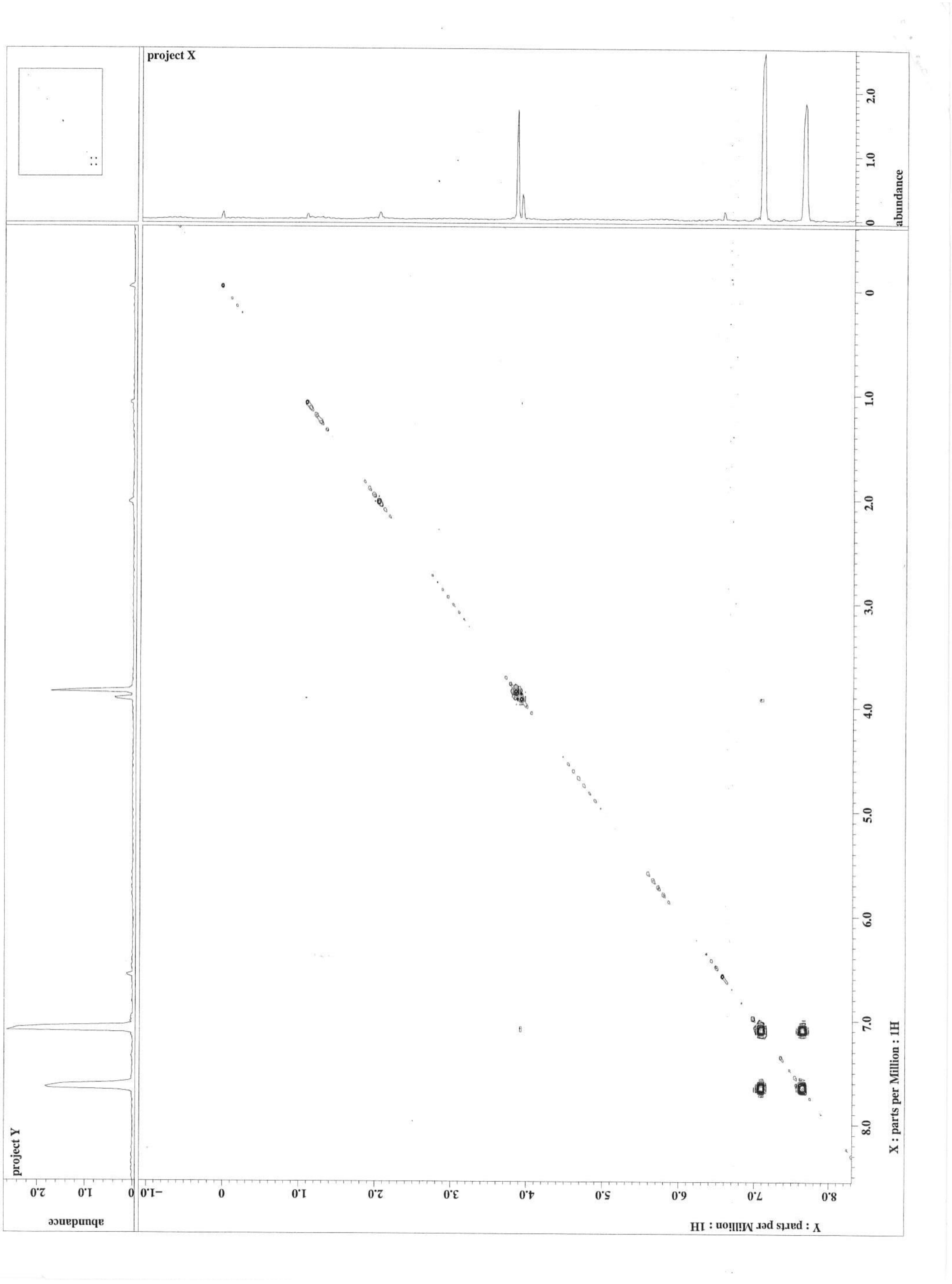
project X
project Y
abundance
abundance
X : parts per Million : 1H
Y : parts per Million : 1H

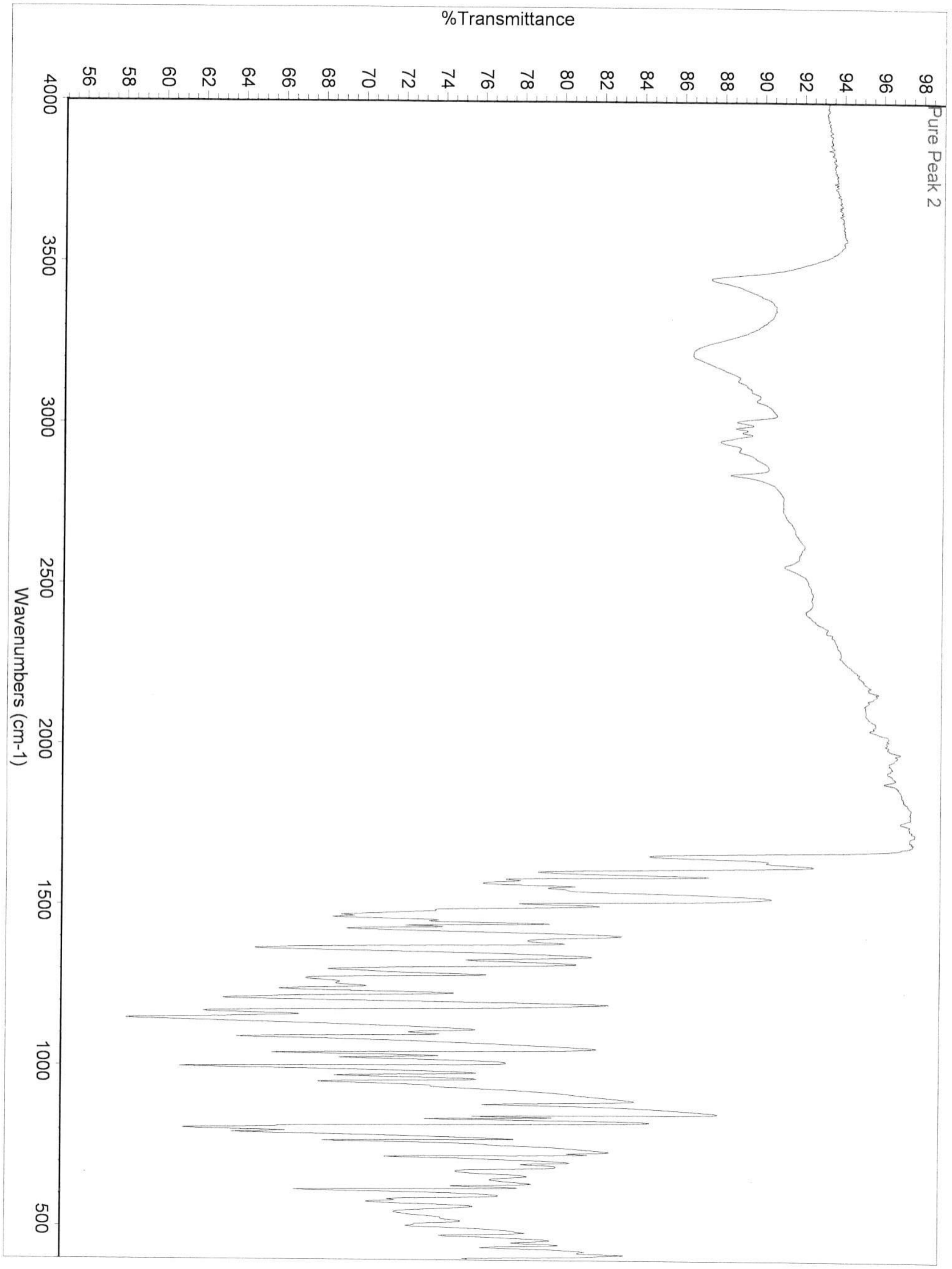
%Transmittance
Pure Peak 2
Wavenumbers (cm-1)
4000
3500
3000
2500
2000
1500
1000
500
56
58
60
62
64
66
68
70
72
74
76
78
80
82
84
86
88
90
92
94
96
98

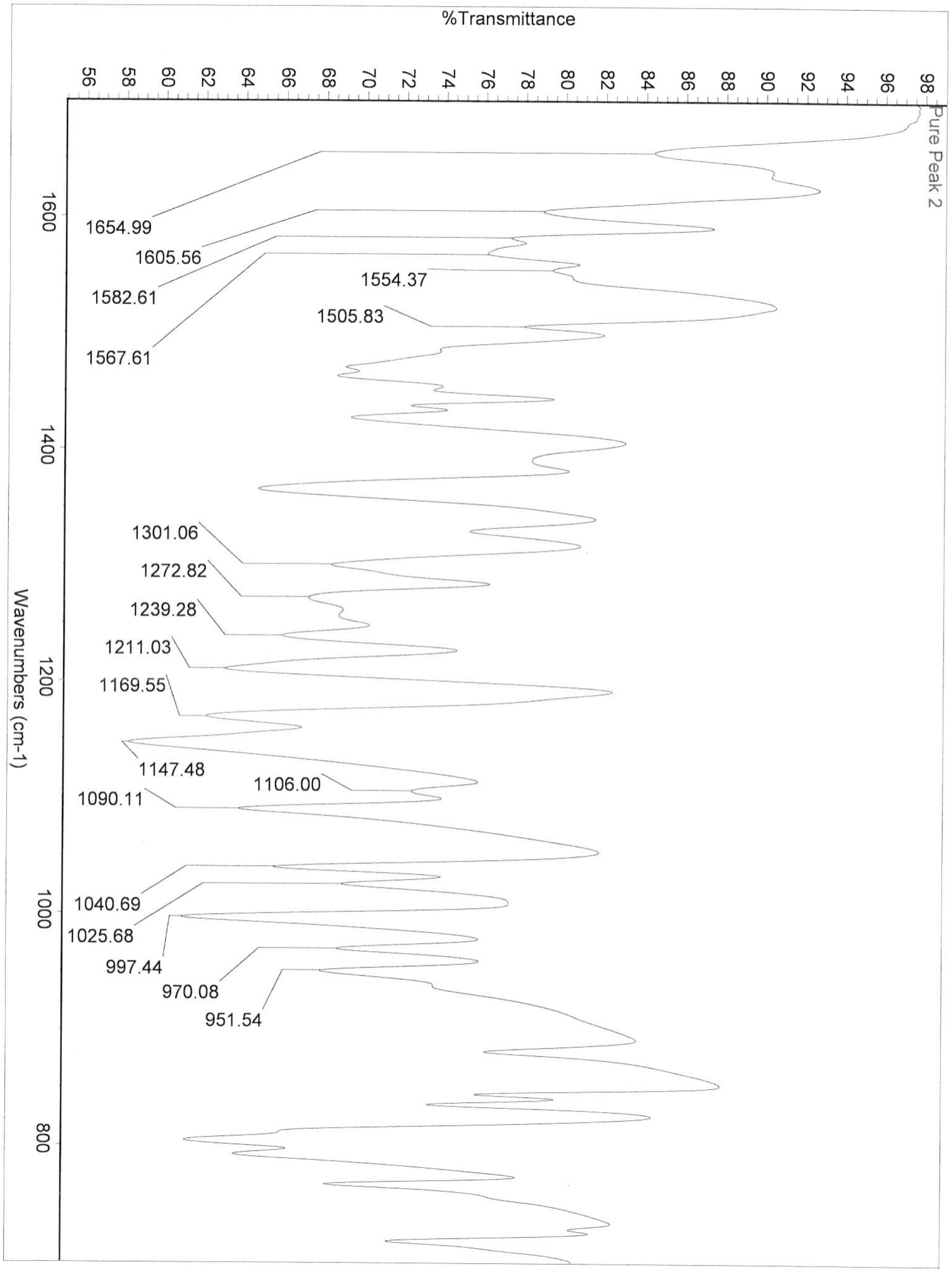
%Transmittance
56
58
60
62
64
66
68
70
72
74
76
78
80
82
84
86
88
90
92
94
96
98
Pure Peak 2
Wavenumbers (cm-1)
1600
1400
1200
1000
800
1654.99
1605.56
1582.61
1567.61
1554.37
1505.83
1301.06
1272.82
1239.28
1211.03
1169.55
1147.48
1106.00
1090.11
1040.69
1025.68
997.44
970.08
951.54

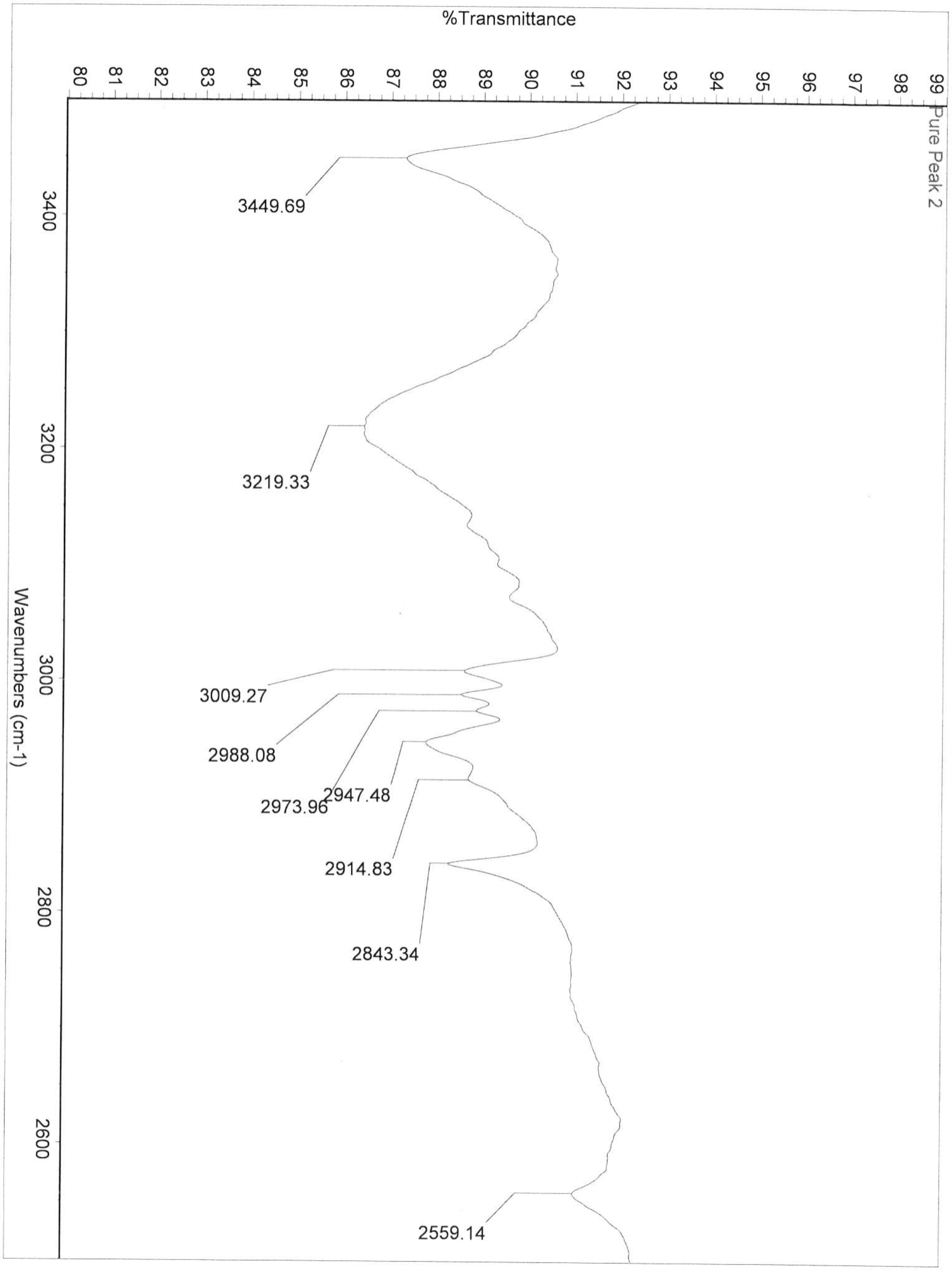
%Transmittance
80
81
82
83
84
85
86
87
88
89
90
91
92
93
94
95
96
97
98
99
Pure Peak 2
3400
3200
3000
2800
2600
Wavenumbers (cm-1)
3449.69
3219.33
3009.27
2988.08
2973.96
2947.48
2914.83
2843.34
2559.14

Qualitative Analysis Report

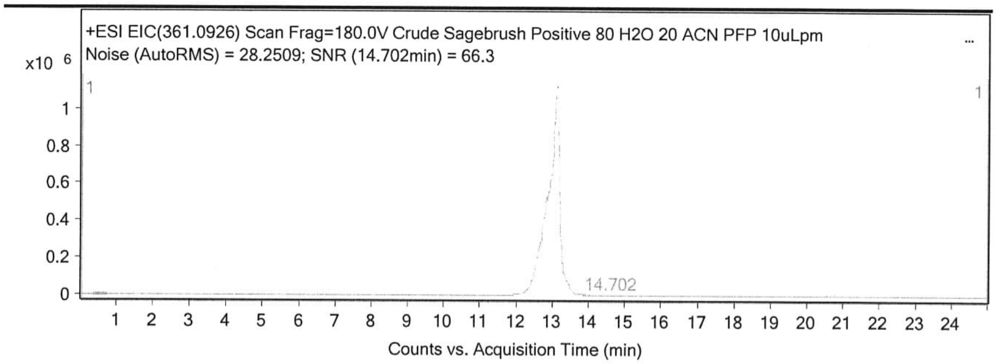

Integration Peak List

Peak	Start	RT	End	Height	Area	Area %	Signal to Noise
1	14.691	14.702	14.724	1872	1739	100	66.3

Noise Measurements

Noise Type	Signal Definition	Noise Multiplier	Noise Value
Auto-RMS	Height	1	28.2508926391602

Noise Regions

Start	End
.397933333	.747933333

Fragmentor Voltage 180 **Collision Energy** 0 **Ionization Mode** ESI

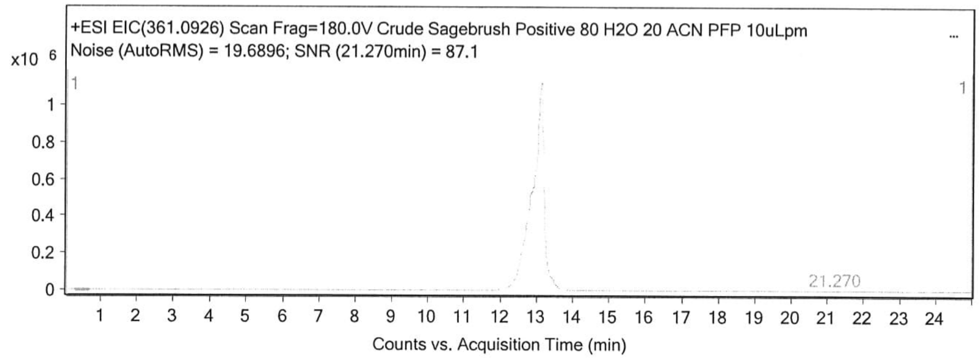

Integration Peak List

Peak	Start	RT	End	Height	Area	Area %	Signal to Noise
1	21.237	21.27	21.347	1714	6164	100	87.1

Noise Measurements

Calculated 361.09231

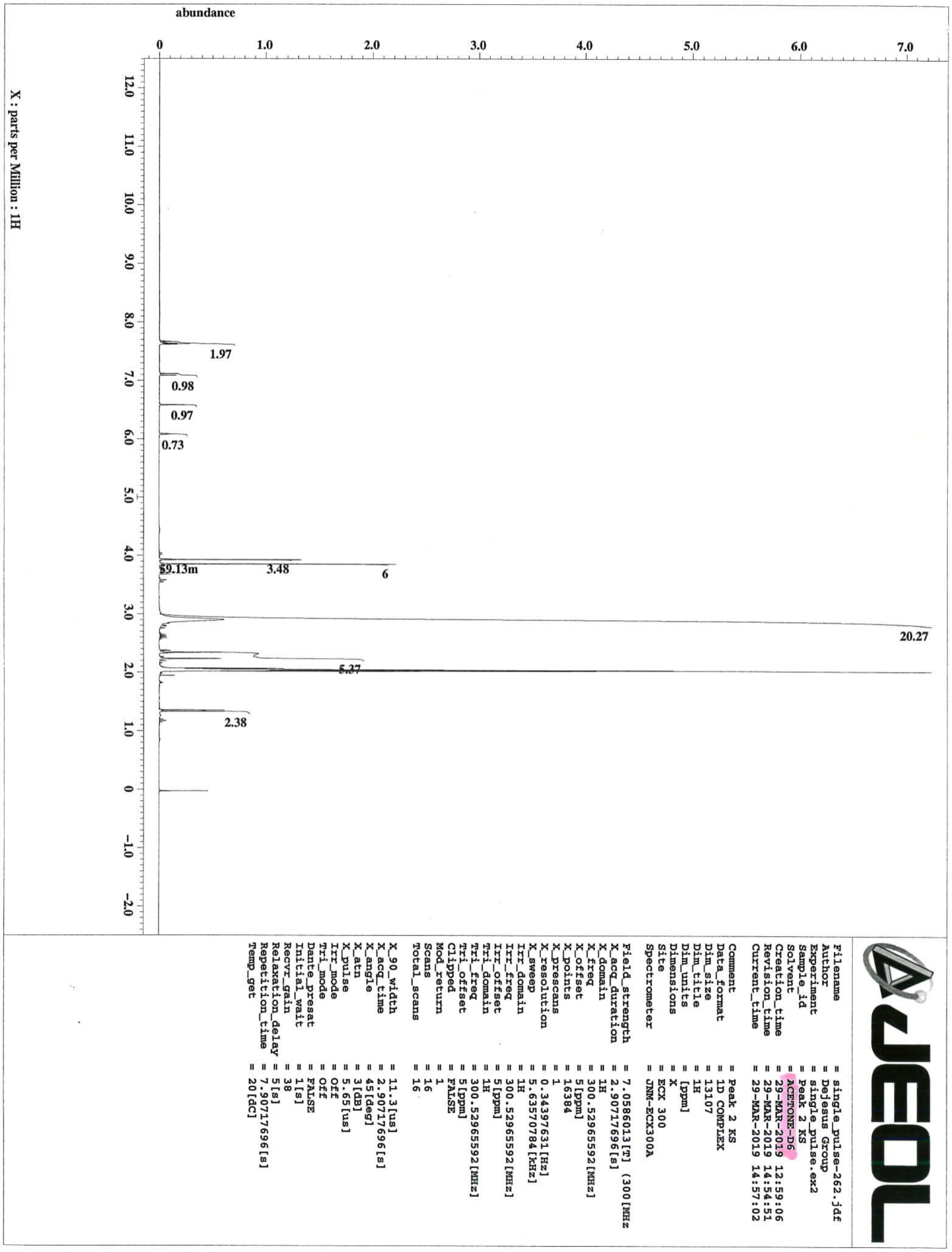
abundance
X : parts per Million : 1H
1.97
0.98
0.97
0.73
59.13m
3.48
6
20.27
5.37
2.38
Filename = single_pulse-262.jdf
Author = Dejesus Group
Experiment = single_pulse.ex2
Sample_id = Peak 2 KS
Solvent = ACETONE-D6
Creation_time = 29-MAR-2019 12:59:06
Revision_time = 29-MAR-2019 14:54:51
Current_time = 29-MAR-2019 14:57:02
Comment = Peak 2 KS
Data_format = 1D COMPLEX
Dim_size = 13107
Dim_title = 1H
Dim_units = [ppm]
Dimensions = X
Site = ECX 300
Spectrometer = JNM-ECX300A
Field_strength = 7.0586013[T] (300[MHz
X_acq_duration = 2.907217696[s]
X_domain = 1H
X_freq = 300.52965592[MHz]
X_offset = 5[ppm]
X_points = 16384
X_prescans = 1
X_resolution = 0.34397631[Hz]
X_sweep = 5.63570784[kHz]
Irr_domain = 1H
Irr_freq = 300.52965592[MHz]
Irr_offset = 5[ppm]
Tri_domain = 1H
Tri_freq = 300.52965592[MHz]
Tri_offset = 5[ppm]
Clipped = FALSE
Mod_return = 1
Scans = 16
Total_scans = 16
X_90_width = 11.3[us]
X_acq_time = 2.907217696[s]
X_angle = 45[deg]
X_atn = 3[dB]
X_pulse = 5.65[us]
Irr_mode = Off
Tri_mode = Off
Dante_presat = FALSE
Initial_wait = 1[s]
Recvr_gain = 38
Relaxation_delay = 5[s]
Repetition_time = 7.907217696[s]
Temp_get = 20[dC]

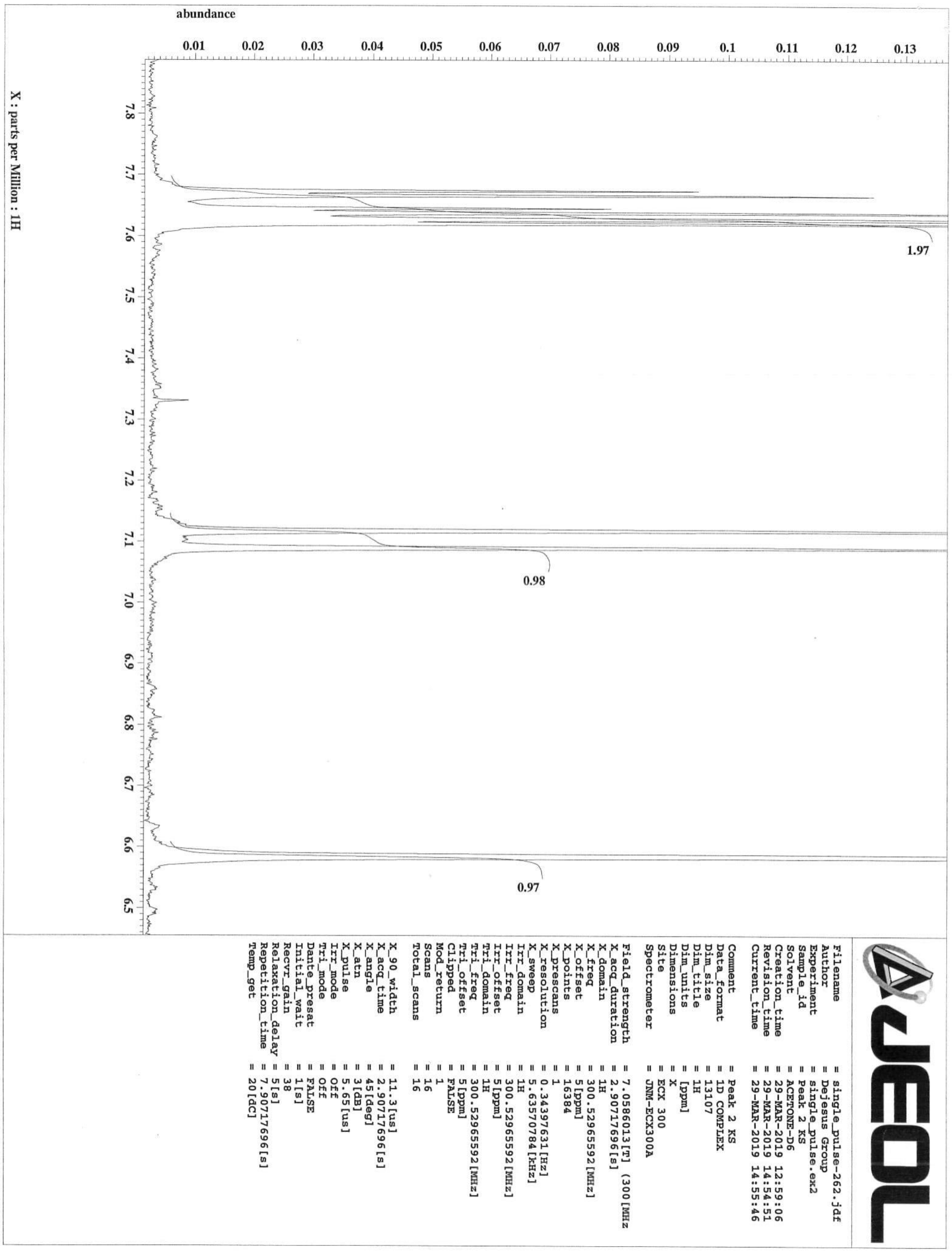
abundance
X : parts per Million : 1H
1.97
0.98
0.97
Filename = single_pulse-262.jdf
Author = Dejesus Group
Experiment = single_pulse.ex2
Sample_id = Peak 2 KS
Solvent = ACETONE-D6
Creation_time = 29-MAR-2019 12:59:06
Revision_time = 29-MAR-2019 14:54:51
Current_time = 29-MAR-2019 14:55:46
Comment = Peak 2 KS
Data_format = 1D COMPLEX
Dim_size = 13107
Dim_title = 1H
Dim_units = [ppm]
Dimensions = X
Site = ECX 300
Spectrometer = JNM-ECX300A
Field_strength = 7.0586013[T] (300[MHz
X_acq_duration = 2.90717696[s]
X_domain = 1H
X_freq = 300.52965592[MHz]
X_offset = 5[ppm]
X_points = 16384
X_prescans = 1
X_resolution = 0.34397631[Hz]
X_sweep = 5.63570784[kHz]
Irr_domain = 1H
Irr_freq = 300.52965592[MHz]
Irr_offset = 5[ppm]
Tri_domain = 1H
Tri_freq = 300.52965592[MHz]
Tri_offset = 5[ppm]
Clipped = FALSE
Mod_return = 1
Scans = 16
Total_scans = 16
X_90_width = 11.3[us]
X_acq_time = 2.90717696[s]
X_angle = 45[deg]
X_atn = 3[dB]
X_pulse = 5.65[us]
Irr_mode = Off
Tri_mode = Off
Dante_presat = FALSE
Initial_wait = 1[s]
Recvr_gain = 38
Relaxation_delay = 5[s]
Repetition_time = 7.90717696[s]
Temp_get = 20[dC]

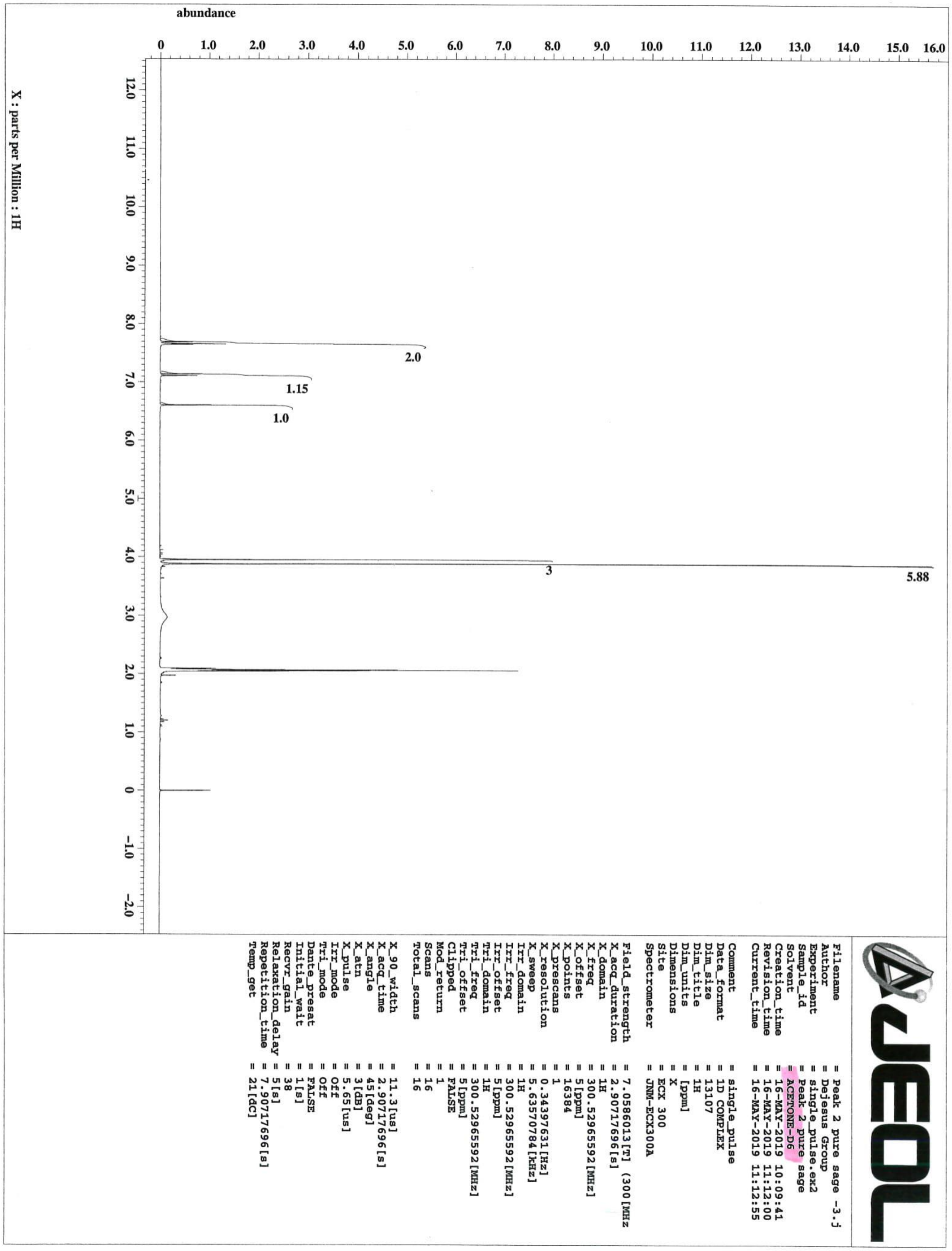
abundance
X : parts per Million : 1H
2.0
1.15
1.0
3
5.88
JEOL
Filename = Peak 2 pure sage -3.j
Author = Dejesus Group
Experiment = single_pulse.ex2
Sample_id = Peak 2 pure sage
Solvent = ACETONE-D6
Creation_time = 16-MAY-2019 10:09:41
Revision_time = 16-MAY-2019 11:12:00
Current_time = 16-MAY-2019 11:12:55
Comment = single_pulse
Data_format = 1D COMPLEX
Dim_size = 13107
Dim_title = 1H
Dim_units = [ppm]
Dimensions = X
Site = ECX 300
Spectrometer = JNM-ECX300A
Field_strength = 7.0586013[T] (300[MHz
X_acq_duration = 2.90717696[s]
X_domain = 1H
X_freq = 300.52965592[MHz]
X_offset = 5[ppm]
X_points = 16384
X_prescans = 1
X_resolution = 0.34397631[Hz]
X_sweep = 5.63570784[kHz]
Irr_domain = 1H
Irr_freq = 300.52965592[MHz]
Irr_offset = 5[ppm]
Tri_domain = 1H
Tri_freq = 300.52965592[MHz]
Tri_offset = 5[ppm]
Clipped = FALSE
Mod_return = 1
Scans = 16
Total_scans = 16
X_90_width = 11.3[us]
X_acq_time = 2.90717696[s]
X_angle = 45[deg]
X_atn = 3[dB]
X_pulse = 5.65[us]
Irr_mode = Off
Tri_mode = Off
Dante_presat = FALSE
Initial_wait = 1[s]
Recvr_gain = 38
Relaxation_delay = 5[s]
Repetition_time = 7.90717696[s]
Temp_get = 21[dC]

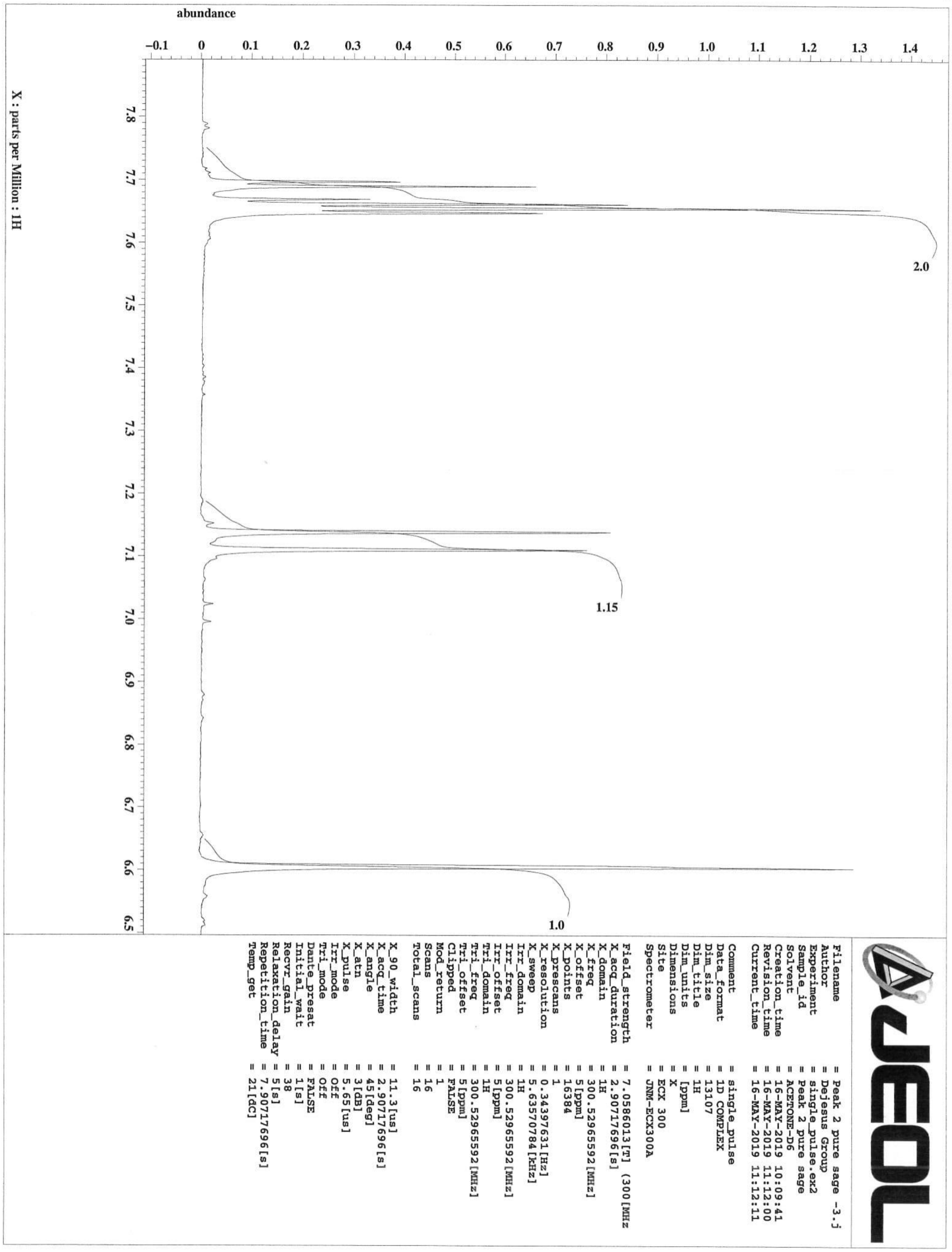
abundance
-0.1 0 0.1 0.2 0.3 0.4 0.5 0.6 0.7 0.8 0.9 1.0 1.1 1.2 1.3 1.4
X : parts per Million : 1H
7.8 7.7 7.6 7.5 7.4 7.3 7.2 7.1 7.0 6.9 6.8 6.7 6.6 6.5
2.0
1.15
1.0
JEOL
Filename = Peak 2 pure sage -3.j
Author = Dejesus Group
Experiment = single_pulse.ex2
Sample_id = Peak 2 pure sage
Solvent = ACETONE-D6
Creation_time = 16-MAY-2019 10:09:41
Revision_time = 16-MAY-2019 11:12:00
Current_time = 16-MAY-2019 11:12:11
Comment = single_pulse
Data_format = 1D COMPLEX
Dim_size = 13107
Dim_title = 1H
Dim_units = [ppm]
Dimensions = X
Site = ECX 300
Spectrometer = JNM-ECX300A
Field_strength = 7.0586013[T] (300[MHz
X_acq_duration = 2.90717696[s]
X_domain = 1H
X_freq = 300.52965592[MHz]
X_offset = 5[ppm]
X_points = 16384
X_prescans = 1
X_resolution = 0.34397631[Hz]
X_sweep = 5.63570784[kHz]
Irr_domain = 1H
Irr_freq = 300.52965592[MHz]
Irr_offset = 5[ppm]
Tri_domain = 1H
Tri_freq = 300.52965592[MHz]
Tri_offset = 5[ppm]
Clipped = FALSE
Mod_return = 1
Scans = 16
Total_scans = 16
X_90_width = 11.3[us]
X_acq_time = 2.90717696[s]
X_angle = 45[deg]
X_atn = 3[dB]
X_pulse = 5.65[us]
Irr_mode = Off
Tri_mode = Off
Dante_presat = FALSE
Initial_wait = 1[s]
Recvr_gain = 38
Relaxation_delay = 5[s]
Repetition_time = 7.90717696[s]
Temp_get = 21[dC]

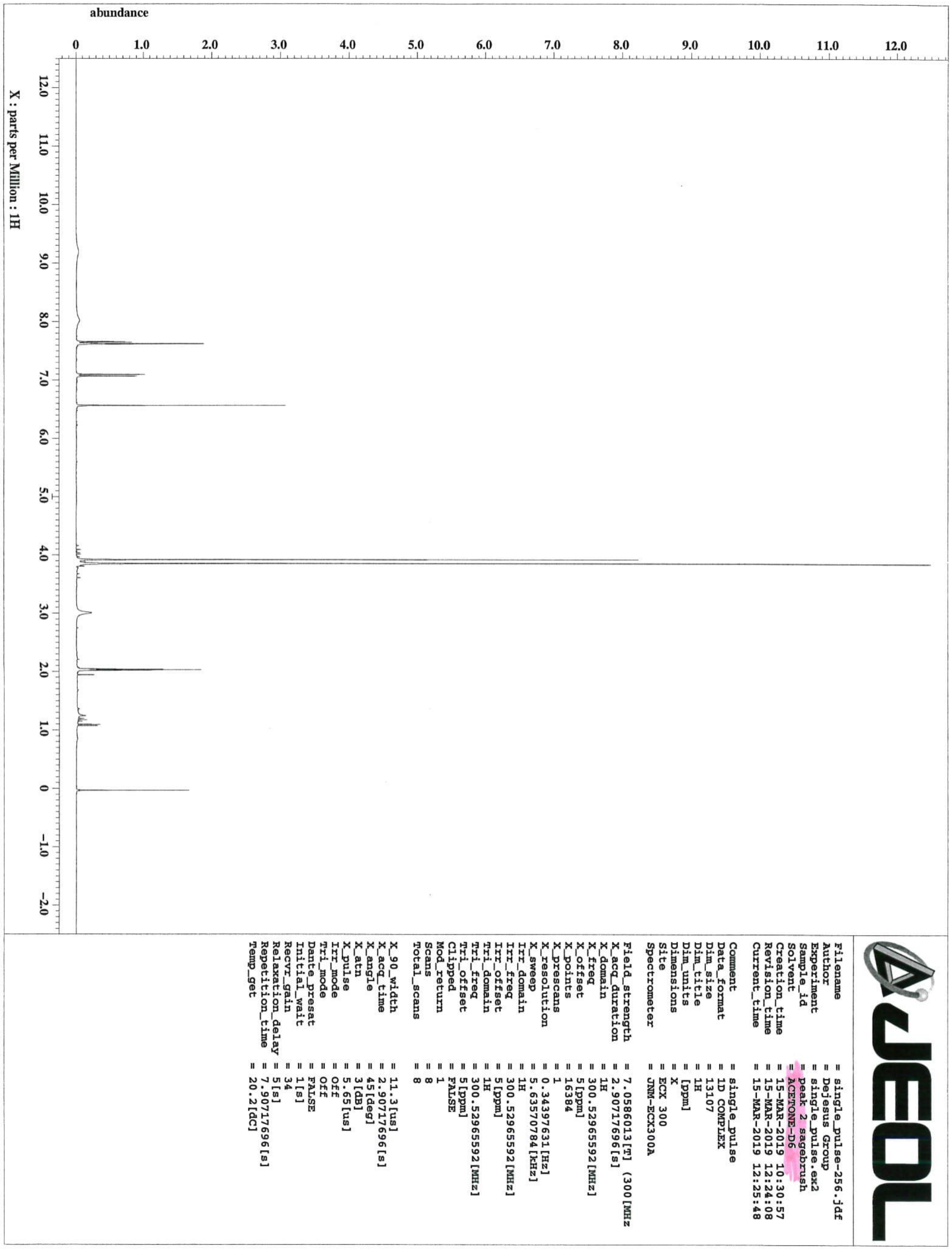
abundance
0 1.0 2.0 3.0 4.0 5.0 6.0 7.0 8.0 9.0 10.0 11.0 12.0
12.0 11.0 10.0 9.0 8.0 7.0 6.0 5.0 4.0 3.0 2.0 1.0 0 -1.0 -2.0
X : parts per Million : 1H
JEOL
Filename = single_pulse-256.jdf
Author = Dejesus Group
Experiment = single_pulse.ex2
Sample_id = peak 2 sagebrush
Solvent = ACETONE-D6
Creation_time = 15-MAR-2019 10:30:57
Revision_time = 15-MAR-2019 12:24:08
Current_time = 15-MAR-2019 12:25:48
Comment = single_pulse
Data_format = 1D COMPLEX
Dim_size = 13107
Dim_title = 1H
Dim_units = [ppm]
Dimensions = X
Site = ECX 300
Spectrometer = JNM-ECX300A
Field_strength = 7.0586013[T] (300[MHz
X_acq_duration = 2.90717696[s]
X_domain = 1H
X_freq = 300.52965592[MHz]
X_offset = 5[ppm]
X_points = 16384
X_prescans = 1
X_resolution = 0.34397631[Hz]
X_sweep = 5.63570784[kHz]
Irr_domain = 1H
Irr_freq = 300.52965592[MHz]
Irr_offset = 5[ppm]
Tri_domain = 1H
Tri_freq = 300.52965592[MHz]
Tri_offset = 5[ppm]
Clipped = FALSE
Mod_return = 1
Scans = 8
Total_scans = 8
X_90_width = 11.3[us]
X_acq_time = 2.90717696[s]
X_angle = 45[deg]
X_atn = 3[dB]
X_pulse = 5.65[us]
Irr_mode = Off
Tri_mode = Off
Dante_presat = FALSE
Initial_wait = 1[s]
Recvr_gain = 34
Relaxation_delay = 5[s]
Repetition_time = 7.90717696[s]
Temp_get = 20.2[dC]

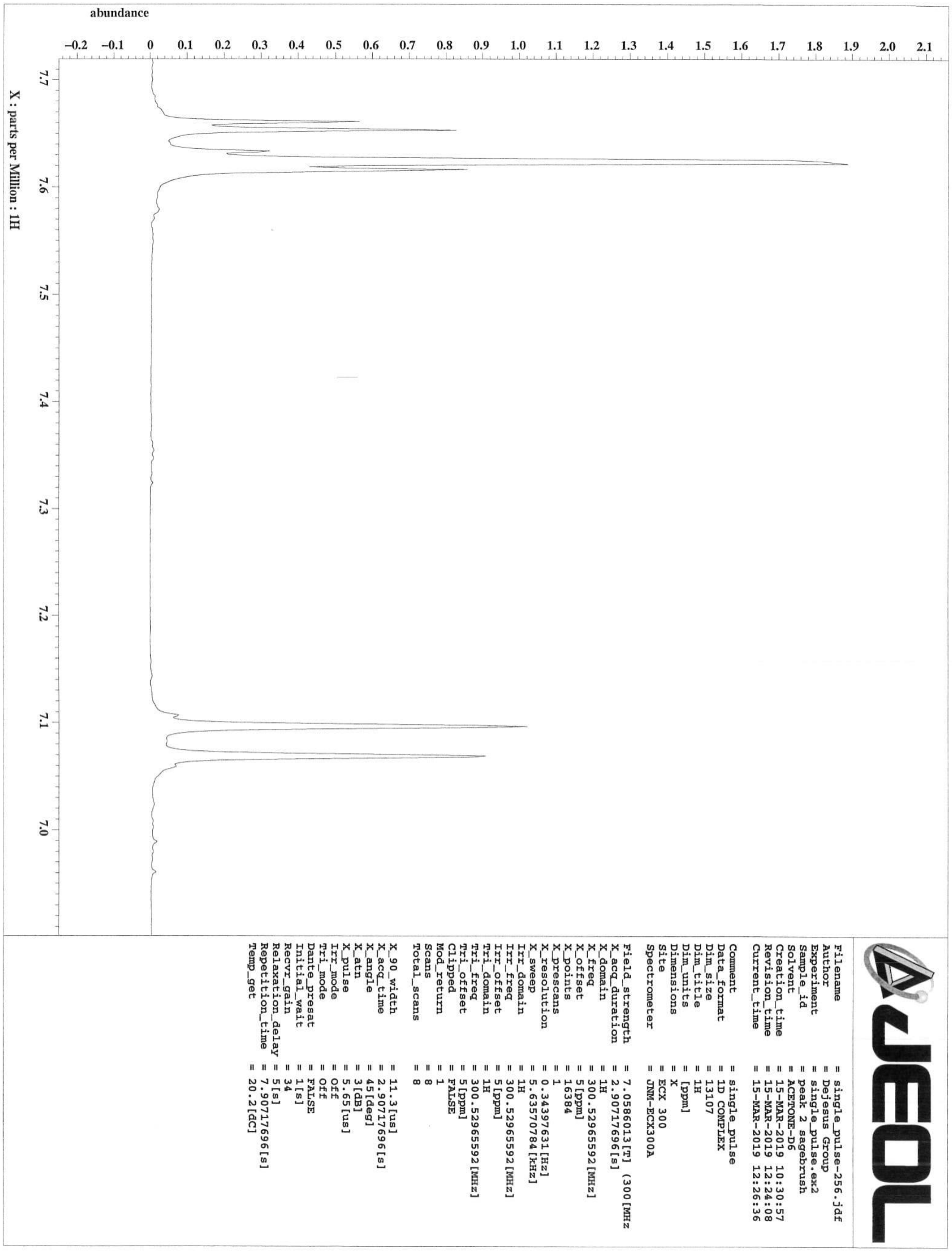
abundance
−0.2 −0.1 0 0.1 0.2 0.3 0.4 0.5 0.6 0.7 0.8 0.9 1.0 1.1 1.2 1.3 1.4 1.5 1.6 1.7 1.8 1.9 2.0 2.1
7.7 7.6 7.5 7.4 7.3 7.2 7.1 7.0
X : parts per Million : 1H
JEOL
Filename = single_pulse-256.jdf
Author = Dejesus Group
Experiment = single_pulse.ex2
Sample_id = peak 2 sagebrush
Solvent = ACETONE-D6
Creation_time = 15-MAR-2019 10:30:57
Revision_time = 15-MAR-2019 12:24:08
Current_time = 15-MAR-2019 12:26:36
Comment = single_pulse
Data_format = 1D COMPLEX
Dim_size = 13107
Dim_title = 1H
Dim_units = [ppm]
Dimensions = X
Site = ECX 300
Spectrometer = JNM-ECX300A
Field_strength = 7.0586013[T] (300[MHz
X_acq_duration = 2.90717696[s]
X_domain = 1H
X_freq = 300.52965592[MHz]
X_offset = 5[ppm]
X_points = 16384
X_prescans = 1
X_resolution = 0.34397631[Hz]
X_sweep = 5.63570784[kHz]
Irr_domain = 1H
Irr_freq = 300.52965592[MHz]
Irr_offset = 5[ppm]
Tri_domain = 1H
Tri_freq = 300.52965592[MHz]
Tri_offset = 5[ppm]
Clipped = FALSE
Mod_return = 1
Scans = 8
Total_scans = 8
X_90_width = 11.3[us]
X_acq_time = 2.90717696[s]
X_angle = 45[deg]
X_atn = 3[dB]
X_pulse = 5.65[us]
Irr_mode = Off
Tri_mode = Off
Dante_presat = FALSE
Initial_wait = 1[s]
Recvr_gain = 34
Relaxation_delay = 5[s]
Repetition_time = 7.90717696[s]
Temp_get = 20.2[dC]

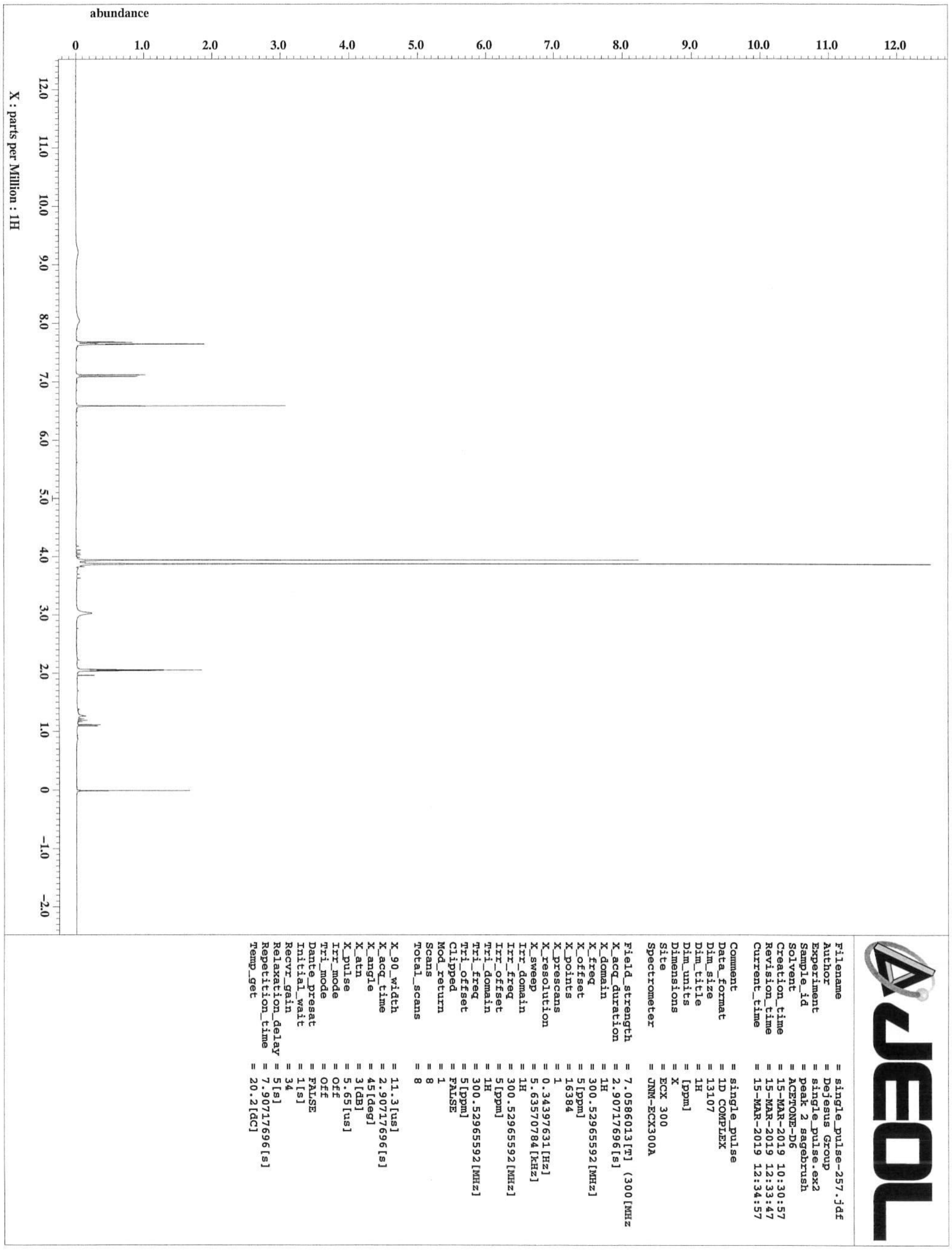
abundance
0
1.0
2.0
3.0
4.0
5.0
6.0
7.0
8.0
9.0
10.0
11.0
12.0
X : parts per Million : 1H
12.0
11.0
10.0
9.0
8.0
7.0
6.0
5.0
4.0
3.0
2.0
1.0
0
-1.0
-2.0
JEOL
Filename = single_pulse-257.jdf
Author = Dejesus Group
Experiment = single_pulse.ex2
Sample_id = peak 2 Sagebrush
Solvent = ACETONE-D6
Creation_time = 15-MAR-2019 10:30:57
Revision_time = 15-MAR-2019 12:33:47
Current_time = 15-MAR-2019 12:34:57
Comment = single_pulse
Data_format = 1D COMPLEX
Dim_size = 13107
Dim_title = 1H
Dim_units = [ppm]
Dimensions = X
Site = ECX 300
Spectrometer = JNM-ECX300A
Field_strength = 7.0586013[T] (300[MHz
X_acq_duration = 2.90717696[s]
X_domain = 1H
X_freq = 300.52965592[MHz]
X_offset = 5[ppm]
X_points = 16384
X_prescans = 1
X_resolution = 0.34397631[Hz]
X_sweep = 5.63570784[kHz]
Irr_domain = 1H
Irr_freq = 300.52965592[MHz]
Irr_offset = 5[ppm]
Tri_domain = 1H
Tri_freq = 300.52965592[MHz]
Tri_offset = 5[ppm]
Clipped = FALSE
Mod_return = 1
Scans = 8
Total_scans = 8
X_90_width = 11.3[us]
X_acq_time = 2.90717696[s]
X_angle = 45[deg]
X_atn = 3[dB]
X_pulse = 5.65[us]
Irr_mode = Off
Tri_mode = Off
Dante_presat = FALSE
Initial_wait = 1[s]
Recvr_gain = 34
Relaxation_delay = 5[s]
Repetition_time = 7.90717696[s]
Temp_get = 20.2[dC]

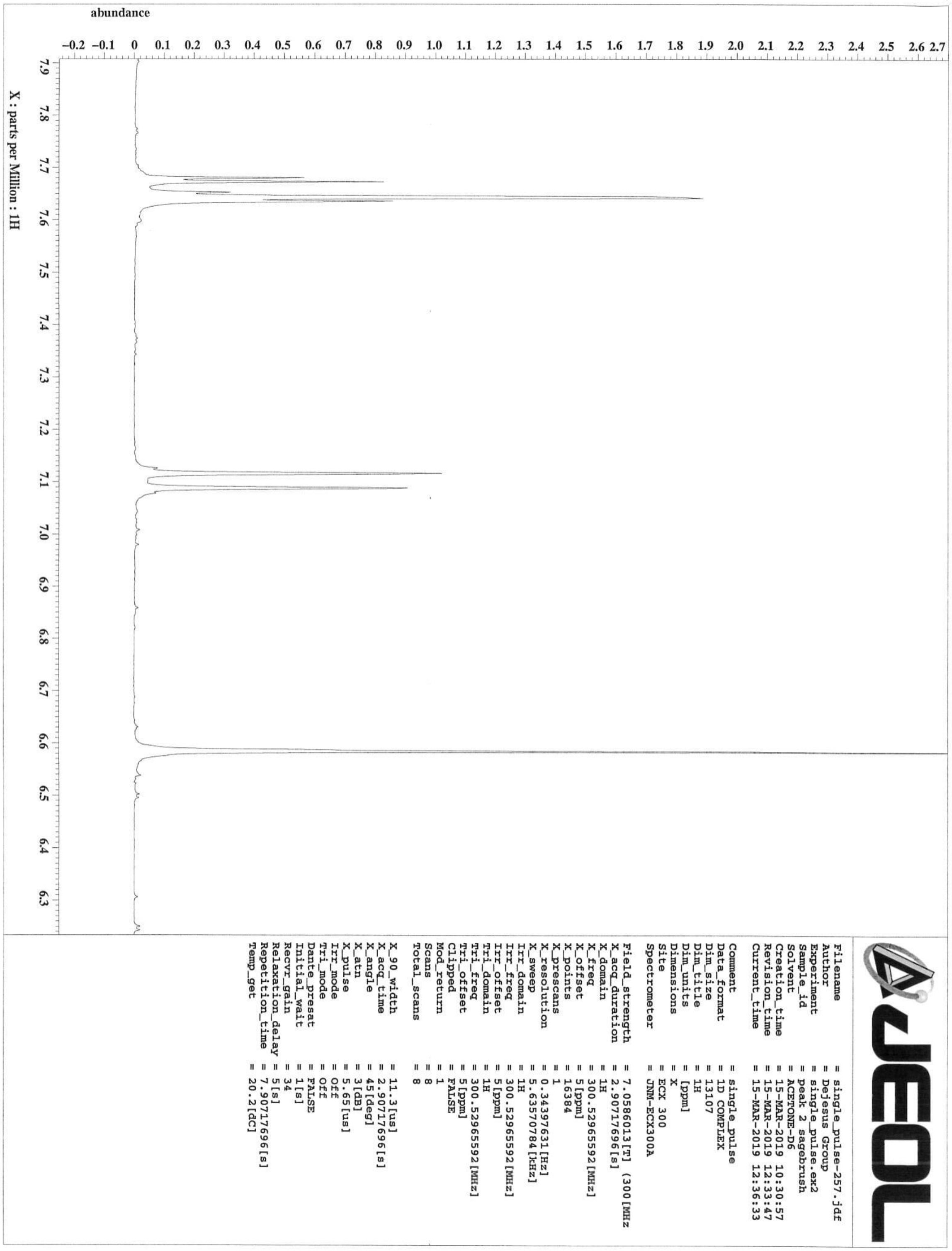
abundance
−0.2 −0.1 0 0.1 0.2 0.3 0.4 0.5 0.6 0.7 0.8 0.9 1.0 1.1 1.2 1.3 1.4 1.5 1.6 1.7 1.8 1.9 2.0 2.1 2.2 2.3 2.4 2.5 2.6 2.7
7.9 7.8 7.7 7.6 7.5 7.4 7.3 7.2 7.1 7.0 6.9 6.8 6.7 6.6 6.5 6.4 6.3
X : parts per Million : 1H
JEOL
Filename = single_pulse-257.jdf
Author = Dejesus Group
Experiment = single_pulse.ex2
Sample_id = peak 2 sagebrush
Solvent = ACETONE-D6
Creation_time = 15-MAR-2019 10:30:57
Revision_time = 15-MAR-2019 12:33:47
Current_time = 15-MAR-2019 12:36:33
Comment = single_pulse
Data_format = 1D COMPLEX
Dim_size = 13107
Dim_title = 1H
Dim_units = [ppm]
Dimensions = X
Site = ECX 300
Spectrometer = JNM-ECX300A
Field_strength = 7.0586013[T] (300[MHz
X_acq_duration = 2.90717696[s]
X_domain = 1H
X_freq = 300.52965592[MHz]
X_offset = 5[ppm]
X_points = 16384
X_prescans = 1
X_resolution = 0.34397631[Hz]
X_sweep = 5.63570784[kHz]
Irr_domain = 1H
Irr_freq = 300.52965592[MHz]
Irr_offset = 5[ppm]
Tri_domain = 1H
Tri_freq = 300.52965592[MHz]
Tri_offset = 5[ppm]
Clipped = FALSE
Mod_return = 1
Scans = 8
Total_scans = 8
X_90_width = 11.3[us]
X_acq_time = 2.90717696[s]
X_angle = 45[deg]
X_atn = 3[dB]
X_pulse = 5.65[us]
Irr_mode = Off
Tri_mode = Off
Dante_presat = FALSE
Initial_wait = 1[s]
Recvr_gain = 34
Relaxation_delay = 5[s]
Repetition_time = 7.90717696[s]
Temp_get = 20.2[dC]
