# Supplementary material for: Sesquiterpene Lactones and Flavonoid from the Leaves of Basin Big Sagebrush (Artemisia tridentata subsp. tridentata): Isolation, Characterization and Biological Activities
Source: Molecules. 2024 Feb 9;29(4):802. doi: 10.3390/molecules29040802 (PMC10892904; doi:10.3390/molecules29040802)
Supplement: Supplementary file 1 [file molecules-29-00802-s001.zip › molecules-2800749-supplementary/Supplementary Data/Supplementry Data-2- LC-MS data.pdf]

Qualitative Analysis Report

**Data File**	Crude sage 1ugpml in Agilent column2.d	**Sample Name**	Cude sage 1ug/ml
**Sample Type**	Sample	**Position**	P1-A1
**Instrument Name**	Instrument 1	**User Name**	
**Acq Method**	Agilent 1p8 micron 3097 ACN 0p6mlpmin.m	**Acquired Time**	8/28/2019 10:19:33 AM (UTC-06:00)
**IRM Calibration Status**	Success	**DA Method**	Agilent 1p8 micron 3097 ACN 0p6mlpmin.m
**Comment**			
**Sample Group**		**Info.**	
**Stream Name**	LC 1	**Acquisition Time (Local)**	8/28/2019 10:19:33 AM (UTC-06:00)
**Acquisition SW Version**	6200 series TOF/6500 series Q-TOF B.08.00 (B8058.3 SP1)	**QTOF Driver Version**	8.00.00
**QTOF Firmware Version**	20.712	**Tune Mass Range Max.**	3200

Chromatograms

Fragmentor Voltage 180 **Collision Energy** 0 **Ionization Mode** ESI

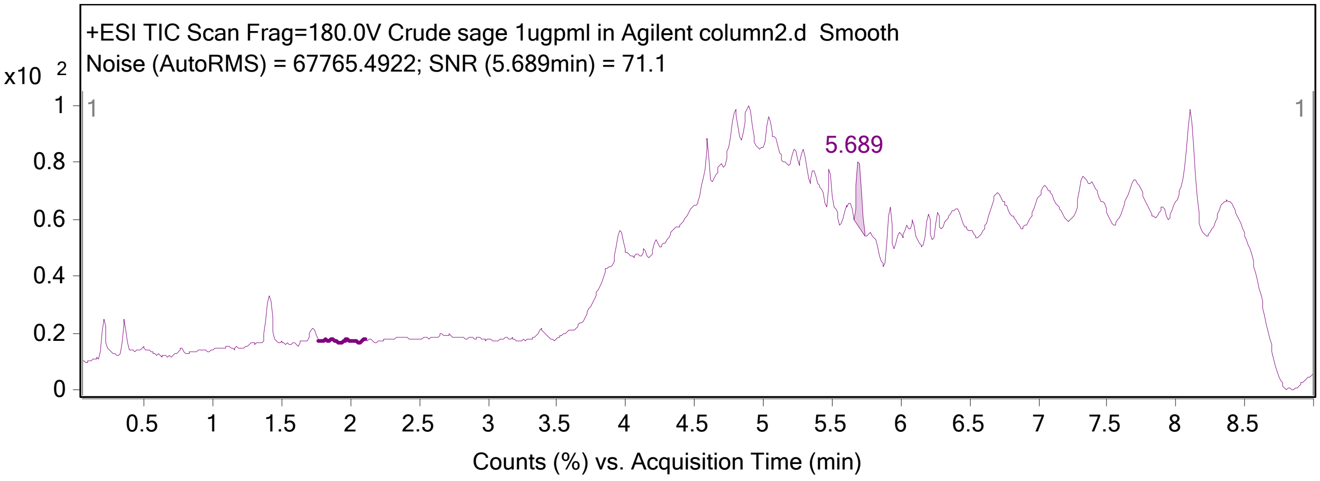

Integration Peak List

Peak	Start	RT	End	Height	Area	Area %	Signal to Noise
1	5.656	5.689	5.744	4816429	11408062	100	71.1

Noise Measurements

Noise Type	Signal Definition	Noise Multiplier	Noise Value
Auto-RMS	Height	1	67765.4921875

Noise Regions

Start	End
1.770533333	2.120533333

Fragmentor Voltage 180 **Collision Energy** 0 **Ionization Mode** ESI

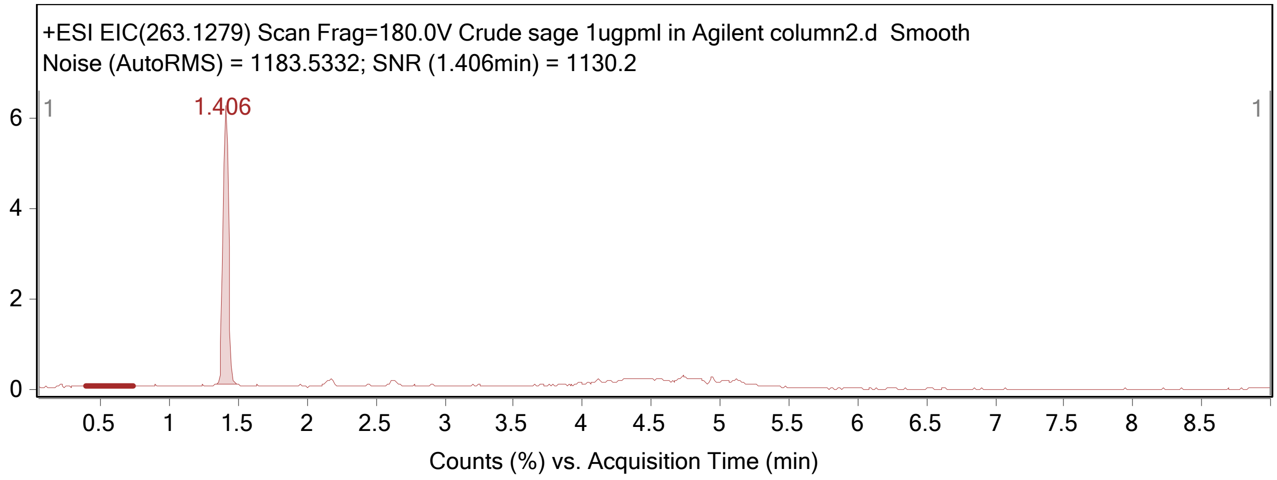

Integration Peak List

Peak	Start	RT	End	Height	Area	Area %	Signal to Noise
1	1.329	1.406	1.484	1337657	3573539	100	1130.2

Noise Measurements

Noise Type	Signal Definition	Noise Multiplier	Noise Value
Auto-RMS	Height	1	1183.533203125

Noise Regions

Start	End
.390866667	.740866667

Fragmentor Voltage 180 **Collision Energy** 0 **Ionization Mode** ESI

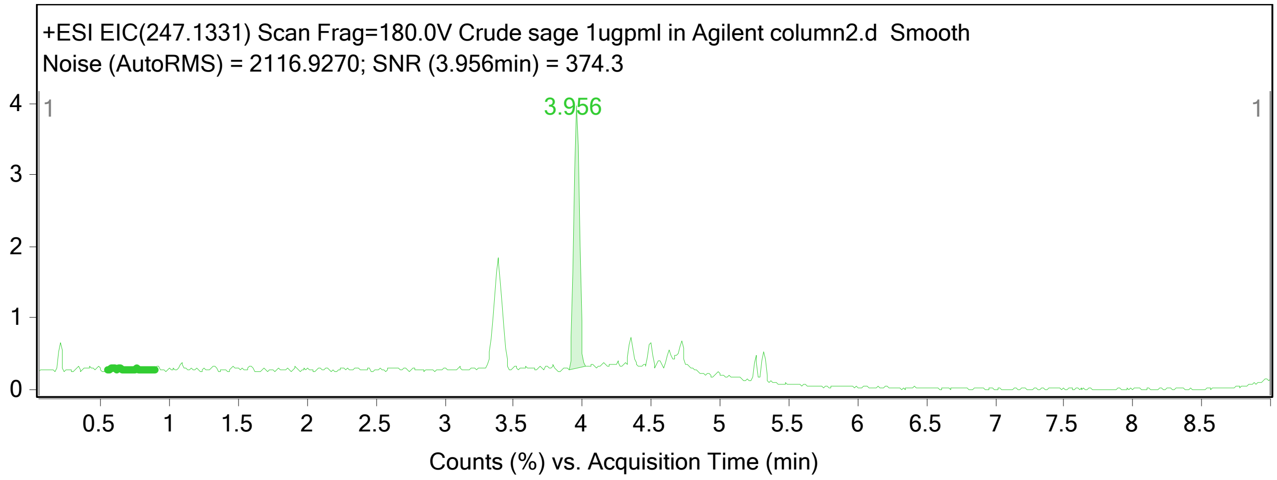

Integration Peak List

Peak	Start	RT	End	Height	Area	Area %	Signal to Noise
1	3.901	3.956	4.022	792358	2005550	100	374.3

Qualitative Analysis Report

Noise Measurements

Noise Type	Signal Definition	Noise Multiplier	Noise Value
Auto-RMS	Height	1	2116.92700195313

Noise Regions

Start	End
.545383333	.895383333

Fragmentor Voltage 180 **Collision Energy** 0 **Ionization Mode** ESI

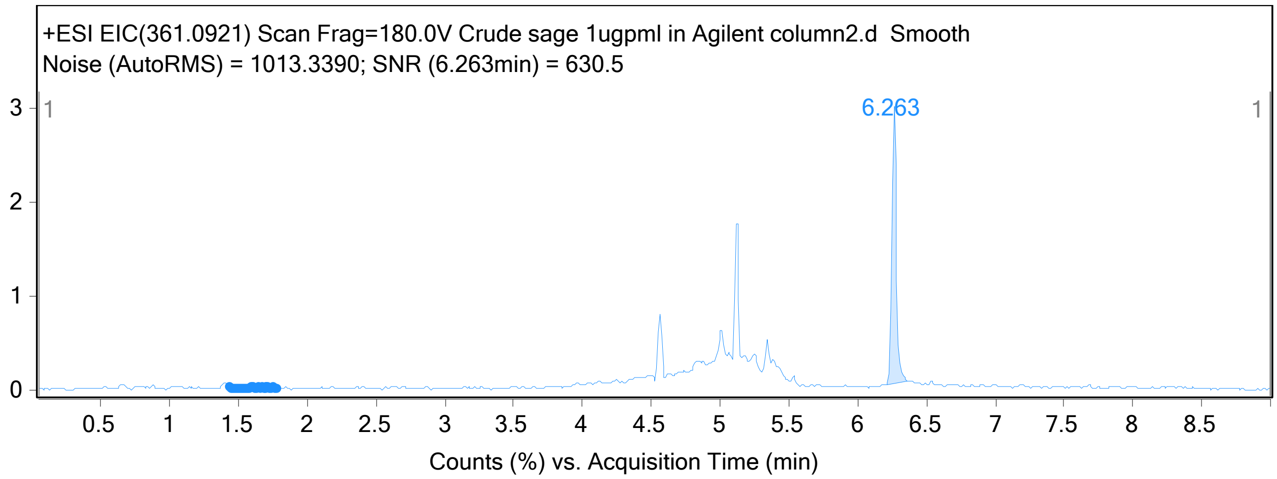

Integration Peak List

Peak	Start	RT	End	Height	Area	Area %	Signal to Noise
1	6.208	6.263	6.362	638890	1470912	100	630.5

Noise Measurements

Noise Type	Signal Definition	Noise Multiplier	Noise Value
Auto-RMS	Height	1	1013.33898925781

Noise Regions

Start	End
1.439416667	1.789416667

Spectra

Spectrum Source	Fragmentor Voltage	Collision Energy	Ionization Mode
Peak (1) in "+ TIC Scan Smo"	180	0	ESI

Qualitative Analysis Report

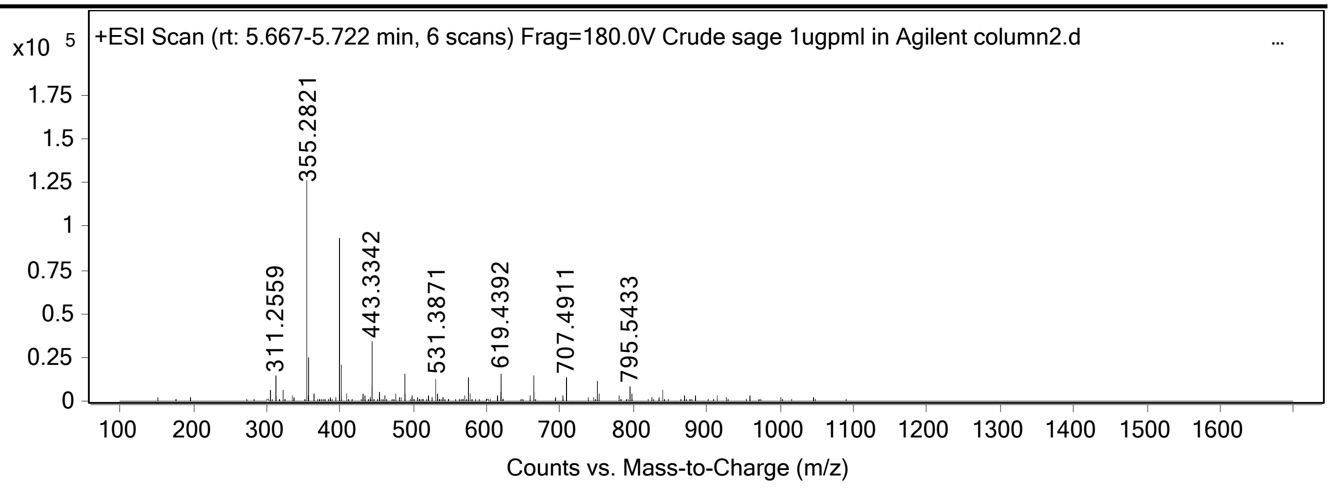

Spectrum Source	Fragmentor Voltage	Collision Energy	Ionization Mode
Peak (1) in "+ EIC(263.1279) Scan Smo"	180	0	ESI

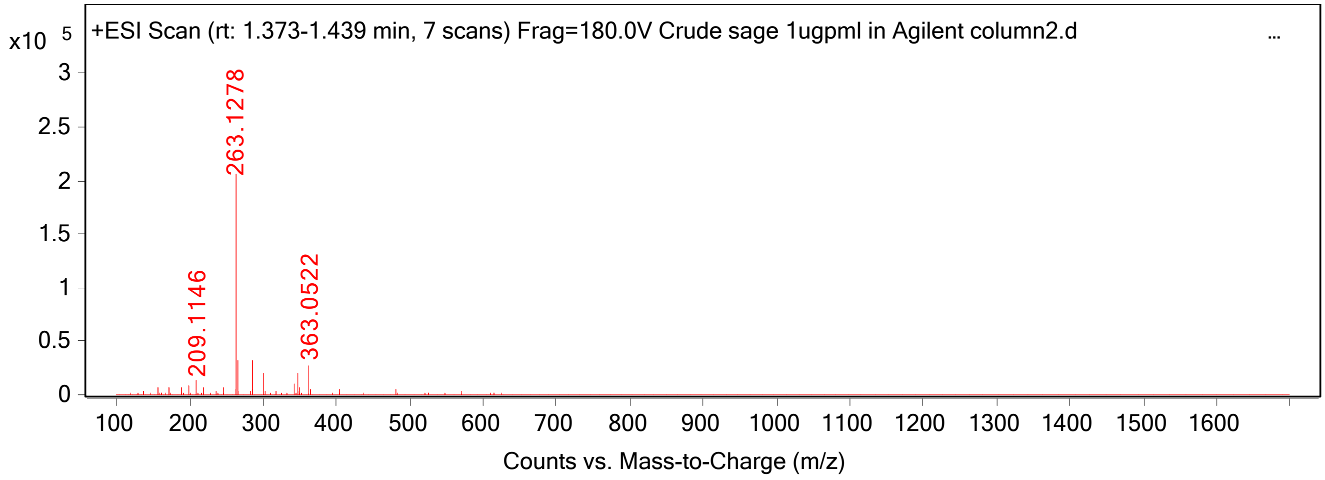

Spectrum Source	Fragmentor Voltage	Collision Energy	Ionization Mode
Peak (1) in "+ EIC(247.1331) Scan Smo"	180	0	ESI

Qualitative Analysis Report

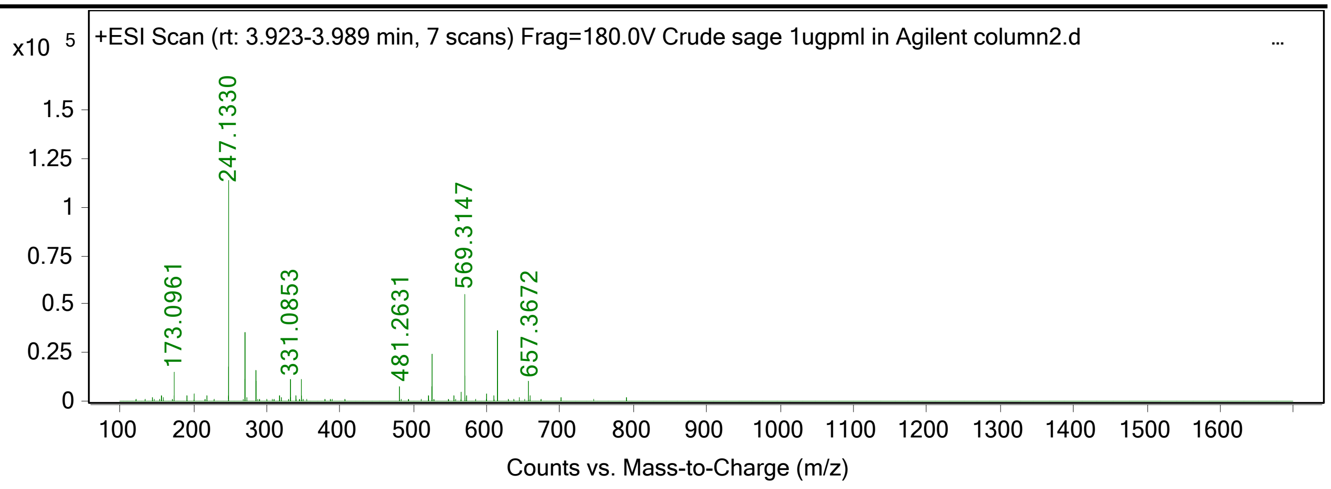

Spectrum Source	Fragmentor Voltage	Collision Energy	Ionization Mode
Peak (1) in "+ EIC(361.0921) Scan Smo"	180	0	ESI

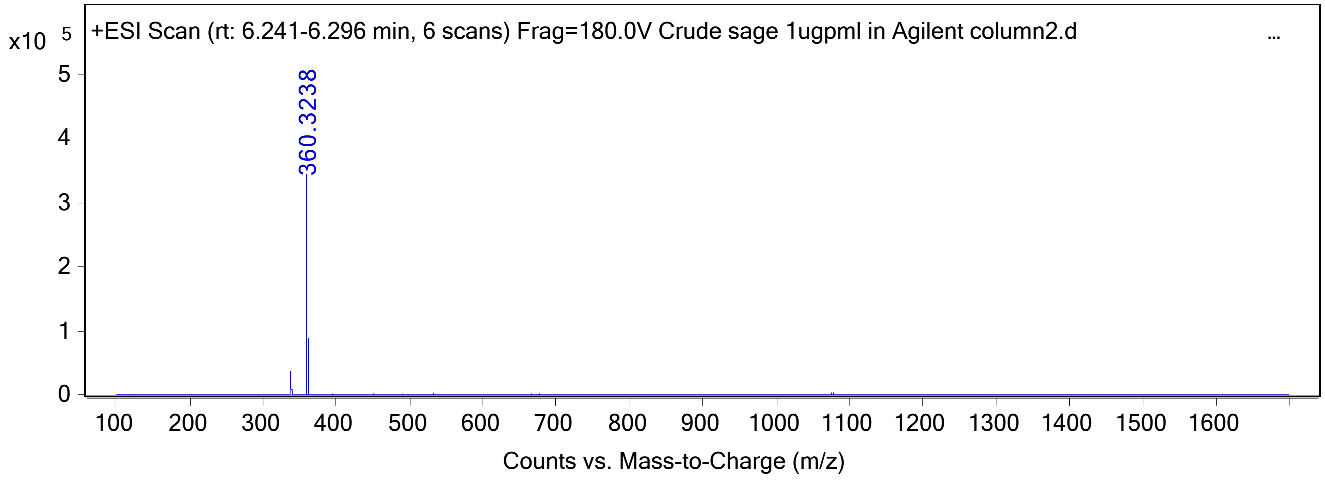

--- End Of Report ---
