# Supplementary figures and images for: Sesquiterpene Lactones and Flavonoid from the Leaves of Basin Big Sagebrush (Artemisia tridentata subsp. tridentata): Isolation, Characterization and Biological Activities
Source: Molecules. 2024 Feb 9;29(4):802. doi: 10.3390/molecules29040802 (PMC10892904; doi:10.3390/molecules29040802)

### 6.3 Achillin NMR Spectra

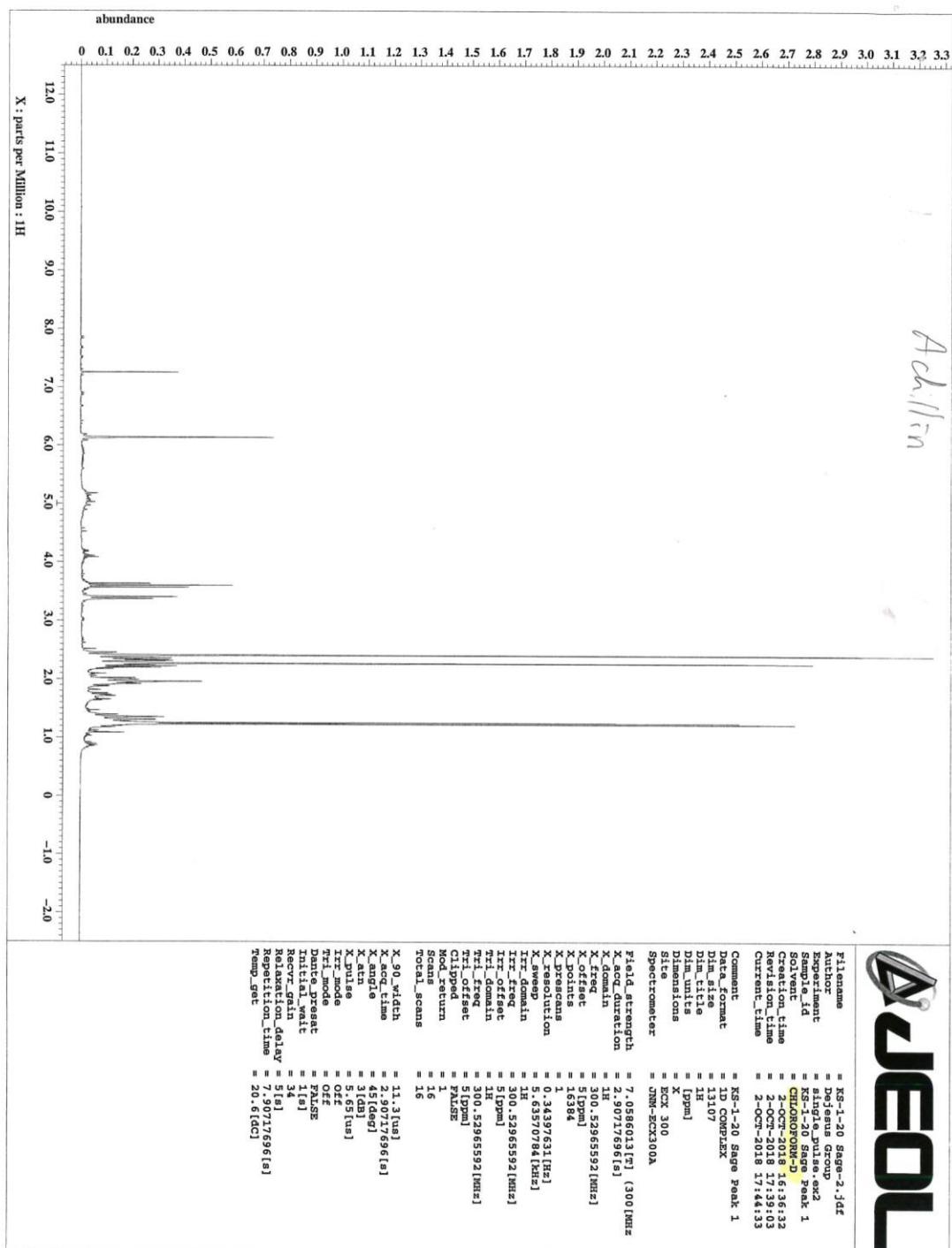

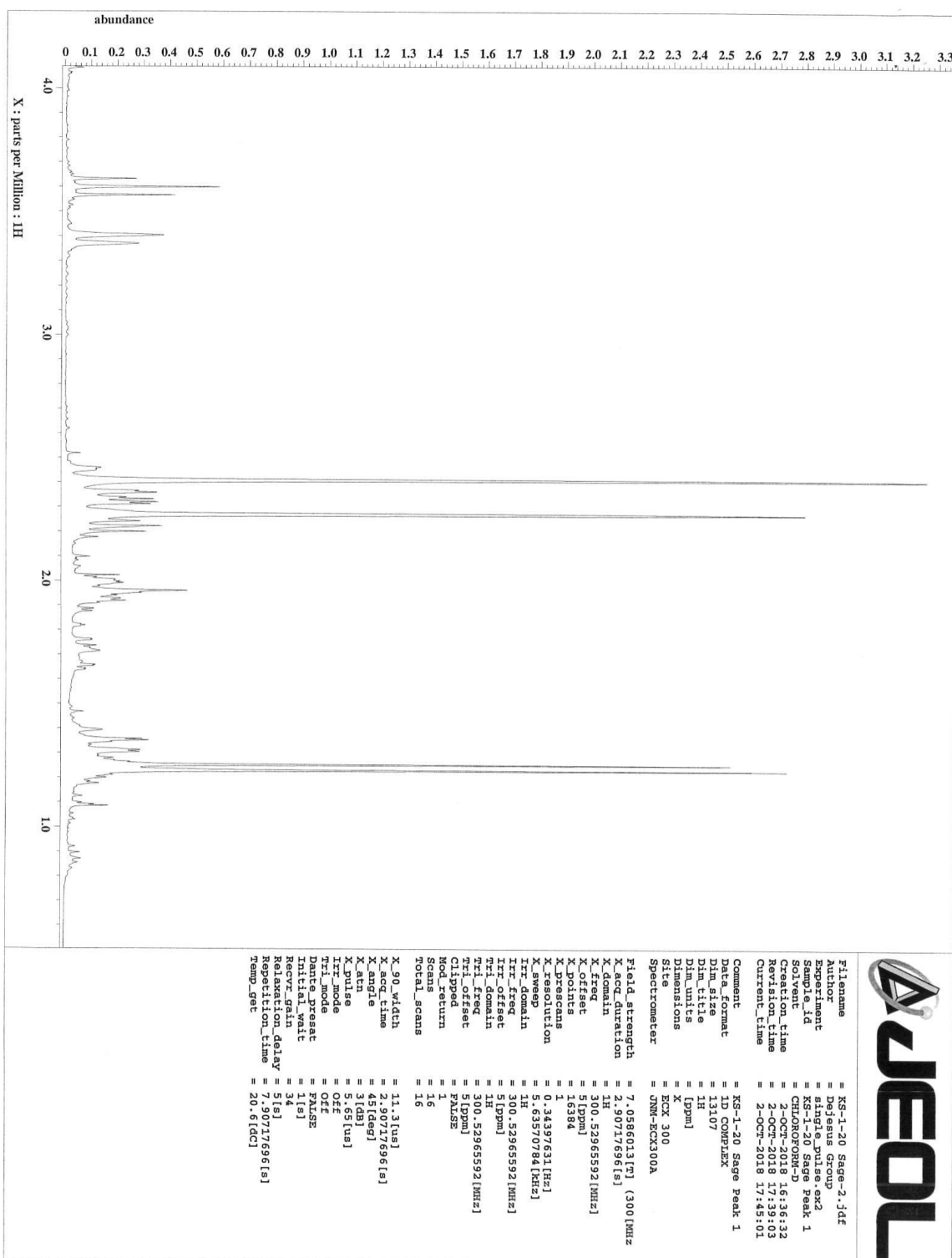

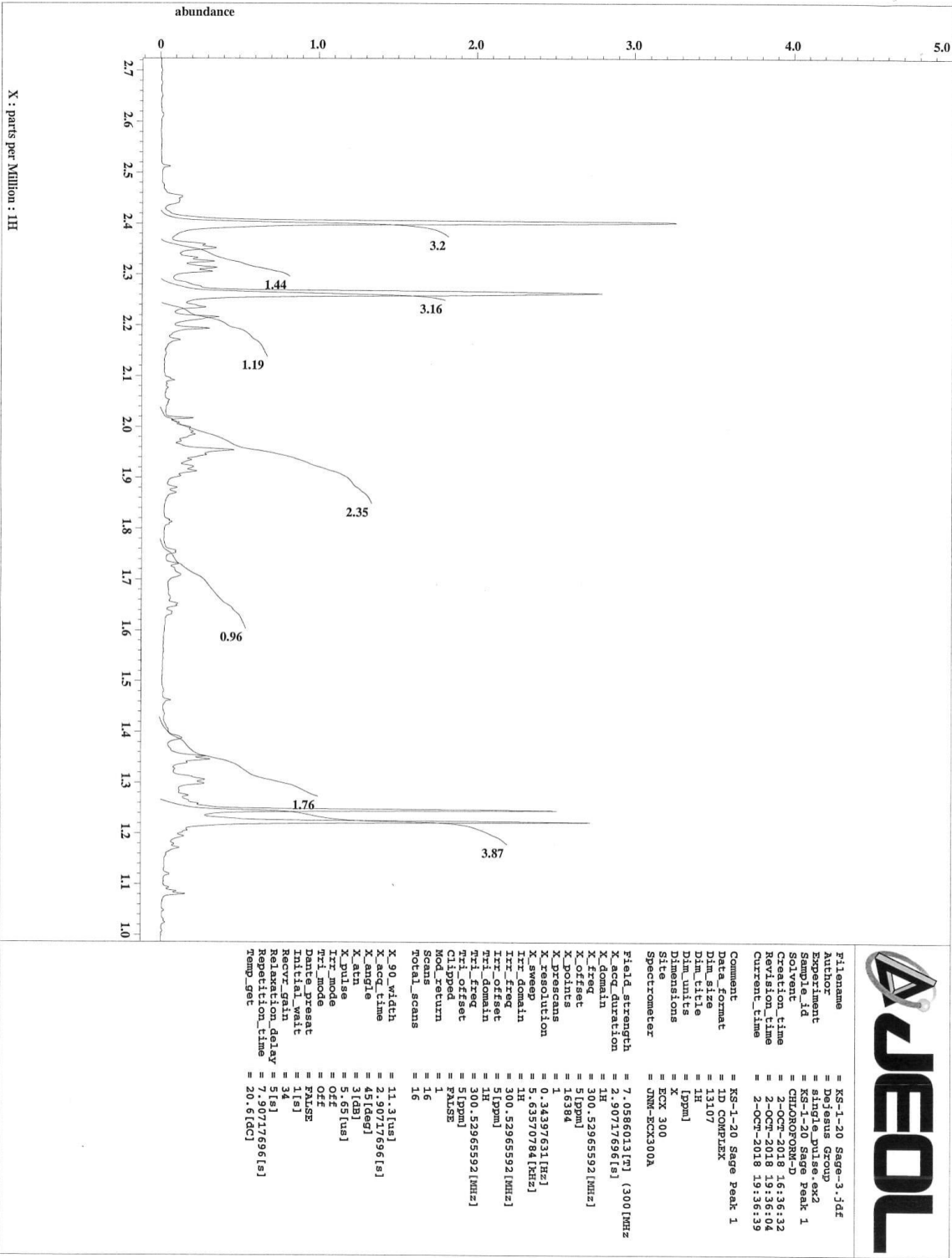

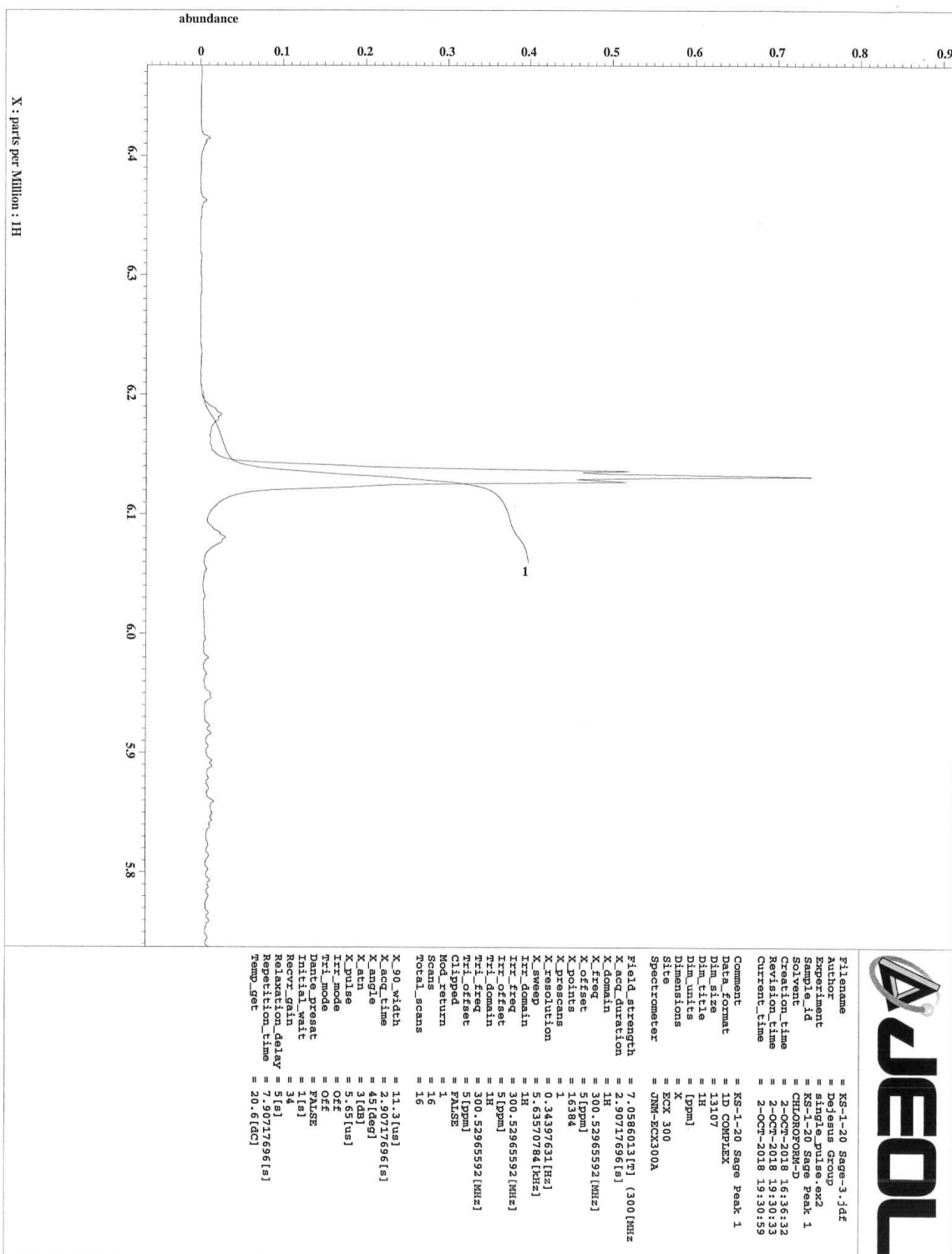

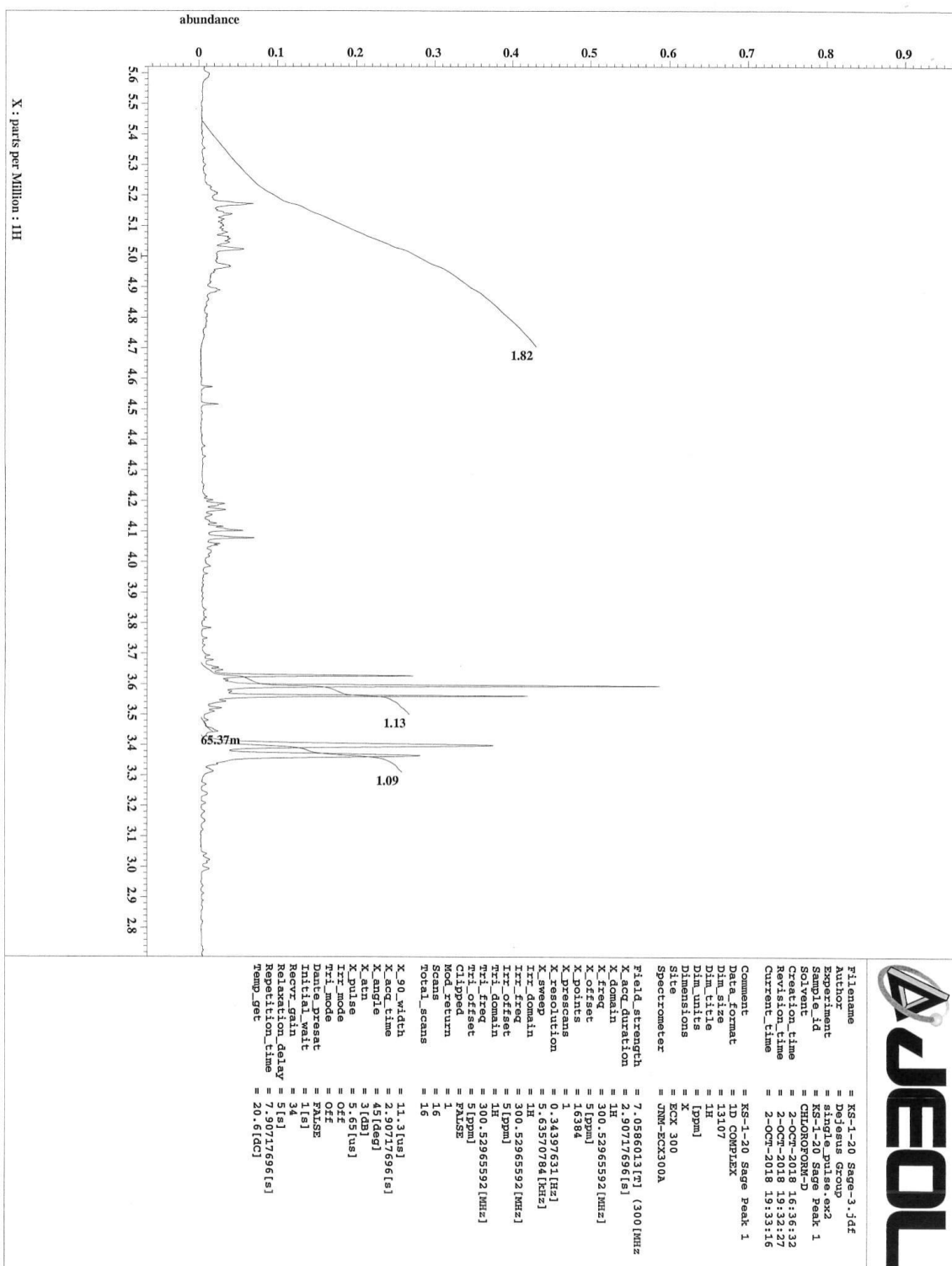

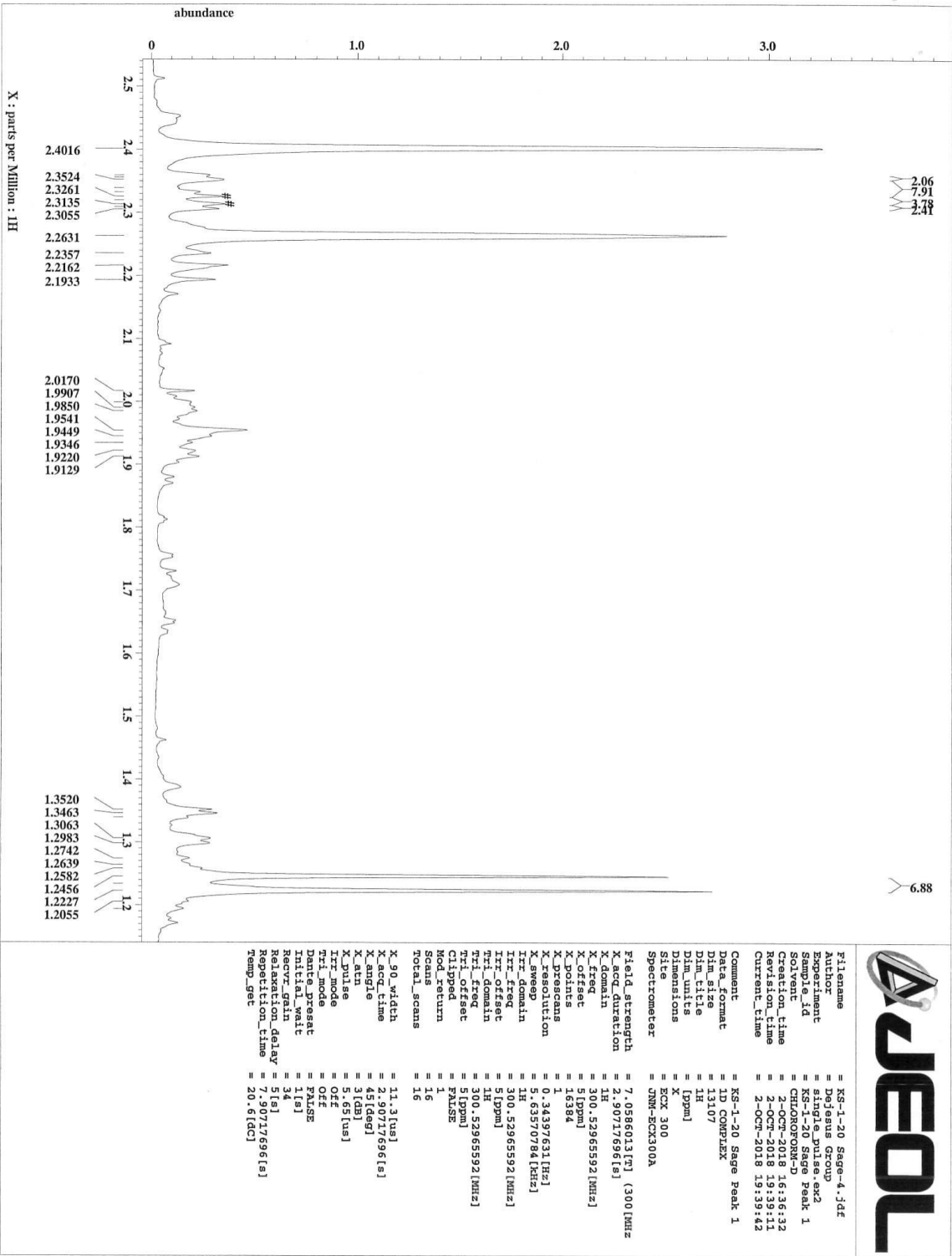

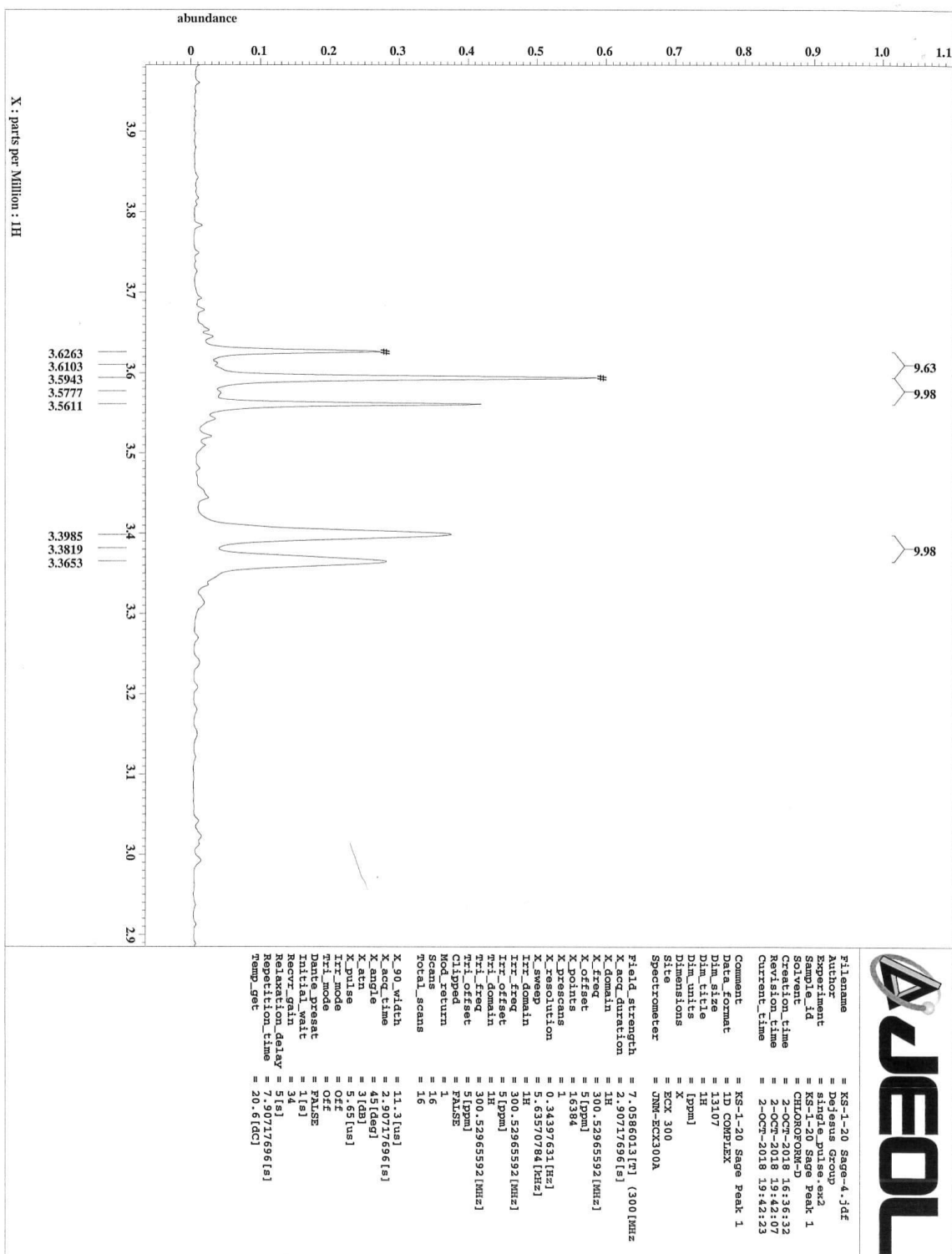

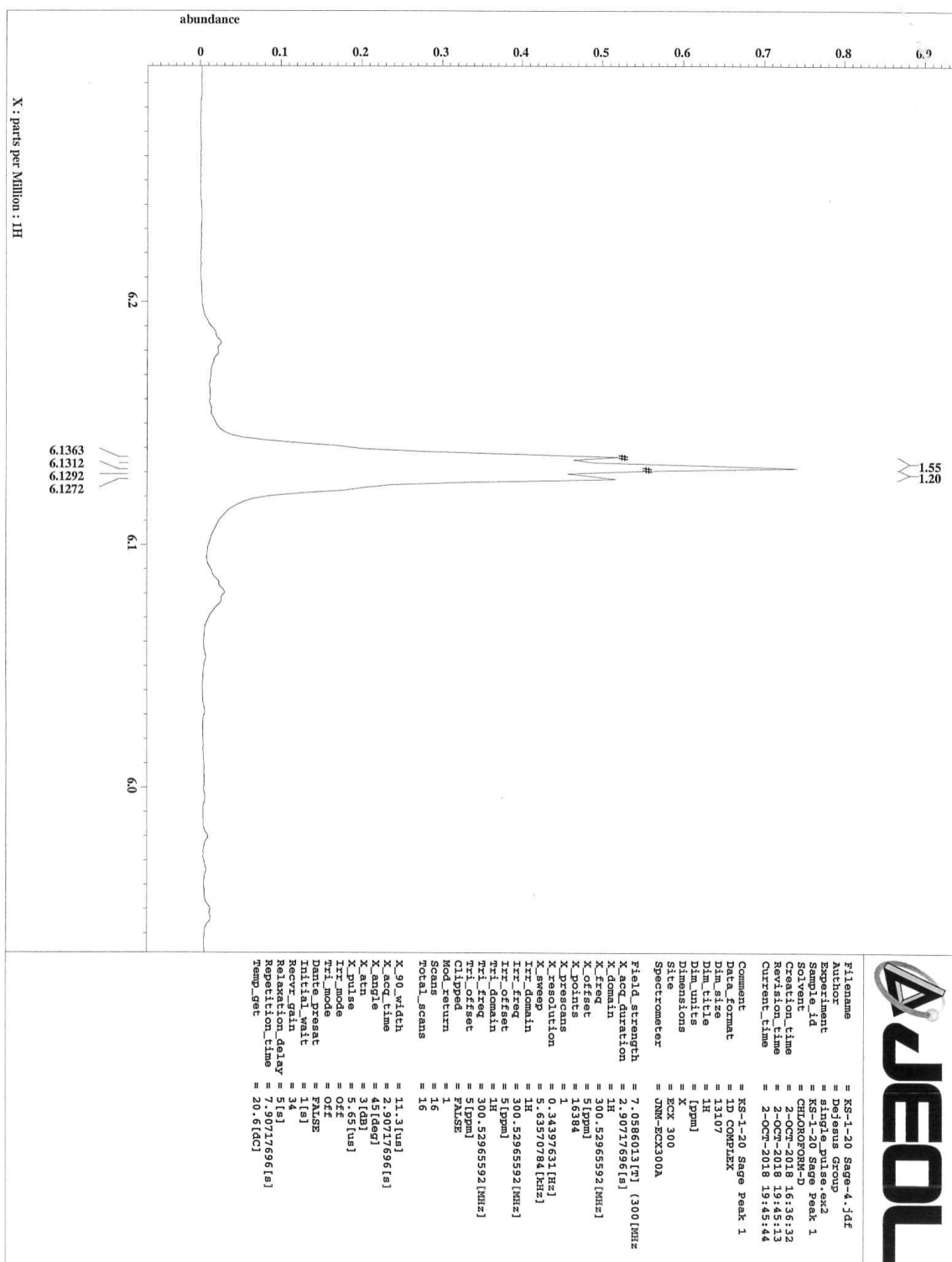

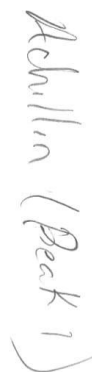

Good coupling constant

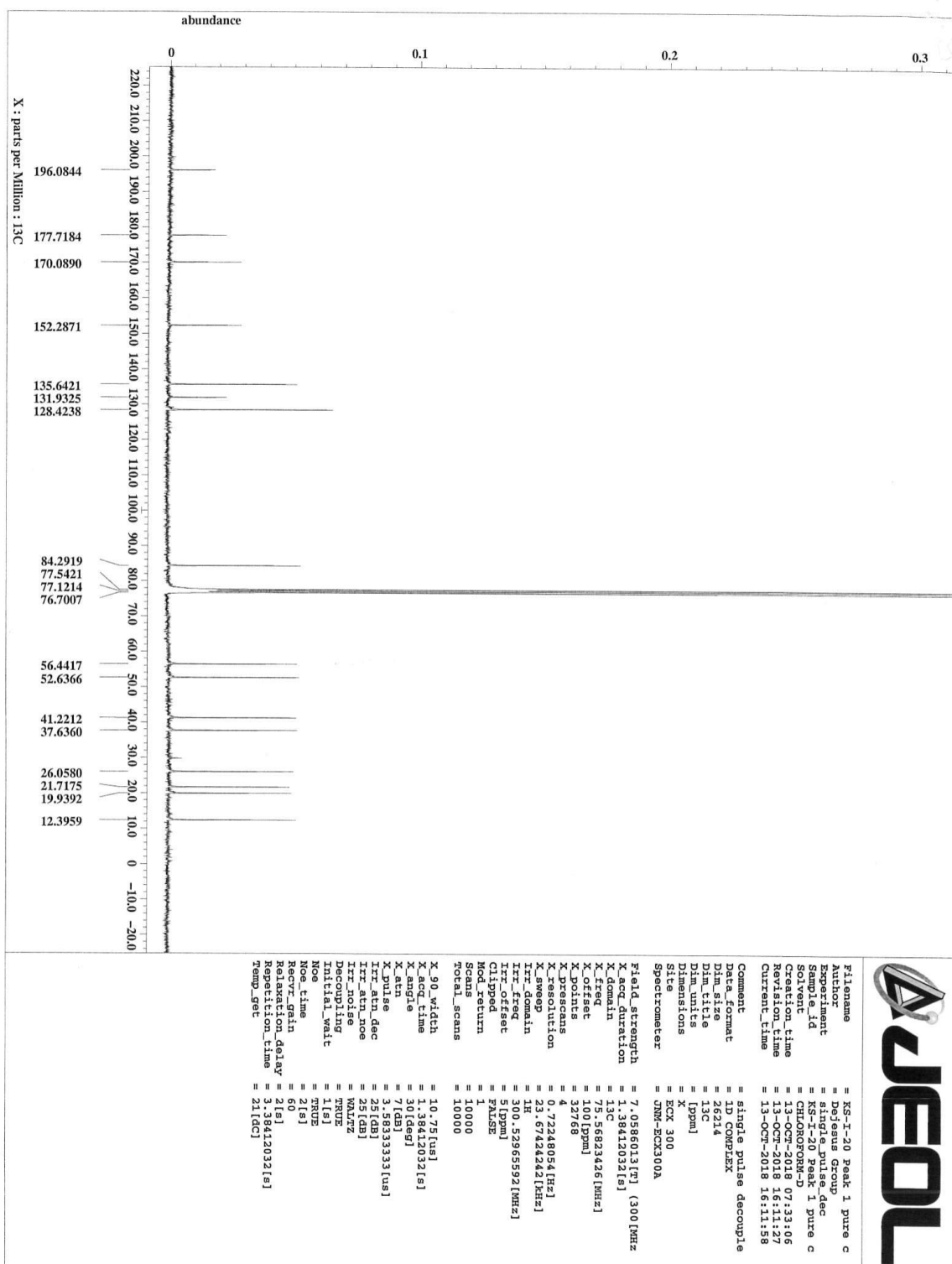

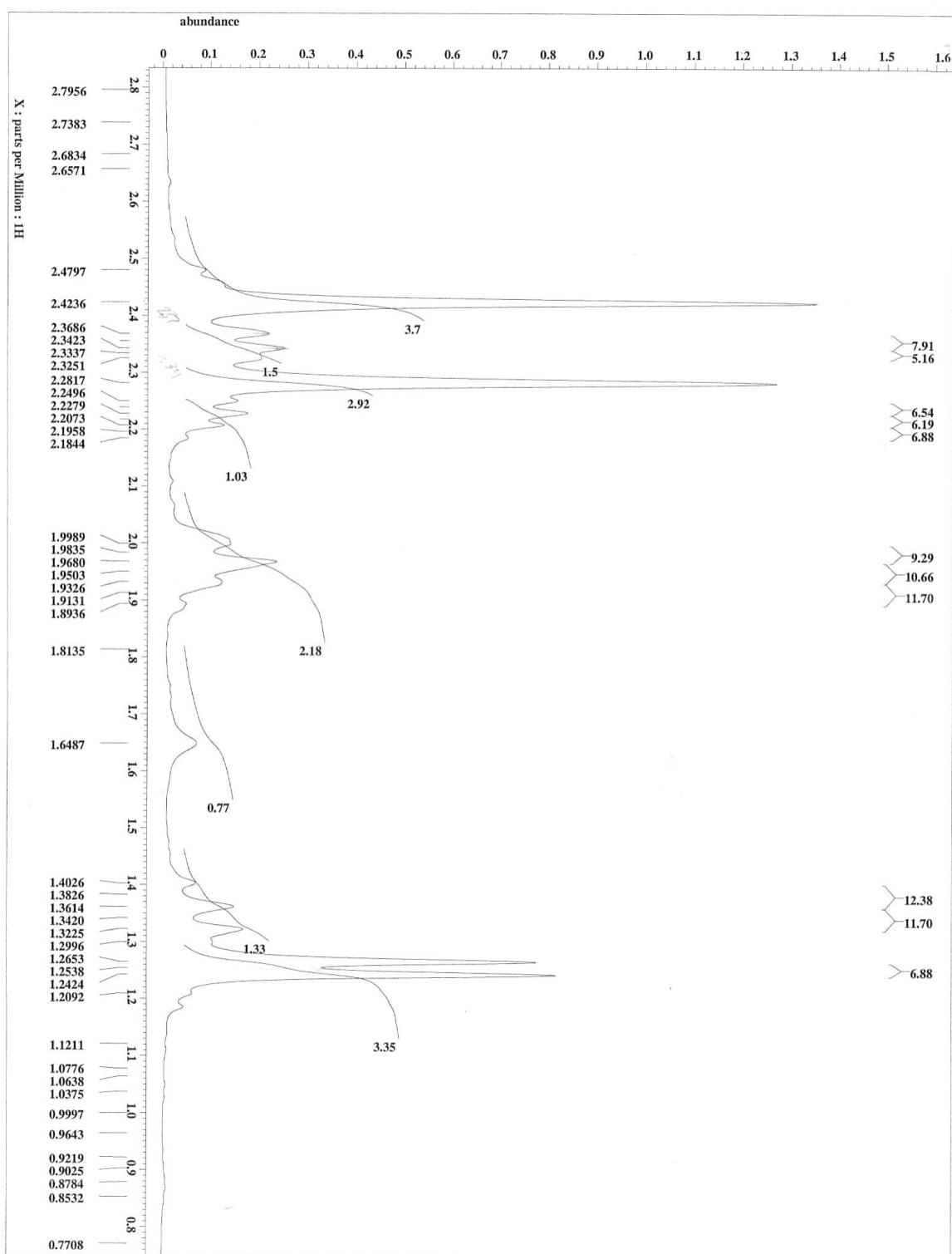

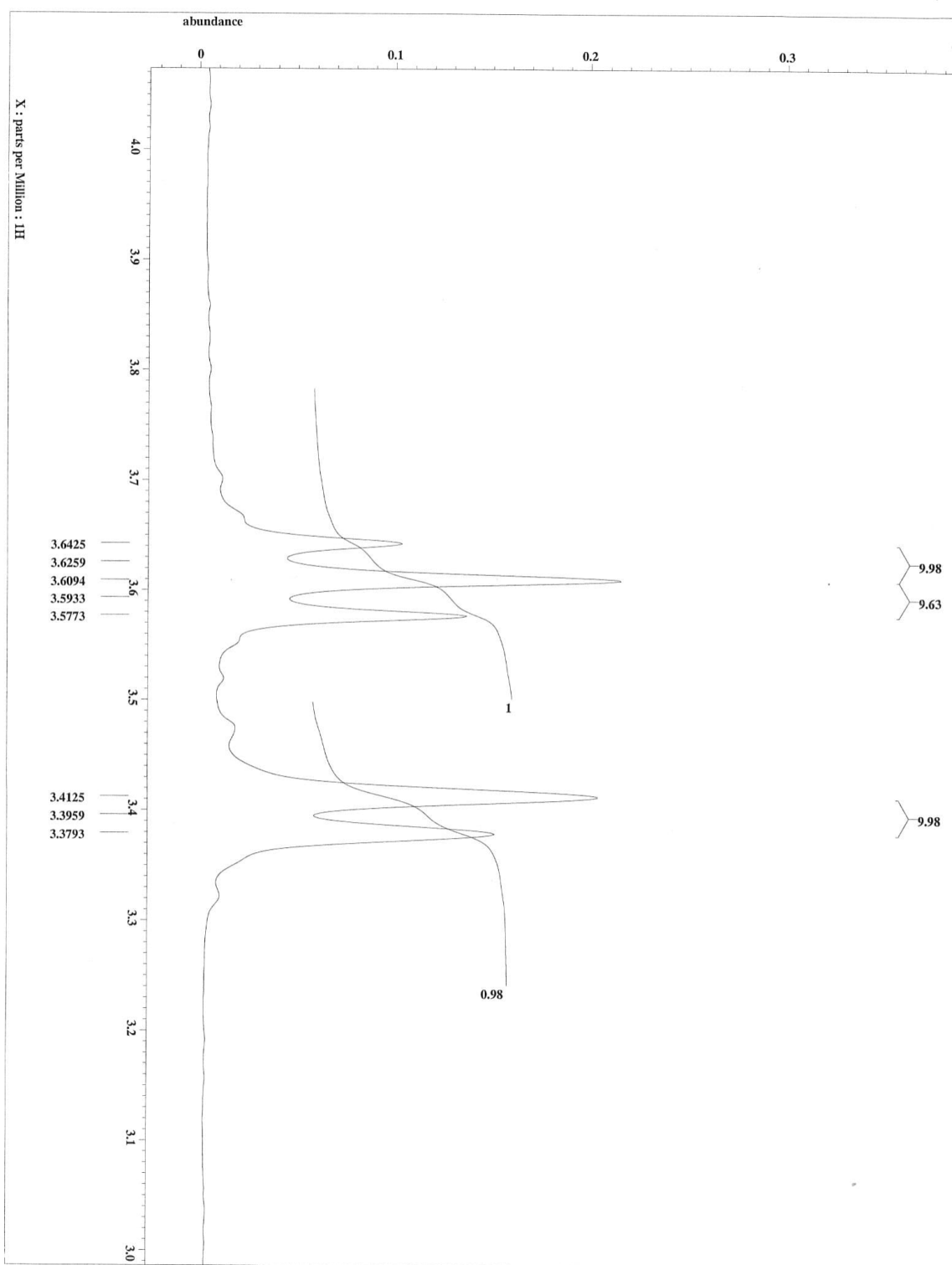

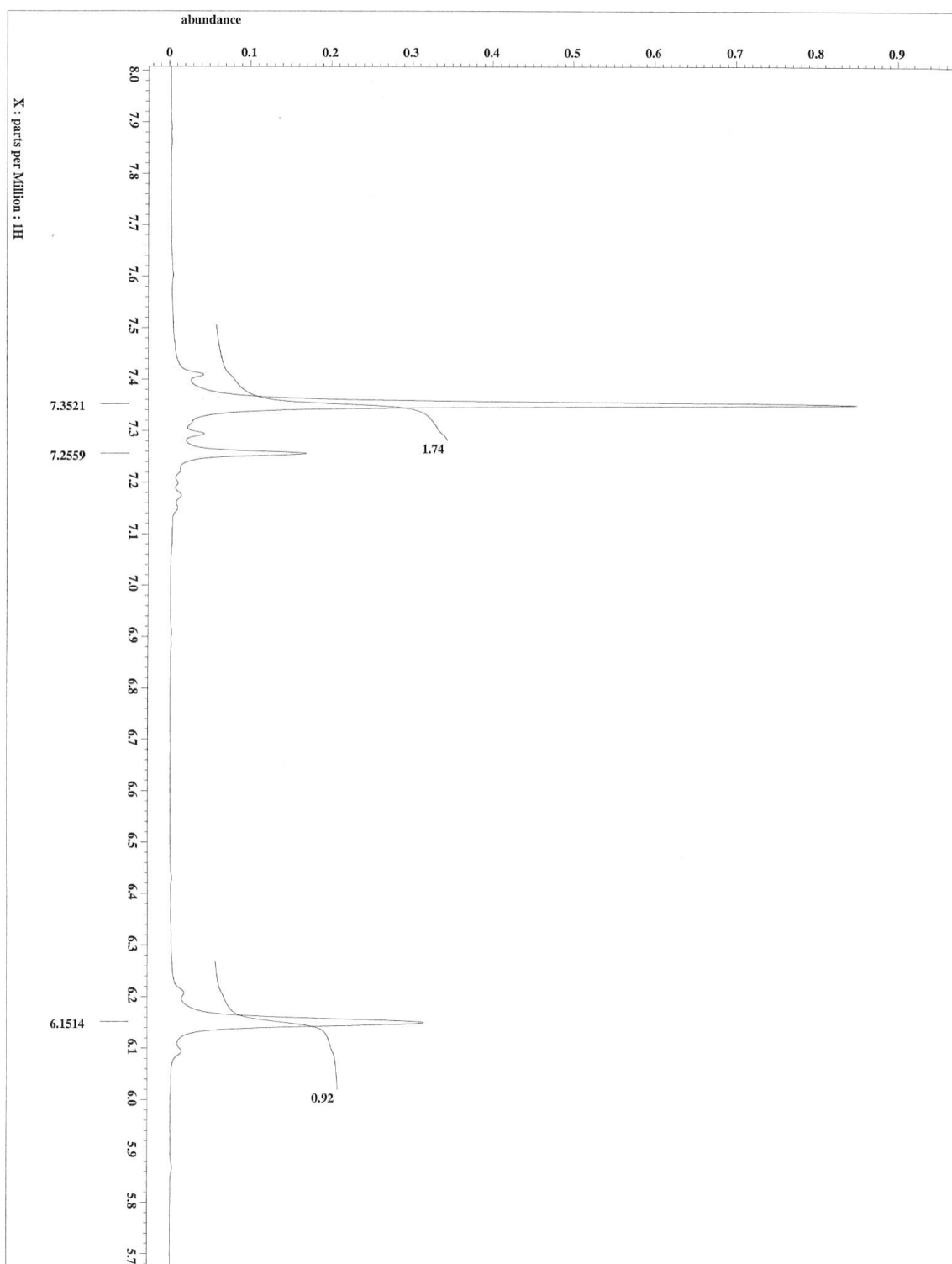

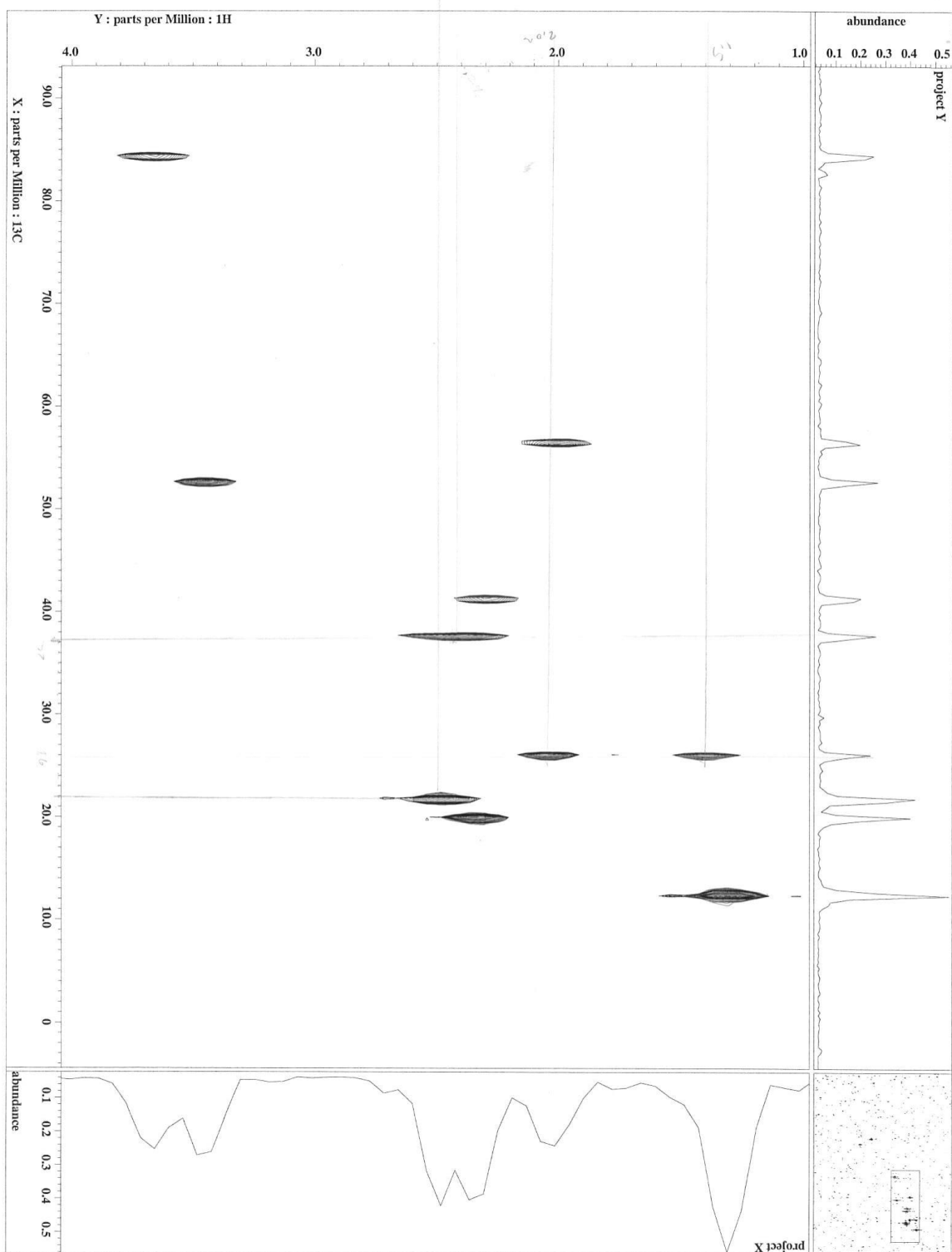

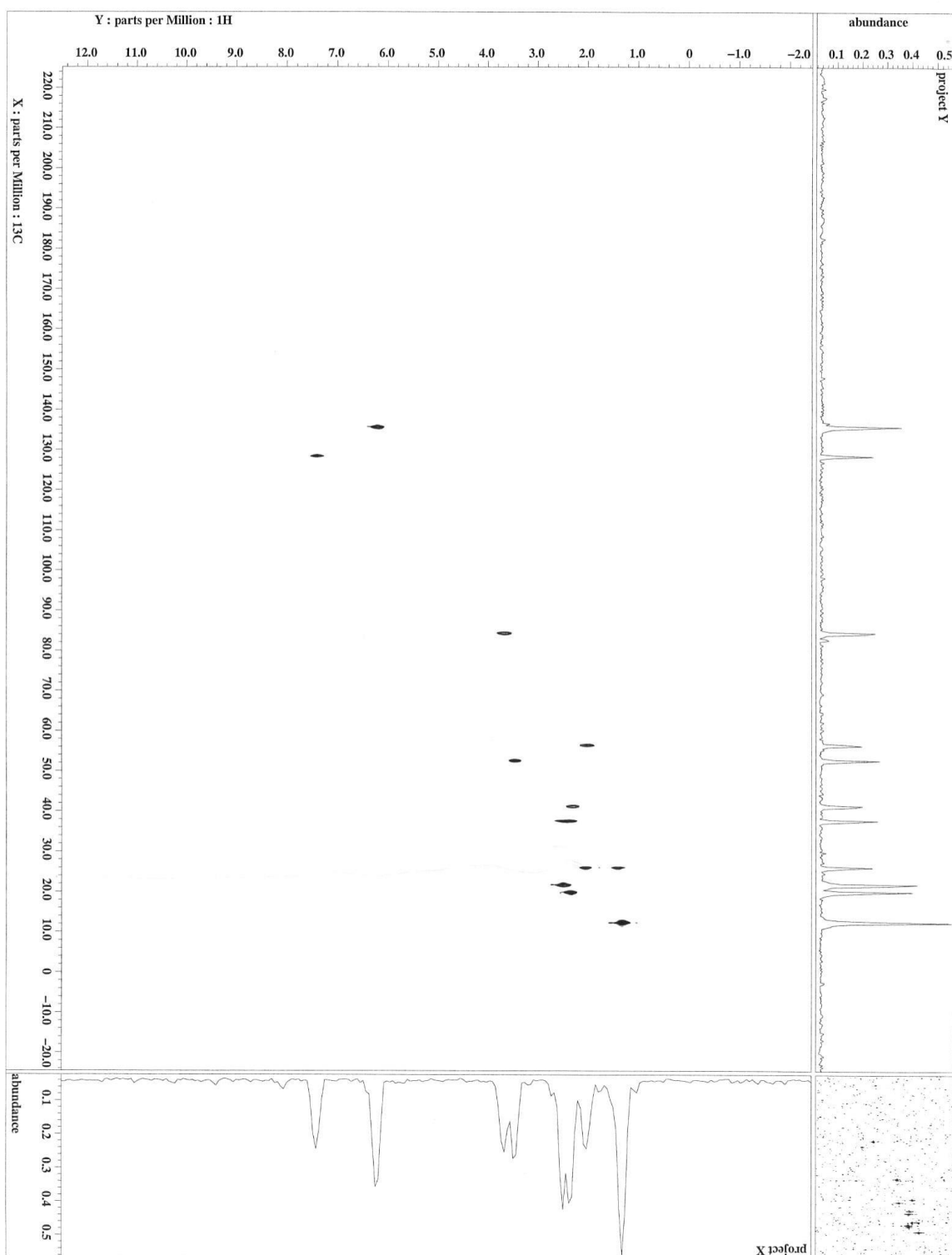

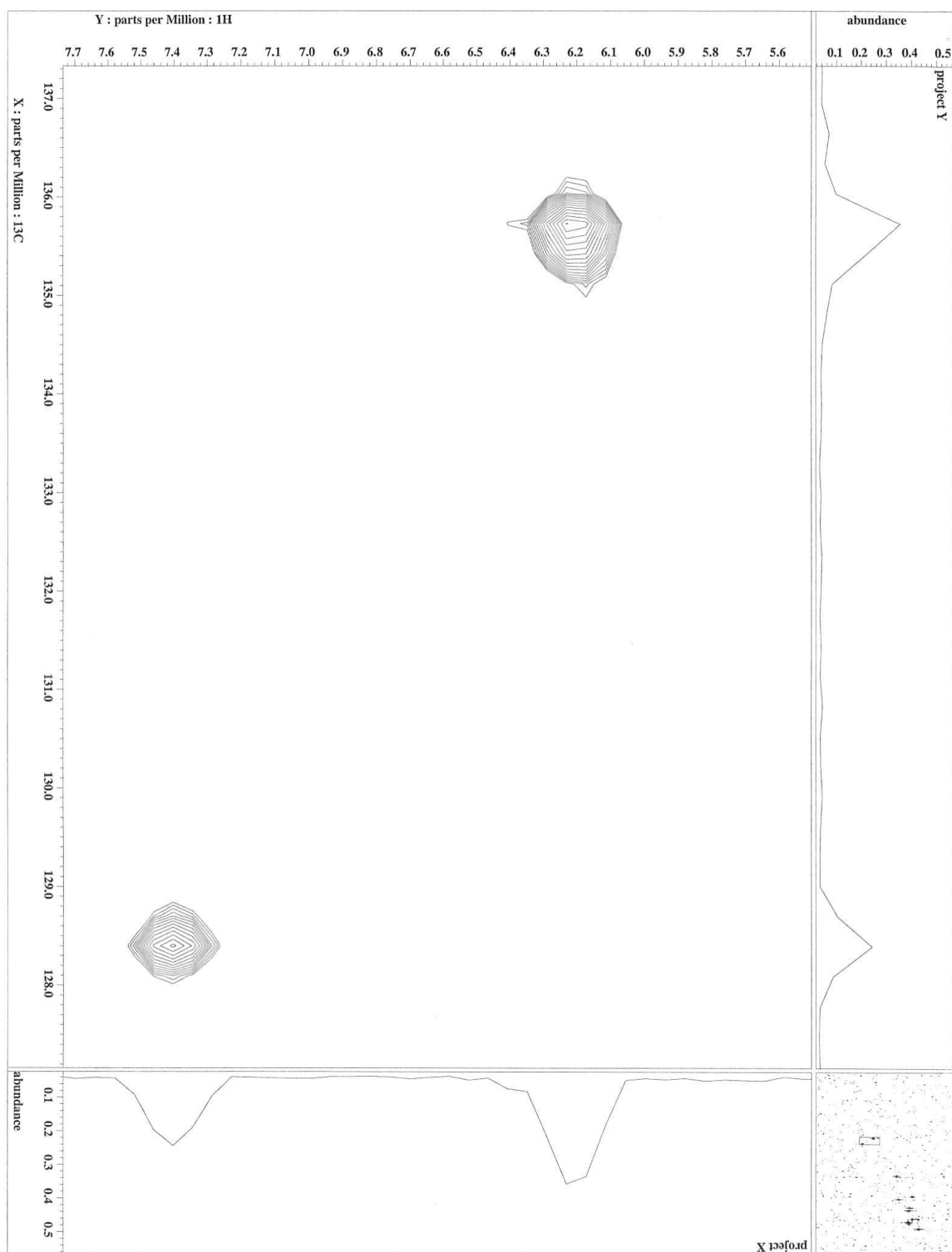

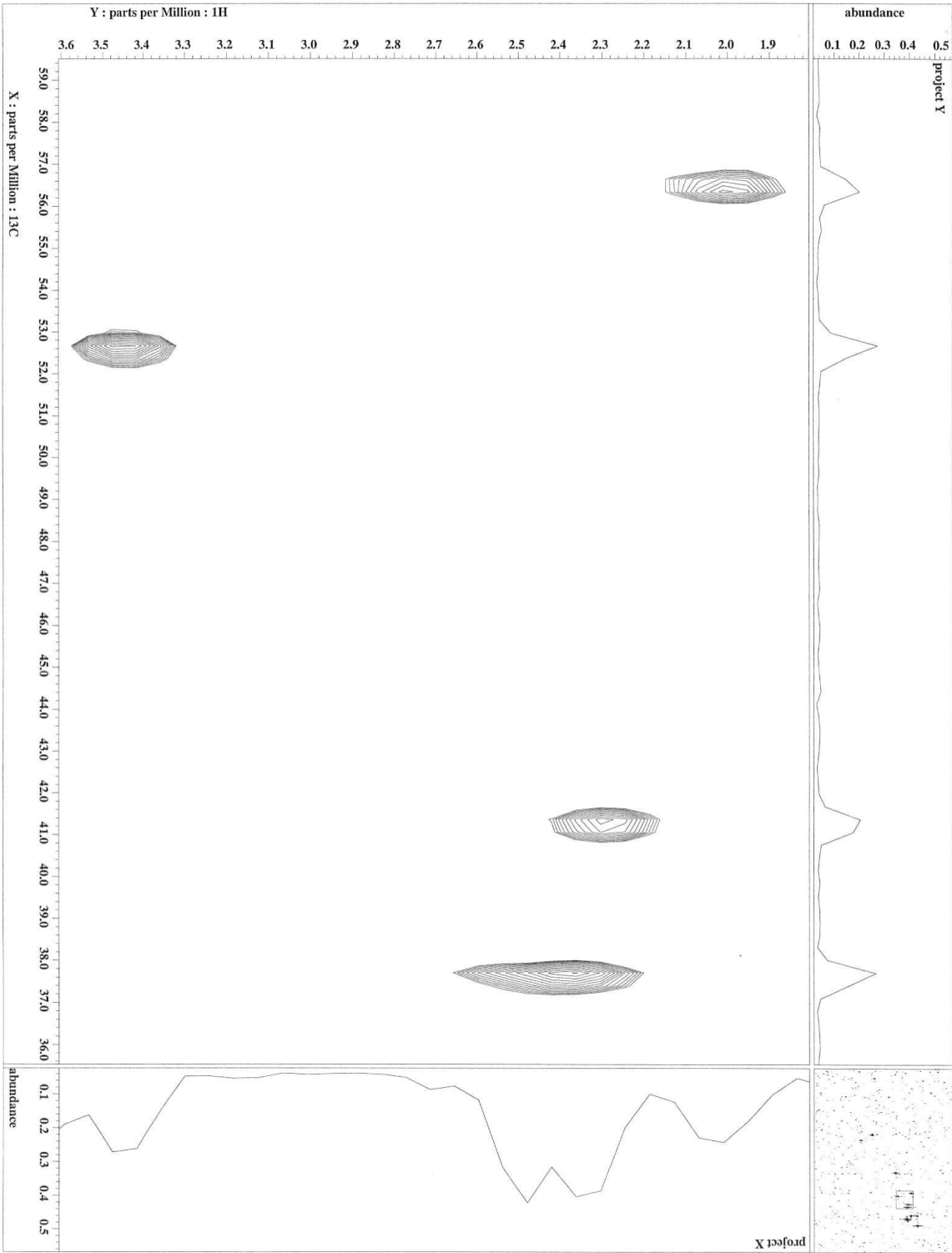

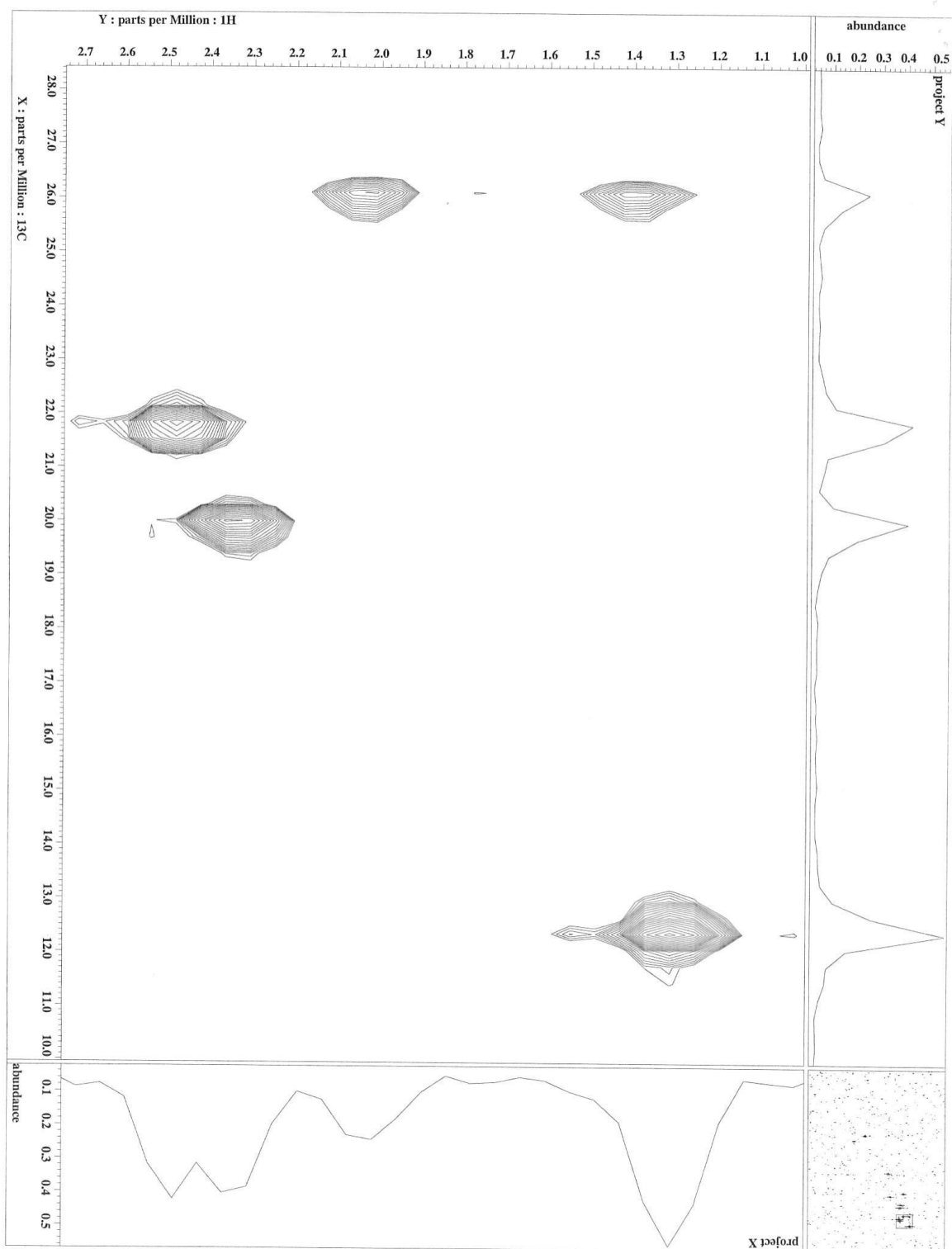

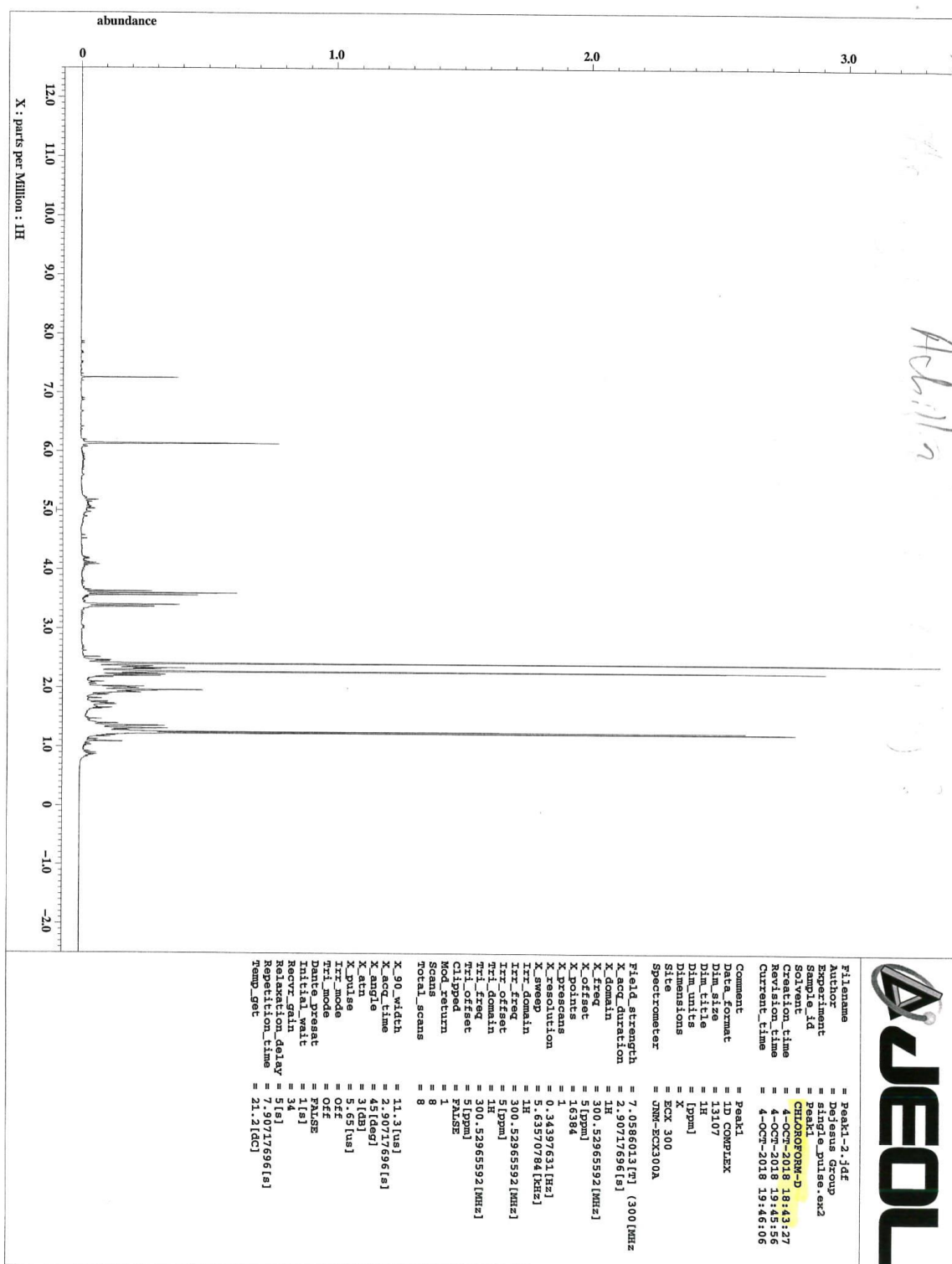

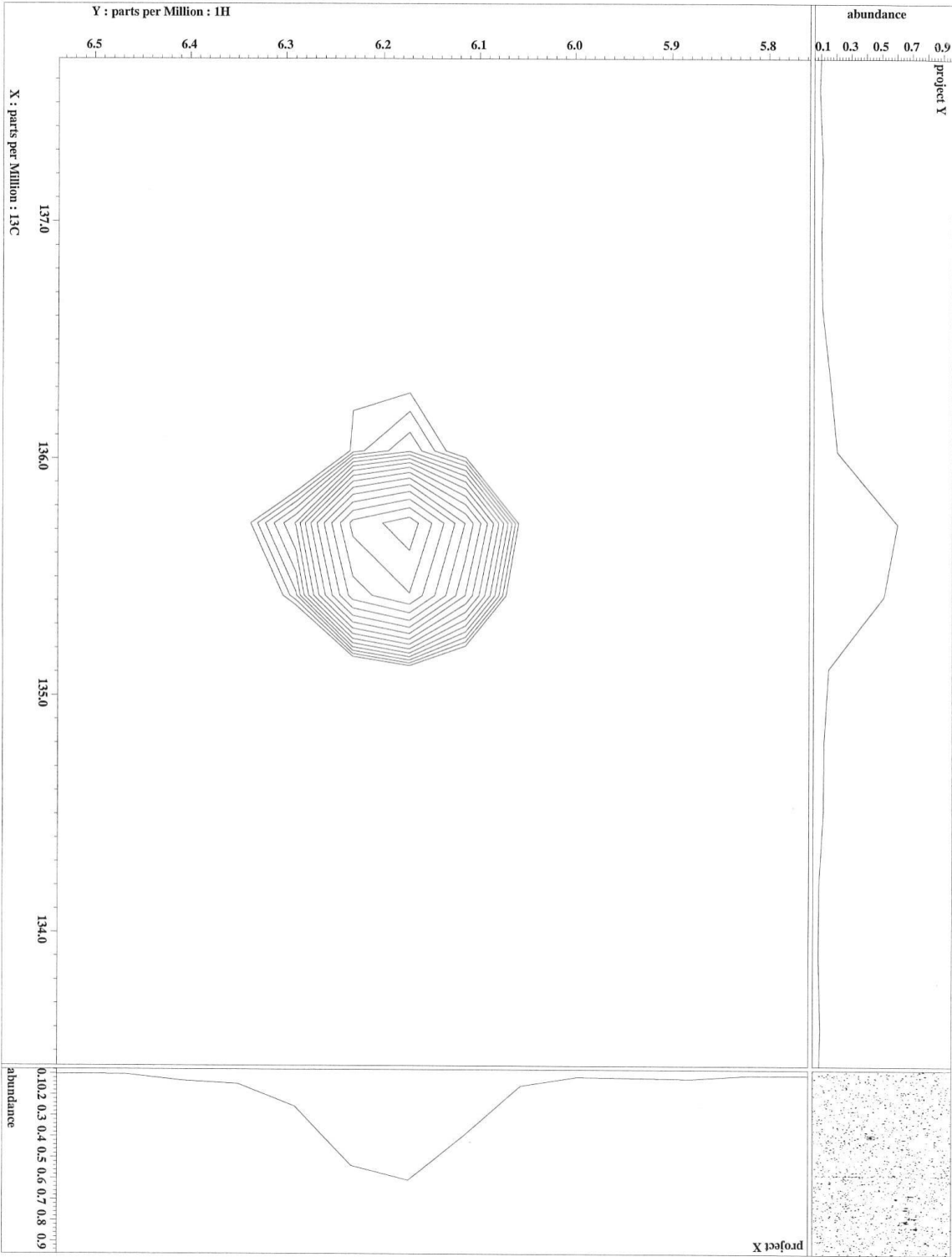

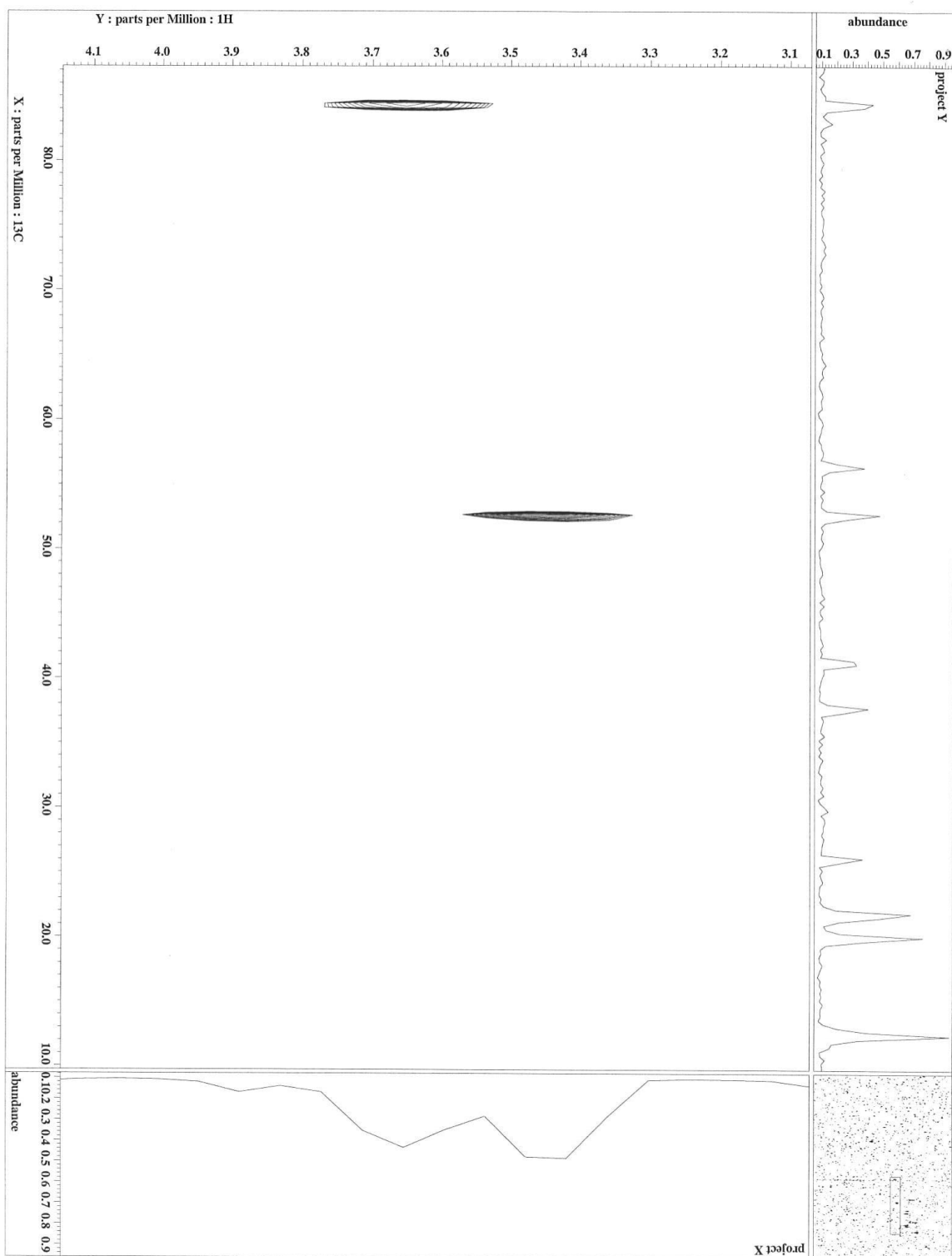

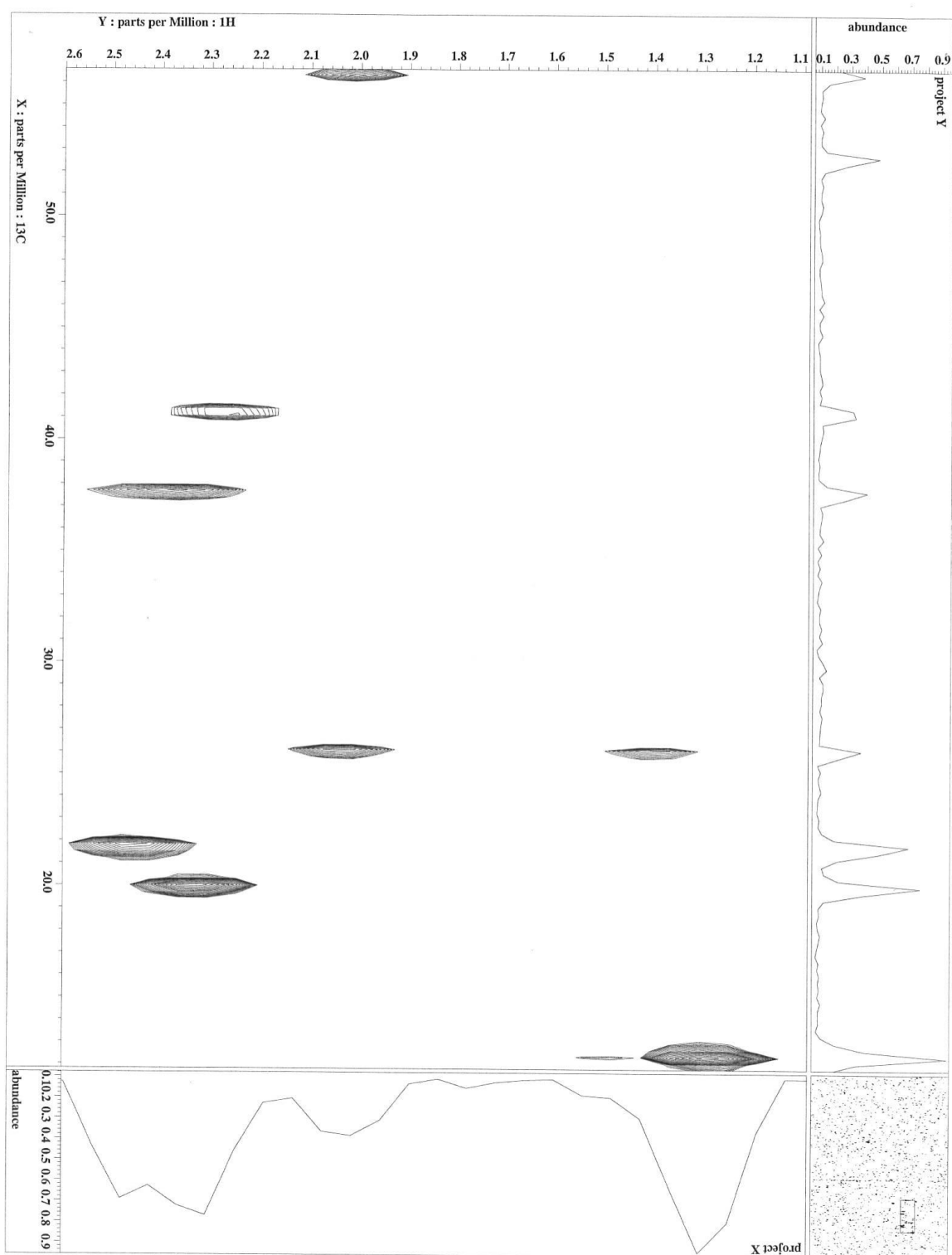

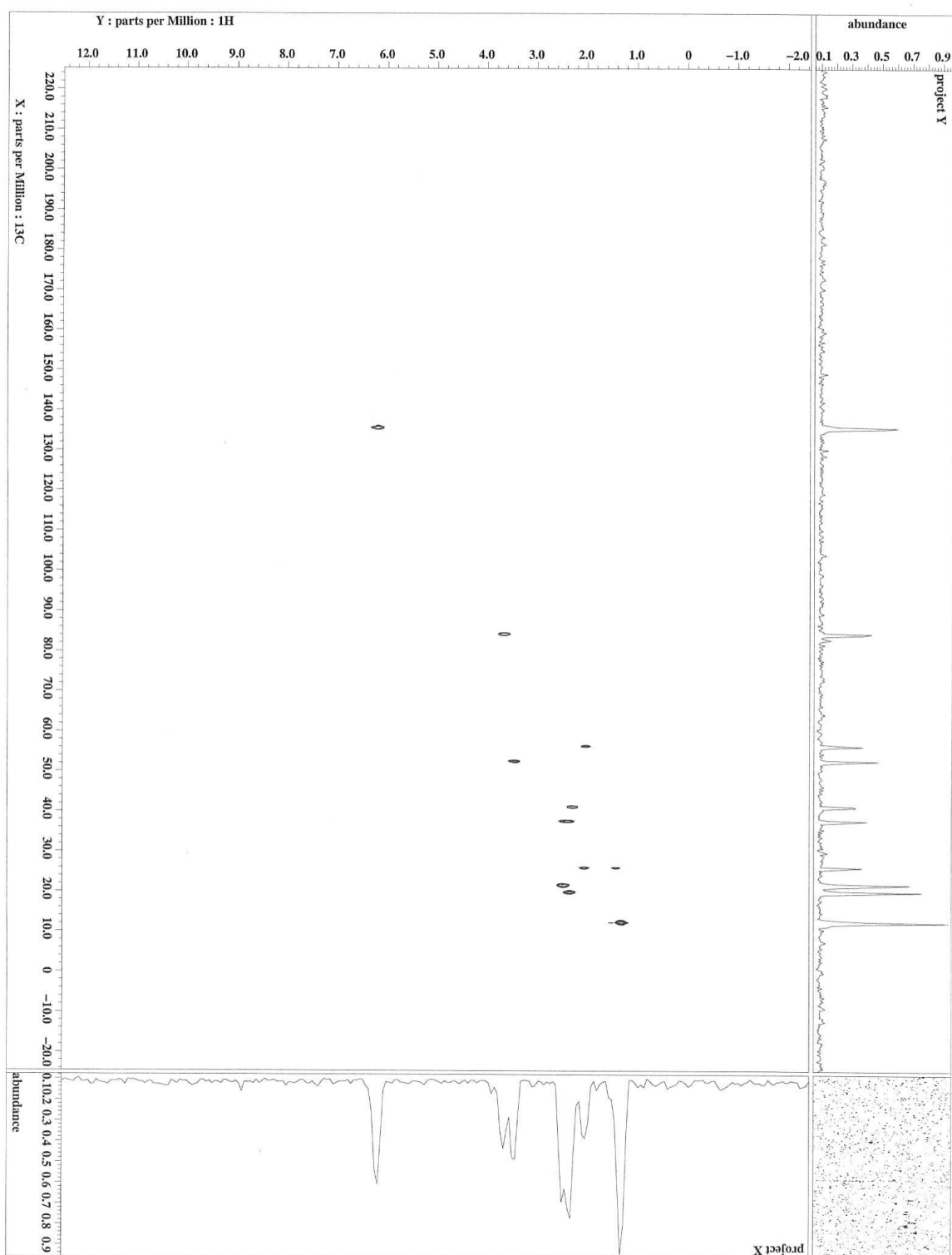

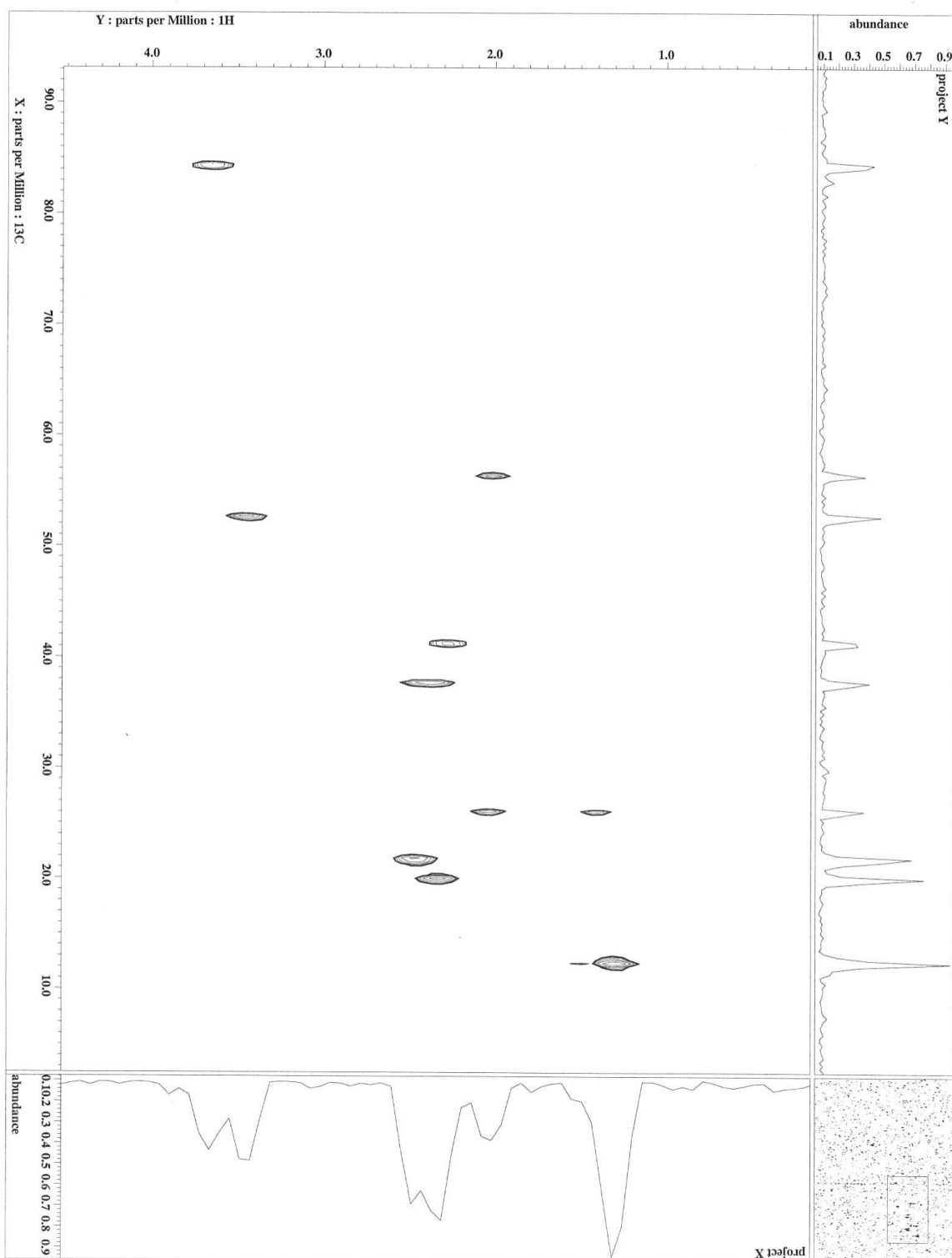

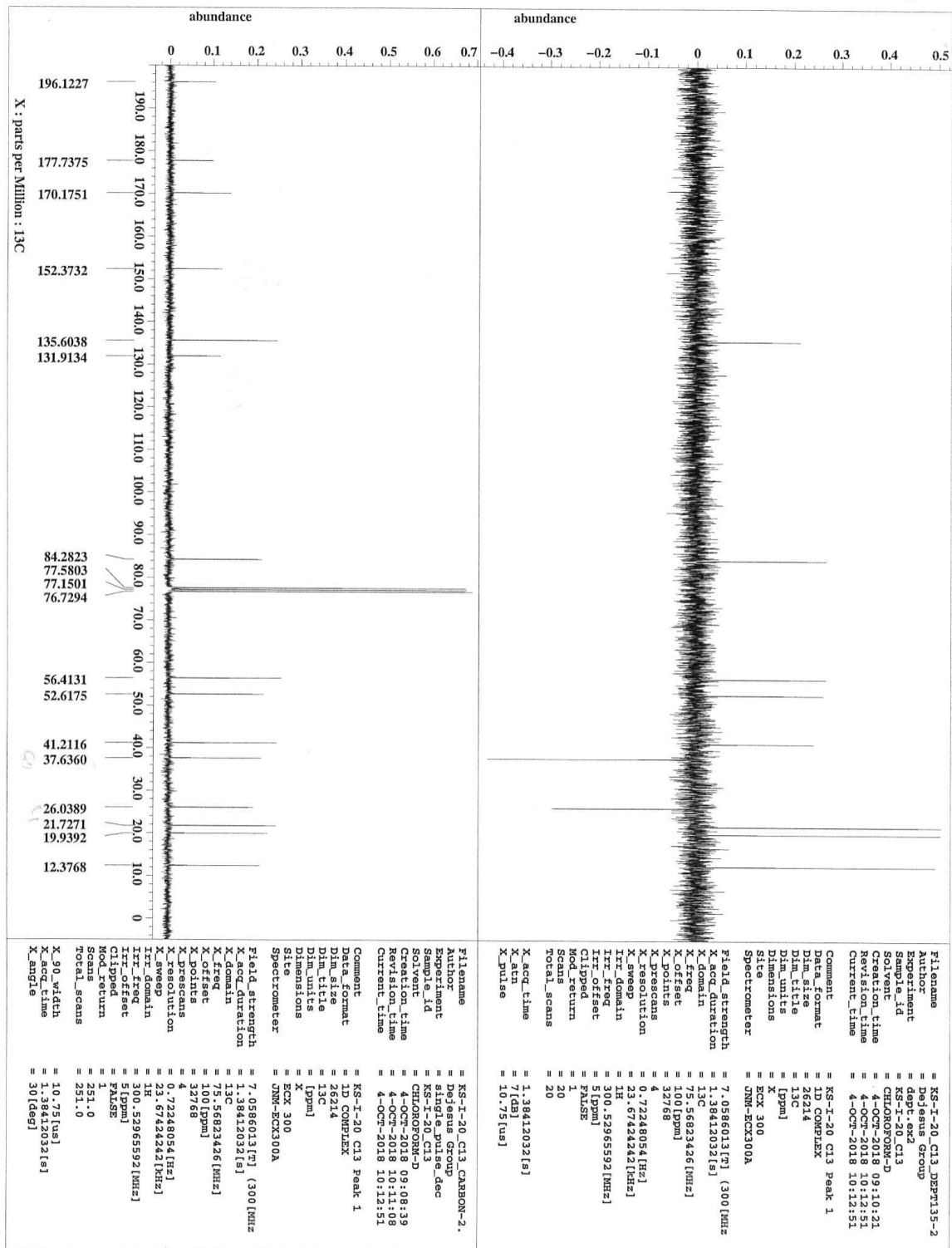

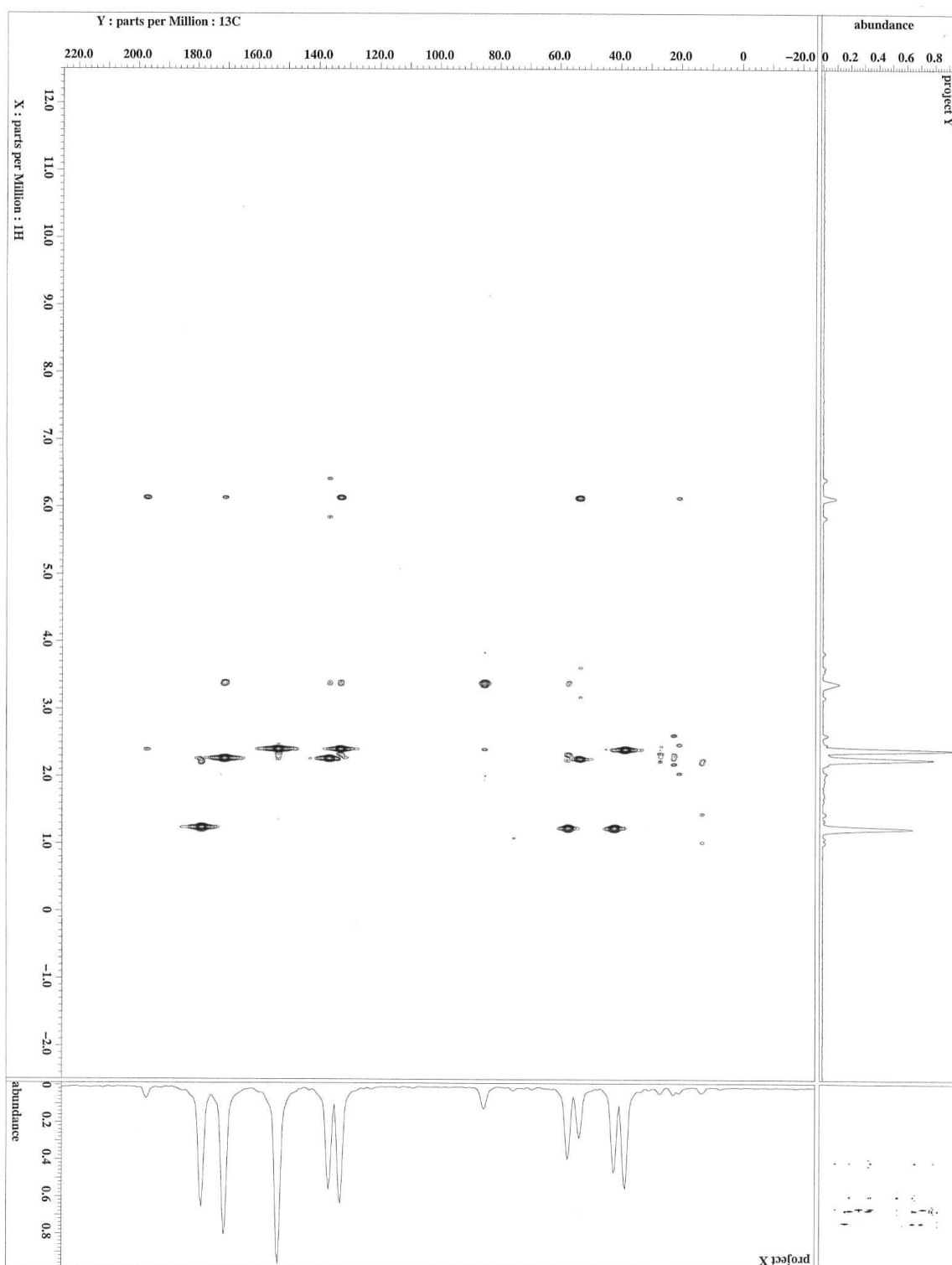

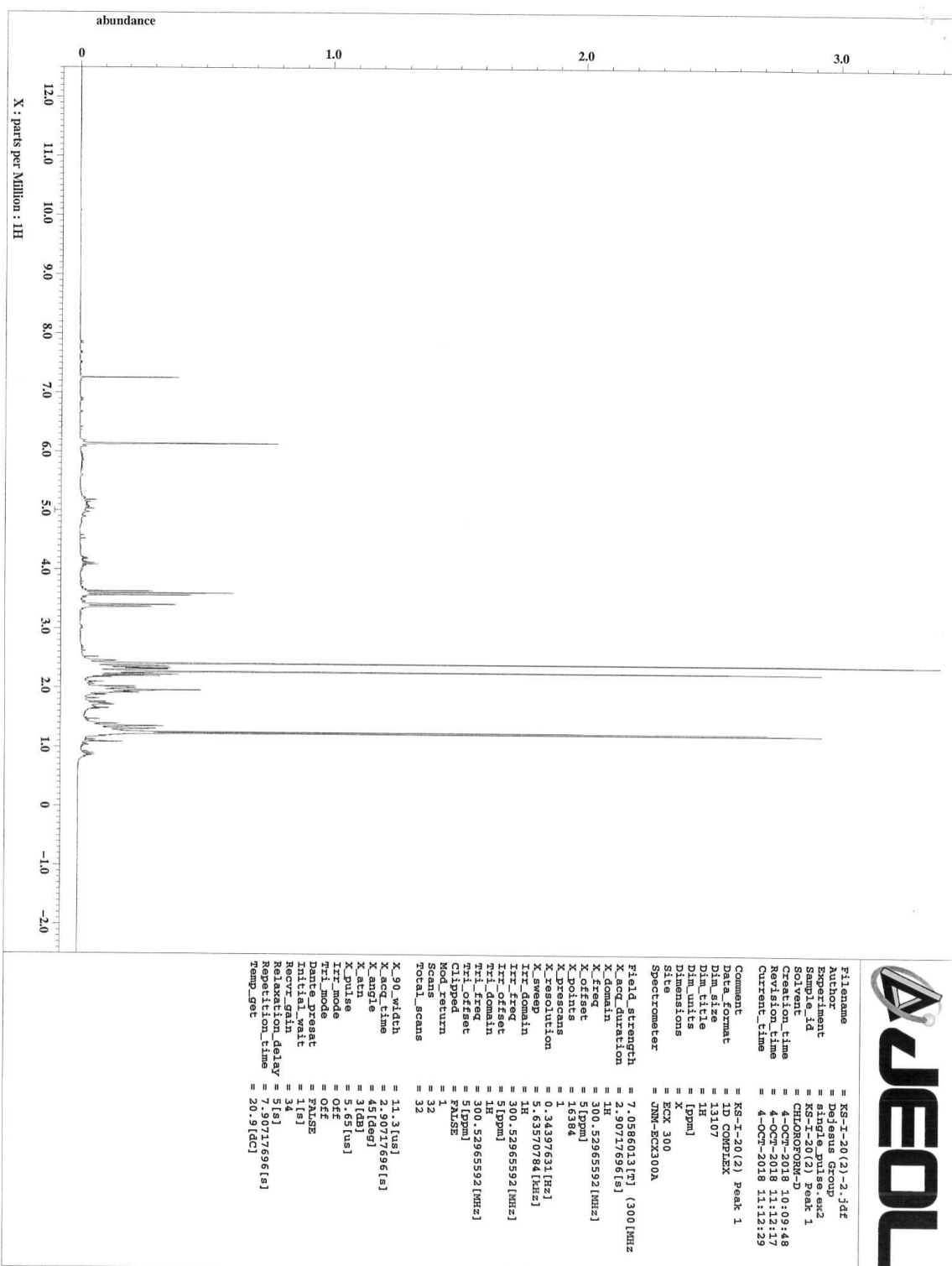

Supplement: Supplementary file 1 [file molecules-29-00802-s001.zip › molecules-2800749-supplementary/Supplementary Data/Supplementary Data-4a-NMR-Scanned Files/Leukodin NMR.pdf]

## 6.2 Acetoxychillin NMR Spectra

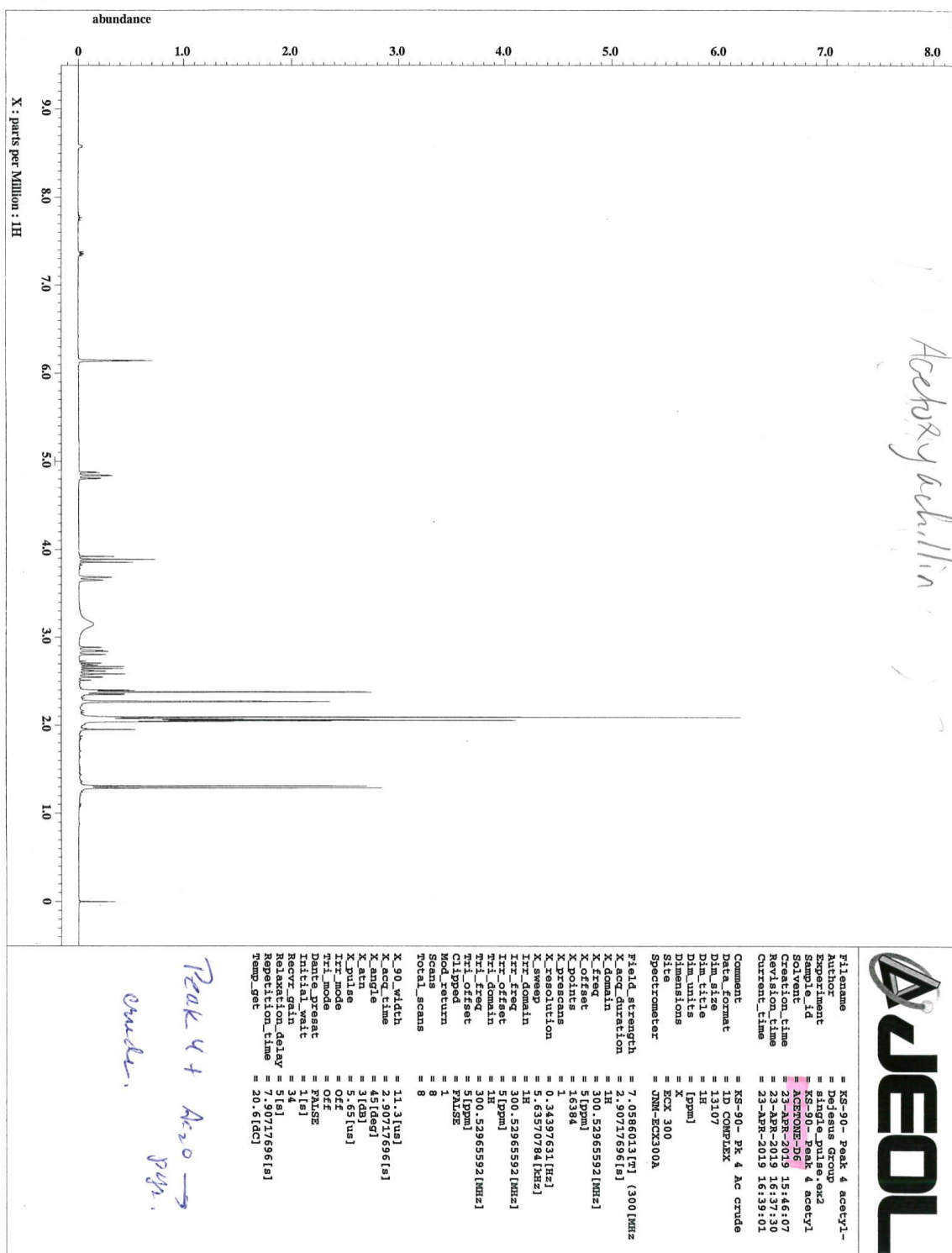

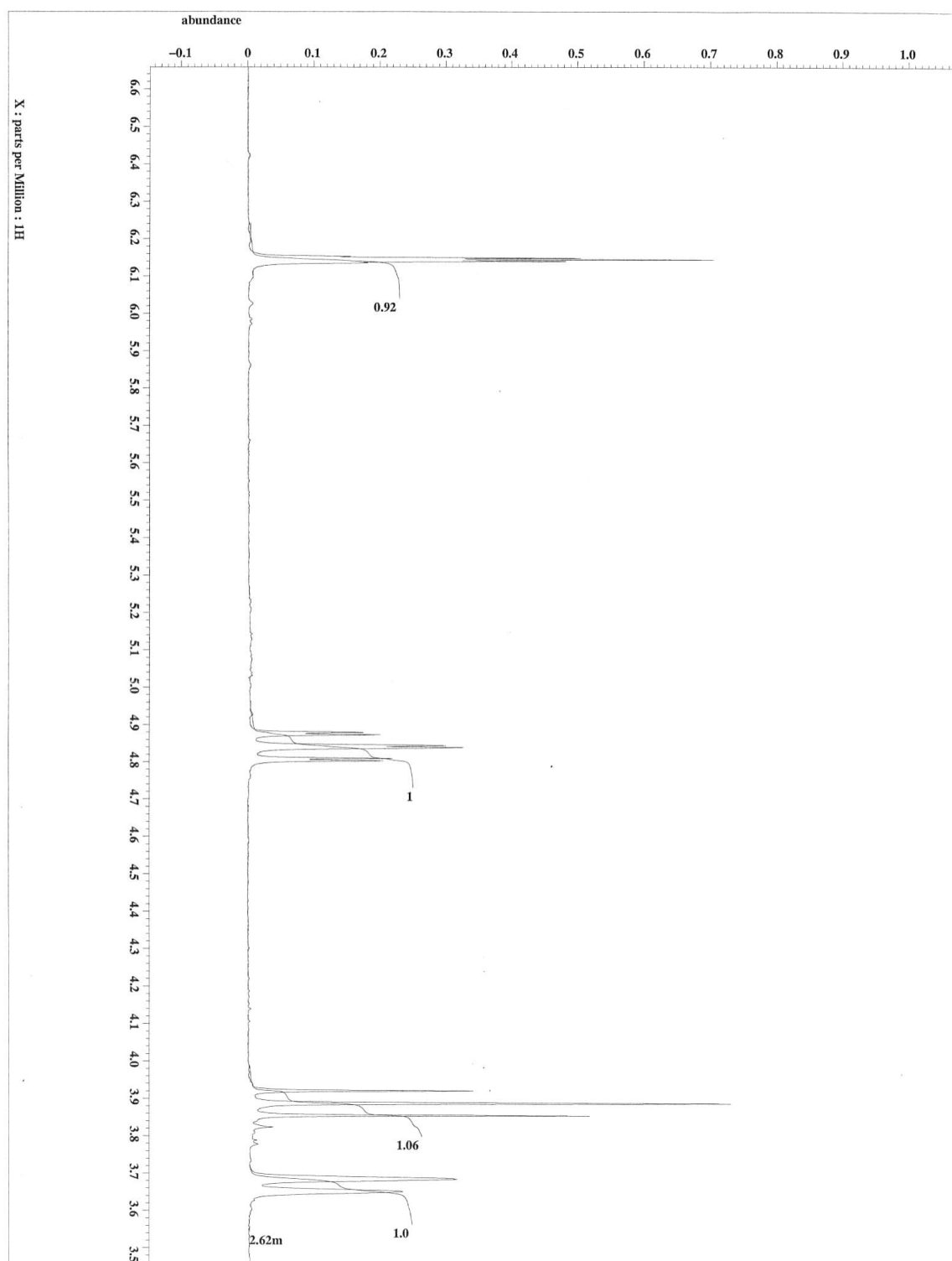

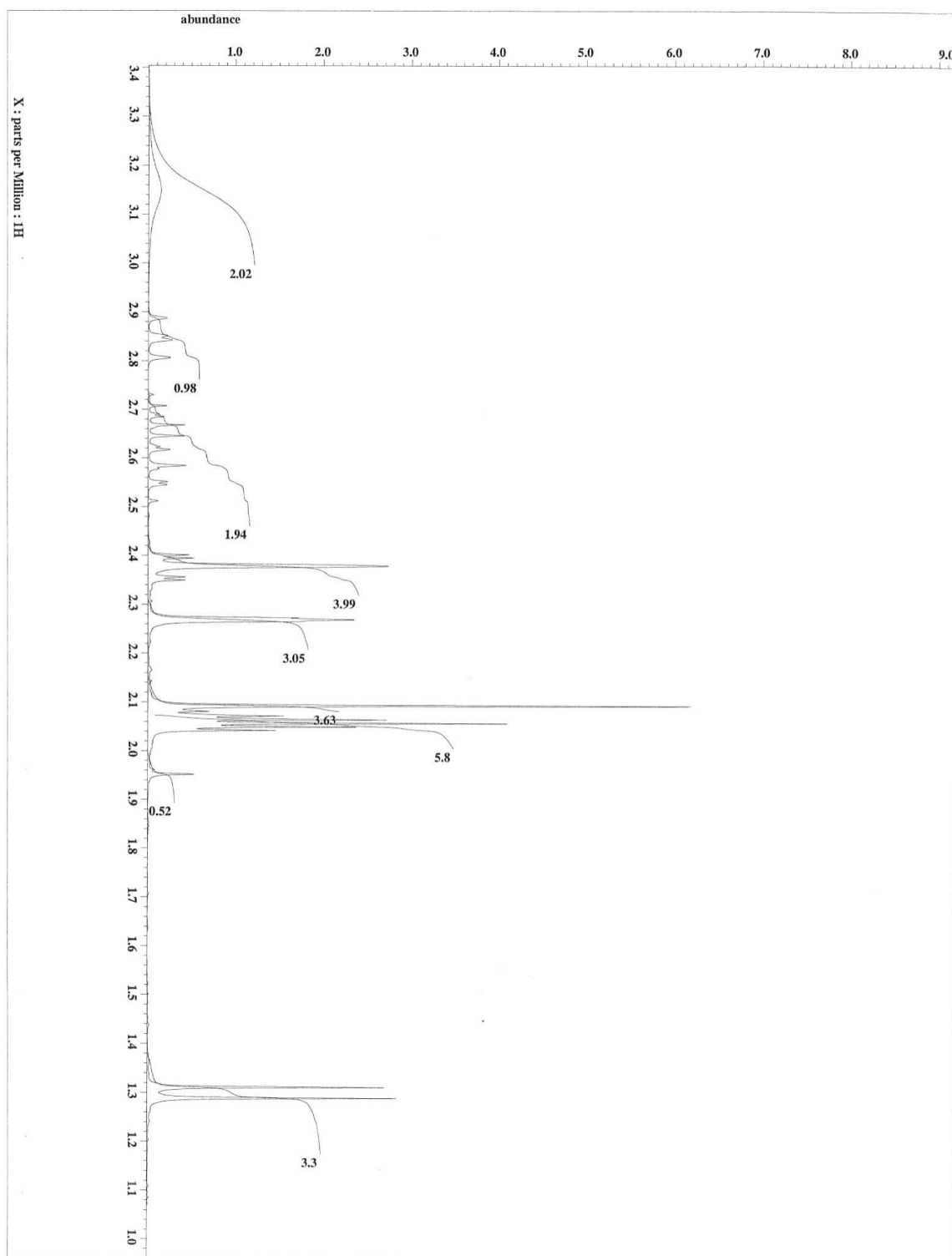

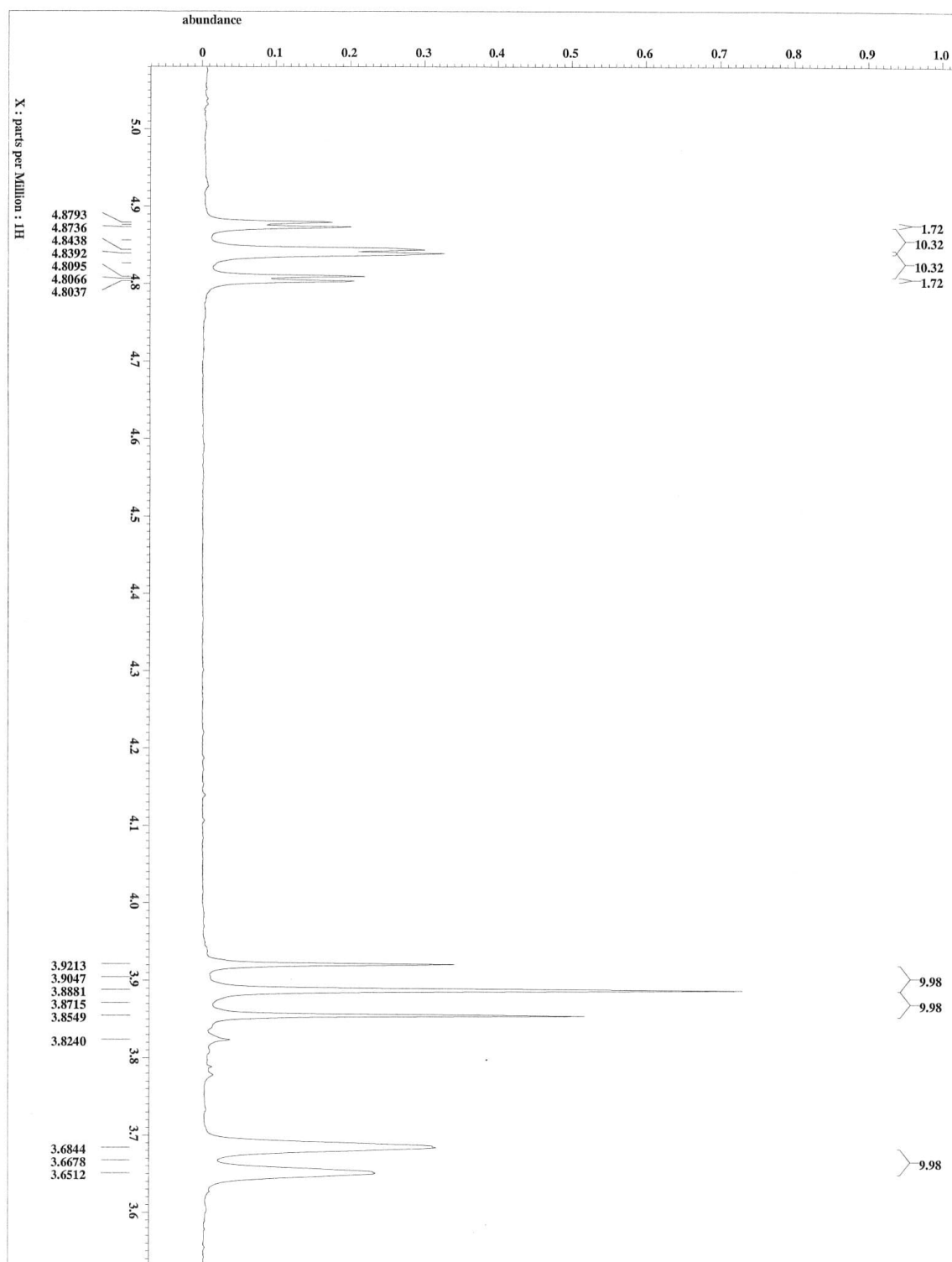

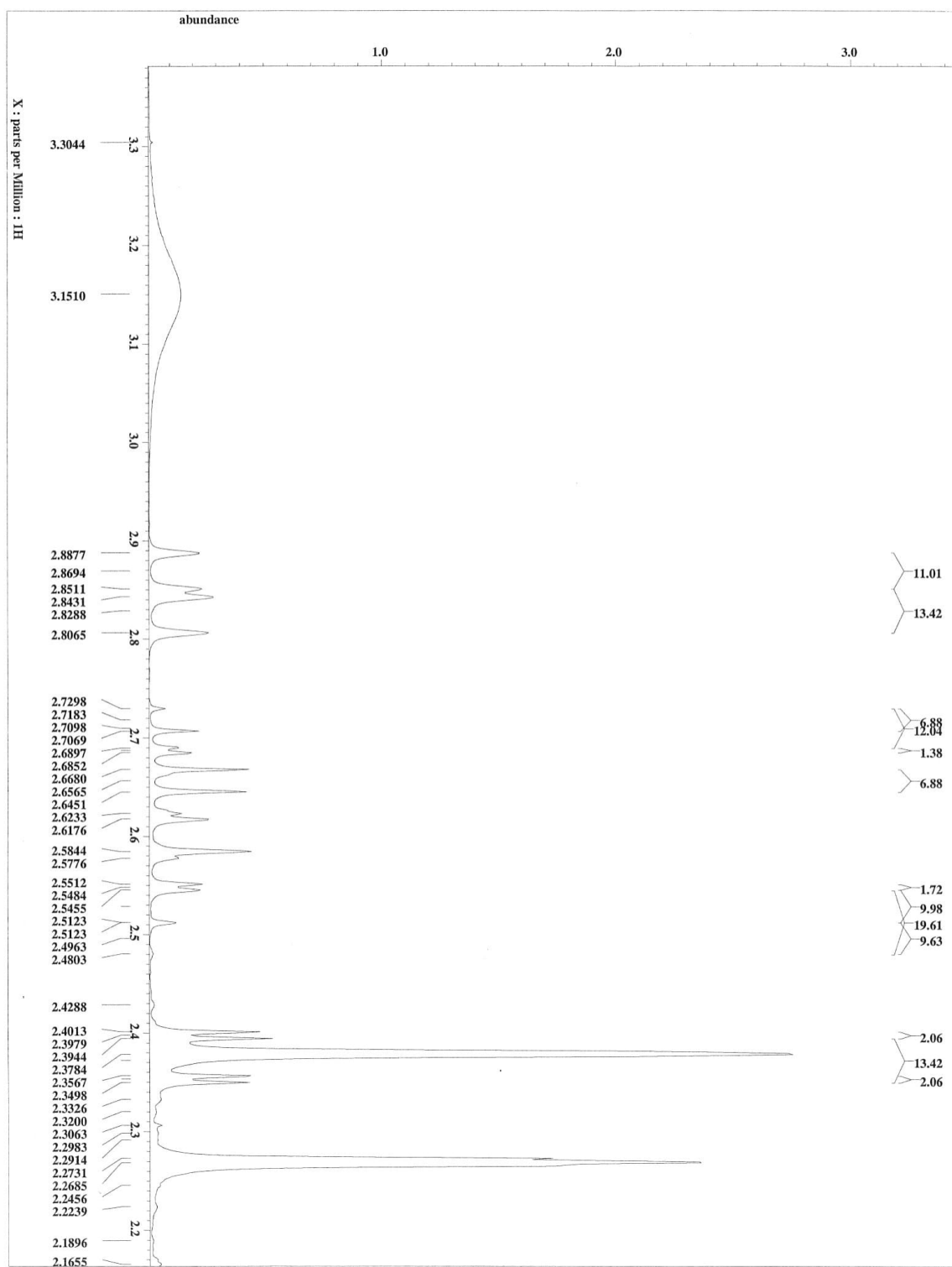

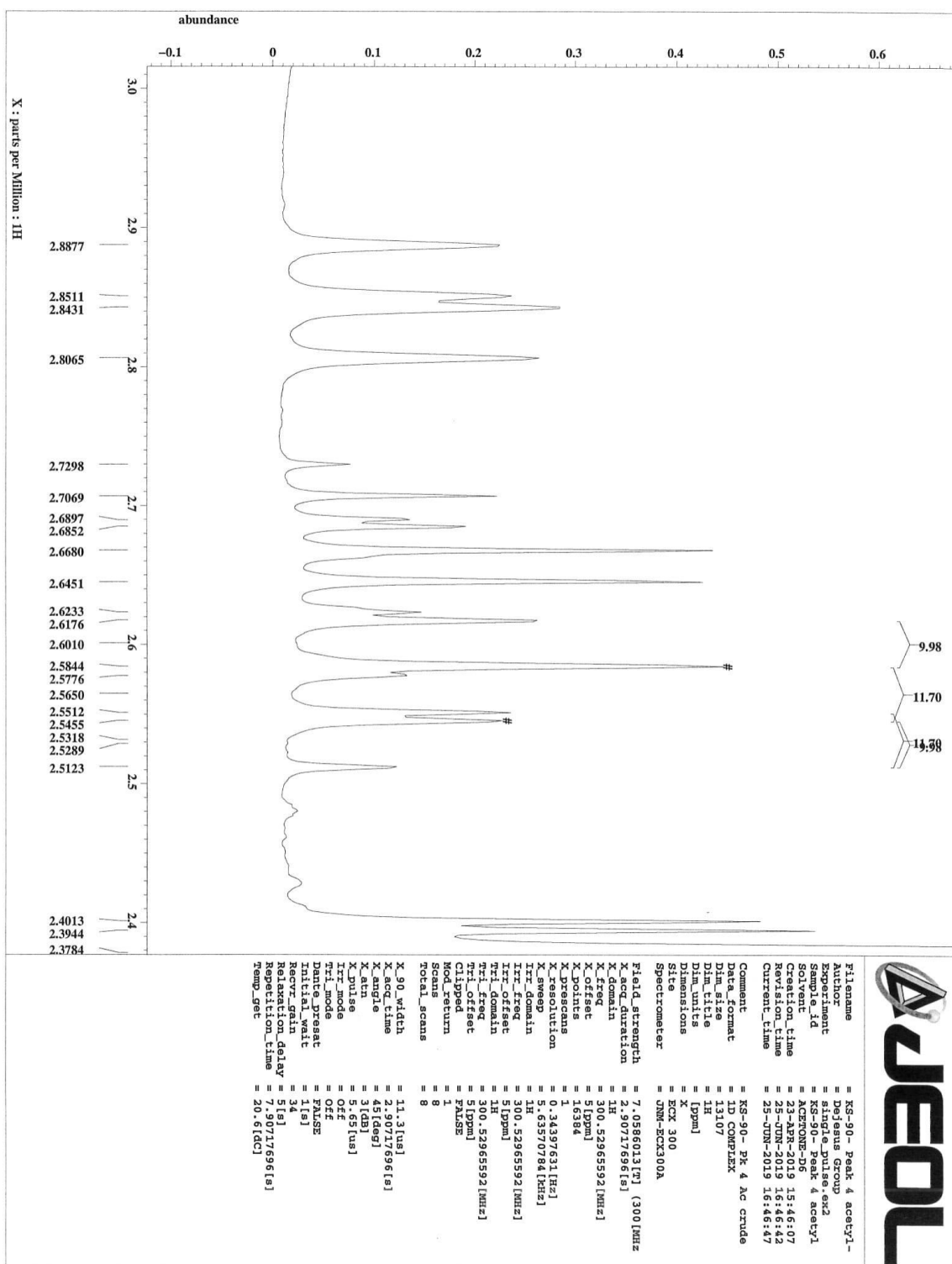

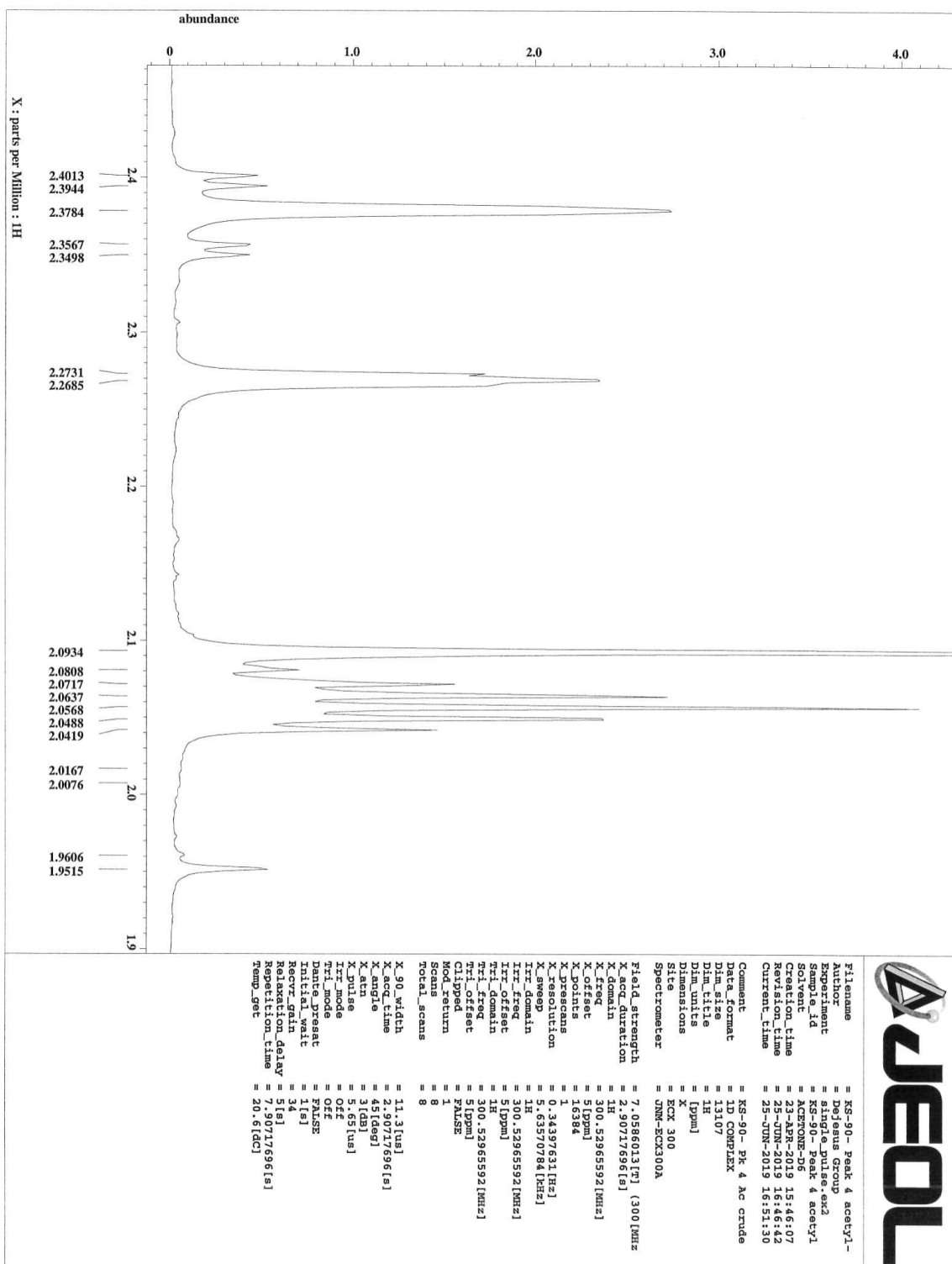

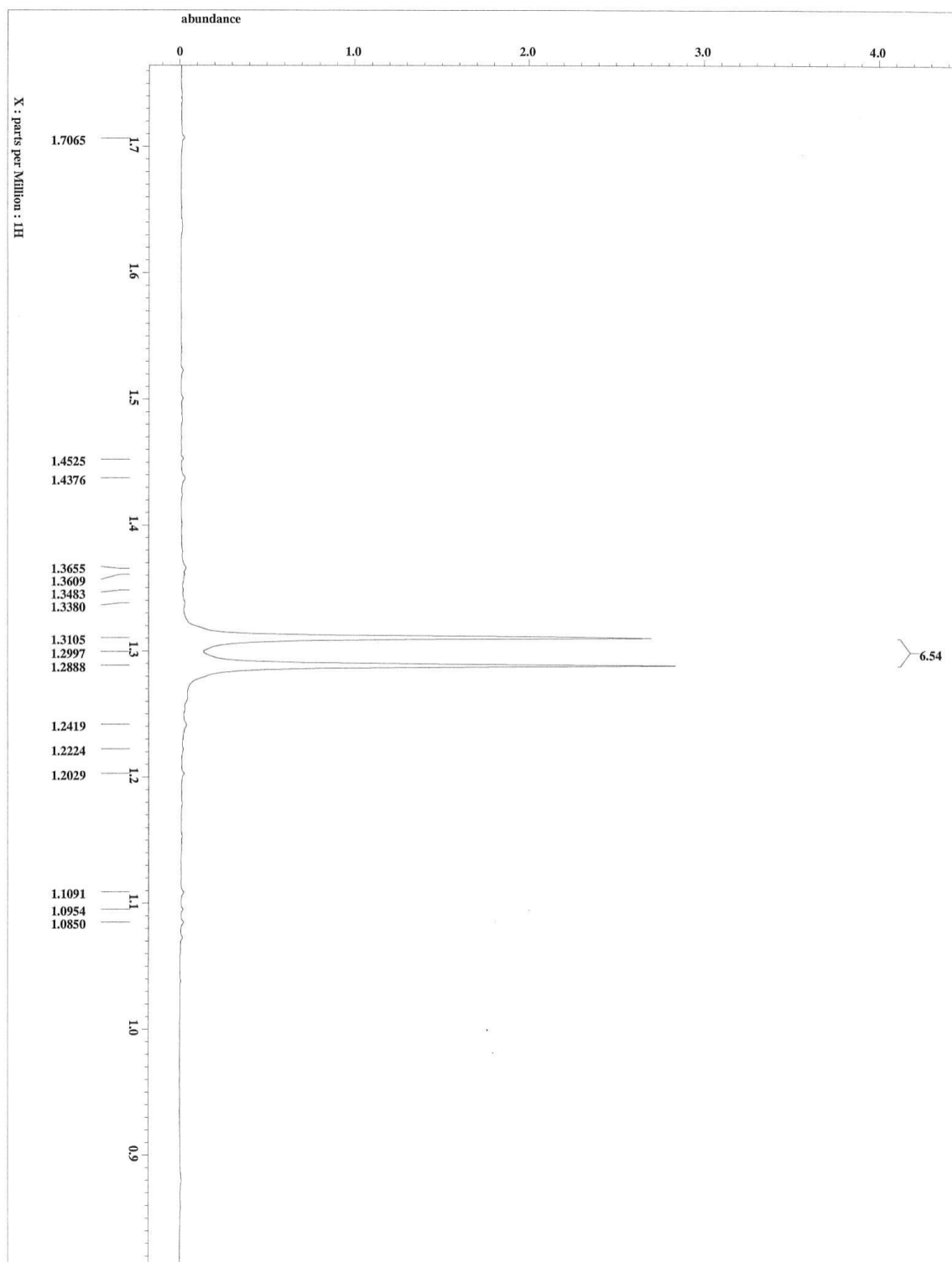

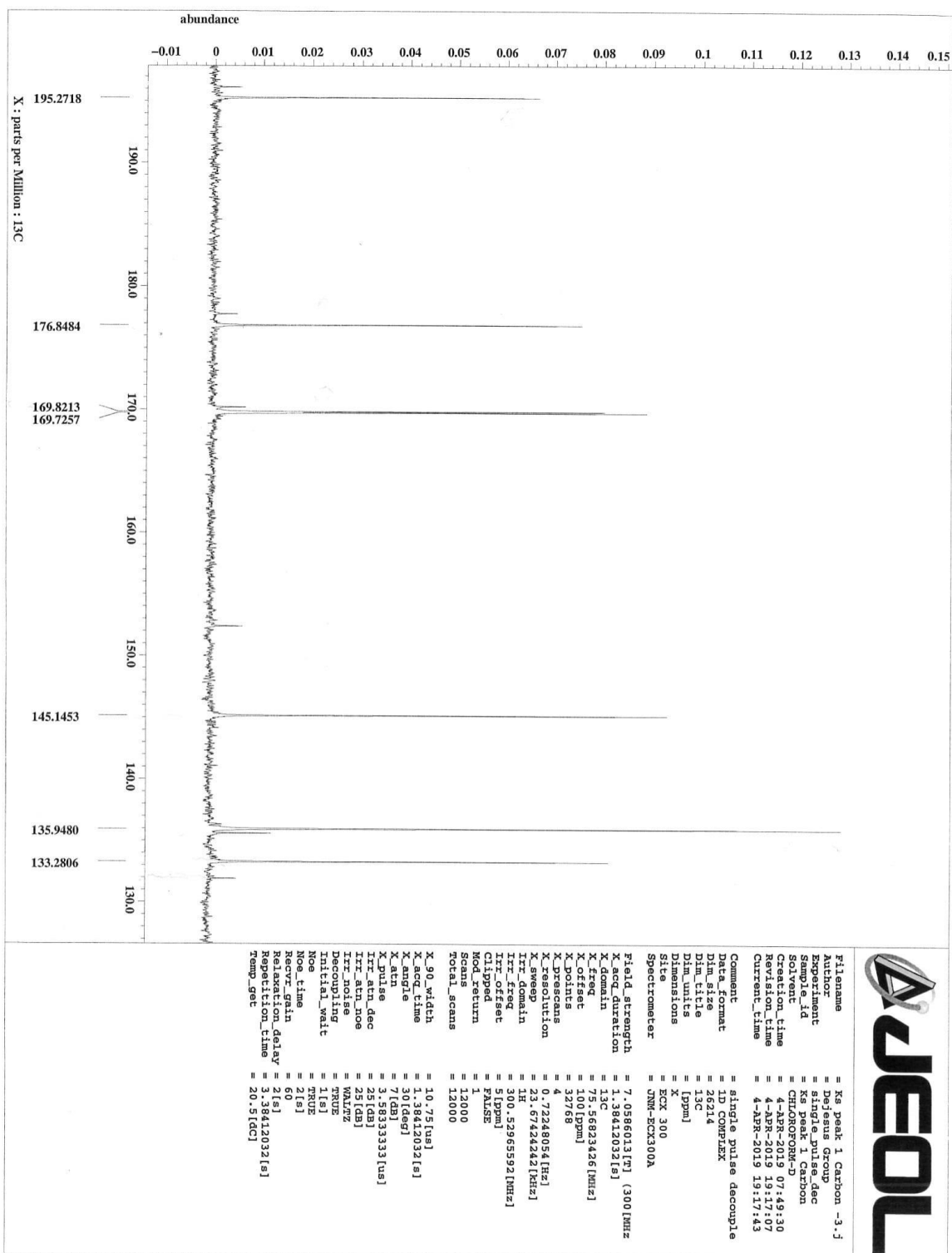

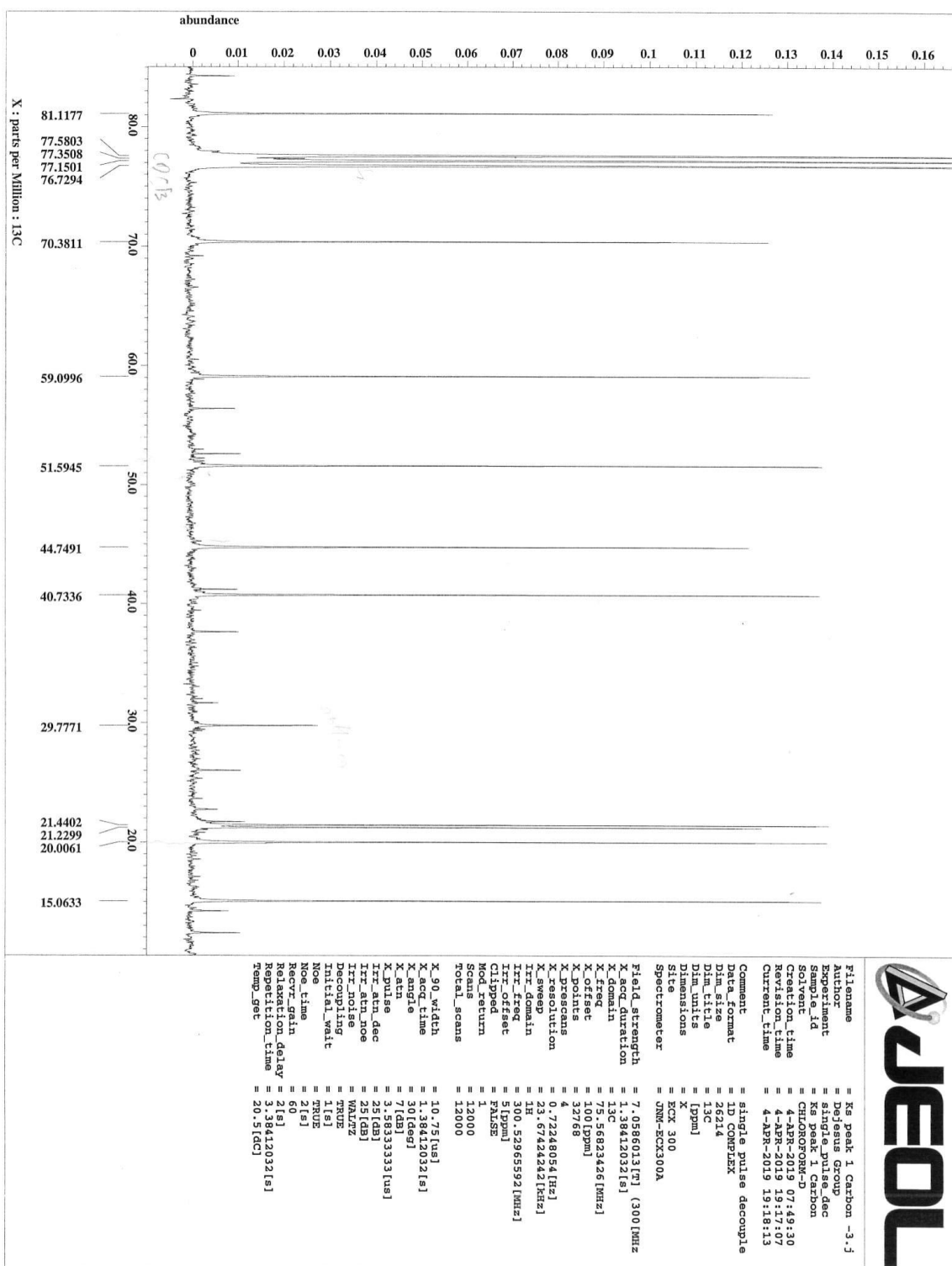

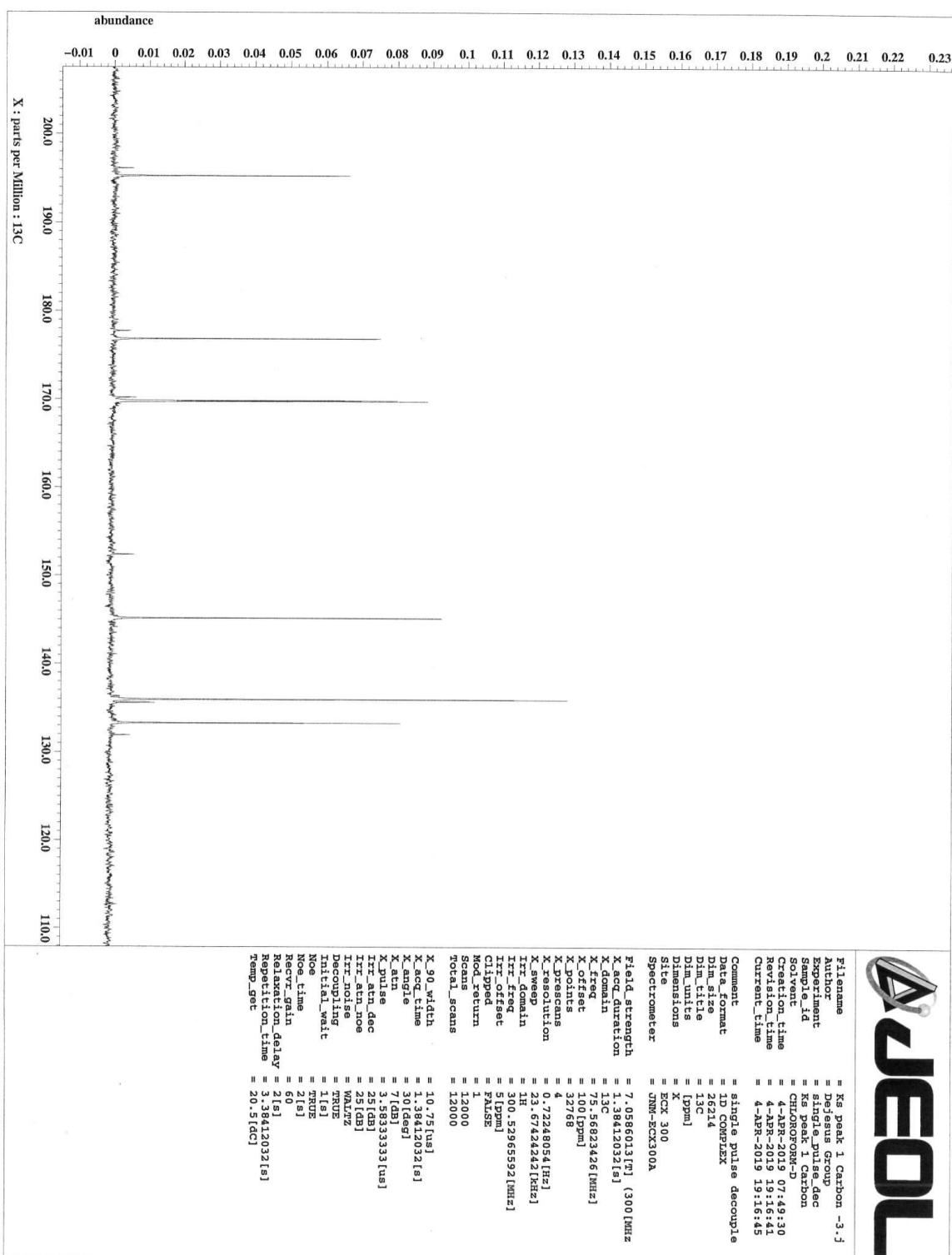

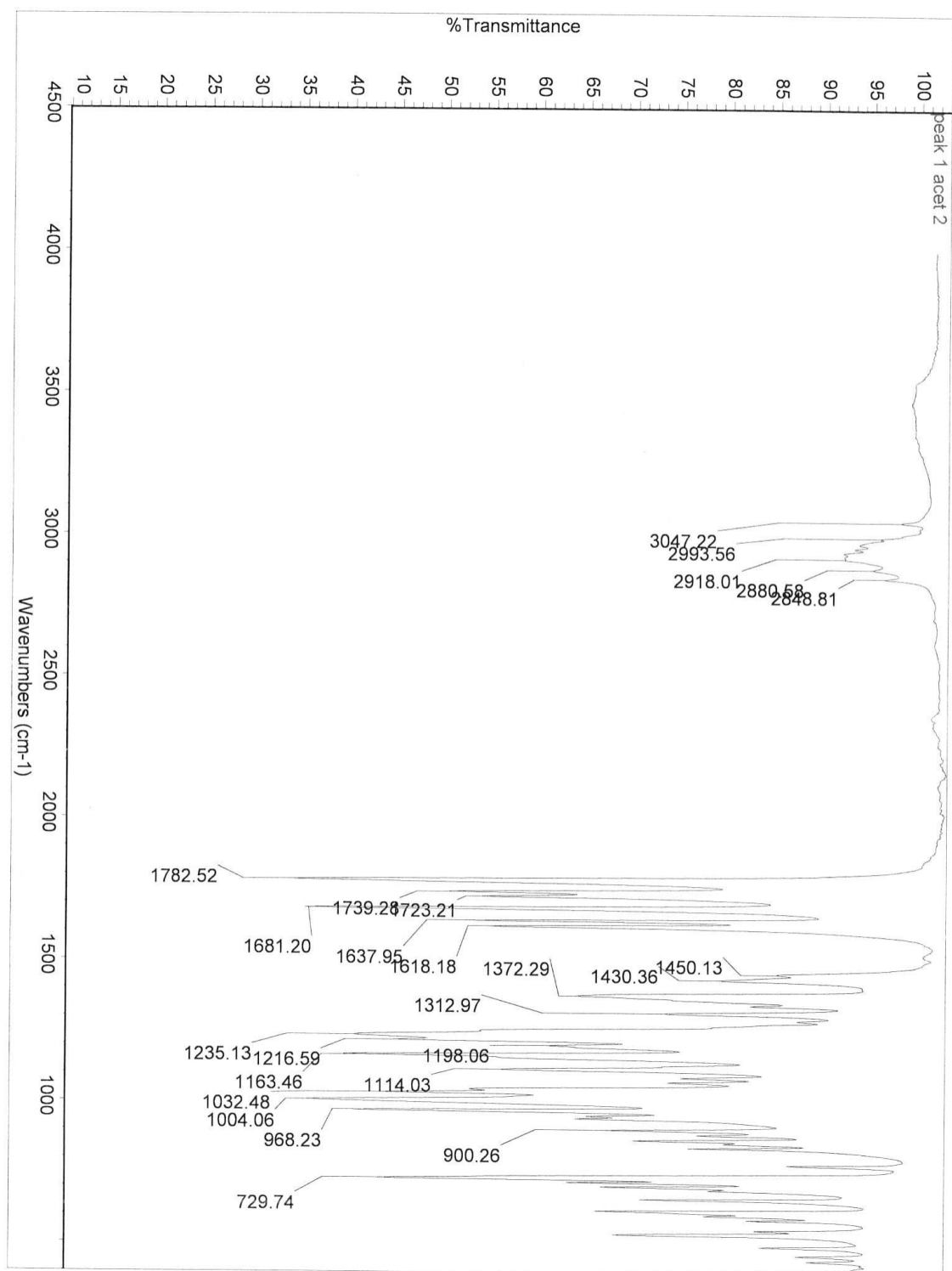

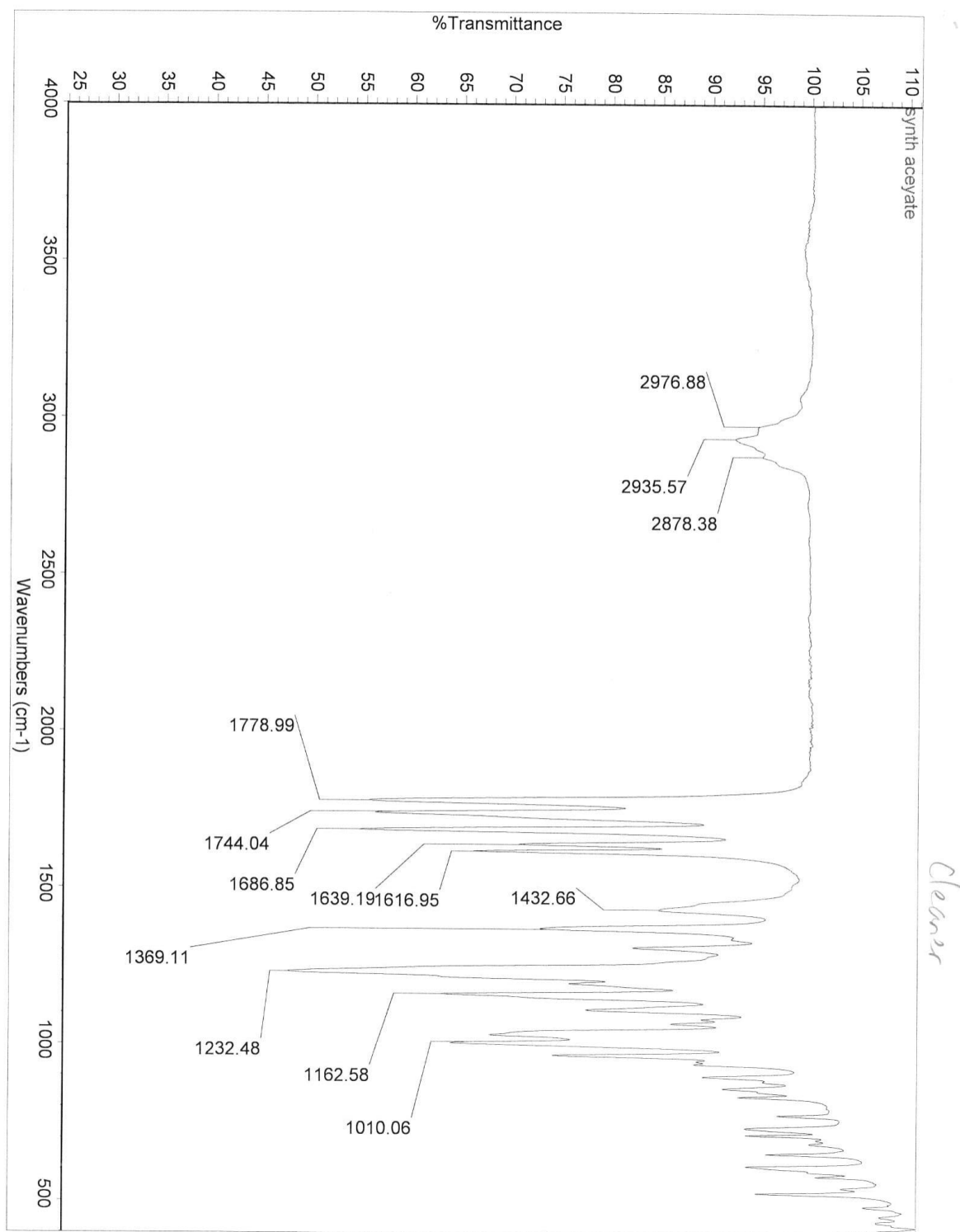

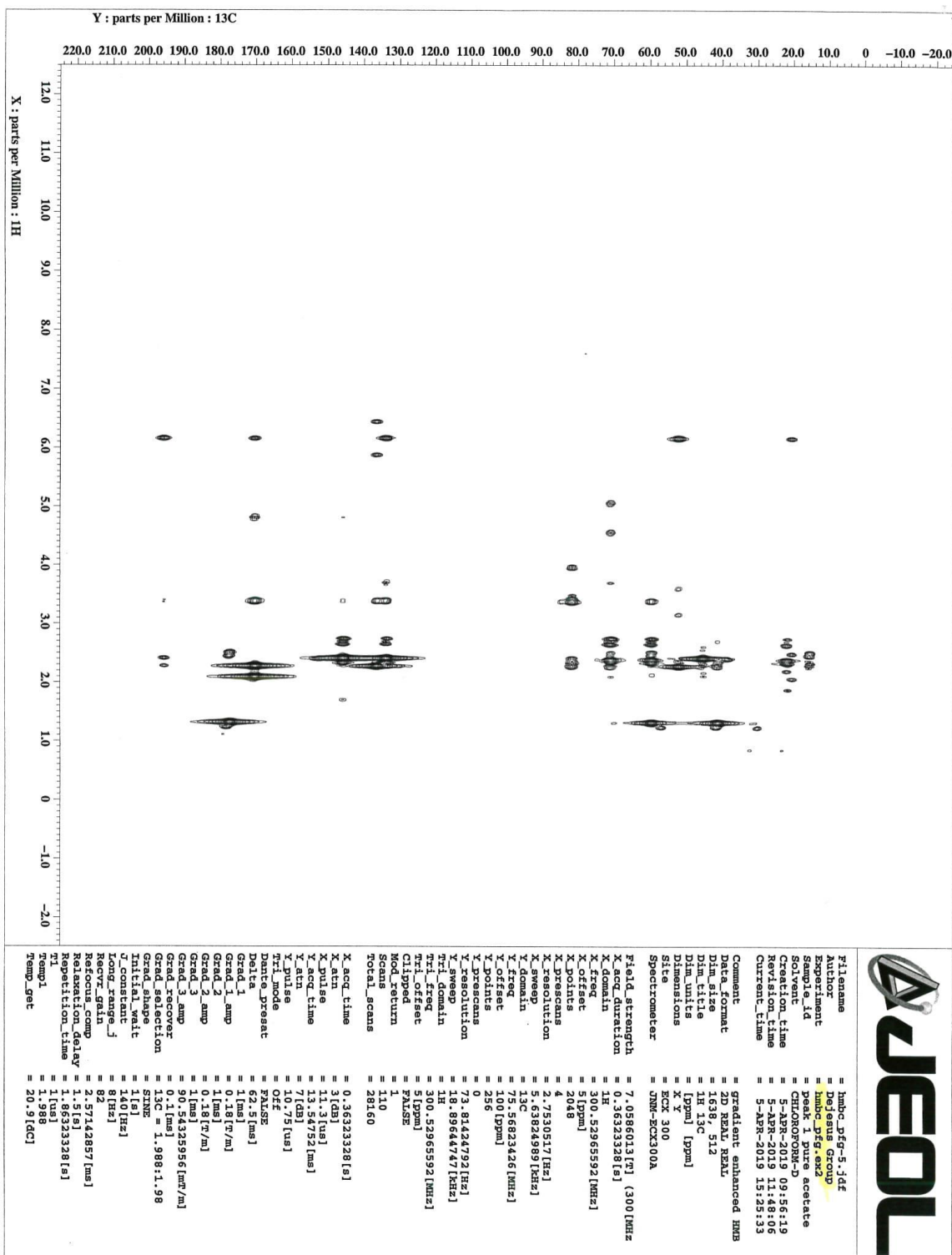

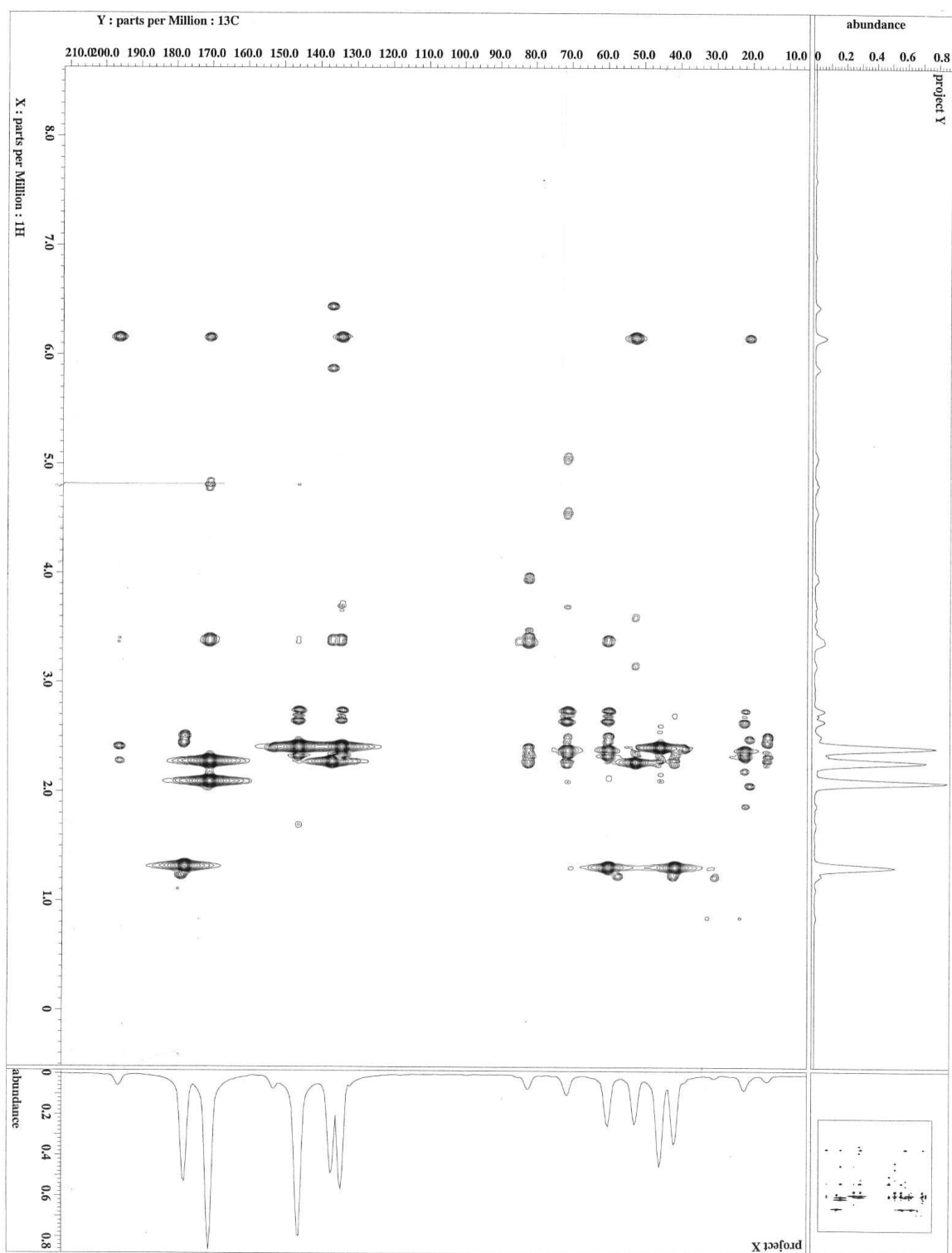

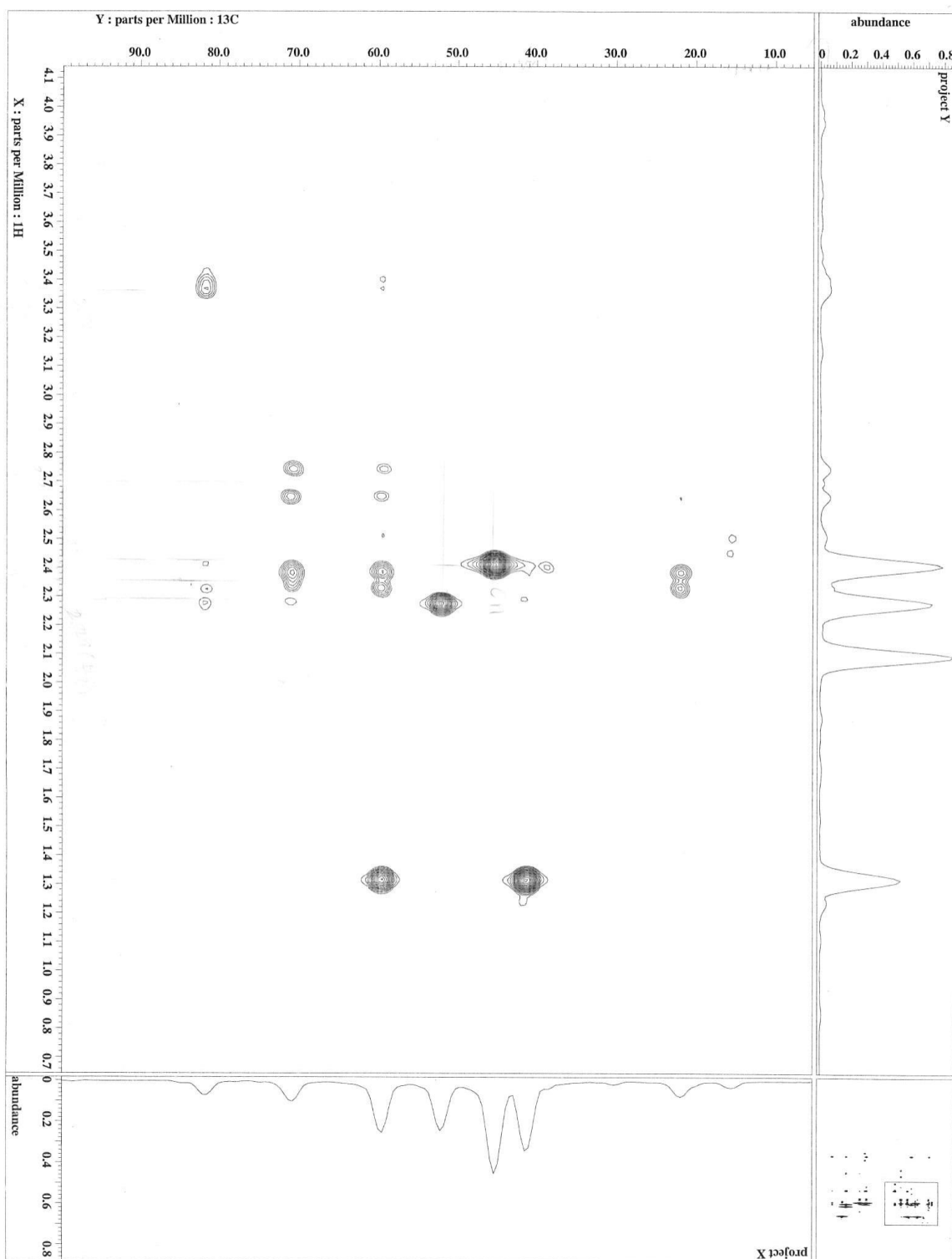

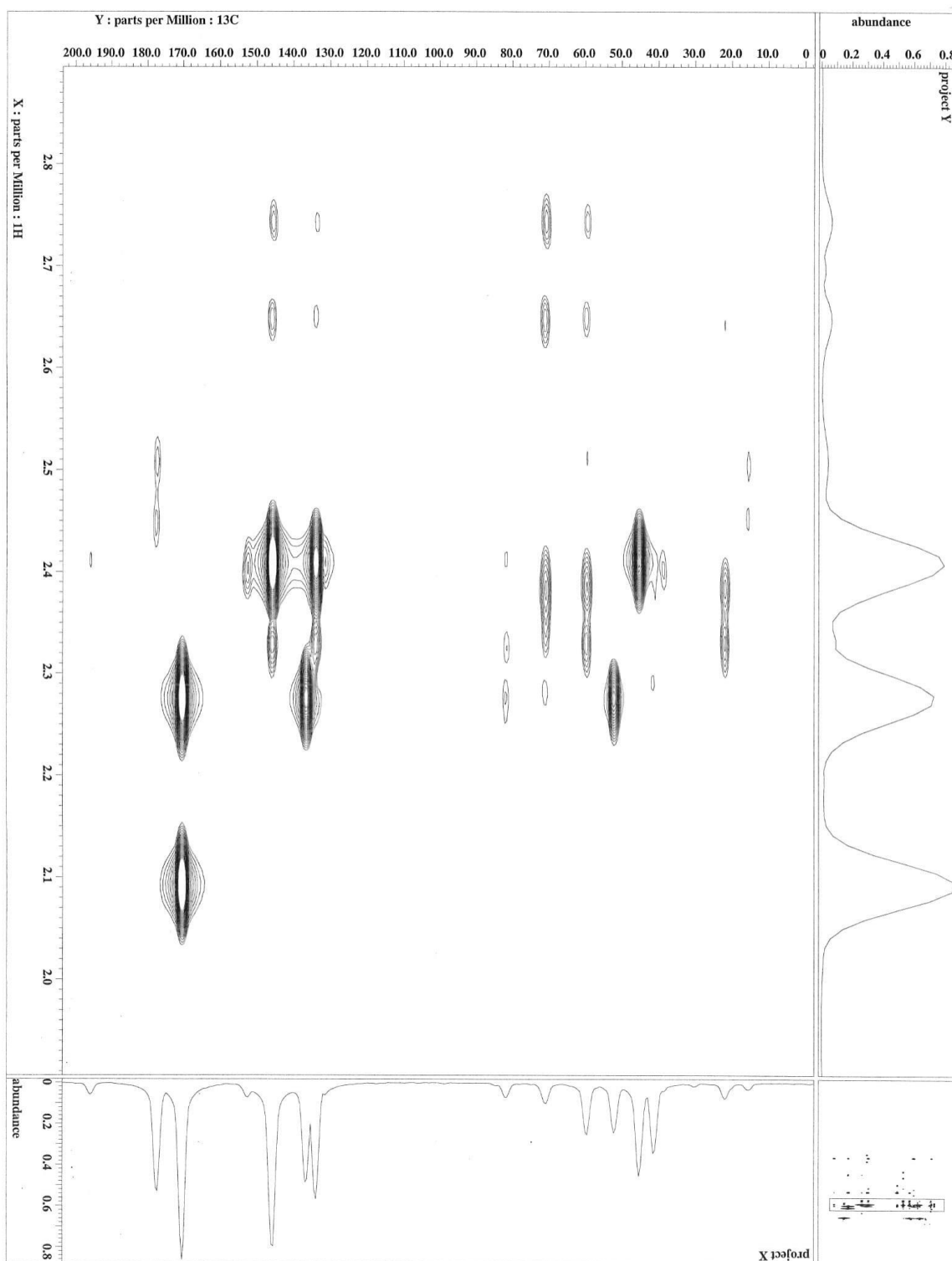

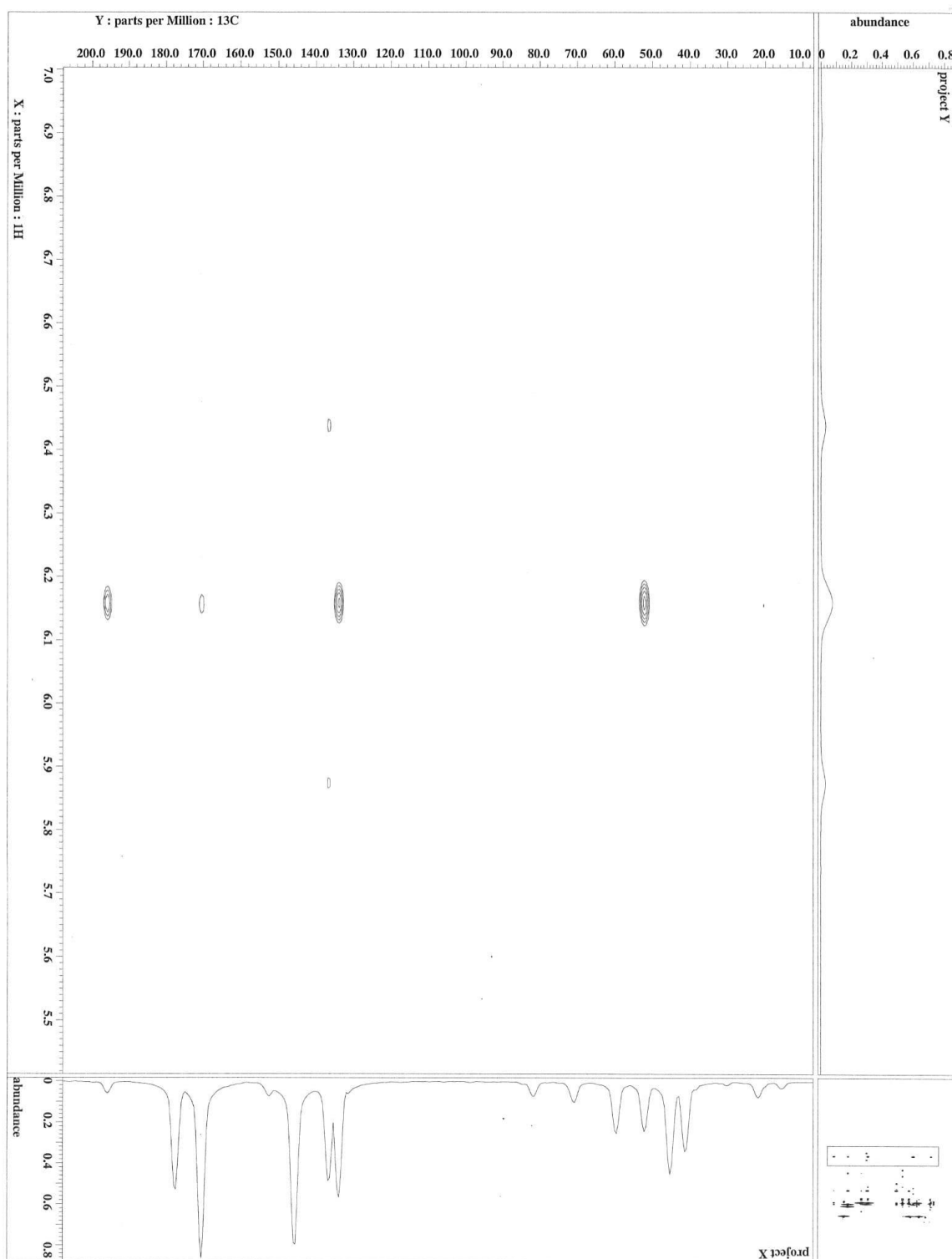

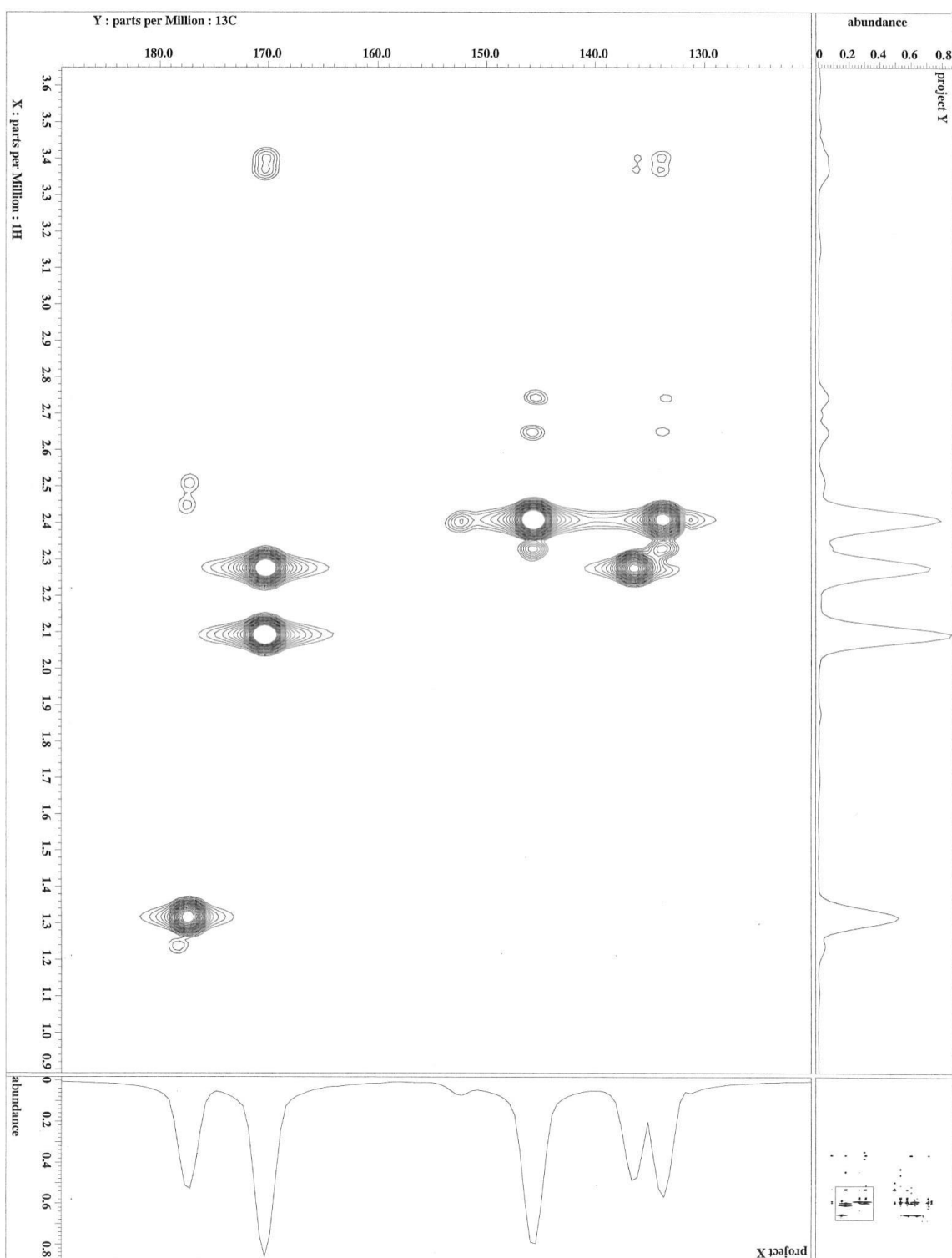

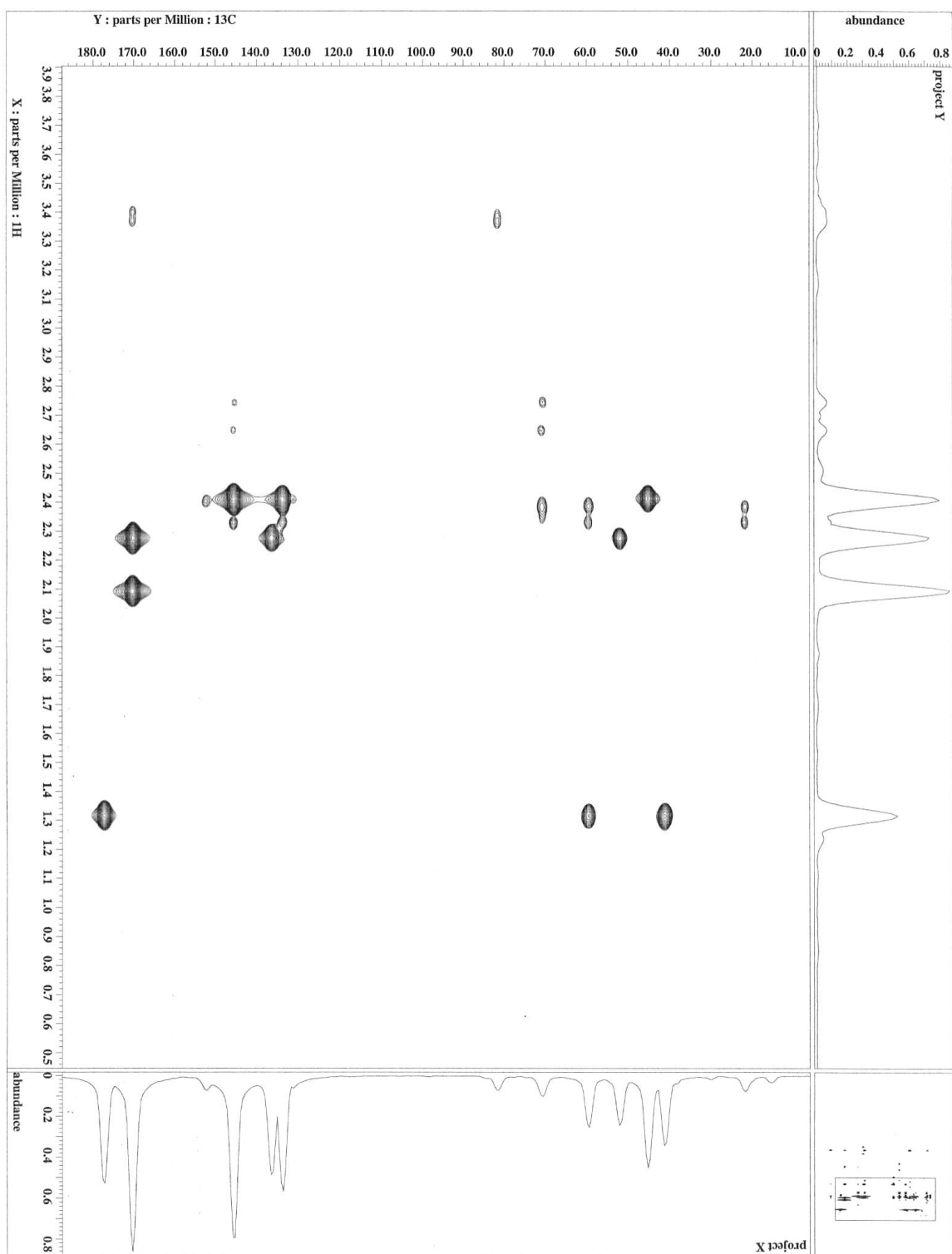

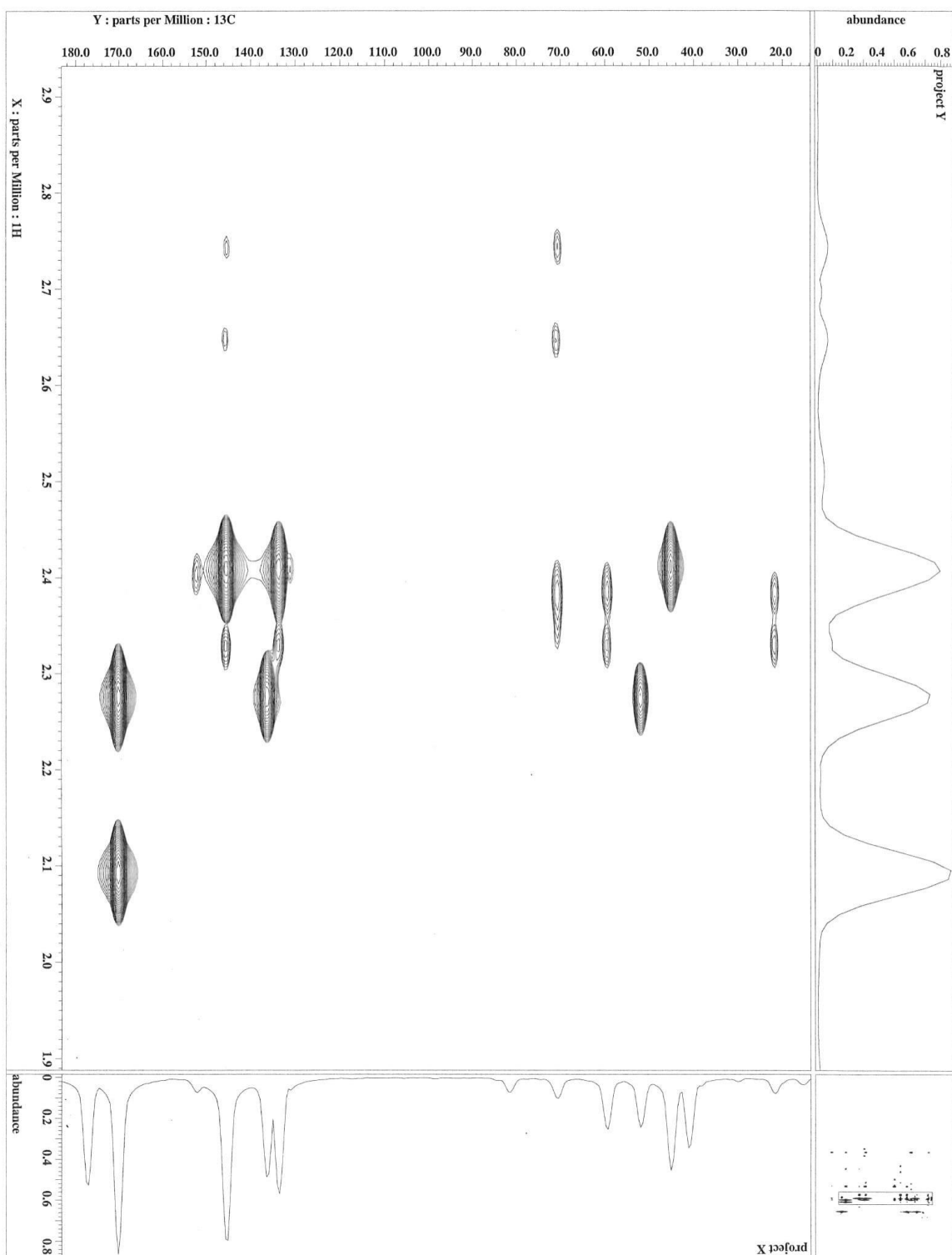

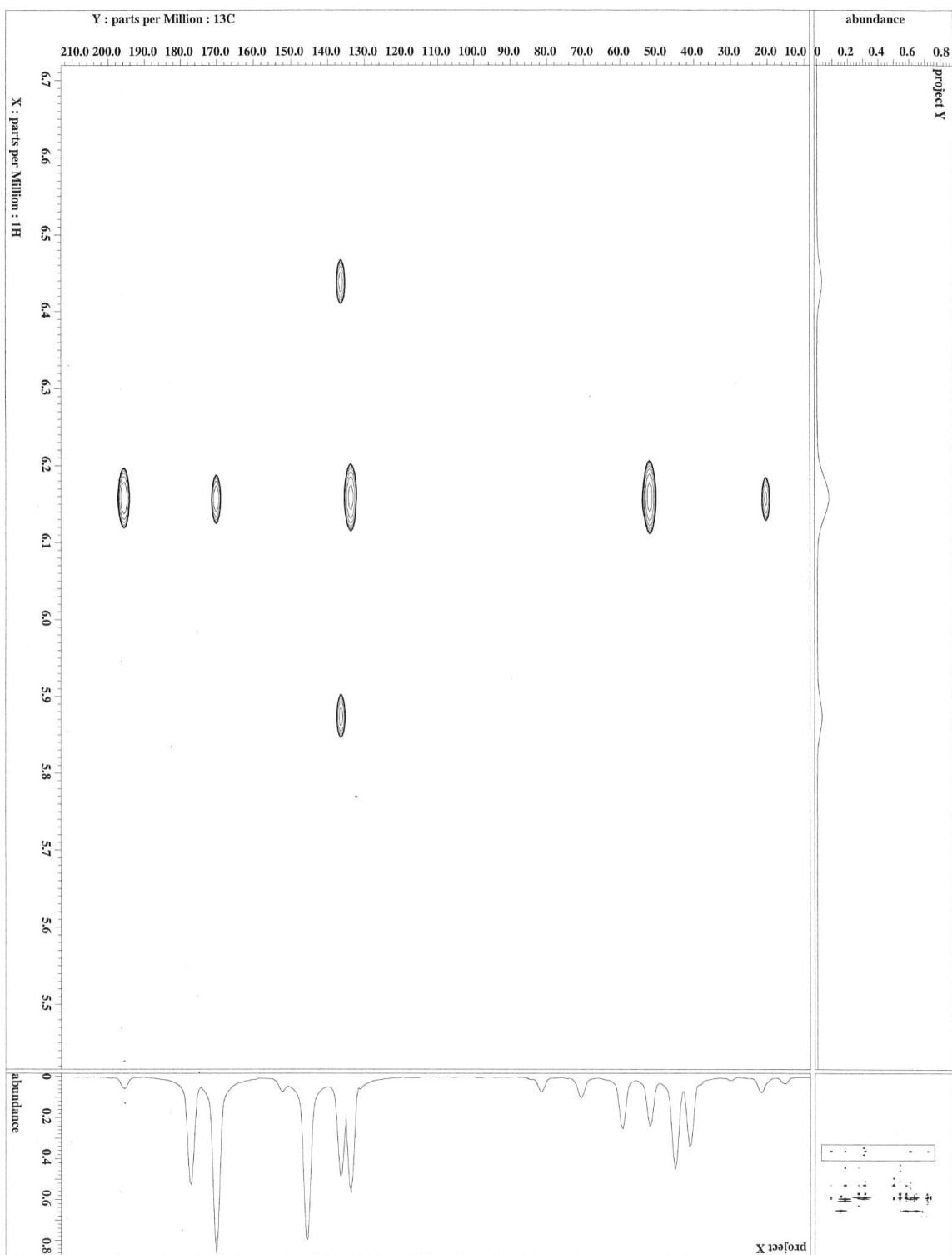

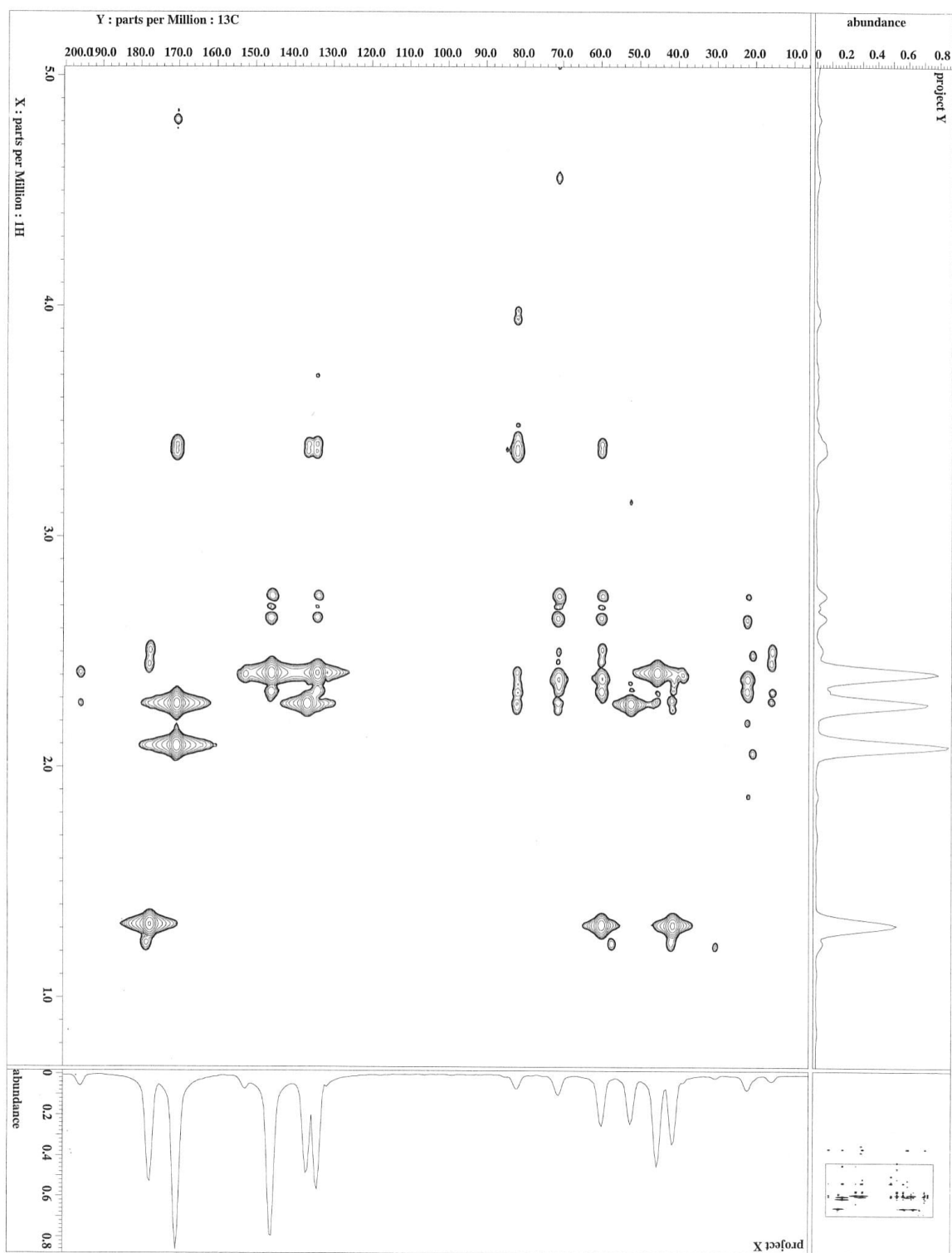

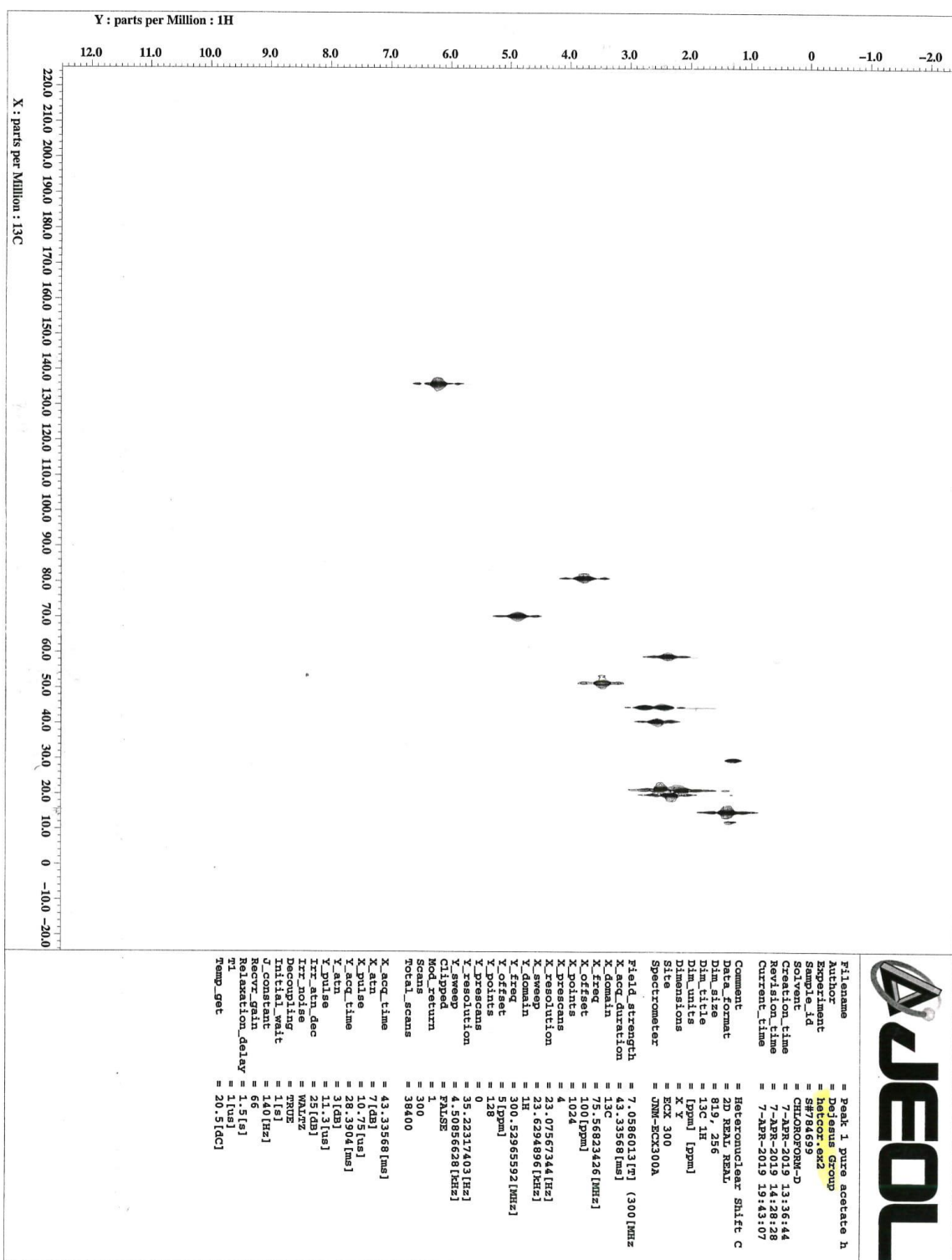

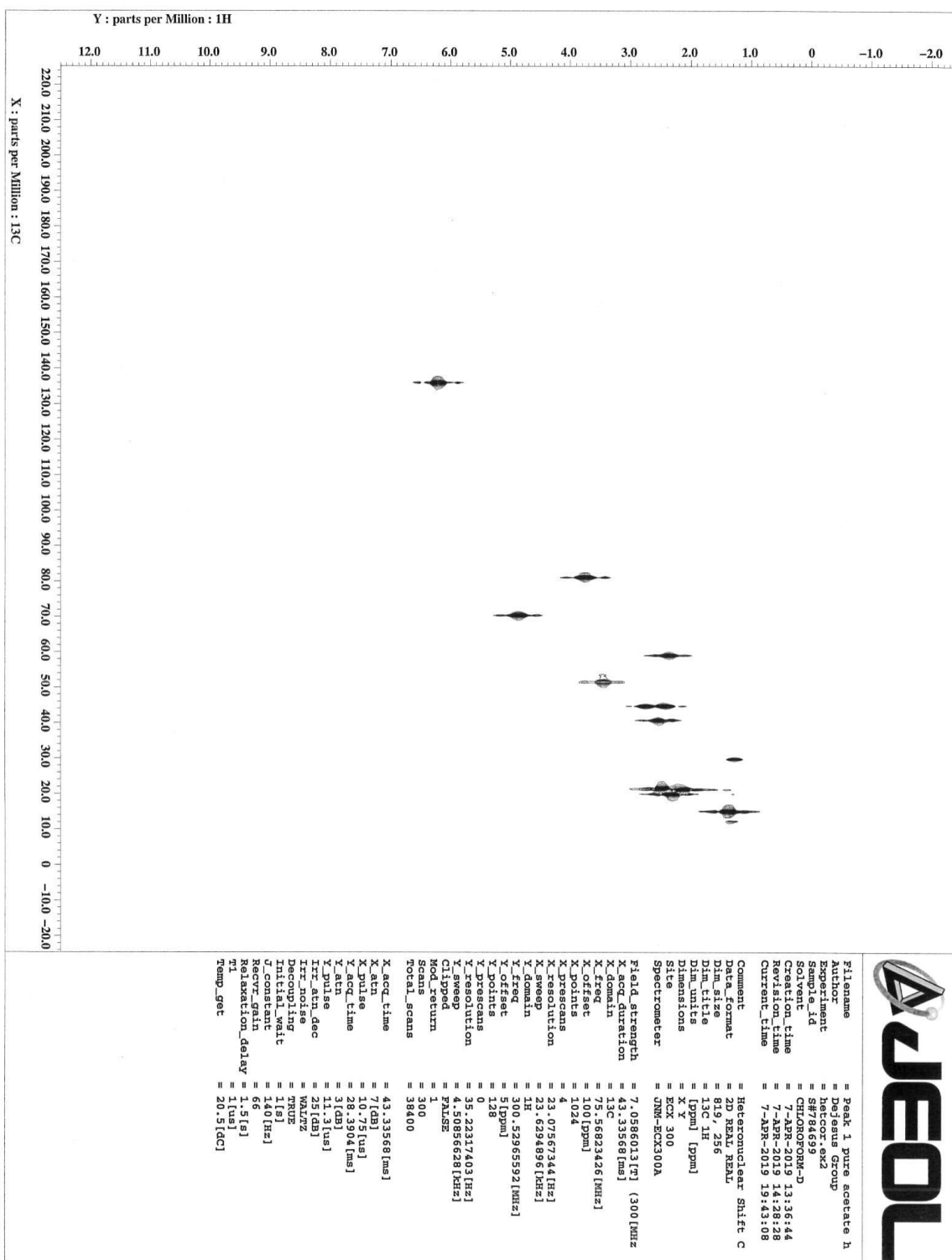

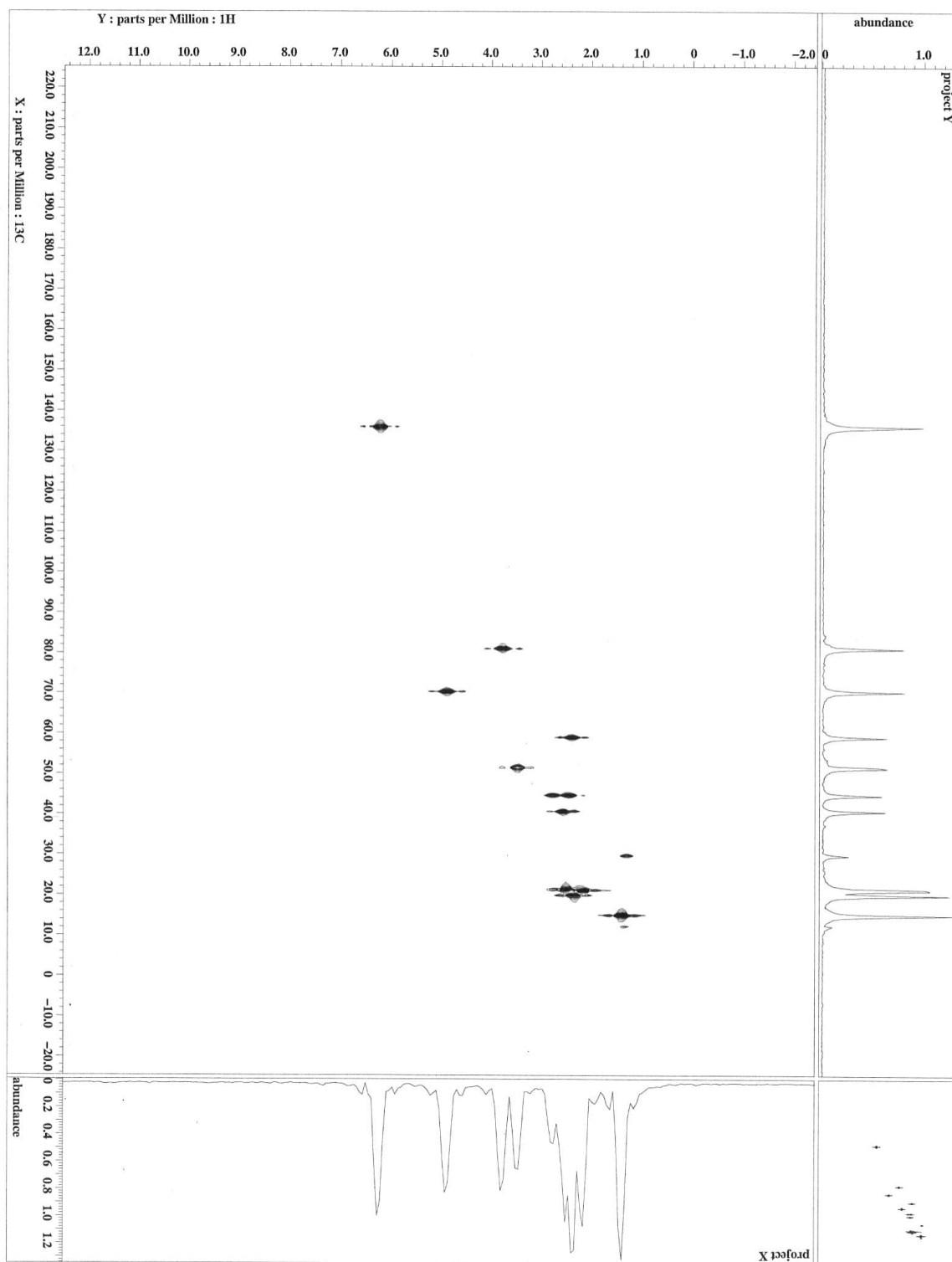

Supplement: Supplementary file 1 [file molecules-29-00802-s001.zip › molecules-2800749-supplementary/Supplementary Data/Supplementary Data-4a-NMR-Scanned Files/Matricarin NMR.pdf]

KS-I-23 peak 4 pure  
KS-I-23 peak 4 pure

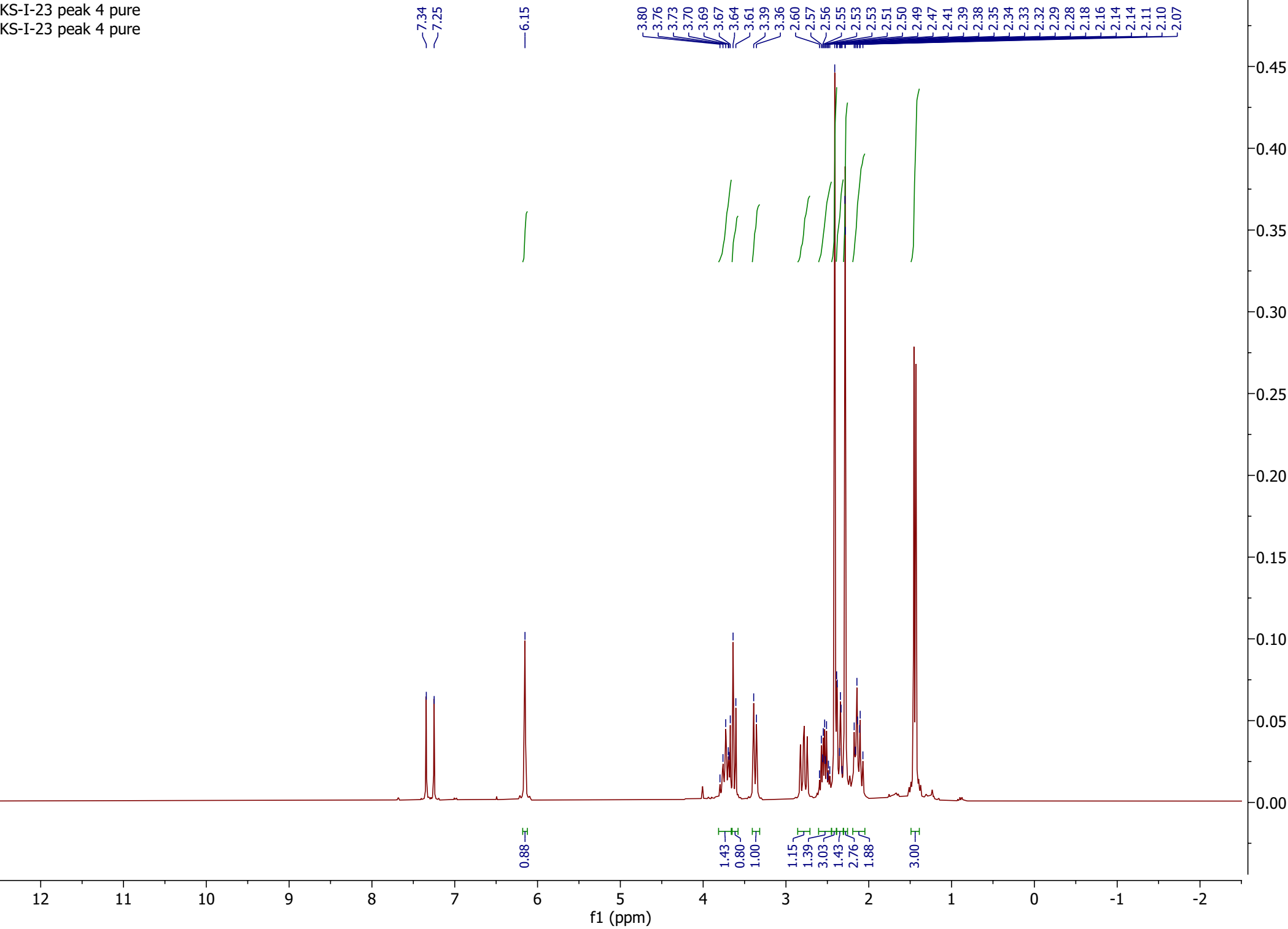

Supplement: Supplementary file 1 [file molecules-29-00802-s001.zip › molecules-2800749-supplementary/Supplementary Data/Supplementary Data-4b-NMR-Raw Data/Leucodin/Leucodin.pdf]

Predicted <sup>1</sup>H NMR Spectrum

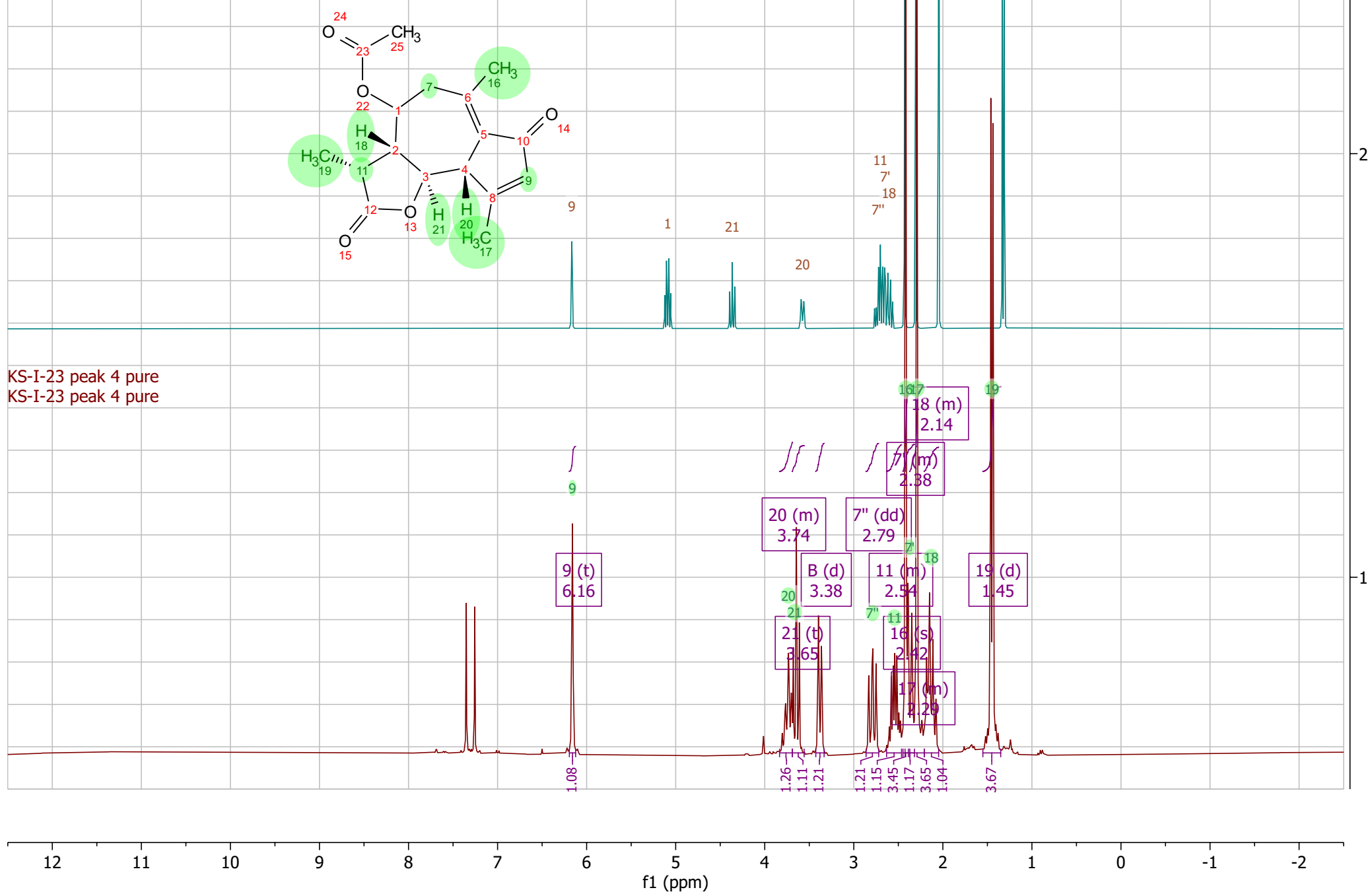

Supplement: Supplementary file 1 [file molecules-29-00802-s001.zip › molecules-2800749-supplementary/Supplementary Data/Supplementary Data-4b-NMR-Raw Data/Matricarin/Matricarin prediction.pdf]
